# Peptide macrocyclisation *via* intramolecular interception of visible-light-mediated desulfurisation†

**DOI:** 10.1039/d3sc05865d

**Published:** 2024-05-14

**Authors:** Frances R. Smith, Declan Meehan, Rhys C. Griffiths, Harriet J. Knowles, Peiyu Zhang, Huw E. L. Williams, Andrew J. Wilson, Nicholas J. Mitchell

**Affiliations:** a School of Chemistry, University of Nottingham, University Park Nottingham NG7 2RD UK nicholas.mitchell@nottingham.ac.uk; b School of Chemistry, University of Leeds Woodhouse Lane Leeds LS2 9JT UK; c Biodiscovery Institute, University of Nottingham, University Park Nottingham NG7 2RD UK; d School of Chemistry, University of Birmingham Edgbaston Birmingham B15 2TT UK

## Abstract

Synthetic methods that enable the macrocyclisation of peptides facilitate the development of effective therapeutic and diagnostic tools. Herein we report a peptide cyclisation strategy based on intramolecular interception of visible-light-mediated cysteine desulfurisation. This method allows cyclisation of unprotected peptides in an aqueous solution *via* the installation of a hydrocarbon linkage. We explore the limits of this chemistry using a range of model peptides of increasing length and complexity, including peptides of biological/therapeutic relevance. The method is applied to replace the native disulfide of the peptide hormone, oxytocin, with a proteolytically/redox-stable hydrocarbon, and internal macrocyclisation of an MCL-1-binding peptide.

## Abstract 2

Herein, we report a peptide cyclisation strategy *via* intramolecular interception of cysteine desulfurisation. This method enables the cyclisation of unprotected peptides in aqueous solution *via* the installation of a hydrocarbon linkage.

## Graphical abstract

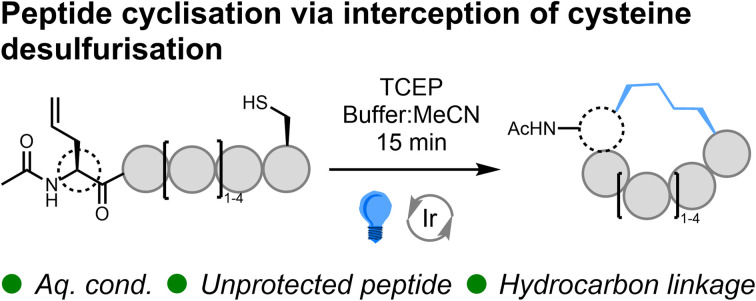

## Introduction

Despite increasing interest in peptide therapeutics within the pharmaceutical industry over the past three decades, relatively few peptides have made it through pre-clinical development to challenge the dominance of small-molecule pharmaceuticals and protein-based biologics.^1^ This is primarily due to the low membrane permeability and poor stability of linear peptides.^2^ Conversely, constrained macrocyclic peptides display enhanced stability relative to their linear counterparts and higher binding affinities due to a rigid conformation. Furthermore, macrocyclic peptides offer an effective tool to enable selective interference of the myriad of traditionally intractable protein–protein interactions (PPIs) that mediate cellular biochemistry.^3,4^ Thus, this modality of therapeutic bridges the gap between the advantageous physicochemical properties of small molecules and the exceptional activity, specificity, and bioavailability inherent to protein-based biologics. While numerous synthetic methods have been developed to enable effective peptide macrocyclisation, biocompatible chemistries that work with readily accessible building blocks in environmentally acceptable solvents are still required to enable the significant impact of macrocyclic peptides as a next-generation drug discovery tool to be realised.

**Fig. 1:**
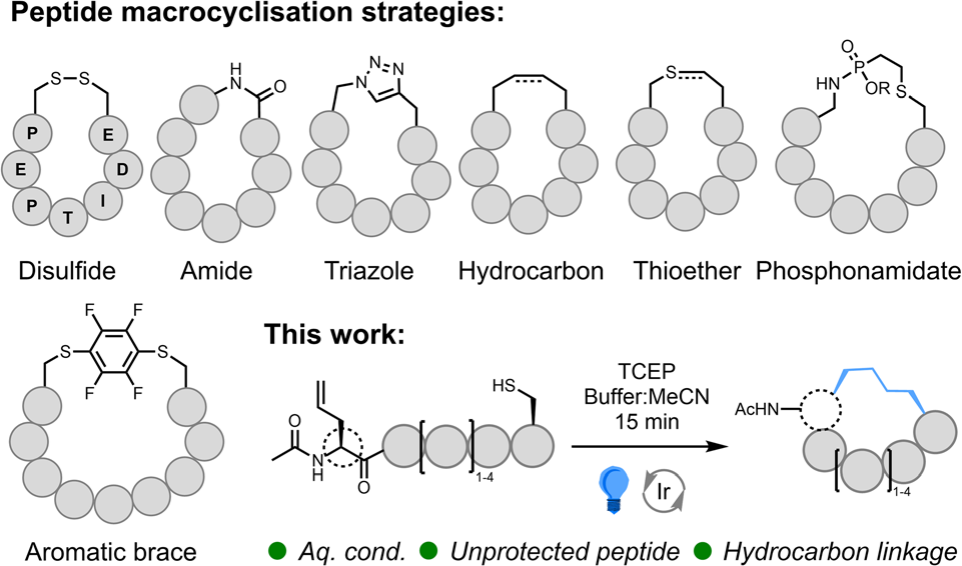
Commonly applied peptide macrocyclisation strategies; interception of desulfurisation as an approach to macrocyclisation of unprotected peptides.

Many of the synthetic methods developed to cyclise peptides^5–8^ take inspiration from nature and utilise the canonical residues, cysteine (Cys) and lysine (Lys), or the native N- and C-terminal functionality to form disulfide,^5^ thioether,^9,10^ and amide bonds (Fig. 1). Cyclisation *via* amide bond formation has been applied using both synthetic methods (peptide ligation^11–13^) and biological approaches exploiting intein chemistry (*e.g.*, SICLOPPS^14,15^), mRNA display,^10^ and ligase enzymes.^16^ Due to the nucleophilicity of the thiol sidechain, numerous Cys-selective reactions (beyond disulfide and thioether formation) have been developed/repurposed for peptide cyclisation. These include the formation of bicycles^17^*via* alkylating scaffolds,^18–21^ bridging Cys residues using perfluoroaryl braces/fluorine displacement^22,23^ (and alternative bridging groups^24,25^), thiol coordination to bismuth,^26^ thiol-addition chemistry,^27,28^ and desulfurative replacement of a disulfide bridge.^29^ Incorporation of non-standard amino acids enables the exploitation of bioorthogonal chemistry such as azide–alkyne cycloaddition^30,31^ (*i.e.*, ‘Click Chemistry’), Staudinger ligation^32^ and azide-phosphonite chemistry.^33^ Simple imine^34^ and oxime^35^ bond formation has been utilized, as well as more complex transition metal (TM)-catalysed^36–41^ and multi-component chemistry.^42^ Radical reactions such as thiol–ene^43–45^ and, more recently, decarboxylative photoredox catalysis^46^ and C–H alkylation^47^ have also been successfully applied. However, among the broad range of available synthetic techniques, Grubbs' ruthenium (Ru)-catalysed ring-closing olefin metathesis (RCM)^48^ reaction has found global application as a method-of-choice for peptide cyclisation due to effective formation of a proteolytic, hydrolytic, and redox-stable hydrocarbon linkage.^49–53^ Whilst an undeniably powerful method, RCM is usually conducted on protected peptides in organic solvent, and access to the saturated hydrocarbon necessitates reduction under harsh conditions. New, operationally simple techniques that retain the benefits of RCM, but that work effectively in more sustainable solvents, would offer an impactful alternative to this universally popular method.

We recently reported a novel strategy for the site-selective modification of peptides and proteins *via* visible-light-mediated desulfurative C(sp^3^)–C(sp^3^) bond formation.^54,55^ Desulfurisation of Cys (and alternative non-proteinogenic thiol-containing amino acids^56–58^) can be applied post-peptide ligation as an elegant method to access a broad range of ligation junctions and facilitate chemical protein synthesis.^56,59–67^ A widely used free-radical-mediated Cys desulfurative protocol,^59^ developed by Danishefsky and co-workers, proceeds *via* a thiophosphoranyl radical species generated using a radical initiator (VA-044) to form a thiyl radical from the thiol sidechain of Cys in the presence of the water-soluble phosphine, tris(2-carboxyethyl)phosphine hydrochloride (TCEP). β-Scission of the thiophosphoranyl radical^62^ produces a peptide ‘alanyl’ radical which, in the presence of a suitable thiol additive (*e.g.* glutathione), will abstract an H-atom to yield the residue, alanine (Ala), at the ligation junction.^59^ In previous work, we demonstrated desulfurisation using an iridium(iii) photocatalyst (PC) and employed alkenes to intercept the alanyl radical species, enabling installation of Lys sidechains carrying natural modifications as well as effective mimics of this modified sidechain.^54,55^ This reaction is initiated *via* excitation of the Ir(iii) PC by a photon of visible light. The activated catalyst is then reduced by the thiol group producing a thiol radical cation which forms a thiyl radical on deprotonation. In the presence of TCEP, the thiophosphoranyl radical is formed; β-scission of this species produces the ‘alanyl’ radical^60^ which is trapped by the alkene. Due to the requirement to out-compete H-atom abstraction during this process, a large excess of the alkene is required (a minimum of 200 equivalents). By installing an appropriate alkene into a peptide containing a Cys residue, we postulated that intramolecular trapping of the radical produced upon desulfurisation may proceed preferentially to H-atom abstraction, essentially allowing us to use an equimolar equivalent of the alkene (Fig. S1†). The cyclic peptide radical produced during this reaction will be quenched to form the macrocyclic product; H-atom transfer (HAT) from the thiol group of remaining starting peptide is a likely pathway. The resulting thiyl radical can then continue the cycle or be reduced by the catalyst and protonated.^68^ Oxygen in the buffer has also been identified as an oxidant for the catalyst during Ru-mediated desulfurisation.^60^

The chemoselectivity of this chemistry should ensure that the reaction enables efficient cyclisation of unprotected peptides in aqueous solution. If realised, this technique would be a valuable addition to the toolkit available to researchers for the production of cyclic peptides.

## Results and discussion

### Optimisation of reaction conditions and radical trap exploration

Initial trials of this strategy focused on the utilisation of both 2-methylallyl and allyl moieties to facilitate peptide cyclisation in the presence of a Cys residue (Table 1). Addition of the alanyl radical to a 2-methylallyl group would generate a tertiary radical intermediate previously shown to improve the efficiency of this chemistry;^55^ however, diastereomers of the desired macrocycle will be produced due to the methyl branch of the linkage. Employing an allyl group as the radical trap would result in a single product, however, the reaction will be less effective and may not fully out-compete H-atom abstraction. Both of these strategies were initially explored. Amino acid building blocks 1 and 2 were synthesised *via* alkylation of Boc-Ser-OH with 1-bromo-3-methylbut-3-ene and allyl bromide, respectively (ESI†). These residues were incorporated into a simple peptide sequence using solid phase peptide synthesis (SPPS) to afford peptides 3 (H–S(*O*-2-methylallyl)AFAC-NH_2_) and 4 (H–S(*O*Allyl)AFAC-NH_2_). Both peptides (3/4, 0.5 mM) were subjected to desulfurisation conditions in 9 : 1 conjugation buffer (6 M Gdn·HCl, 0.1 M Na_2_HPO_4_, pH 7.5–8.0):acetonitrile (MeCN) in the presence of a phosphine (TCEP; 5 mM) and an Ir(iii) PC (5 mol%, (Ir[dF(CF_3_)ppy]_2_(dtbpy))PF_6_), under irradiation of blue light (450 nm) using inexpensive blue LED light strips (see ESI† for details). The reaction progress was monitored using analytical HPLC. Excess phosphine was employed to ensure that the thiyl radical did not interfere with the reaction *via* thiol–ene radical addition.^45^ Promisingly, we observed complete and clean conversion of the starting peptide 3 within 10 min. As anticipated, two products were observed, each with the same mass. Purification of the material *via* preparative HPLC was followed by NMR analysis which determined that the two products were diastereomers of the desired cyclised peptide, isolated in an excellent combined yield of 77% (5a/5b, 53% and 24%, respectively) (Table 1, entry 1). No resonances were observed corresponding to the allyl group in the ^1^H NMR spectrum, which precludes the misidentification of the desulfurised starting peptide as the product; a concern considering these structures share the same molecular weight.

**Table 1:** Exploration of peptide cyclisation *via* intramolecular interception of visible-light-mediated desulfurisation

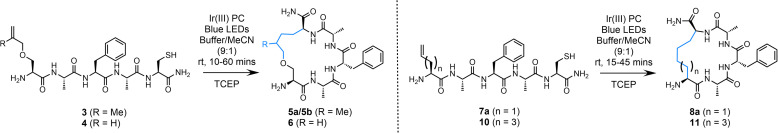						
Entry	Peptide	[Peptide]^a^/mM	[TCEP]/mM	Ir(iii) PC (mol%)	Reaction duration^b^ (min)	Isolated yield^c^ [% conversion^d^]
1	3	0.5	5	5	10	77%^e^ (*dr* 69 : 31)^f^
2	4	0.5	5	5	60	[72%]
3	4	0.5	2.5	5	60	[38%]
4	4	0.5	5	1	60	[36%]
5	4	0.5	25	5	60	78%
6^g^	7a	0.5	25	1	45 [15]^h^	79%
7	10	0.5	25	1	60	32%

a Reaction conducted in 10% acetonitrile (MeCN)/6 M Gdn·HCl, 0.1 M Na_2_HPO_4_, pH 7.5–8.0.

b LED strips, photochem set up 1 (ESI†).

c Peptide products isolated by preparative HPLC.

d % Conversion calculated *via* analytical HPLC.

e Combined yield from both diastereomers.

f Diastereomeric ratio calculated *via* analytical HPLC.

g No reaction observed in the absence of blue light.

h Reaction completed in 15 min using a PhotoRedOx box (photochem set up 2 – ESI†).

Quantitative conversion of the starting sequence to cyclised product, with negligible formation of the linear desulfurised by-product (formed *via* the alanyl radical abstracting an H-atom), is a gratifying initial result that allows high-yielding and rapid access to macrocyclic peptides. However, the formation of diastereomers is not ideal. Peptide 4 carries an allyl group as the radical trap; while this moiety would generate a less stable secondary radical upon addition of the alanyl radical, our previous results gave us confidence that intramolecular trapping should still out-compete H-atom abstraction. Under the conditions described, the starting peptide (4) was consumed within 60 min leading to the production of a major product (72% conversion to product by analytical HPLC, entry 2). It was noted that the reaction was equally effective without using degassed buffer; therefore, this step was omitted from the protocol. Prior to scaling the reaction up for isolation, a brief optimisation study was undertaken. It was observed that a reduction in the equivalents of TCEP led to a dramatic decrease in conversion to the product (Entry 3; 38% conversion to product 6), as did decreasing the mol% loading of the PC from 5 to 1 mol% (entry 4). When scaling up to an isolable yield it was observed that the conditions detailed in entry 2 were not optimal; an increase in the equivalents of TCEP to 50 (25 mM) was necessary to maintain high conversion to product. Using these adjusted conditions (entry 5) the desired product (6) was isolated in 78% yield and characterised *via* MS and NMR spectroscopy. The remaining mass balance for these reactions was the linear desulfurised by-product. No peptide degradation or epimerisation was observed over the course of the reaction.

**Fig. 2:**
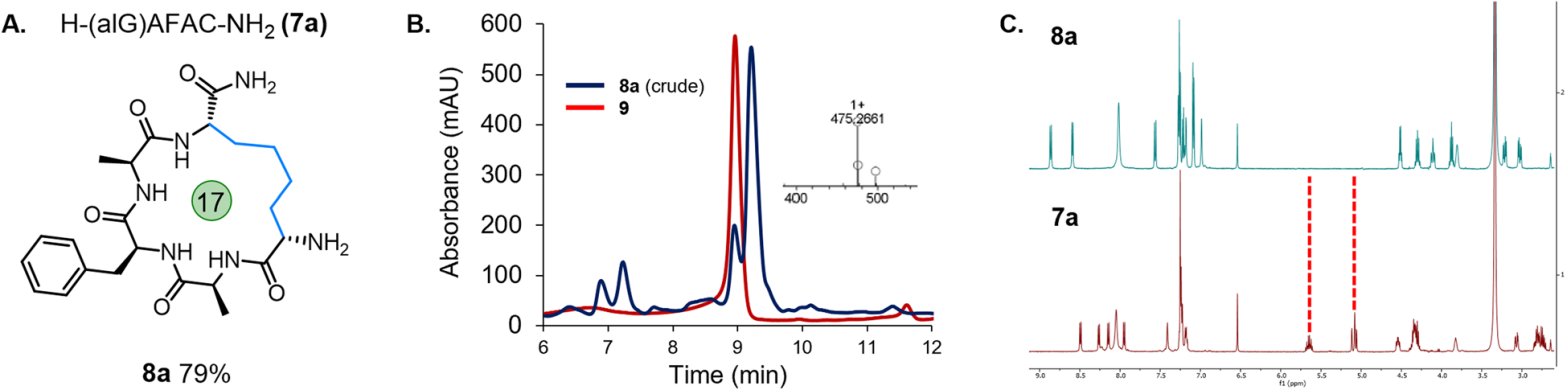
(A) Cyclised peptide 8a from H-(alG)AFAC-NH_2_ (7a); (B) analytical HPLC of the crude cyclisation of 7a (blue trace) overlaid with peptide H-(alG)AFAA-NH_2_ (9), inset – ESI MS showing the mass of the desired cyclised product (8a); (C) ^1^H NMR spectrum for linear peptide 7a (red trace) and peptide macrocycle 8a (blue trace) showing the absence of the allylic protons.

Due to the need to synthesise the allyl-protected serine (Ser) building block, a more readily accessible option was sought. The commercially available amino acid, Fmoc-allyl-Gly-OH (alGly, alG), would afford a cyclic peptide with a butyl hydrocarbon linker. Therefore, Fmoc-protected alGly was incorporated into a model peptide (H-(alG)AFAC-NH_2_; 7a) and cyclised using the optimised conditions based on entry 5, Table 1 with a decrease in the PC loading to 1 mol% (entry 6), a change which which did not hinder conversion to the product. The reaction proceeded to completion as expected over 45 min and the desired product (8a) was isolated in an excellent yield of 79% by preparative HPLC (Fig. 2). The following conditions were therefore identified as optimal: 0.5 mM peptide, 1 mol% PC, 25 mM TCEP, in 10% MeCN/6 M Gdn·HCl, 0.1 M Na_2_HPO_4_, pH 7.5–8.0 (degassing step omitted). The reaction was then repeated using a PhotoRedOx Box equipped with a 34 mW cm^−2^, 450 nm LED (HeptatoChem). The ratio of desired product to by-product remained consistent with the reaction performed using blue LED light strips, however the rate of the reaction was enhanced, reaching completion in just 15 min (Fig. S49–S54†).

**Fig. 3:**
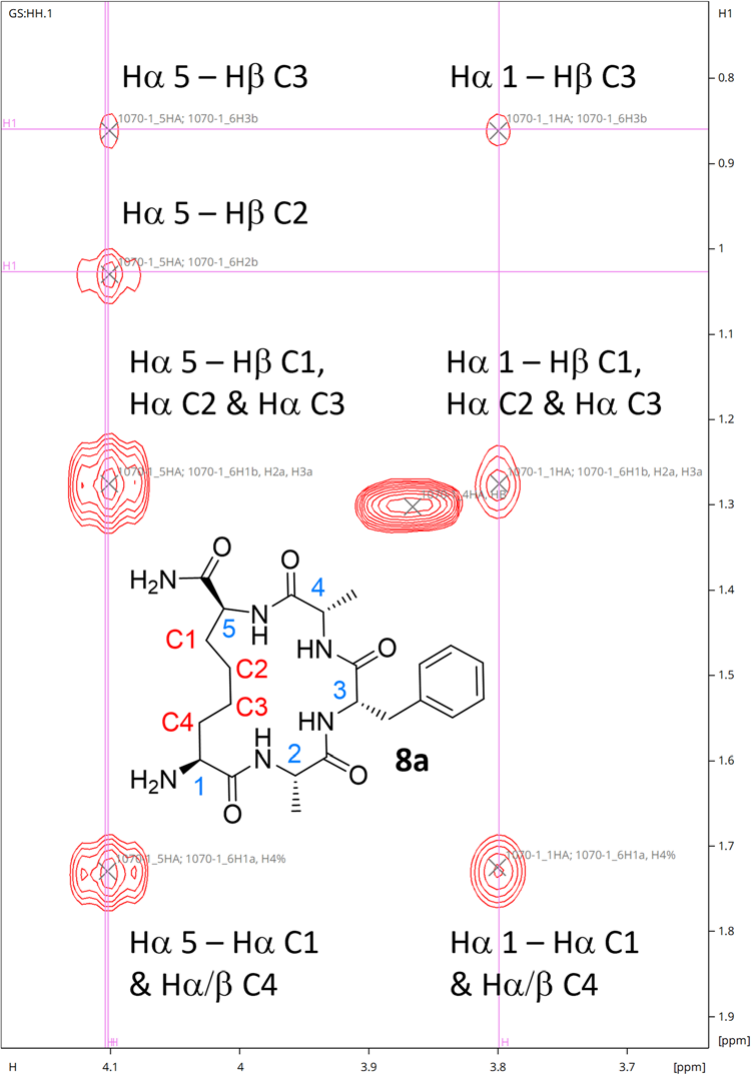
Hα region for the TOCSY NMR spectrum of 8a confirming successful formation of the macrocycle.

While ^1^H NMR analysis of 8a confirmed that the allylic protons were not present (indicating successful cyclisation) (Fig. 2), further analysis was sought to fully characterise the macrocycle for peptide 8a. A TOCSY NMR experiment was carried out on this model and a complete assignment of the macrocycle was achieved (Fig. 3). Proton environments in the hydrocarbon linker were found to couple to Hα signals on residues at each end of the macrocycle indicating successful cyclisation. Moreover, the Hα signal from what was initially the Cys residue prior to cyclisation coupled to the Hα of the first Ala residue, which could only occur as a result of macrocycle formation. A significant chemical shift dispersion suggests an ordered structure. Furthermore, the phenylalanine (Phe) Hα resonances show an NOE interaction to the Hα of the allyl glycine position, suggesting that the macrocycle is strained.

To further explore the optimised reaction, we considered buffer composition, the phosphine additive, and alternative desulfurisation conditions. Cyclisation of model 7a in PBS did not proceed cleanly (Fig. S55†), while employing HEPES buffer did lead to clean conversion of the starting peptide, however, the undesired desulfurised linear by-product was the dominant product (Fig. S56†). Thus, we conclude that cyclisation is most effective in a buffer containing a high concentration of chaotropic salt. Replacing TCEP with the water soluble phosphine, 3,3′,3′′-phosphanetriyltris(benzenesulfonic acid) trisodium salt (TPPTS) did not lead to effective conversion of the starting peptide 7a (Fig. S57†). Employing 1,3,5-triaza-7-phosphaadamantane (PTA) initially appeared to eliminate the production of the undesired linear desulfurised by-product (Fig. S58†). However, repetition of the reaction on an isolable scale and purification by preparative HPLC revealed the by-product and other impurities eluting under the broader product peak (Fig. S59–S62†) which ultimately limited the isolated yield. Finally, the peptide H-(alG)AFAC-OH (7b) was synthesised on 2-CTC resin to probe the cyclisation on peptides bearing a C-terminal carboxylic acid. While conversion to the desired product was observed (Fig. S68†), the ratio of by-product to macrocycle was less favourable when compared to C-terminal amide peptides. In addition, for this simple model, the retention times for the desired product and by-product were very similar making separation a significant challenge.

Several methods for the desulfurisation of Cys residues have been reported in the literature, these include; photo-induced desulfurisation^69^ using a ruthenium PC,^60^ photo-desulfurisation in flow,^61^ accelerated desulfurisation using tetraethylborate (NaBEt_4_),^63^ desulfurative borylation,^64^ and the exploitation of phosphite^66^ and phosphine-dependent pathways.^65^ We attempted to adapt and explore two appropriate examples.^60,63^ However, both the use of NaBEt_4_ ^63^ and a ruthenium photocatalyst^60^ failed to improve on our reported conditions (Fig. S63 and S64–S67†).

**Fig. 4:**
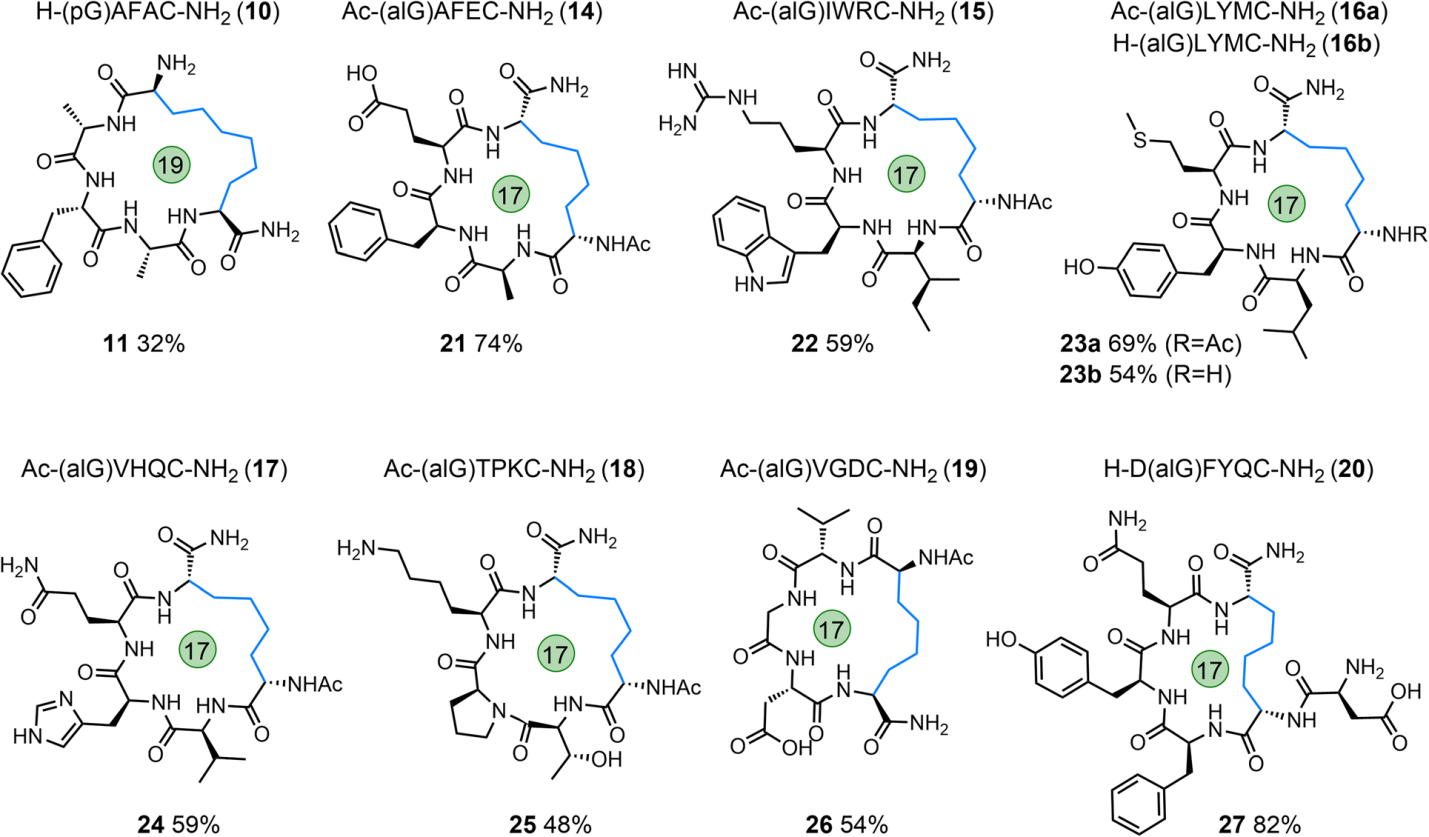
Linear starting peptides (10, 14–20) and the macrocyclic peptide products formed *via* desulfurative C–C bond formation (11, 21–27); macrocycle size indicated.

To enable the synthesis of peptide macrocycles with longer hydrocarbon linkages, the amino acid pentenyl glycine (pGly, pG) was incorporated into the N-terminus of a simple model peptide (H-(pG)AFAC-NH_2_; 10) and this peptide subjected to the optimised cyclisation conditions. The desired product (11) was successfully formed but in low yield compared to the alGly example (Table 1 and Fig. 4). Furthermore, in addition to the use of non-proteinogenic amino acids carrying alkenes, we also explored the on-resin installation of similar radical traps. The N-terminus of H-AFAC-NH_2_ was functionalised on-resin using a solution of pentafluorophenyl acrylate^46^ (12) to afford the linear acrylamide peptide, 13. When subjected to the optimised cyclisation conditions the starting peptide was fully consumed within 60 min; LC-MS analysis of the crude reaction material suggested the formation of an interesting phosphonium salt by-product (Fig. S74†) but no cyclised material was observed.

### Exploration of reaction scope and tolerance

**Fig. 5:**
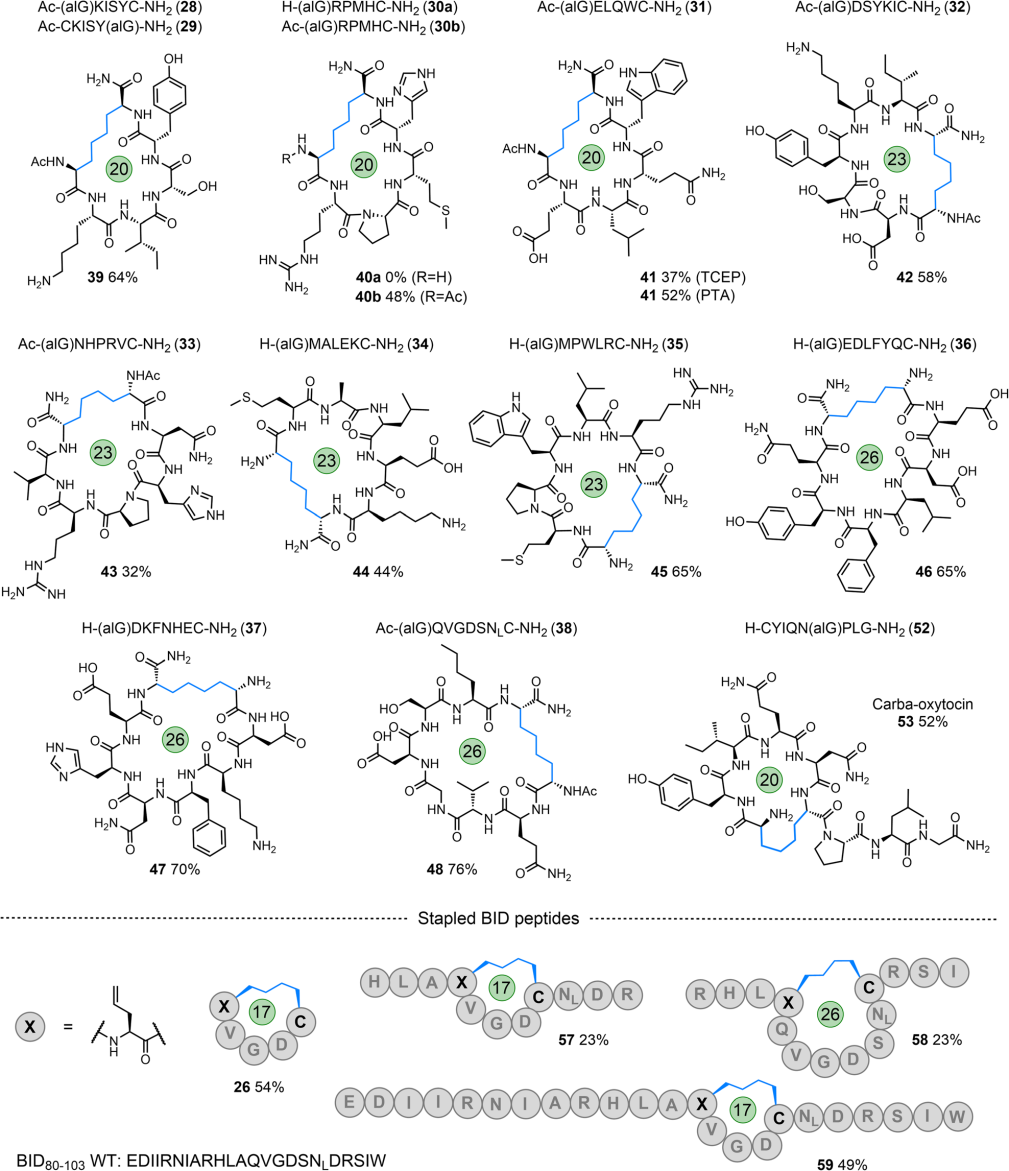
Starting peptides (28–38) and the macrocyclic peptide products formed *via* desulfurative C–C bond formation (39–48); carba-oxytocin (53); internally ‘stapled’ MCL-1 binding peptides (57–59), initial positions of alGly and Cys indicated within the sequence of these products; macrocycle size indicated.

To further explore the tolerance and scope of this cyclisation methodology we synthesised several pentapeptides carrying a range of proteinogenic amino acids (14–20; Fig. 4). When positioning the alGly residue at the N-terminus and the Cys at the C-terminus, effective N- to C-terminal macrocyclisation was realised for all sequences explored to yield 17-membered macrocycles (*wrt* the number of bonds within the macrocycle) in moderate to excellent yields (21–27, 48–74% isolated yield). The model peptides explored carried the majority of the 20 canonical amino acids, demonstrating the tolerance of this reaction to the diverse chemical functionality displayed across the proteome. Interestingly, switching the terminal residues eliminated or hindered conversion to the desired product for this size of macrocycle (Table S1†). To explore macrocyclisation of a peptide carrying the radical trap residue at an internal position, peptide 20 was synthesised with alGly as the penultimate N-terminal residue. For this model, cyclisation was successful in high yield (27, 82%). Moving to longer sequences we explored several 6, 7, & 8-residue peptides carrying a range of proteinogenic residues (28–38) to yield 20, 23, and 26-membered peptide macrocycles in moderate to excellent yield (39–48, 32–76%, Fig. 5). All macrocyclic products were characterised by analytical HPLC, MS and ^1^H NMR; CD spectroscopy was run for examples of each size of macrocycle (ESI†). Switching the N- and C-terminal residues for model 29 (H-CKISY(alG)-NH_2_) had no effect on yield for this size of macrocycle, unlike the smaller model peptides explored (Table S1†). In several cases of low or negligible yield (*e.g.*, 40a) acetylation of the N-terminus reinstated a moderate yield (40b, 48%). To confirm the effect that buffer composition has on the isolated yield, cyclisation of model 28 was carried out in PBS. Conversion to the desired product was again not as effective in this buffer compared to cyclisation in conjugation buffer. However, for this model, the ratio of product to desulfurised linear by-product was comparable to that of the conjugation buffer example. Additionally, PTA was revisited for the low-yielding model 31. While this additive did not give an improvement in yield for model 7a, we found that it improved the conversion to the product for the more complex model 31, increasing the yield from 37% to 52% (41). This result indicates that, while TCEP gives more favourable results for the majority of sequences, alternative phosphines can be employed.

A final 14-residue model peptide (49) was prepared to explore the preparation of larger (44-membered) macrocycles. Under the optimised cyclisation conditions the starting peptide was fully consumed to afford an inseparable mixture of the desired cyclised product and the desulfurised linear by-product. Increasing the loading of the PC to 5 mol% resulted in formation of the postulated phosphonium by-product observed for acrylamide model 13 (Fig. S144–S146†). This by-product (50) was isolated in 58% yield; ^31^P NMR analysis gave a single phosphorus resonance at 36.6 ppm. Our strategy is, therefore, highly effective for the formation of peptide macrocycles up to a 26-membered ring, and tolerates the full range of proteinogenic chemical functionality found across the proteome. Access to larger macrocycles may be possible, but will be dependent on successful separation of the desired product from the linear desulfurised by-product.

### Macrocyclisation of therapeutic peptides

To apply this technology to biomedically relevant peptides, the hormone oxytocin was synthesised with alGly replacing one of the Cys residues in the peptide to install a hydrocarbon ‘brace’ in place of the native disulfide (carba-oxytocin). This modification has been previously explored to enhance the proteolytic and hydrolytic stability of this peptide.^70^ Positioning alGly at the N-terminus and Cys at an internal position for this model (51, ESI†) failed to afford the desired product when applying the cyclisation conditions, instead producing desulfurised linear material only. Interestingly, Scanlan, Petracca and co-workers observed the same challenge when attempting to cyclise oxytocin *via* a thiol–ene reaction using an N-terminal alGly residue.^71^ We found that switching the positions of the Cys and alGly residues (sequence 52) re-instated the cyclisation, affording the desired macrocycle in moderate yield (53, 52% isolated yield, Fig. 5).

Finally, to explore internal peptide ‘stapling’ using this strategy, we selected a region of a BH3 protein – BID_80–102_, that binds MCL-1 to regulate apoptosis.^53,72^ This PPI is known to play a significant role in cancer development and progression.^53,72^ Three sequences (54–56) were synthesised with alGly and Cys positioned to afford either an internal *i*, *i* + 4 staple (57, 59; Fig. 5 and ESI†) or *i*, *i* + 7 staple (58; Fig. 5 and ESI†), fixing the length of either one or two full helical turns of the peptide, respectively. In addition, peptide macrocycle 26 is a head-to-tail macrocycle representing the *i*, *i* + 4 binding region of these longer sequences. The staple for all three peptides was successfully formed under the standard conditions, albeit in lower yield than the N- to C-terminal cyclisations previously explored (57–59). The alpha-helical structure of peptide 59 was assessed *via* CD spectroscopy. While macrocyclisation did increase the helicity by a few percent compared to the native BH3 sequence (22% compared to 19%), the starting peptide carrying the allyl glycine residue had a relatively high helical content (38%, Fig. S159†). This can be rationalised by considering the strain on the macrocycle imposed by the linker, and the fact that the native residues glutamine (Gln) and Ser were switched for alGly and Cys, respectively. These original residues have higher helical propensities relative to their replacements. Competitive inhibition studies (measured *via* fluorescence anisotropy) using MCL-1 and the fluorophore-labelled WT BH3 sequence demonstrated slightly lower inhibitory potency (26 ± 4 mM) for 59 compared to BID-wt (7.4 ± 0.9 mM) (Fig. S160†).

## Conclusions

Herein, we report a powerful method for the cyclisation of peptides *via* desulfurative C(sp^3^)–C(sp^3^) bond formation. Our approach is operationally simple, effective ‘on the bench’ under ambient conditions with irradiation of blue light, utilising readily available starting building blocks. The reaction is rapid, high yielding (for most cases studied) on unprotected peptides in aqueous solution and tolerant to all proteinogenic chemical functionality. The technique enables the preparation of a range of macrocycle sizes and extends to internal macrocyclisation (peptide stapling). This technology presents a more sustainable alternative to the widely employed RCM, offering an effective new method for peptide cyclisation.

## Data availability

The experimental procedures and compound/peptide characterisation data can be found in the ESI.†

## Author contributions

R. C. G. and N. J. M. conceived the project, F. R. S., R. C. G., D. M., H. J. K., P. Z., and H. E. L. W. carried out the experimental work. A. J. W. and N. J. M. supervised the work, N. J. M. wrote the manuscript. All authors contributed to writing the manuscript.

## Conflicts of interest

There are no conflicts to declare.

## Supplementary Material

SC-015-D3SC05865D-s001
