# Correction: Peptide macrocyclisation *via* intramolecular interception of visible-light-mediated desulfurisation

**DOI:** 10.1039/d5sc90021b

**Published:** 2025-01-31

**Authors:** Frances R. Smith, Declan Meehan, Rhys C. Griffiths, Harriet J. Knowles, Peiyu Zhang, Huw E. L. Williams, Andrew J. Wilson, Nicholas J. Mitchell

**Affiliations:** a School of Chemistry, University of Nottingham, University Park Nottingham NG7 2RD UK nicholas.mitchell@nottingham.ac.uk; b School of Chemistry, University of Leeds Woodhouse Lane Leeds LS2 9JT UK; c Biodiscovery Institute, University of Nottingham, University Park Nottingham NG7 2RD UK; d School of Chemistry, University of Birmingham Edgbaston Birmingham B15 2TT UK

## Abstract

Correction for ‘Peptide macrocyclisation *via* intramolecular interception of visible-light-mediated desulfurisation’ by Frances R. Smith *et al.*, *Chem. Sci.*, 2024, **15**, 9612–9619, https://doi.org/10.1039/D3SC05865D.

The authors regret that the incorrect analytical HPLC trace was assigned to product **53** (carba-oxytocin) in the ESI. The corrected experimental procedure, and the analytical HPLC trace and ESI-MS data for **53** (**Fig. S148**) have been provided here; the revised isolated yield for **53** is 35%.

The updated supplementary information file has been included with this correction article.

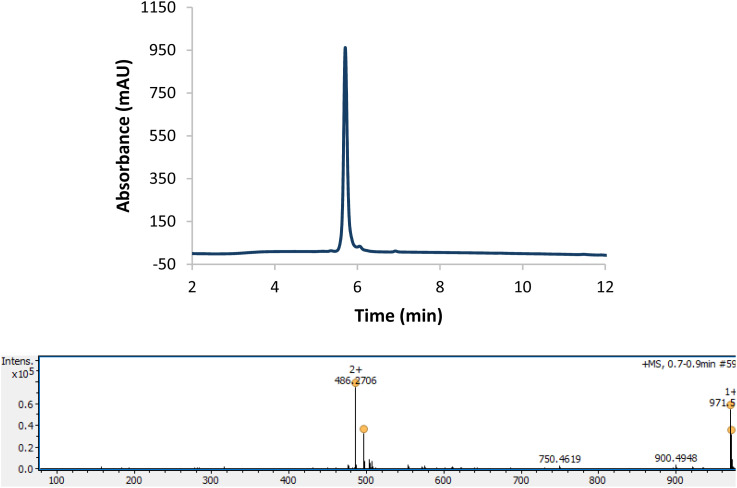



**Fig. S148.** Analytical HPLC trace and ESI MS of cyclised H-CYIQN(alG)PLG-NH_2_ (**53**); analytical gradient 10–50% B over 10 minutes, 210 nm. Calculated mass [M + H]^+^: 971.52, [M + 2H]^2+^: 486.26; observed mass [M + H]^+^: 971.53, [M + 2H]^2+^: 486.27.

Product **53** was synthesised following the optimised cyclisation protocol using H-CYIQN(alG)PLG-NH_2_ (**52**, 5 mg, 4.98 μmol). After analysis the remaining solution (4.89 μmol) was purified using semi-preparative HPLC (10–70% B over 30 minutes); the fractions containing the main products were lyophilised to yield the cyclised title compound (1.7 mg, 1.73 μmol, 35% yield) and the linear desulfurised by-product (2.5 mg, 2.54 μmol, 52% yield), both as fluffy white solids.

The Royal Society of Chemistry apologises for these errors and any consequent inconvenience to authors and readers.

## Supplementary Material

SC-016-D5SC90021B-s001

