# Supplementary material for: Peptide macrocyclisation via intramolecular interception of visible-light-mediated desulfurisation
Source: Chem Sci. 2024 May 14;15(25):9612–9. doi: 10.1039/d3sc05865d (PMC11206203; doi:10.1039/d3sc05865d)
Supplement: SC-015-D3SC05865D-s001 [file SC-015-D3SC05865D-s001.pdf]

Electronic Supplementary Material (ESI) for Chemical Science.
This journal is © The Royal Society of Chemistry 2024

Supporting Information

Peptide Macrocyclisation via Intramolecular Interception of Visible-Light-Mediated Desulfurisation

Frances R. Smith,[a] Declan Meehan,[a] Rhys C. Griffiths,[a] Harriet J. Knowles,[a] Peiyu Zhang,[b] Huw E. L. Williams,[c] Andrew J. Wilson,[b,d] and Nicholas J. Mitchell[a]*

[a] School of Chemistry, University of Nottingham, University Park, Nottingham NG7 2RD, UK

[b] School of Chemistry, University of Leeds, Woodhouse Lane, Leeds LS2 9JT, UK

[c] Biodiscovery Institute, University of Nottingham, University Park, Nottingham NG7 2RD, UK

[d] School of Chemistry, University of Birmingham, Edgbaston, Birmingham B15 2TT, UK

*Email: nicholas.mitchell@nottingham.ac.uk

Contents

General Methods 3
- Materials 4
- Solid Phase Peptide Synthesis (SPPS) 4
- Circular dichroism 6
- Fluorescence anisotropy 7
- Photochemistry apparatus 8
- Peptide macrocyclisation via intramolecular trapping of desulfurisation 9

Compound Synthesis 10
Peptide Models 14
Peptide Macrocyclisation 47
References 157

General Methods

NMR samples were analysed on either a Bruker AVIII 400 NMR system (^{1}H-NMR frequency 400 MHz; ^{13}C-NMR frequency 100 MHz), a Bruker 500 system (^{1}H-NMR frequency 500 MHz; ^{13}C-NMR frequency 125 MHz), or a Bruker 600 Advance III (^{1}H-NMR frequency 600 MHz). Chemical shifts are reported in parts per million (ppm) and are referenced to solvent residual signals: $CDCl_3$ (δ 7.26 [^{1}H]), DMSO (δ 2.50 [^{1}H]), MeOD (δ 3.31 [^{1}H]). ^{1}H NMR data is reported as chemical shift (δ), multiplicity (s = singlet, d = doublet, t = triplet, q = quartet or combinations of these splitting patterns; m = unassigned multiplet), relative integral, and coupling constant (J Hz). ^{13}C NMR data is reported as chemical shift (δ) and classification of the carbon (e.g., CH_3/CH_2/CH/C).

NMR spectra for peptide macrocycle **8a** were recorded at 500 MHZ using a 5 mm DCI Cryoprobe on a Bruker AV3 HD spectrometer. NMR samples were acquired in d6-DMSO and locked and referenced as per manufacturers standard. Two-dimensional TOCSY and NOESY data were acquired with 16 transients, 512 points in t1 and employed States-TPPI for quadrature detection in the indirect dimension . TOCSY data were acquired with a 60 ms mixing period using a DIPSI2 spin lock. NOESY data were acquired with 300 ms mixing and employed a zero-quantum filter to reduce artefacts. Additional ^{13}C-^{1}H heteronuclear experiments (HSQC with multiplicity editing and HMBC) were acquired to aid to confirm assignments. All data were processed using TOPSPIN version 3 or 4. A shifted sine squared function was employed and data were zero-filled. Data were assigned using CCPNMR v3 software.

High-resolution mass spectra were recorded on a Bruker MicroTOF Focus II MS (ESI) operating in positive or negative ionisation mode. Analytical HPLC was performed on a Thermo Ultimate 3000 mHPLC system equipped with PDA eλ detector (λ = 210 – 400 nm). Peptides were analyzed using a Waters Sunfire 5 µm, 2.1 x 150 mm column (C-18) at a flow rate of 0.6 mL•min^{-1}. The mobile phase composed of 0.1% trifluoroacetic acid in H_2O (Solvent A) and 0.1% trifluoroacetic acid in acetonitrile (Solvent B). The analysis of the chromatograms was conducted using Chromeleon 7 software. Reaction conversions (%) were calculated from the integrals of all peptide peaks in the analytical HPLC spectra of the crude reaction mixtures.

Preparative reverse-phase HPLC was performed using a Waters 1525 binary pump HPLC equipped with a dual wavelength UV detector set to 210 nm and 280 nm. Peptides were purified on a Waters Sunfire 5 μm, 19 x 150 mm (C-18) preparative column operating at a flow rate of 6 mL•min^{-1} using a mobile phase of 0.1% trifluoroacetic acid in water (Solvent A) and 0.1% trifluoroacetic acid in acetonitrile (Solvent B) using the gradient specified. Semi-preparative reverse-phase HPLC was performed using the same HPLC and solvent system. The column used was a Waters Sunfire 5 μm, 10 x 250 mm (C-18) preparative column, operating at a flow rate of 5 mL•min^{-1} using the gradient specified.

Circular dichroism was carried out using an Applied Photophysics Chirascan Plus.

Materials

Commercial materials were used as received unless otherwise noted. Amino acids, coupling reagents and resins were obtained from Novabiochem, Fluorochem or GL Biochem. Reagents that were not commercially available were synthesised as outlined. Solvents were obtained as reagent grade from Merck or Fisher.

Solid Phase Peptide Synthesis (SPPS)

Manual Fmoc-SPPS

Preloading Rink Amide resin: Rink amide resin was initially washed with DCM (5 × 3 mL) followed by removal of the Fmoc group by treatment with 20% piperidine/DMF (2 × 5 min). The resin was washed with DMF (5 × 3 mL), DCM (5 × 3 mL) and DMF (5 × 3 mL). Oxyma Pure (4 eq.) and DIC (4 eq.) were added to a solution of Fmoc-AA-OH (4 eq.) in DMF. After 5 min of pre-activation, the mixture was added to the resin. After 2 h the resin was washed with DMF (5 × 3 mL), DCM (5 × 3 mL) and DMF (5 × 3 mL), capped with acetic anhydride/pyridine (1:9 v/v) (2 × 3 min) and washed with DMF (5 × 3 mL), DCM (5 × 3 mL) and DMF (5 × 3 mL).

Preloading 2-chlorotrityl chloride resin: 2-Chlorotrityl chloride resin was swollen in DCM for 30 min then washed with DCM (2 × 3 mL). A solution of Fmoc-AA-OH (0.5 equiv. relative to resin functionalization) and *i*Pr_2NEt (2.0 eq. relative to resin functionalization) in DCM (final concentration 0.1 M of amino acid) was added and the

resin shaken at rt for 16 h. The resin was washed with DMF (5 × 3 mL) and DCM (5 × 3 mL). The resin was treated with a solution of DCM/CH_3OH/$i$$Pr_2$NEt (17:2:1 v/v/v, 3 mL) for 1 h and washed with DMF (5 × 3 mL), DCM (5 × 3 mL), and DMF (5 × 3 mL). The resin was subsequently submitted to iterative peptide assembly (Fmoc-SPPS).

Quantification of amino acid loading: The resin was treated with 20% piperidine/DMF (2 x 3 mL, 3 min) and 20 μL of the combined deprotection solution was diluted to 10 mL using 20% piperidine/DMF in a volumetric flask. The UV absorbance of the resulting piperidine-fulvene adduct was measured (λ = 301 nm, ε = 7800 M^{-1} cm^{-1}) to determine the loading of the resin.

General amino acid coupling: A solution of appropriately protected Fmoc-amino acid (4 eq.), DIC (4 eq.) and Oxyma Pure (4 eq.) in DMF (final concentration 0.1 M) was added to the resin. After 1 h, the resin was washed with DMF (5 × 3 mL), DCM (5 × 3 mL) and DMF (5 × 3 mL).

Capping: Acetic anhydride/pyridine (1:9 v/v) was added to the resin (3 mL). After 3 min the resin was washed with DMF (5 × 3 mL), DCM (5 × 3 mL) and DMF (5 × 3 mL).

Deprotection: The resin was treated with 20% piperidine/DMF (2 x 3 mL, 3 min) and washed with DMF (5 × 3 mL), DCM (5 × 3 mL) and DMF (5 × 3 mL).

Cleavage: A mixture of TFA, thioanisole, tri*iso*propylsilane (TIS) and water (90:4:4:2 v/v/v/v) was added to the resin. After 3 h, the resin was washed with TFA (3 × 2 mL).

Work-up: The combined cleavage solutions were concentrated under a stream of nitrogen to < 5 mL. 40 mL of diethyl ether was added to precipitate the peptide and the suspension centrifuged. The pellet was then dissolved in water containing 0.1% TFA, filtered and purified by preparative HPLC and analyzed by LC-MS and ESI mass spectrometry.

Automated solid-phase peptide synthesis

Automated Fmoc-SPPS was carried out on either a Biotage Initiator$^+$ Alstra or CEM Liberty Blue microwave peptide synthesiser. General synthetic procedures for Fmoc-deprotection and capping were carried out in accordance with the manufacturer's specifications. Biotage Initiator$^+$ Alstra: standardized amino acid couplings were

performed for 15 min at 50 ^{o}C under microwave irradiation in the presence of amino acid (0.5 M in DMF, 4 eq.), Oxyma Pure (0.5 M in DMF, 4 eq.) and di*iso*propylcarbodiimide (0.5 M in DMF, 4 eq.). Peptide cleavage and work-up were carried out as described above for manual SPPS. CEM Liberty Blue: standardized amino acid couplings were performed for 2.5 min at 90 ºC under microwave irradiation in the presence of amino acid (0.2 M in DMF, 4 eq.), Oxyma Pure (1 M in DMF, 4 eq.) and di*iso*propylcarbodiimide (1 M in DMF, 4 eq.). Peptide cleavage and work-up were carried out as described above for manual SPPS.

Circular dichroism

All samples were dissolved in phosphate buffer (20 mM phosphate, 100 mM NaCl, pH 7.4). After centrifugation (15000 rpm × 3 min), the accurate concentrations of supernatants were determined using a NanoDrop 2000 spectrometer (ThermoFisher, Waltham, MA, USA) and all samples were diluted to a final concentration of 50 μM. The CD experiments were performed on a Chirascan circular dichroism spectrometer (Applied Photophysics, UK) at 20 °C, using a 1 mm quartz cuvette. The parameters were set as follows: wavelength 190-260 nm, scan speed 5 nm min-1, step resolution 1nm. The spectra were averaged over triplicates after subtracting buffer baselines. The percentages of helicity were calculated based on the mean residue ellipticity at 222 nm using the following equation:

$$f = \frac{[\theta]_{222} - [\theta]_0}{[\theta]_{max} - [\theta]_0} \quad (1)$$

Where $[\theta]_{222}$ is the observed residue ellipticity at 222 nm and calculated using: $[\theta]_{222}$=1/n·$[\theta]_{obs}$/(10·l·C), where n = number of residues; $[\theta]_{obs}$ = measured value in mdeg; C = sample concentration (mol/L); l = path length of the cuvette in cm. $[\theta]_0$ is the mean residue ellipticity of a random coiled peptide and calculated by (2220-53T). $[\theta]_{max}$ is the theoretically maximum mean residue ellipticity of an n-mer helical peptide and equals to $[\theta]_{\infty}$ · (n-3)/n, where $[\theta]_{\infty}$ equals to (-44000 + 250T) (T is the temperature of peptide solutions in Celsius; 20 °C was used in this study).

Fluorescence anisotropy

MCL-1 (residues 172-327) was expressed and purified as described previously.[1-2] FAM-Ahx-BID was synthesised by solid-phase peptide synthesis and purified by reverse phase HPLC as described previously.[3]

All samples were prepared in 20mM Tris, 150 mM NaCl, pH 7.6 solutions. All assays were performed using 384-well plates (Greiner Bio-one, UK) and tested in triplicate using an EnVisionTM 2103 MultiLabel plate reader (Perkin Elmer; Waltham, MA, USA). The parameters were set as follows: excitation wavelength = 480 nm (30 nm bandwidth) and emission wavelength = 535 nm (30 nm bandwidth). Measured data were processed and analysed as previously described.[1-2] Specifically, the perpendicular intensity (*P*) and parallel intensity (*S*) were subtracted by the control values and used for calculations of intensity and anisotropy using the following equations:

$$I = (2PG) + S \tag{2}$$

$$r = (S - PG)/I \tag{3}$$

$$y = r_{max} + \frac{r_{min} - r_{max}}{1 + (x/x_0)^p} \tag{4}$$

Where I = total intensity; P = perpendicular intensity; S = parallel intensity; G = instrument factor which was set to 1.1 for all assays; r = anisotropy. For competition FA assays, the average anisotropy and the average standard deviation of the values derived from equation (3) were calculated and fit to a sigmoidal logistic model (equation (4)) using Origin 2021.

Photochemistry apparatus

Set up 1 A blue LED light strip (blue LED, 6W, 6500 K) wrapped around a pyrex dish, placed on top of a stirrer plate. To ensure consistency, places for up to 4 vials were marked on the plate. The temperature was monitored and observed to reach no higher than 30 °C.

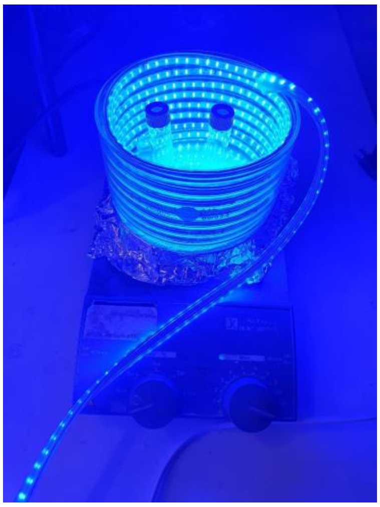

Set up 2 To compare set up 1 with specialized equipment, all reactions were also carried out in a temperature controlled PhotoRedOx Box (HCK1006-01-016, HepatoChem) operated with a 450 nm, 34 mW/cm^2 bulb (450PF, HCK1012-01-002, Hapatochem) at room temp.

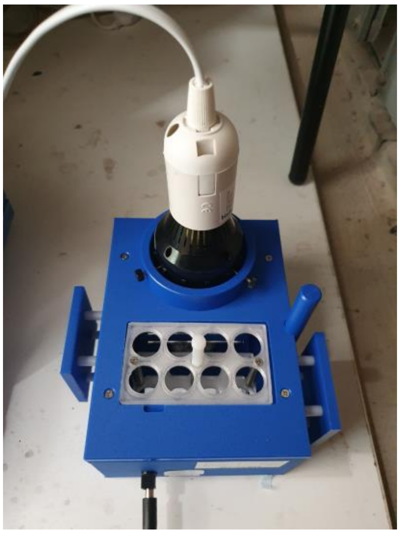

Peptide macrocyclisation via intramolecular trapping of desulfurisation

Optimised cyclisation protocol

To peptide dissolved in 10% acetonitrile (MeCN) in 6 M Gdn•HCl, 0.1 M Na_2HPO_4, pH 7 to a concentration of 1 mM was added a solution of TCEP (0.5 M stock solution in 6 M Gdn•HCl, 0.1 M Na_2HPO_4, pH adjusted to 7 - 50 eq.) and $(Ir[dF(CF_3)ppy]_2(dtbpy))PF_6$ (1 mM stock solution in MeCN, 0.01 eq.). The pH of the reaction mixture was checked to be 7.5 - 8.0 and the reaction mixture diluted to the final peptide concentration of 0.5 mM. The reaction vessel was then placed into either blue LEDs (set up 1) or into a PhotoRedOx Box (HepatoChem; set up 2); once the starting material was shown to be fully consumed by analytical HPLC (15 min PhotoRedOx Box, 45 min LED light strips), the reaction mixture was purified by semi-preparative HPLC.

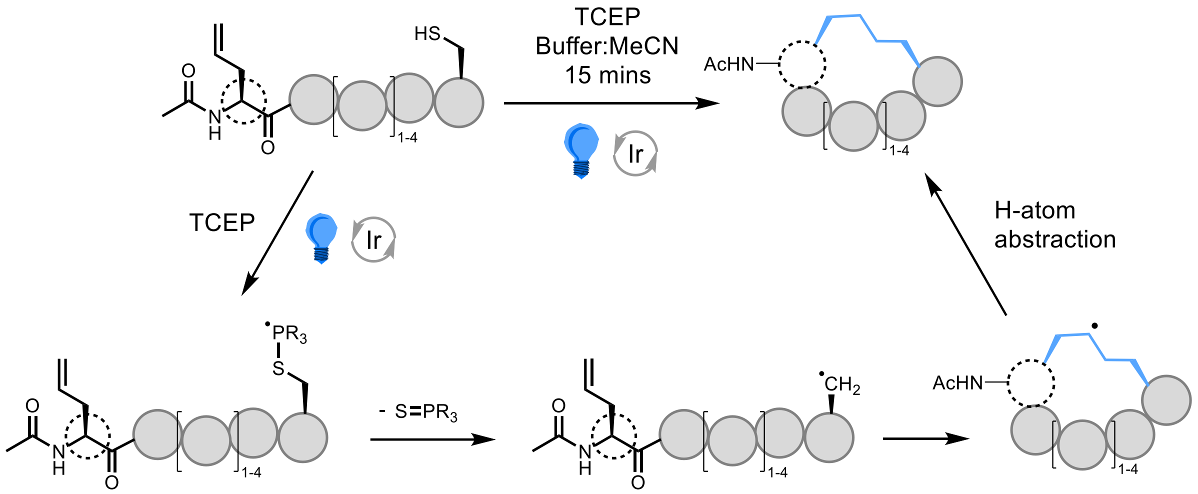

Figure S1. Peptide cyclisation via intramolecular interception of visible-light-mediated desulfurisation.

Compound Synthesis

N-(*tert*-butoxycarbonyl)-*O*-(2-methylallyl)-*L*-serine (**1**)

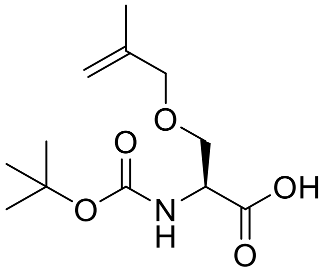

To Boc-Ser-OH (0.25 g, 1.22 mmol) in 5 mL DMF was added NaH (60% suspension in mineral oil; 0.15 g, 3.75 mmol) under nitrogen at 0 deg C and the solution was allowed to stir for 15 min before 1-bromo-3-methylbut-3-ene (0.2 g, 1.34 mmol) in 2 mL DMF was added dropwise. The reaction was allowed to stir under nitrogen for 2 hours at 0 deg C before pouring into water and extracting with ethyl acetate. The separated organic phase was washed with 1 M HCl, saturated $NaHCO_3$, and brine, dried over $MgSO_4$, and the solvent removed under reduced pressure. The crude material was purified via flash chromatography (SiO_2) using a 1:1 diethyl ether/pentane solvent systems with addition of acetic acid after the first two column volumes were eluted to yield the desired product (**1**, 0.31 g, 1.19 mmol, 98% yield). HRMS Calc. = 258.1420 $[M-H]^-$; Obs. = 258.1369; ^{1}H NMR (400 MHz, $CDCl_3$) δ 5.03 – 4.84 (m, 2H), 4.48 (dd, *J* = 8.4, 3.9 Hz, 1H), 3.93 (d, *J* = 5.4 Hz, 2H), 3.91 – 3.83 (m, 1H), 3.66 (dd, *J* = 9.5, 3.6 Hz, 1H), 1.72 (s, 3H), 1.47 (s, 9H). ^{13}C NMR (101 MHz, $CDCl_3$) δ 175.6, 155.7, 141.3, 113.0, 80.3, 75.4, 69.4, 53.8, 28.3, 19.3.

O-Allyl-*N*-(*tert*-butoxycarbonyl)-*L*-serine (**2**)

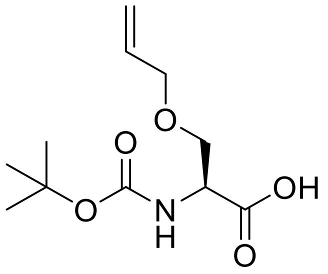

Compound **2** was prepared as previously described.[4] Boc-Ser-OH (500 mg, 2.44 mmol) was dissolved in anhydrous DMF (3 mL) and was added slowly to a suspension of NaH (60% suspension in mineral oil; 146 mg, 3.65 mmol) in anhydrous DMF (2 mL) at 0 ^{o}C. After the addition was completed, allyl bromide (262 μL, 3.05 mmol) was added and the reaction mixture was stirred at room temperature for 18 h. The reaction was then quenched using water (1 mL) and the solvent removed under reduced pressure. The residue was dissolved in water and acidified to pH 4 using 1 M HCl, and extracted into ethyl acetate (3 x 30 mL). The organic extracts were combined, dried using $MgSO_4$, and concentrated under reduced pressure to give the title compound as a pale yellow oil (**2**, 246 mg, 1.00 mmol, 41%). HRMS Calc. = 244.1190 $[M-H]^-$; Obs. = 244.1202 $[M-H]^-$. ^{1}H NMR (400 MHz, DMSO) δ 6.84 (d, *J* = 8.2 Hz, 1H), 5.85 (ddt, *J* = 17.3, 10.5, 5.3 Hz, 1H), 5.37 – 4.95 (m, 2H), 4.12 (dt, *J* = 8.3, 5.4 Hz, 1H), 3.93 (dq, *J* = 5.3, 1.7 Hz, 2H), 3.65 – 3.54 (m, 2H), 1.38 (s, 9H). ^{13}C NMR (126 MHz, DMSO) δ 172.0, 135.0, 116.6, 78.1, 71.0, 69.2, 53.9, 28.2.

Commercially available amino acid building blocks

Fmoc-allyl-Gly-OH

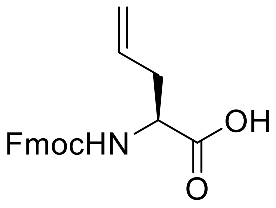

CAS: **146549-21-5**

(*S*)-*N*-Fmoc-2-(4'-pentenyl)glycine

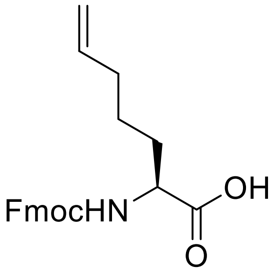

CAS: **856412-22-1**

Pentafluorophenyl acrylate (**12**)

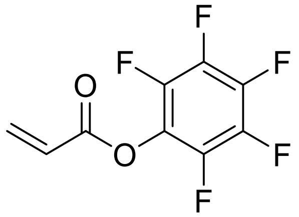

Pentafluorophenol (10.8 mmol, 1.0 eq.) and trimethylamine (1.82 mL, 13 mmol, 1.2 eq.) were dissolved in diethyl ether (15 mL) and cooled to 0 ℃ in an ice bath. Acryloyl chloride (969 μL, 12 mmol, 1.1 eq.) was dissolved in diethyl ether (500 μL) and added dropwise to the solution over 5 min. The solution was then brought to rt and the reaction preceded for 3 h. The product was then concentrated under reduced pressure and purified via flash chromatography (SiO_2) run in petroleum ether. The product was isolate as a colourless liquid (2.23 g, 9.36 mmol, 87%). ^{1}H NMR (400 MHz, $CDCl_3$) δ_H ppm 6.74 (d, *J* = 17.3, 1.1 Hz, 1H), 6.39 (dd, *J* = 17.3, 10.5 Hz, 1H), 6.20 (d, *J* = 10.5, 1.1 Hz, 1H). ^{19}F NMR (376 MHz, $CDCl_3$) δ_F ppm -162.6 (d, 2F) -158.2 (t, 1F), -152.8 (d, 2F).

Peptide Models

H-S(2-methylallyl)AFAC-NH_2 (**3**)

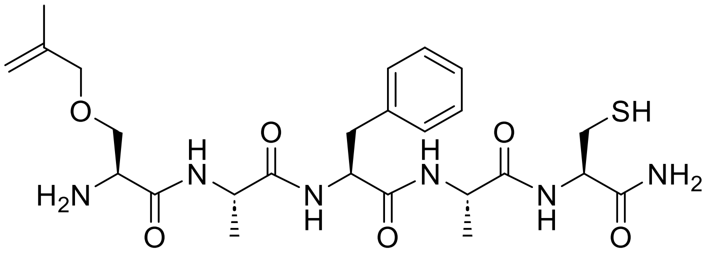

Model peptide **3** was synthesised using the general automated synthesiser procedure with microwave assistance on Rink Amide resin (0.05 mmol). The crude peptide was purified by preparative RP-HPLC (5-60% B over 30 minutes) and lyophilized to produce the desired peptide (12 mg, 22 µmol, 44% yield).

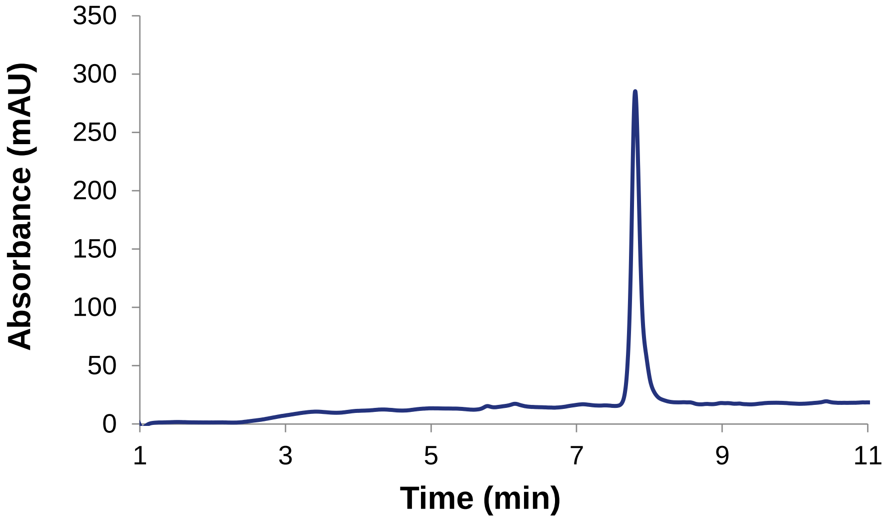

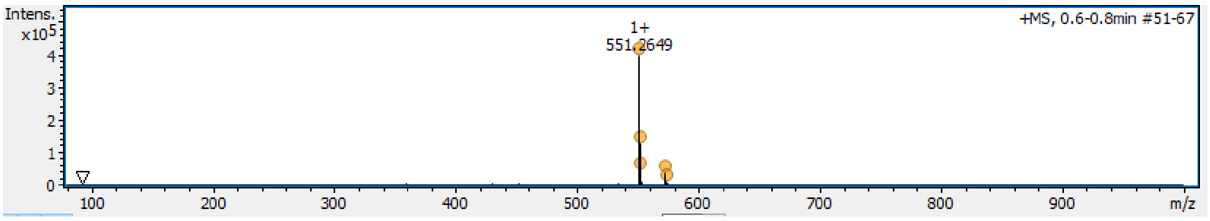

Figure S2. Analytical HPLC trace and ESI MS for product **3**; analytical gradient 5-60% B over 10 min, 210 nm. Calculated mass $[M+H]^+$: 551.27; observed mass $[M+H]^+$: 551.26.

H-S(*O*Allyl)AFAC-NH_2 (**4**)

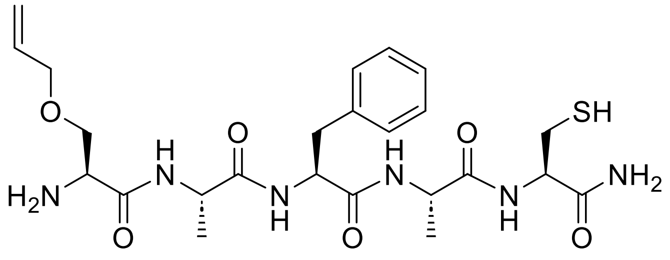

Model peptide **4** was synthesised using the general automated synthesiser procedure with microwave assistance on Rink Amide resin (0.10 mmol). The crude peptide was purified by preparative RP-HPLC (2-95% B over 30 minutes) and lyophilized to produce the desired peptide (26 mg, 0.05 mmol, 49% yield).

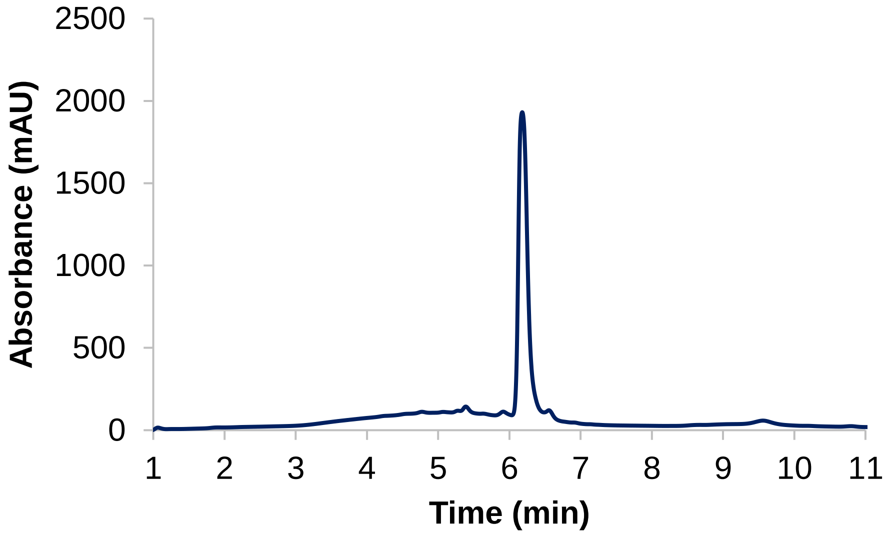

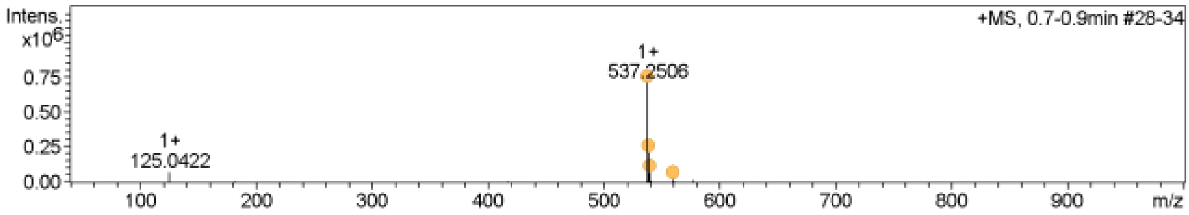

Figure S3. Analytical HPLC trace and ESI MS for product **4**; analytical gradient 5-60% B over 10 min, 210 nm. Calculated mass $[M+H]^+$: 537.25; observed mass $[M+H]^+$: 573.25.

H-(alG)AFAC-NH2 (**7a**)

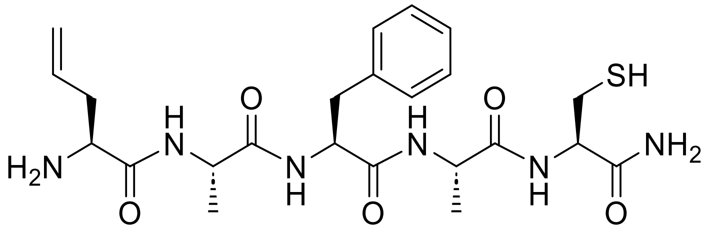

Model peptide **7a** was synthesised using the general automated synthesiser procedure with microwave assistance on Rink Amide resin (0.05 mmol). The crude peptide was purified by preparative RP-HPLC (5-60% B over 30 minutes) and lyophilized to produce the desired peptide (14 mg, 27.6 µmol, 55% yield).

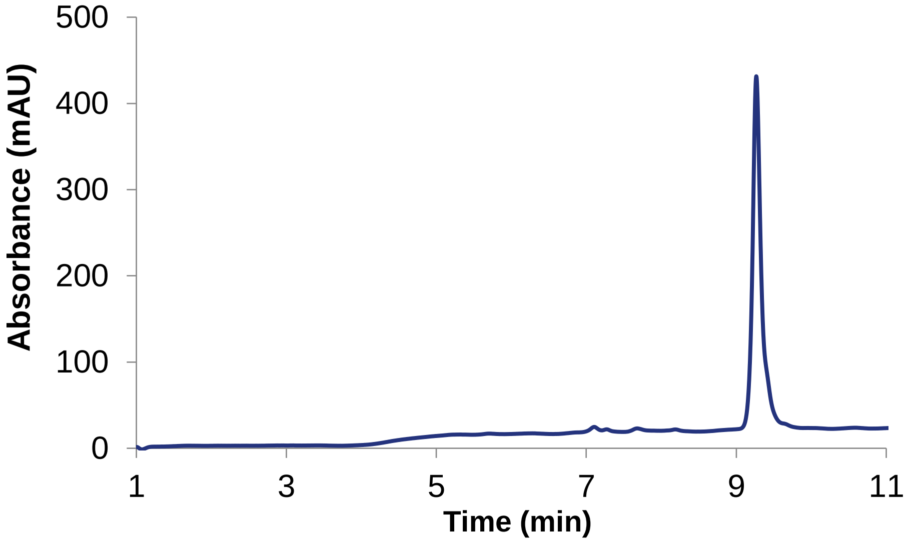

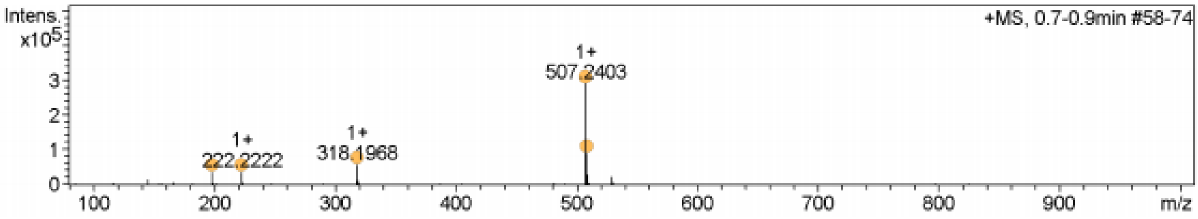

Figure S4. Analytical HPLC trace and ESI MS for product **7a**; analytical gradient 10-30% B over 10 min, 210 nm. Calculated mass $[M+H]^+$: 507.24; observed mass $[M+H]^+$: 507.24.

H-(alG)AFAC-OH (**7b**)

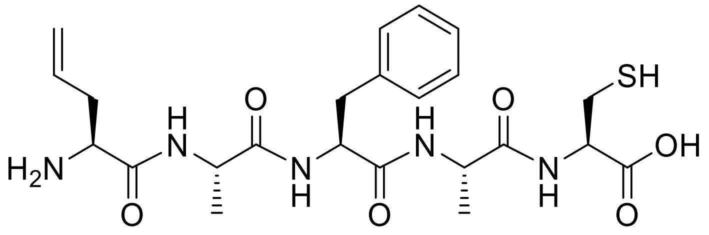

Model peptide **7b** was synthesised using the general automated synthesiser procedure with microwave assistance on 2-CTC resin (0.25 mmol). The crude peptide was purified by preparative RP-HPLC (5-60% B over 30 minutes) and lyophilized to produce the desired peptide (36.1 mg, 71.2 µmol, 67% yield).

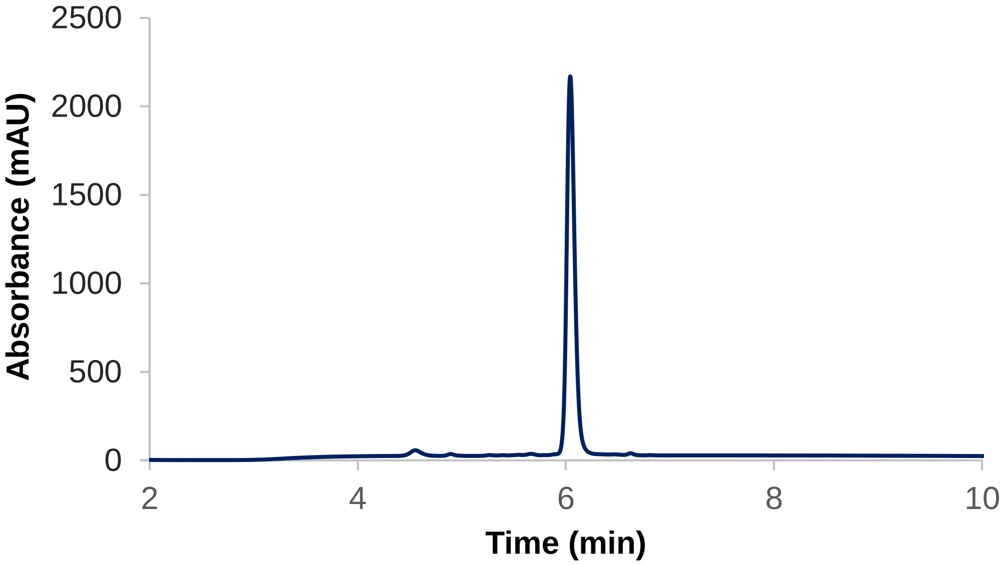

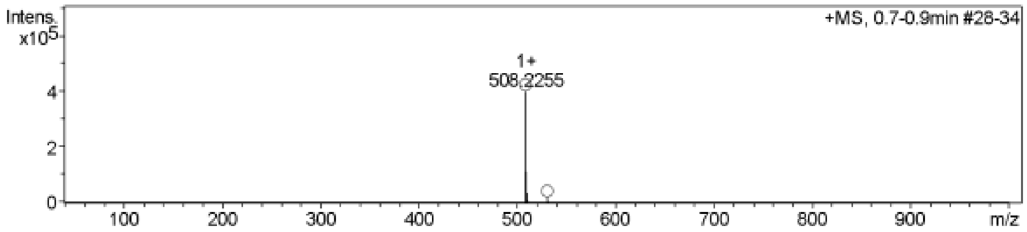

Figure S5 - Analytical HPLC trace and ESI MS for product **7b**; analytical gradient 5-60% B over 10 min, 210 nm. Calculated mass $[M+H]^+$: 508.22; observed mass $[M+H]^+$: 508.23

H-(alG)AFAA-NH_2 (**9**)

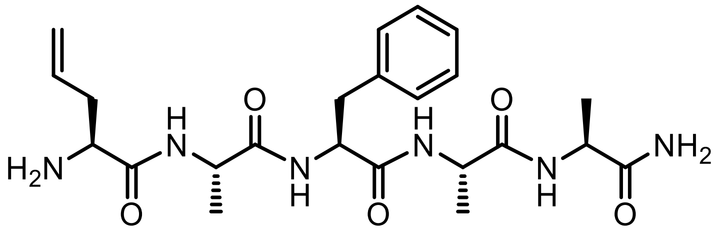

Model peptide **9** was synthesised using the general automated synthesiser procedure with microwave assistance on Rink Amide resin (0.05 mmol). The crude peptide was purified by preparative RP-HPLC (5-60% B over 30 minutes) and lyophilized to produce the desired peptide (12 mg, 25.3 µmol, 51% yield).

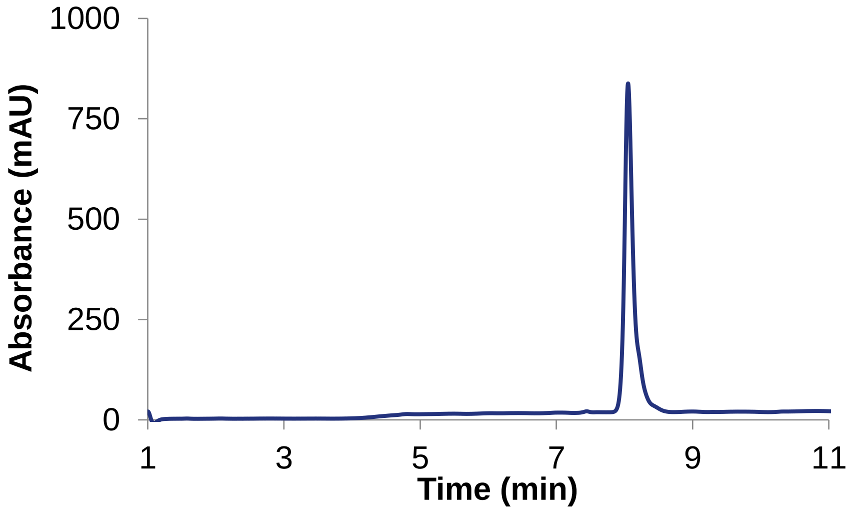

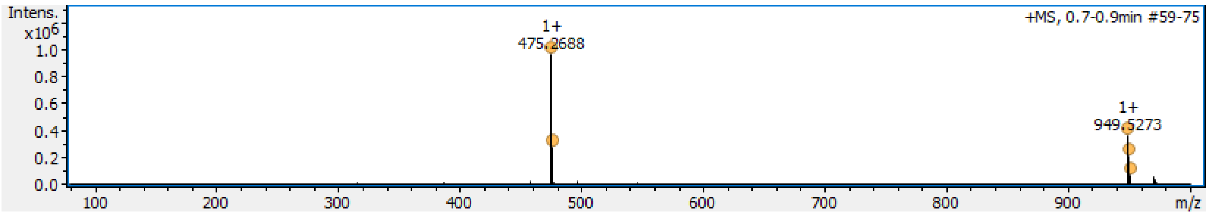

Figure S6. Analytical HPLC trace and ESI MS for product **9**; analytical gradient 5-25% B over 10 min, 210 nm. Calculated mass $[M+H]^+$: 475.27; observed mass $[M+H]^+$: 475.27.

H-(pG)AFAC-NH2 (**10**)

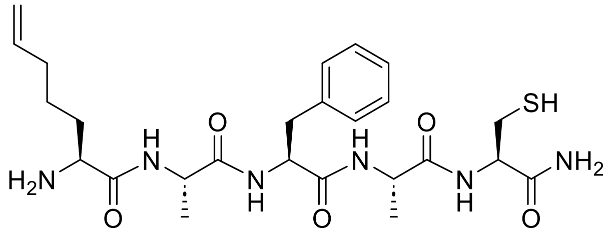

Model peptide **10** was synthesised using the general automated synthesiser procedure with microwave assistance on Rink Amide resin (0.25 mmol). The crude peptide was purified by preparative RP-HPLC (15-80% B over 30 minutes) and lyophilized to produce the desired peptide (69.6 mg, 0.13 mmol, 52% yield).

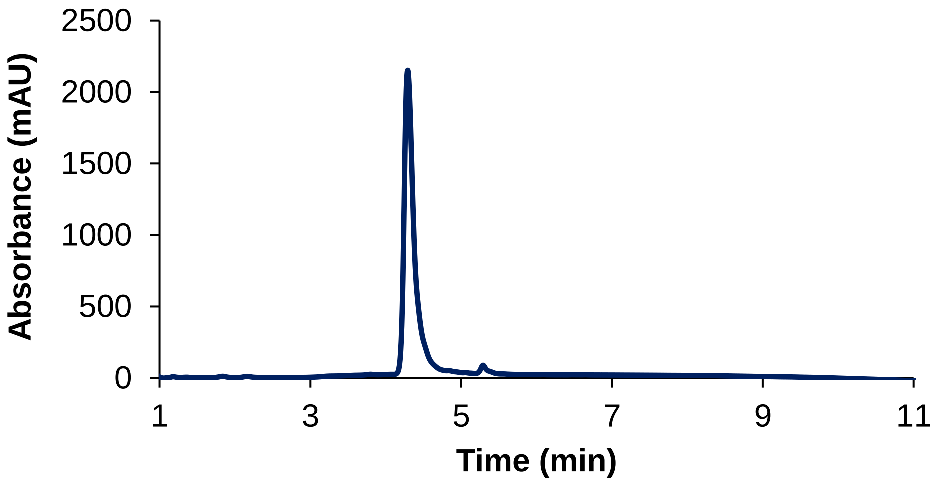

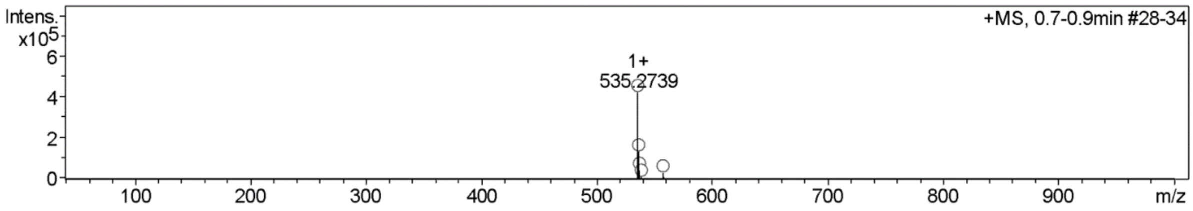

Figure S7 - Analytical HPLC trace and ESI MS for product **10**; analytical gradient 15-80% B over 10 min, 210 nm. Calculated mass $[M+H]^+$: 535.27; observed mass $[M+H]^+$: 535.27

Acr-AFEC-NH_2 (**13**)

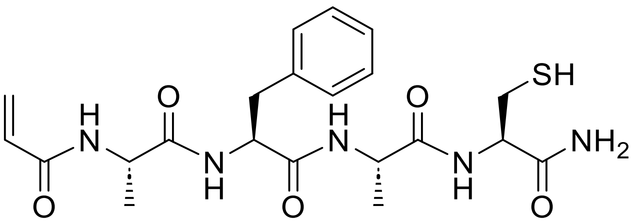

Model peptide **13** was synthesised using the general automated synthesiser procedure with microwave assistance on Rink Amide resin (0.25 mmol). On resin acryloyl capping was carried out with a solution of pentafluorophenyl acrylate (179 mg, 0.75 mmol, 3.0 eq.), and DIPEA (216.6 μL, 1.5 mmol, 6.0 eq.) in DMF (4 mL). The solution was shaken at 200 rpm at rt for 4 h. The resin was drained and rinsed with DMF (6 x 5 mL). Acryloyl capping procedure was repeated twice. The crude peptide was purified by preparative RP-HPLC (10-80% B over 30 minutes) and lyophilized to produce the desired peptide (4 mg, 8.63 μmol, 4% yield).

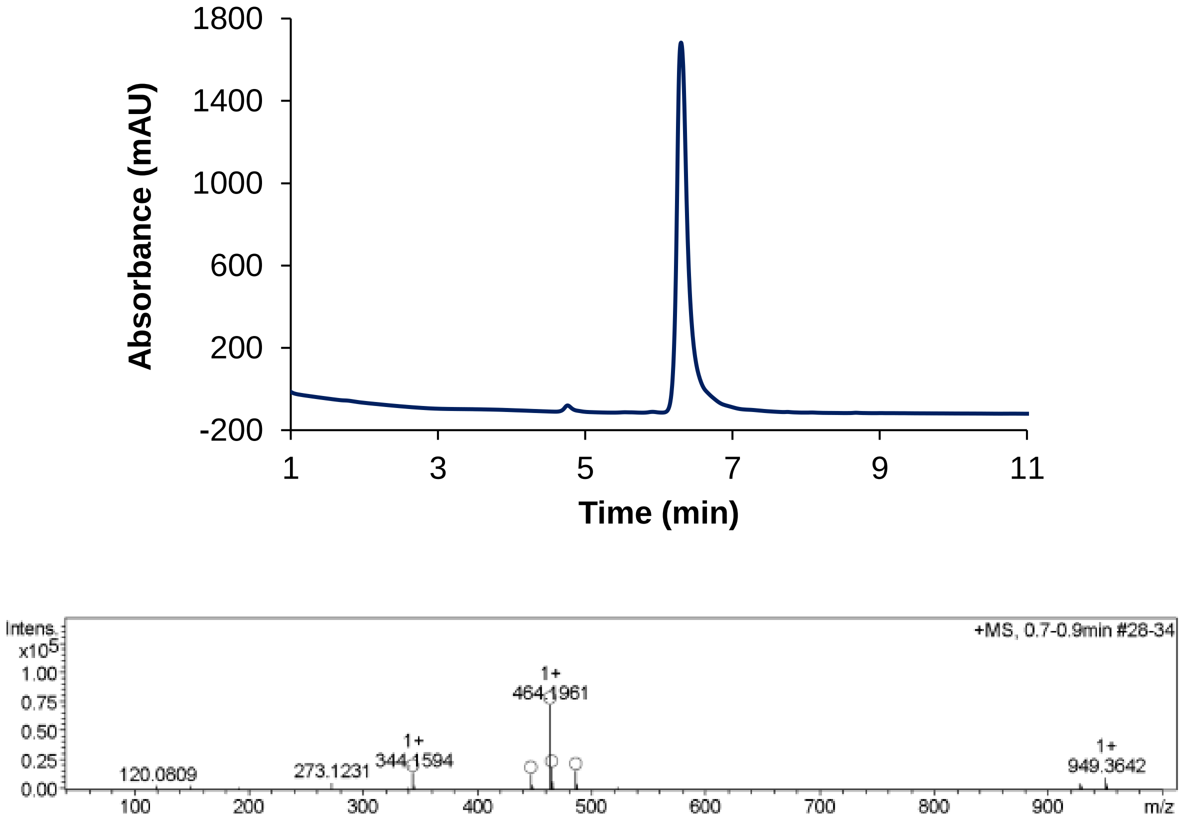

Figure S8 - Analytical HPLC trace and ESI MS for product **13**; analytical gradient 10-50% B over 10 min, 210 nm. Calculated mass $[M+H]^+$: 464.19; observed mass $[M+H]^+$: 464.19.

Ac-(alG)AFEC-NH2 (**14**)

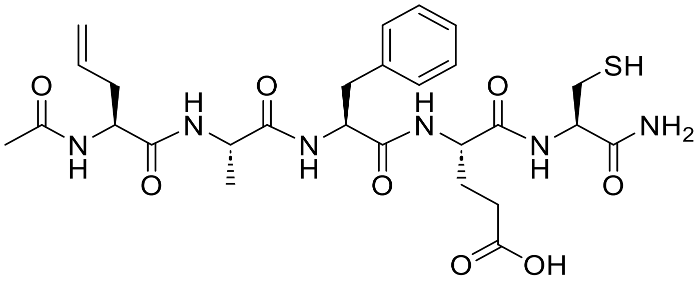

Model peptide **14** was synthesised using the general automated synthesiser procedure with microwave assistance on Rink Amide resin (0.25 mmol). The crude peptide was purified by preparative RP-HPLC (15-80% B over 30 minutes) and lyophilized to produce the desired peptide (84 mg, 0.14 mmol, 55% yield).

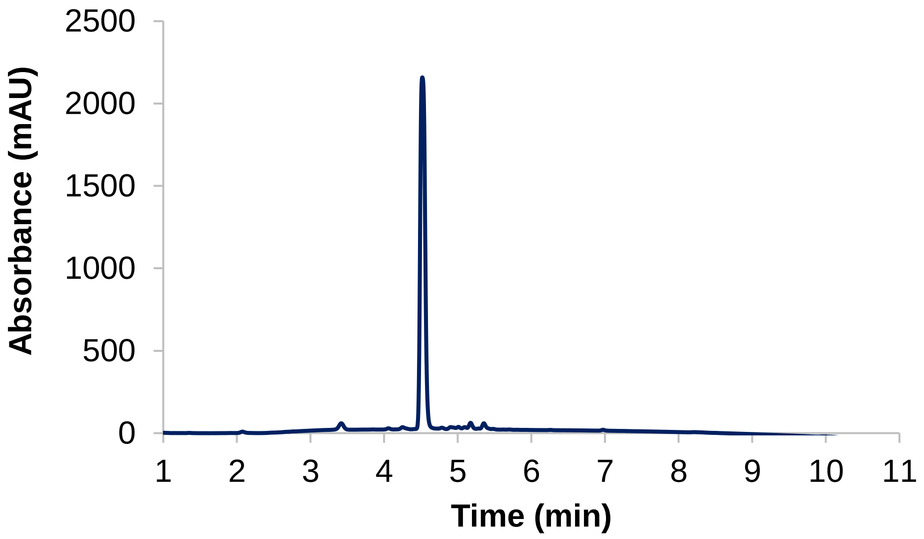

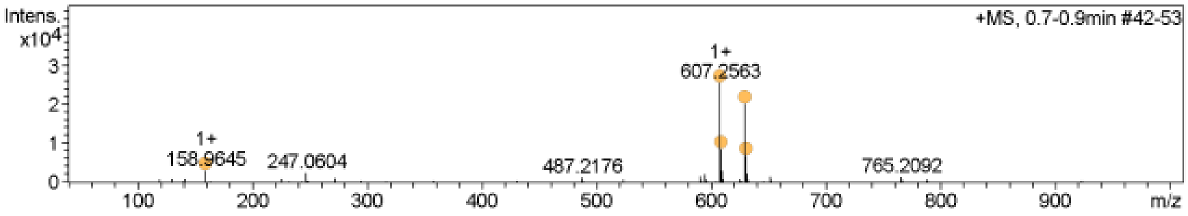

Figure S9 - Analytical HPLC trace and ESI MS for product **14**; analytical gradient 15-80% B over 10 min, 210 nm. Calculated mass $[M+H]^+$: 607.25; observed mass $[M+H]^+$: 607.25

Ac-(alG)IWRC-NH2 (**15**)

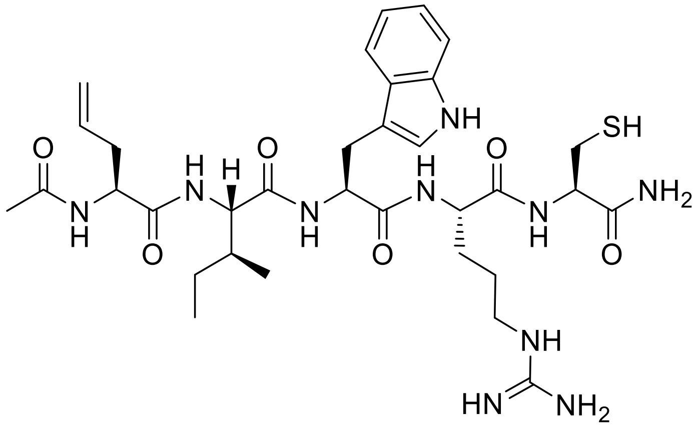

Model peptide **15** was synthesised using the general automated synthesiser procedure with microwave assistance on Rink Amide resin (0.25 mmol). The crude peptide was purified by preparative RP-HPLC (15-80% B over 30 minutes) and lyophilized to produce the desired peptide (72 mg, 0.10 mmol, 40% yield).

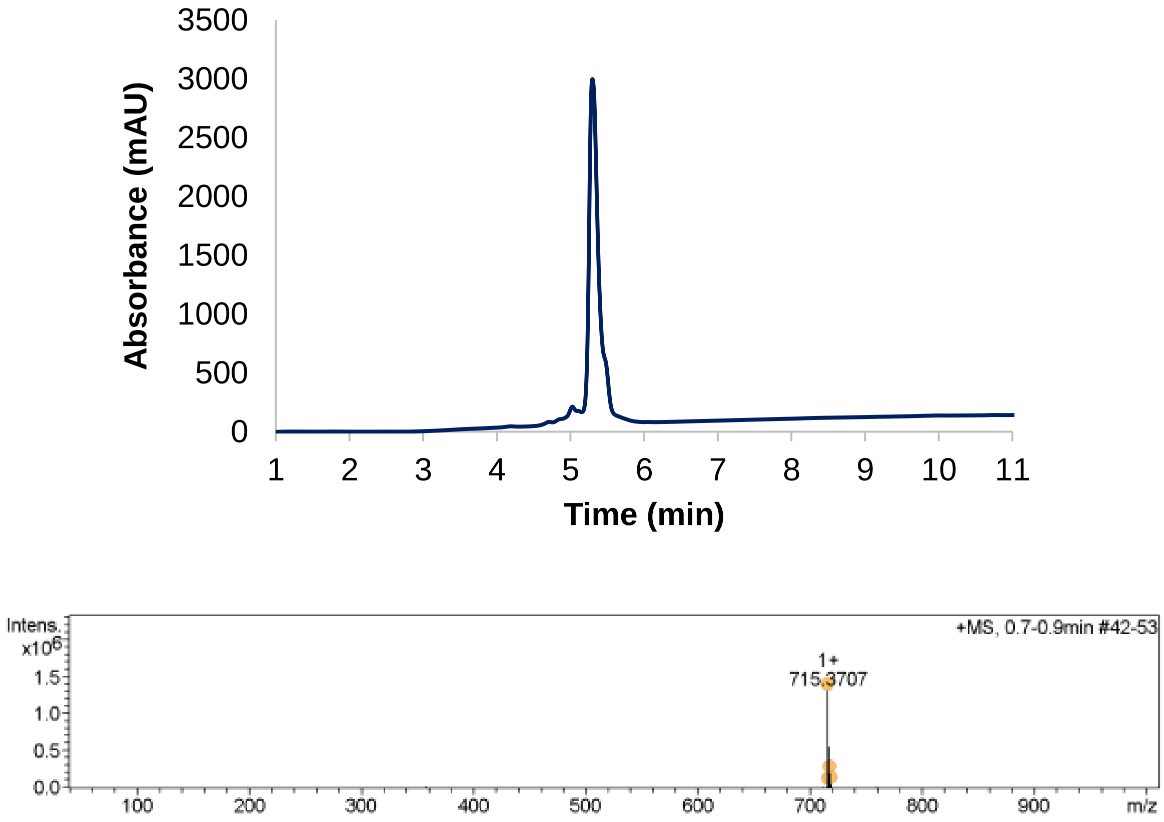

Figure S10 - Analytical HPLC trace and ESI MS for product **15**; analytical gradient 15-80% B over 10 min, 210 nm. Calculated mass $[M+H]^+$: 715.37; observed mass $[M+H]^+$: 715.37.

Ac-(alG)LYMC-NH2 (**16a**)

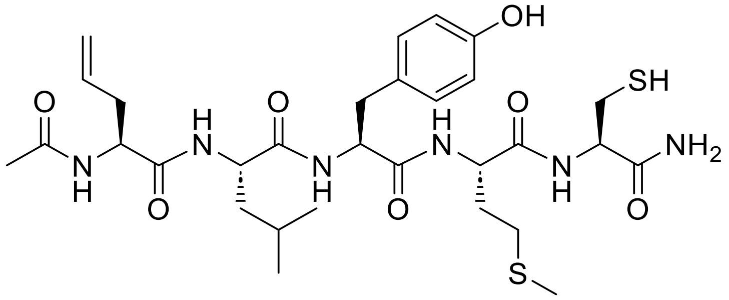

Model peptide **16a** was synthesised using the general automated synthesiser procedure with microwave assistance on Rink Amide resin (0.25 mmol). The crude peptide was purified by preparative RP-HPLC (15-80% B over 30 minutes) and lyophilized to produce the desired peptide (83 mg, 0.125 mmol, 50% yield).

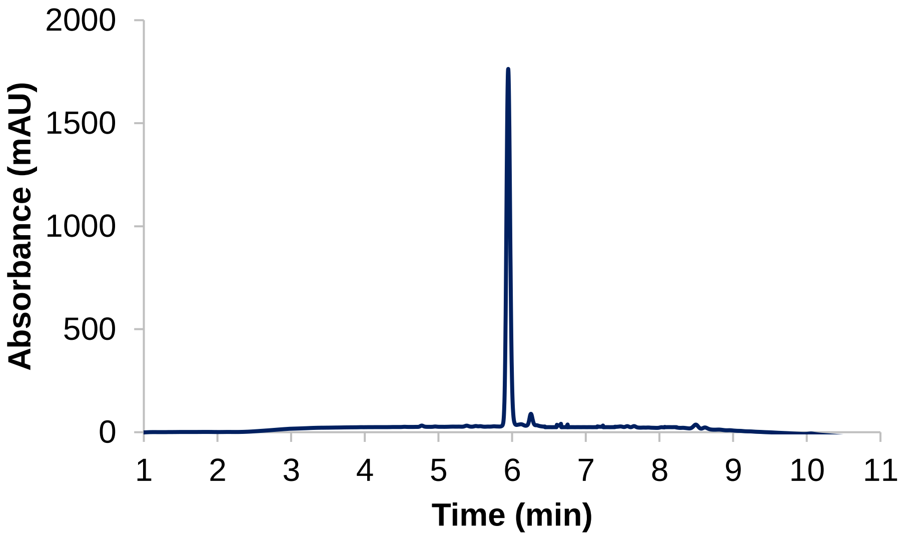

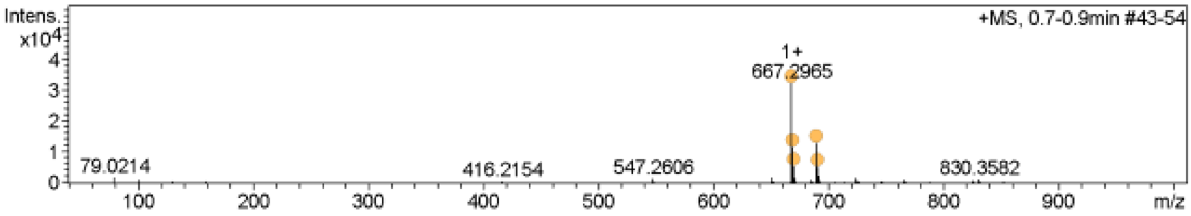

Figure S11 - Analytical HPLC trace and ESI MS for product **16a**; analytical gradient 15-80% B over 10 min, 210 nm. Calculated mass $[M+H]^+$: 667.29; observed mass $[M+H]^+$: 667.29.

H-(alG)LYMC-NH2 (**16b**)

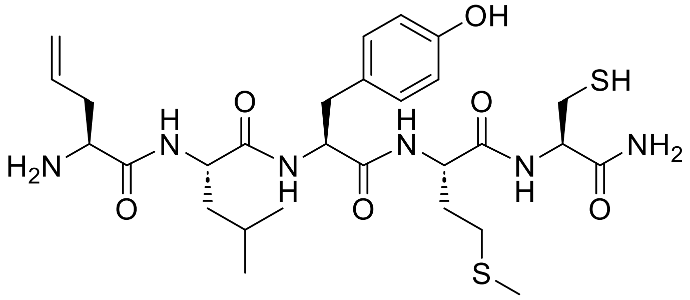

Model peptide **16b** was synthesised using the general automated synthesiser procedure with microwave assistance on Rink Amide resin (0.125 mmol). The crude peptide was purified by preparative RP-HPLC (15-80% B over 30 minutes) and lyophilized to produce the desired peptide (59 mg, 0.09 mmol, 76% yield).

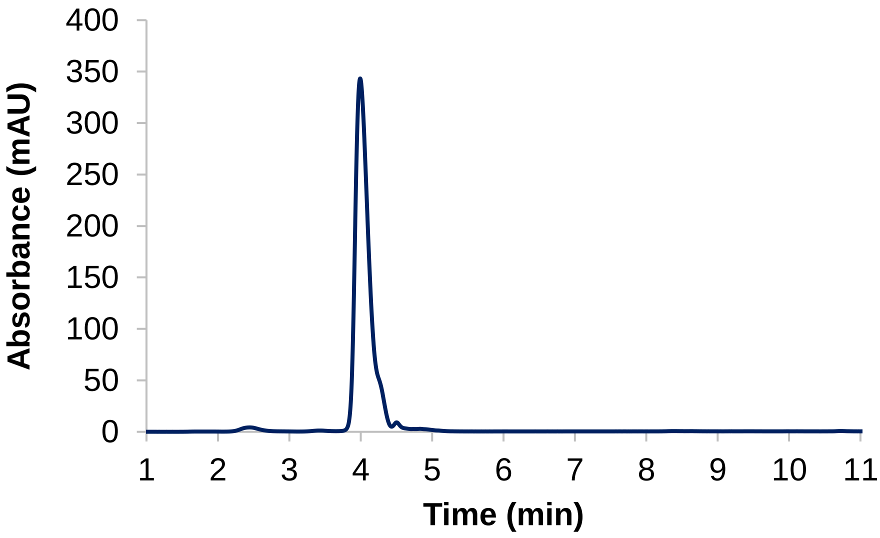

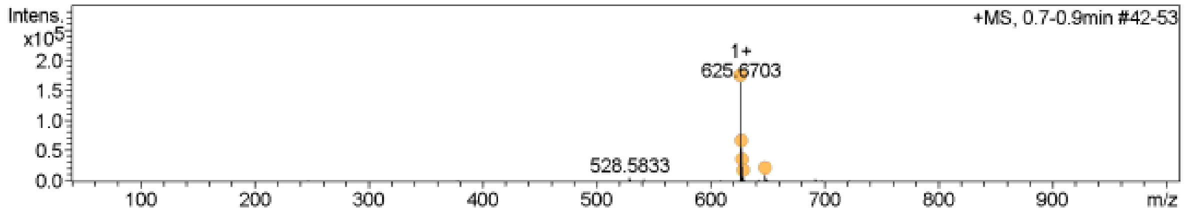

Figure S12 - Analytical HPLC trace and ESI MS for product **16b**; analytical gradient 15-80% B over 10 min, 280 nm. Calculated mass $[M+H]^+$: 625.28; observed mass $[M+H]^+$: 625.67.

Ac-(alG)VHQC-NH_2 (**17**)

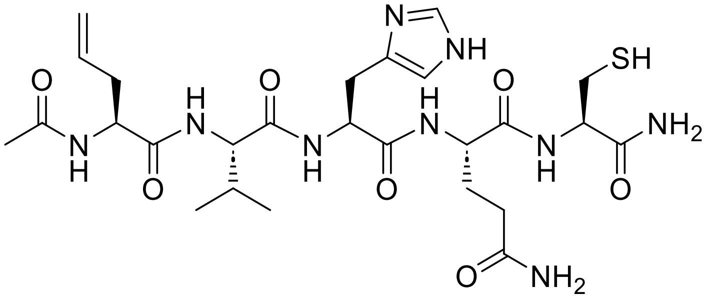

Model peptide **17** was synthesised using the general automated synthesiser procedure with microwave assistance on Rink Amide resin (0.25 mmol). The crude peptide was purified by preparative RP-HPLC (2-95% B over 30 minutes) and lyophilized to produce the desired peptide (89 mg, 0.14 mmol, 57% yield).

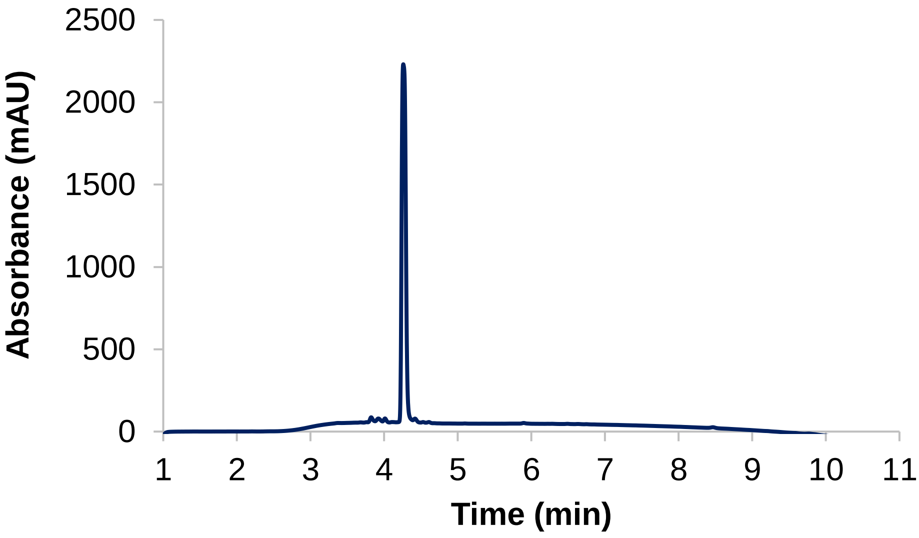

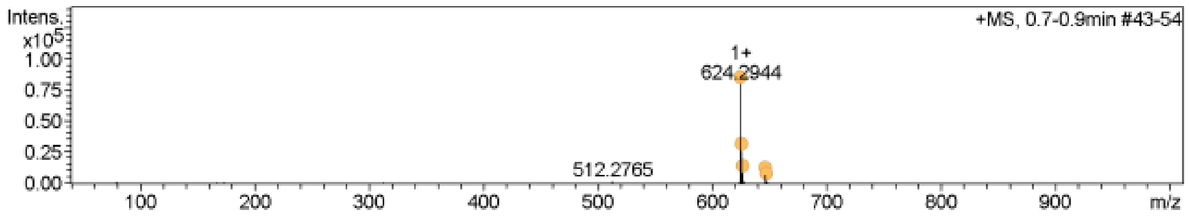

Figure S13 - Analytical HPLC trace and ESI MS for product **17**; analytical gradient 2-95% B over 10 min, 210 nm. Calculated mass $[M+H]^+$: 624.29; observed mass $[M+H]^+$: 624.29.

Ac-(alG)TPKC-NH2 (**18**)

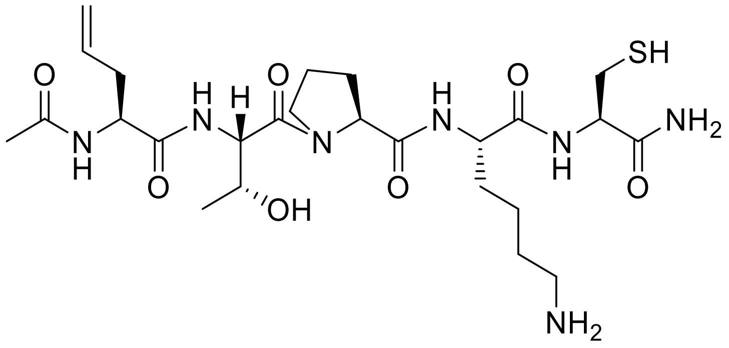

Model peptide **18** was synthesised using the general automated synthesiser procedure with microwave assistance on Rink Amide resin (0.25 mmol). The crude peptide was purified by preparative RP-HPLC (2-95% B over 30 minutes) and lyophilized to produce the desired peptide (36 mg, 0.06 mmol, 24% yield).

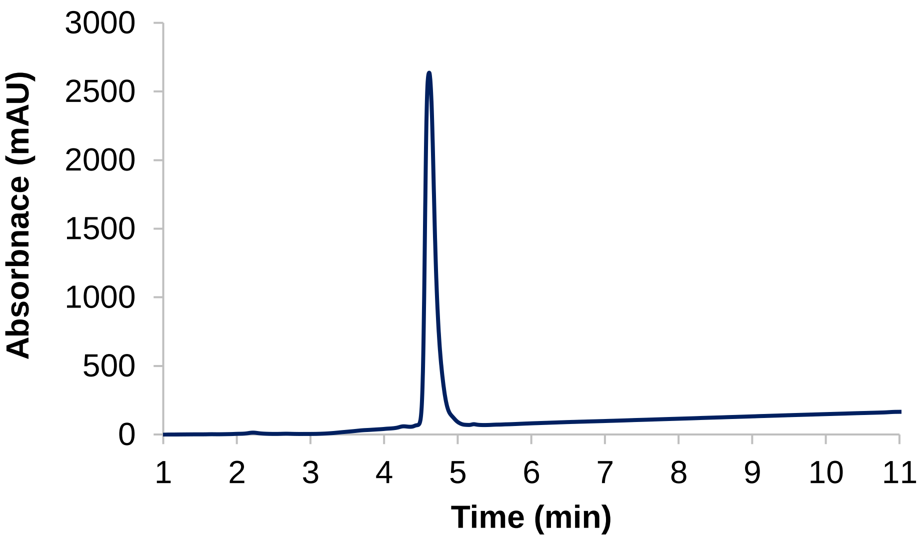

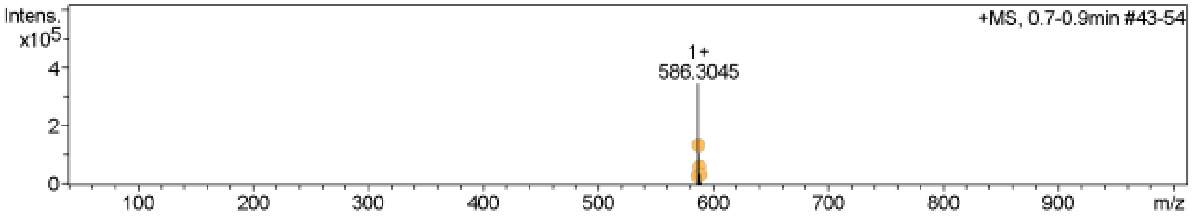

Figure S14 - Analytical HPLC trace and ESI MS for product **18**; analytical gradient 5-60% B over 10 min, 210 nm. Calculated mass $[M+H]^+$: 586.30; observed mass $[M+H]^+$: 586.30.

Ac-(alG)VGDC-NH2 (**19**)

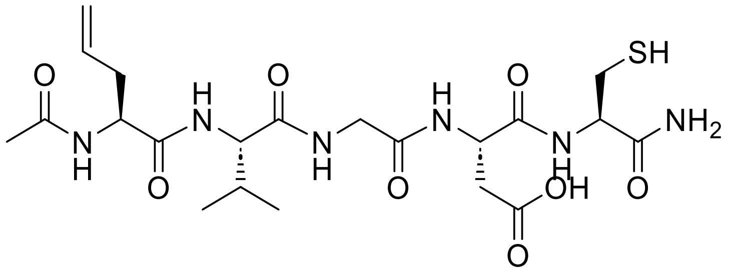

Model peptide **19** was synthesised using the general automated synthesiser procedure with microwave assistance on Rink Amide resin (0.25 mmol). The crude peptide was purified by preparative RP-HPLC (5-60% B over 30 minutes) and lyophilized to produce the desired peptide (42 mg, 0.79 mmol, 32% yield).

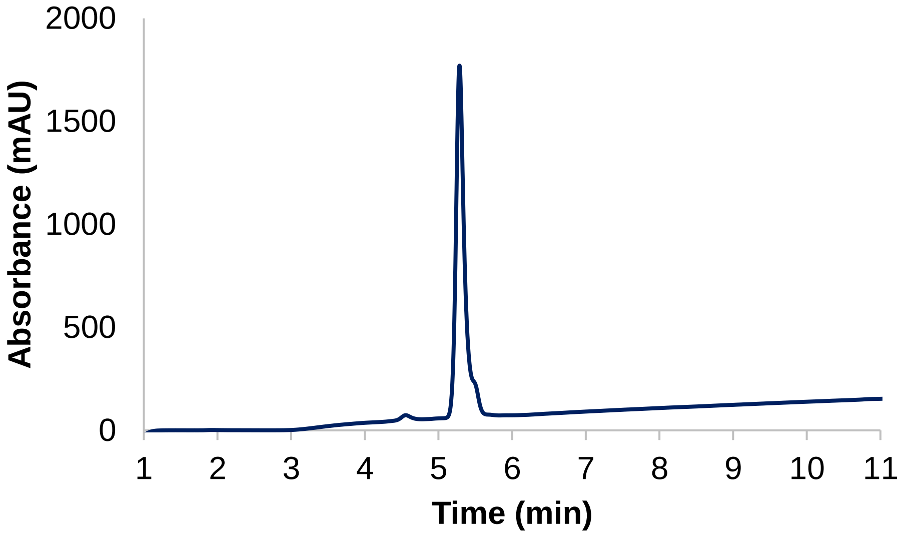

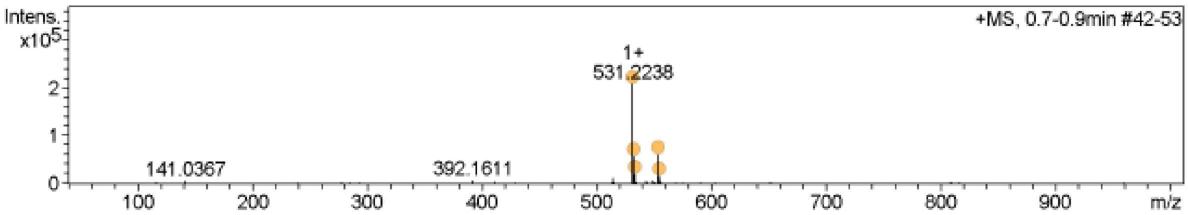

Figure S15 - Analytical HPLC trace and ESI MS for product **19**; analytical gradient 5-60% B over 10 min, 210 nm. Calculated mass $[M+H]^+$: 531.22; observed mass $[M+H]^+$: 531.55.

H-D(alG)FYQC-NH2 (**20**)

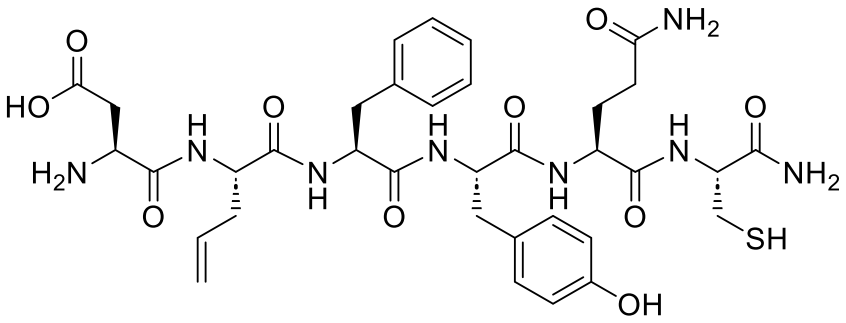

Model peptide **20** was synthesised using the general automated synthesiser procedure with microwave assistance on Rink Amide resin (0.05 mmol). The crude peptide was purified by preparative RP-HPLC (5-60% B over 30 minutes) and lyophilized to produce the desired peptide (16 mg, 20.8 µmol, 42% yield).

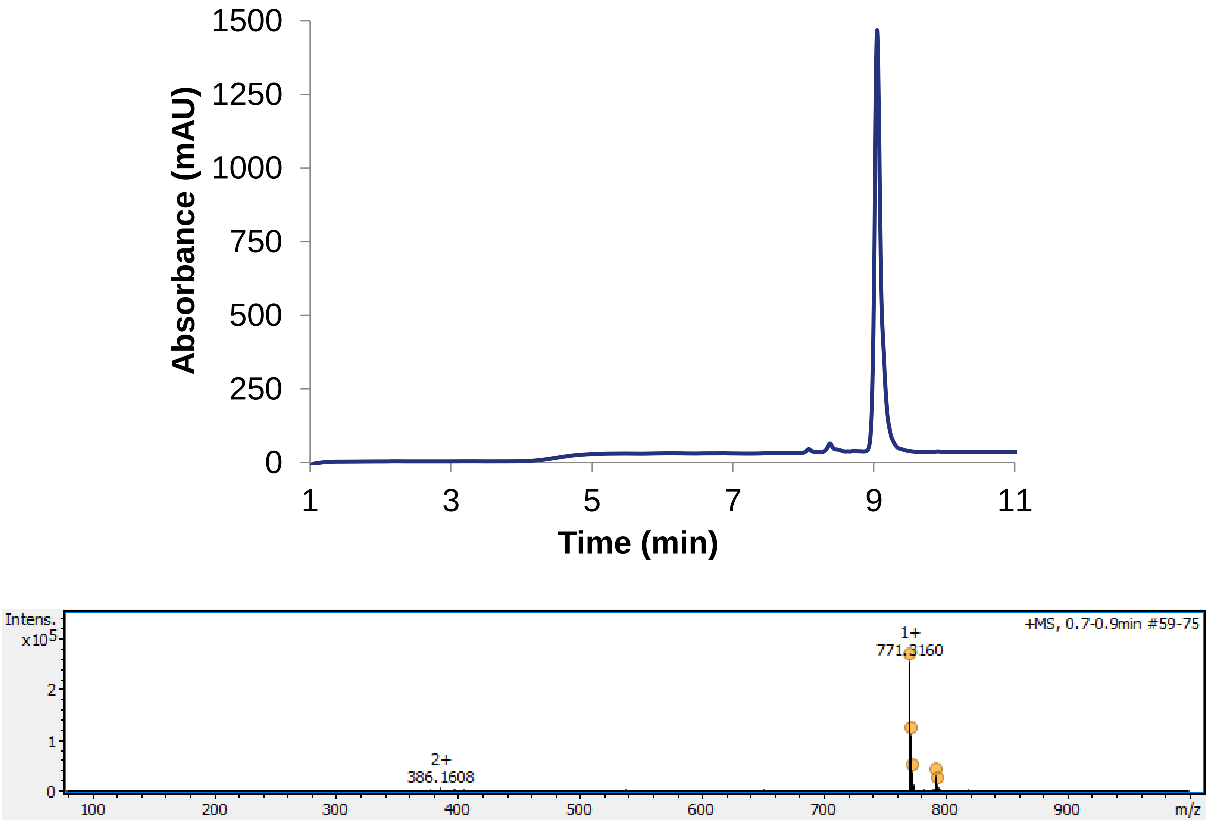

Figure S16. Analytical HPLC trace and ESI MS for product **20**; analytical gradient 2-40% B over 10 min, 210 nm. Calculated mass $[M+H]^+$: 771.31; observed mass $[M+H]^+$: 771.32.

Ac-(alG)KISYC-NH2 (**28**)

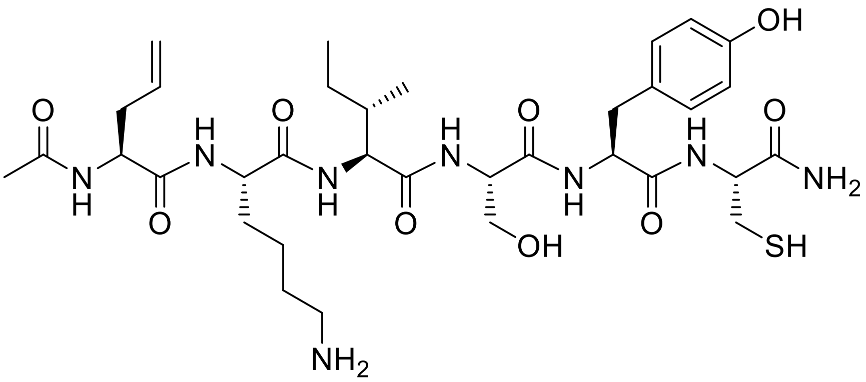

Model peptide **28** was synthesised using the general automated synthesiser procedure with microwave assistance on Rink Amide resin (0.25 mmol). The crude peptide was purified by preparative RP-HPLC (15-80% B over 30 minutes) and lyophilized to produce the desired peptide (64 mg, 0.09 mmol, 36% yield).

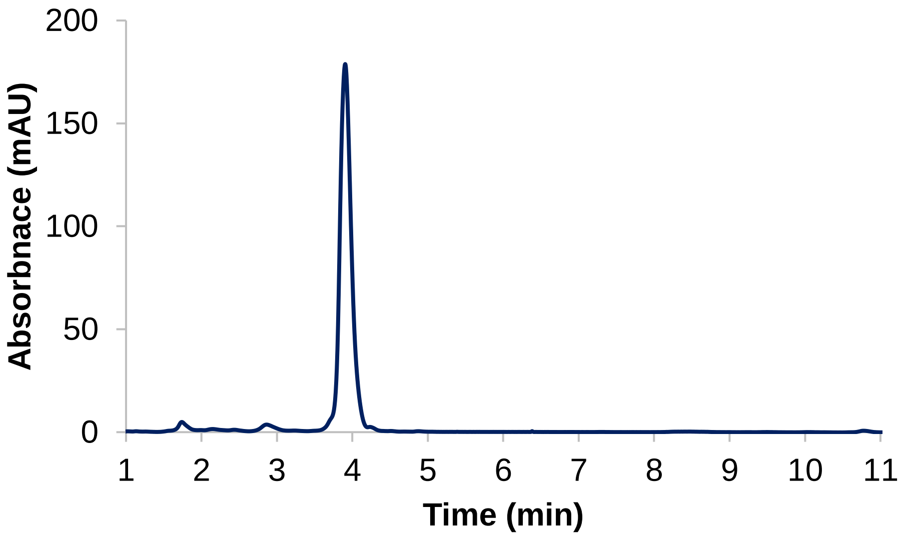

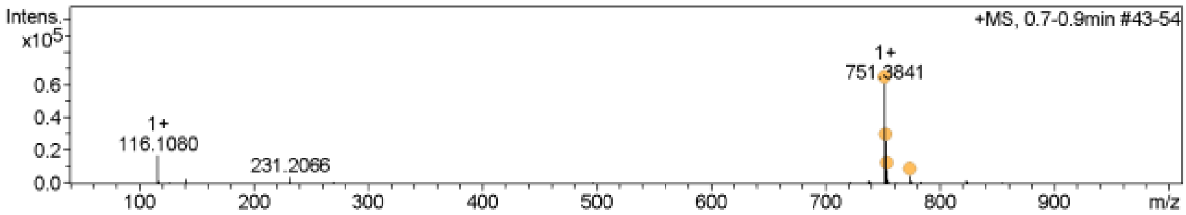

Figure S17 - Analytical HPLC trace and ESI MS for product **28**; analytical gradient 15-80% B over 10 min, 280 nm. Calculated mass $[M+H]^+$: 751.38; observed mass $[M+H]^+$: 751.38.

Ac-CKISY(alG)-NH2 (**29**)

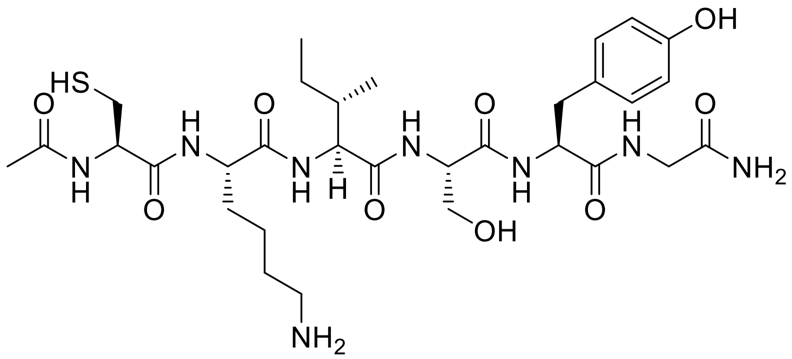

Model peptide **29** was synthesised using the general automated synthesiser procedure with microwave assistance on Rink Amide resin (0.125 mmol). The crude peptide was purified by preparative RP-HPLC (2-95% B over 30 minutes) and lyophilized to produce the desired peptide (32 mg, 0.04 µmol, 34% yield).

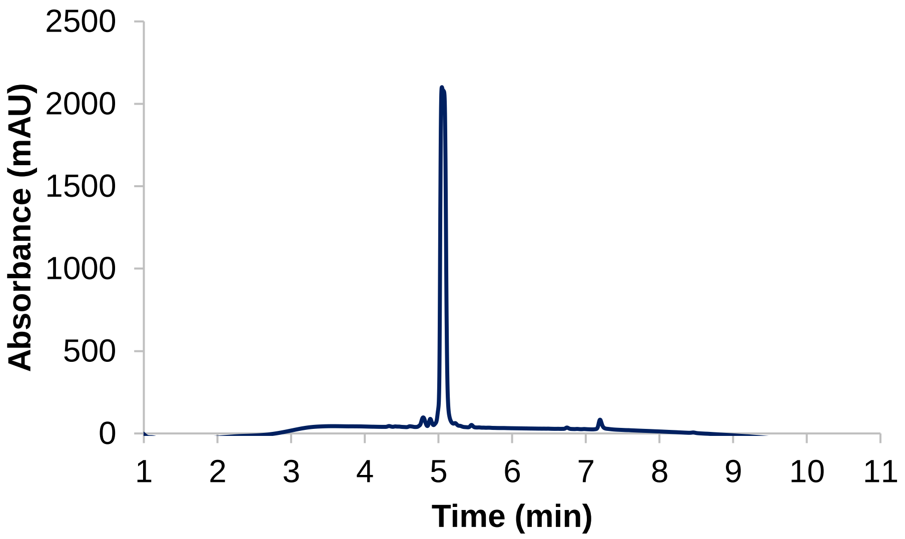

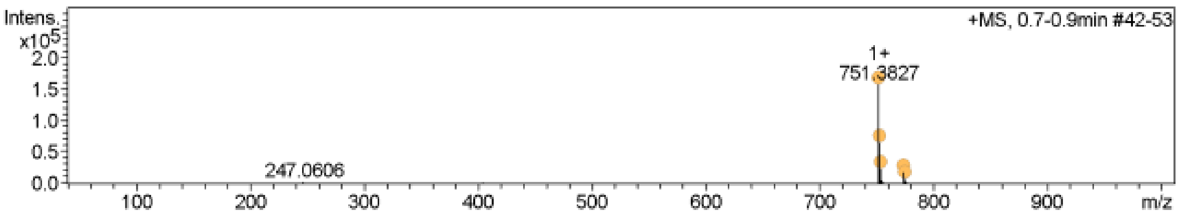

Figure S18 - Analytical HPLC trace and ESI MS for product **29**; analytical gradient 2-95% B over 10 min, 210 nm. Calculated mass $[M+H]^+$: 751.38; observed mass $[M+H]^+$: 751.38.

H-(alG)RPMHC-NH_2 (**30a**)

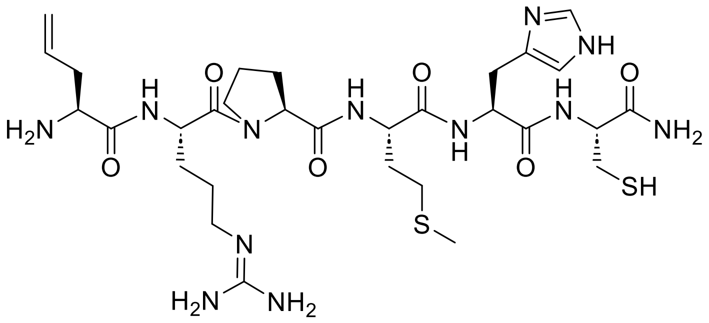

Model peptide **30a** was synthesised using the general automated synthesiser procedure with microwave assistance on Rink Amide resin (0.25 mmol). The crude peptide was purified by preparative RP-HPLC (5-60% B over 30 minutes) and lyophilized to produce the desired peptide (24.9 mg, 0.034 mmol, 18.5% yield).

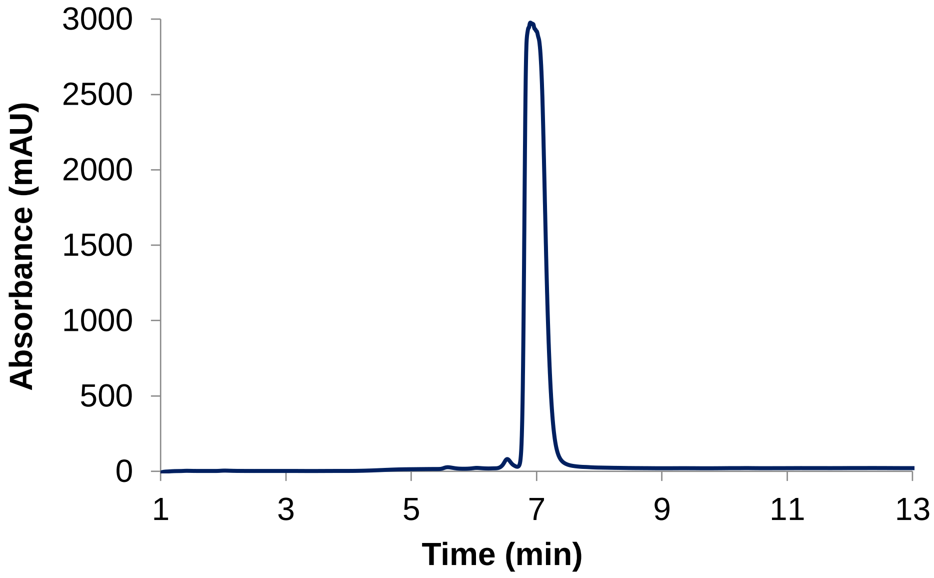

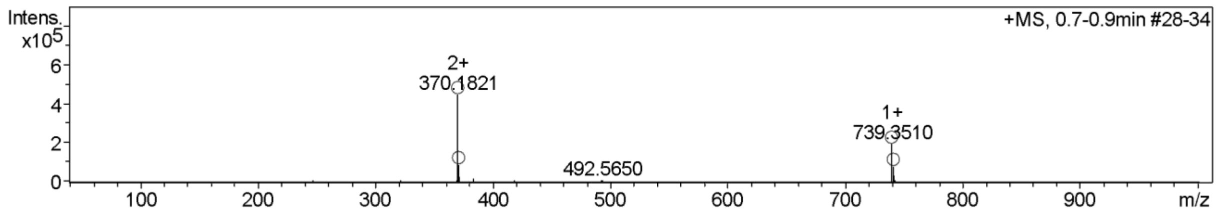

Figure S19 - Analytical HPLC trace and ESI MS for product **30a**; analytical gradient 2-95% B over 10 min, 210 nm. Calculated mass $[M+H]^+$: 751.38; observed mass $[M+H]^+$: 751.38.

Ac-(alG)RPMHC-NH2 (**30b**)

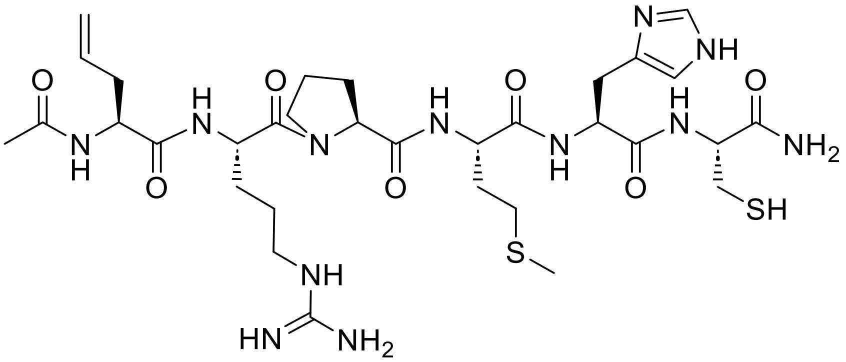

Model peptide **30b** was synthesised using the general automated synthesiser procedure with microwave assistance on Rink Amide resin (0.125 mmol). The crude peptide was purified by preparative RP-HPLC (5-60% B over 30 minutes) and lyophilized to produce the desired peptide (27 mg, 0.03 µmol, 28% yield).

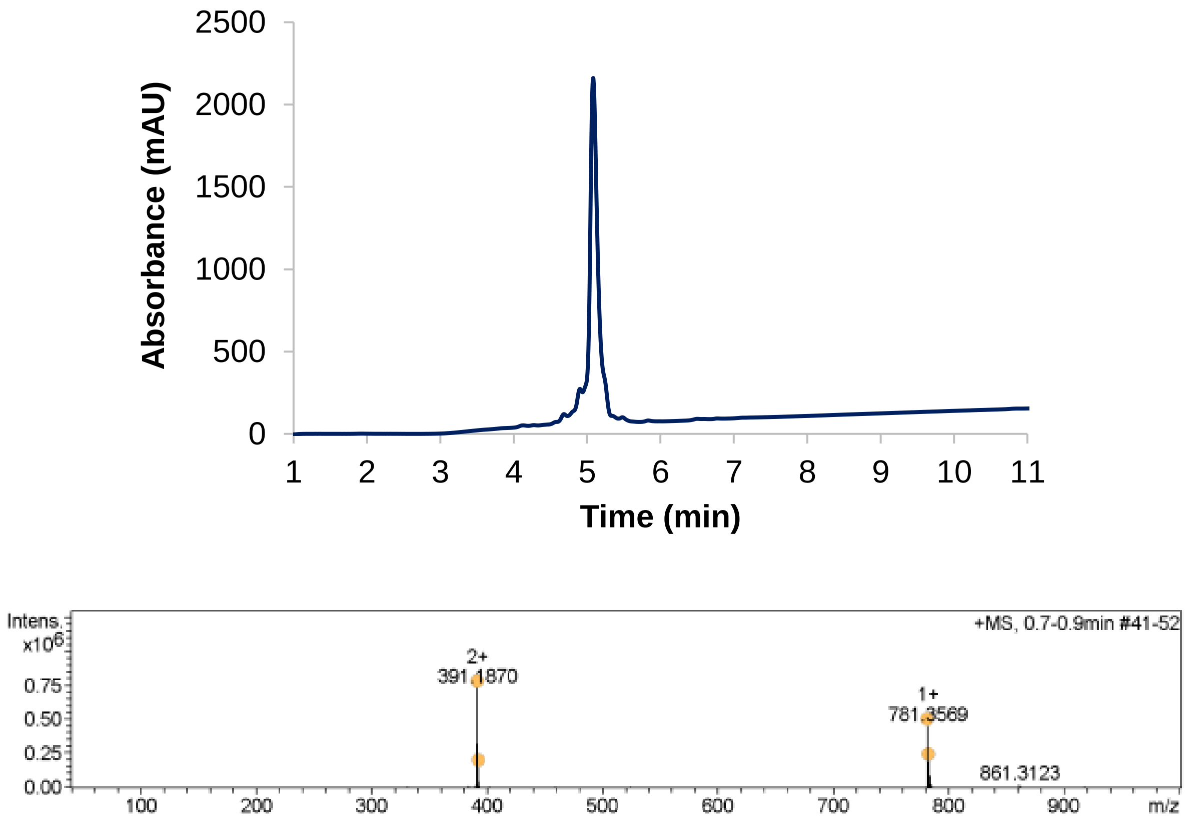

Figure S20 - Analytical HPLC trace and ESI MS for product **30b**; analytical gradient 5-60% B over 10 min, 210 nm. Calculated mass $[M+H]^+$: 781.36; observed mass $[M+H]^+$: 781.36.

Ac-(alG)ELQWC-NH2 (**31**)

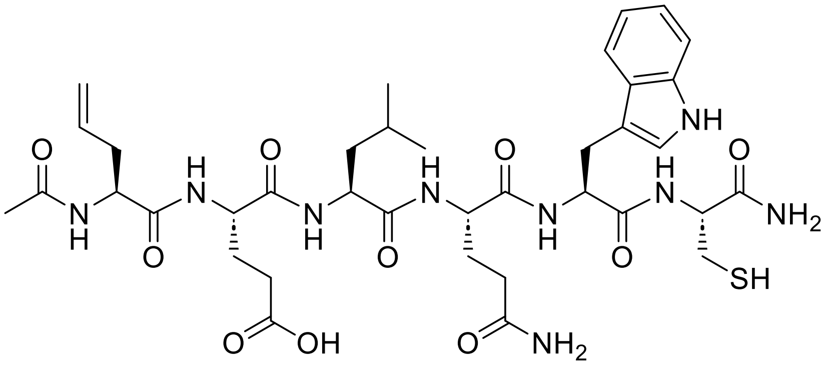

Model peptide **31** was synthesised using the general automated synthesiser procedure with microwave assistance on Rink Amide resin (0.25 mmol). The crude peptide was purified by preparative RP-HPLC (2-95% B over 30 minutes) and lyophilized to produce the desired peptide (43 mg, 0.05 mmol, 20% yield).

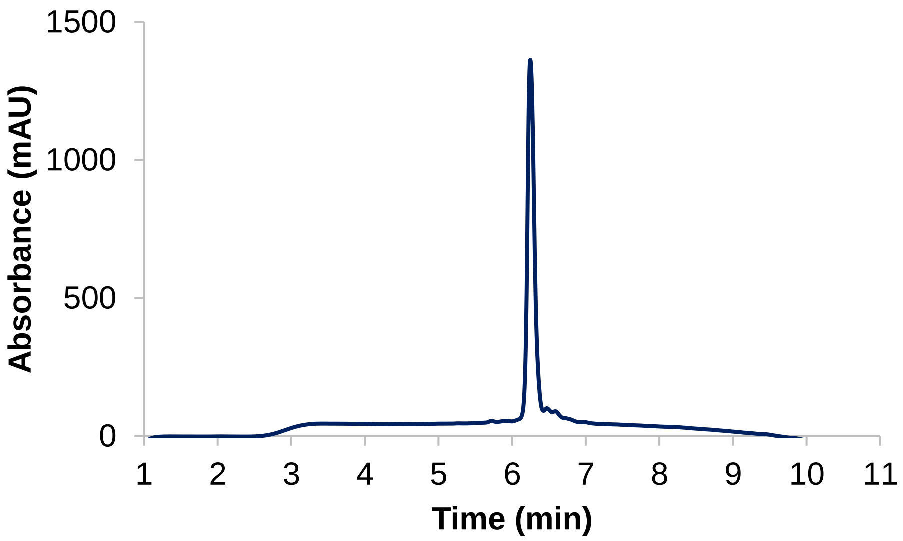

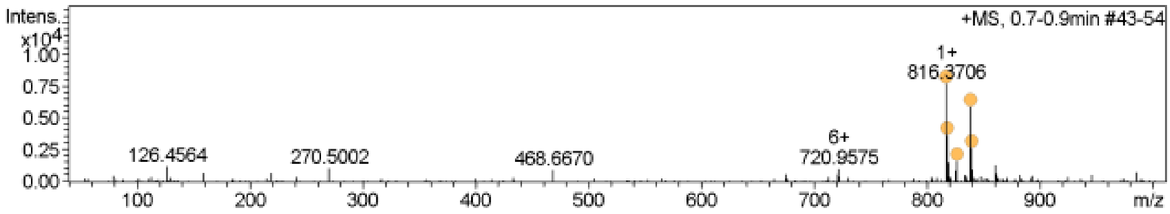

Figure S21 - Analytical HPLC trace and ESI MS for product **31**; analytical gradient 2-95% B over 10 min, 210 nm. Calculated mass $[M+H]^+$: 816.37; observed mass $[M+H]^+$: 816.37.

Ac-(alG)DSYKIC-NH2 (**32**)

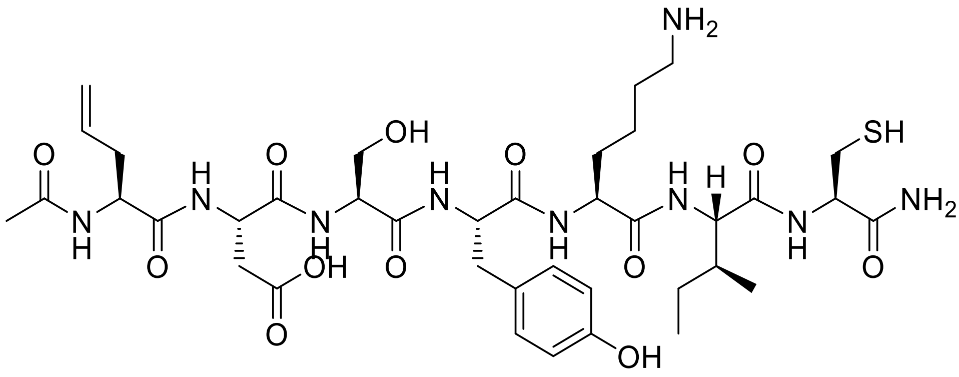

Model peptide **32** was synthesised using the general automated synthesiser procedure with microwave assistance on Rink Amide resin (0.125 mmol). The crude peptide was purified by preparative RP-HPLC (5-60% B over 30 minutes) and lyophilized to produce the desired peptide (42 mg, 0.05 µmol, 39% yield).

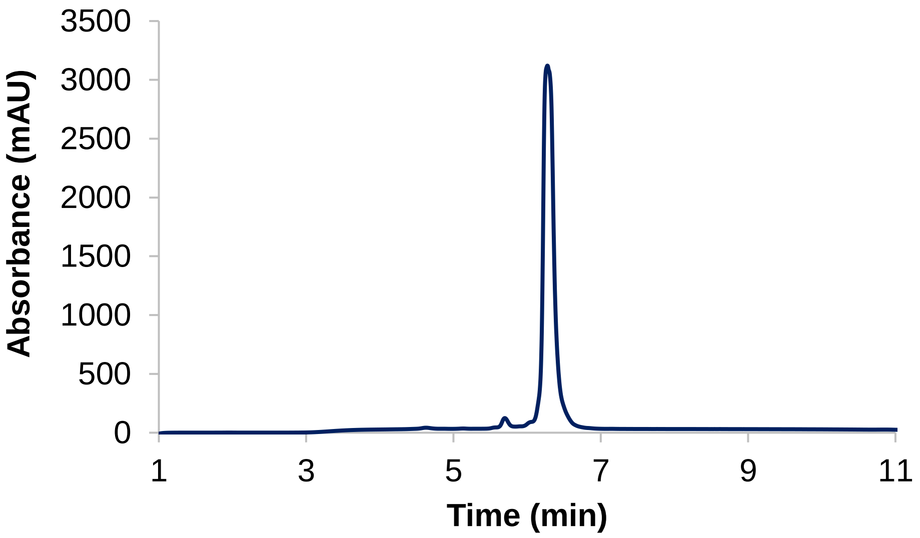

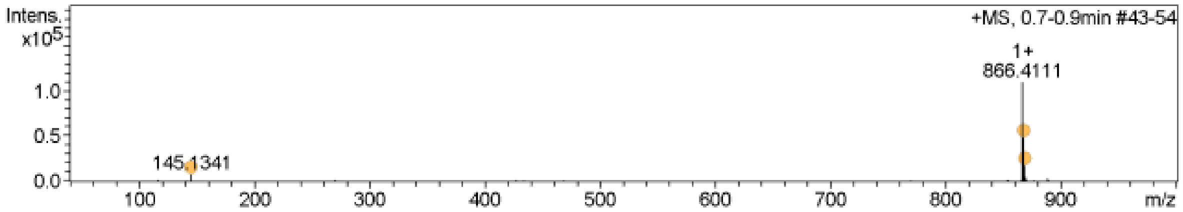

Figure S22 - Analytical HPLC trace and ESI MS for product **32**; analytical gradient 5-60% B over 10 min, 210 nm. Calculated mass $[M+H]^+$: 866.41; observed mass $[M+H]^+$: 866.41.

Ac-(alG)NHPRVC-NH2 (**33**)

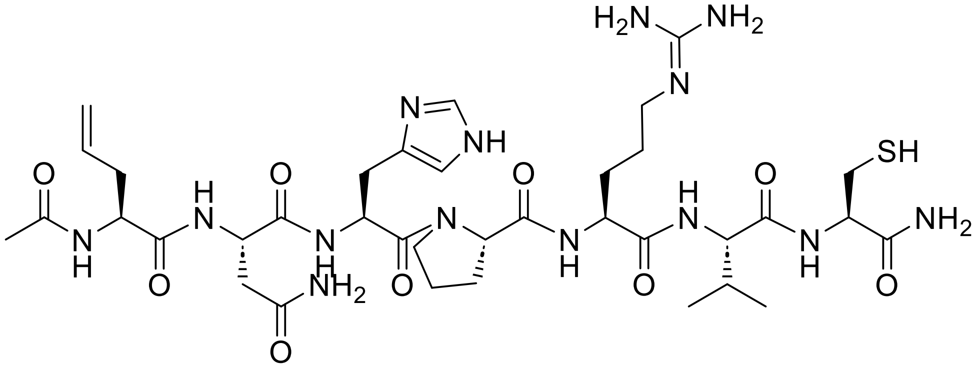

Model peptide **33** was synthesised using the general automated synthesiser procedure with microwave assistance on Rink Amide resin (0.125 mmol). The crude peptide was purified by preparative RP-HPLC (5-60% B over 30 minutes) and lyophilized to produce the desired peptide (29 mg, 0.04 mmol, 29% yield).

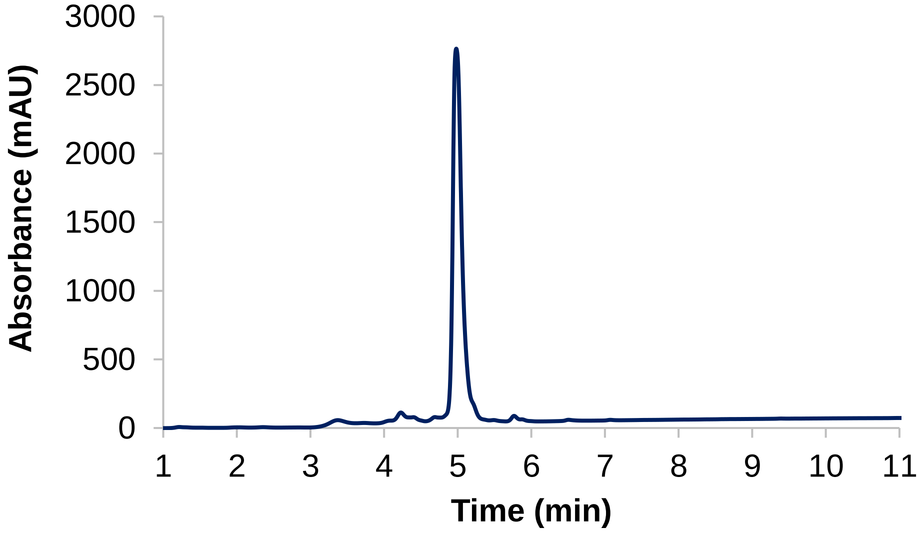

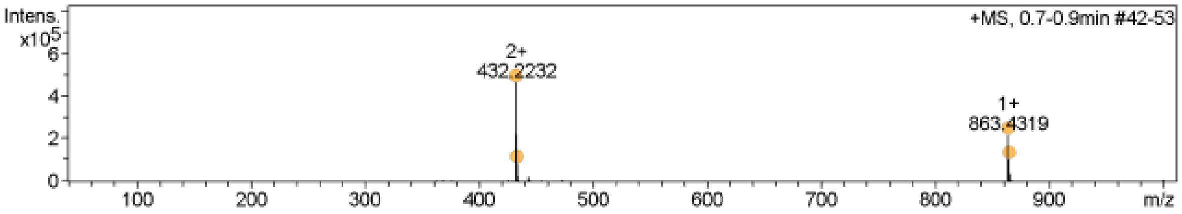

Figure S23 - Analytical HPLC trace and ESI MS for product **33**; analytical gradient 5-60% B over 10 min, 210 nm. Calculated mass $[M+H]^+$: 863.43; observed mass $[M+H]^+$: 863.43.

H-(alG)MALEKC-NH_2 (**34**)

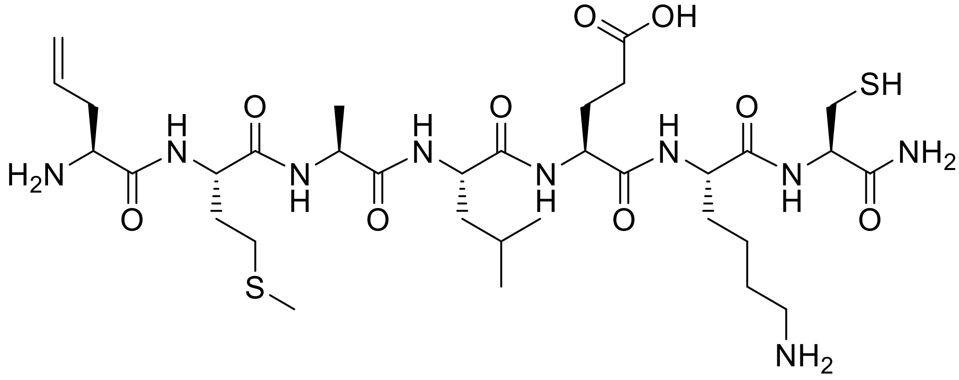

Model peptide **34** was synthesised using the general automated synthesiser procedure with microwave assistance on Rink Amide resin (0.1 mmol). The crude peptide was purified by preparative RP-HPLC (2-95% B over 30 minutes) and lyophilized to produce the desired peptide (yield not calculated).

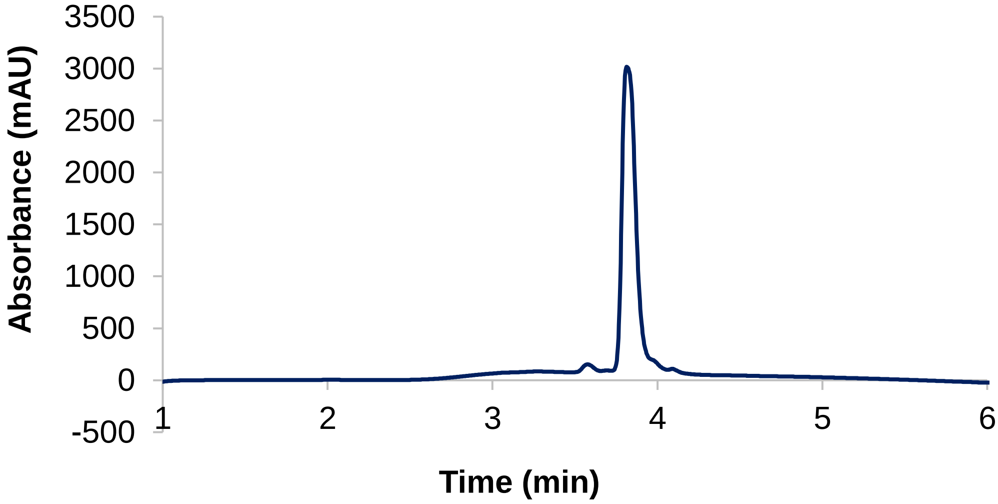

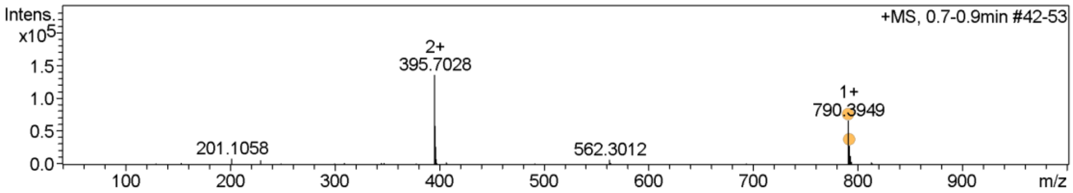

Figure S24 - Analytical HPLC trace and ESI MS for product **34**; analytical gradient 2-95% B over 5 min, 210 nm. Calculated mass $[M+H]^+$: 790.39; observed mass $[M+H]^+$: 790.39.

H-(alG)MPWLRC-NH2 (**35**)

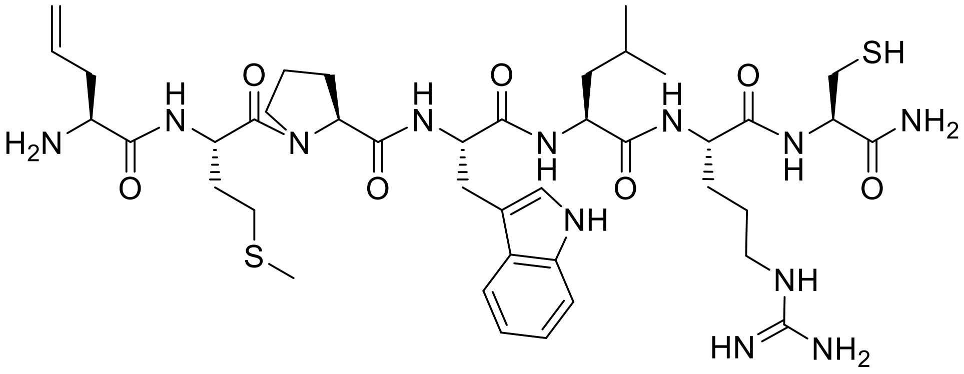

Model peptide **35** was synthesised using the general automated synthesiser procedure with microwave assistance on Rink Amide resin (0.05 mmol). The crude peptide was purified by preparative RP-HPLC (5-60% B over 30 minutes) and lyophilized to produce the desired peptide (12 mg, 13.3 µmol, 27% yield).

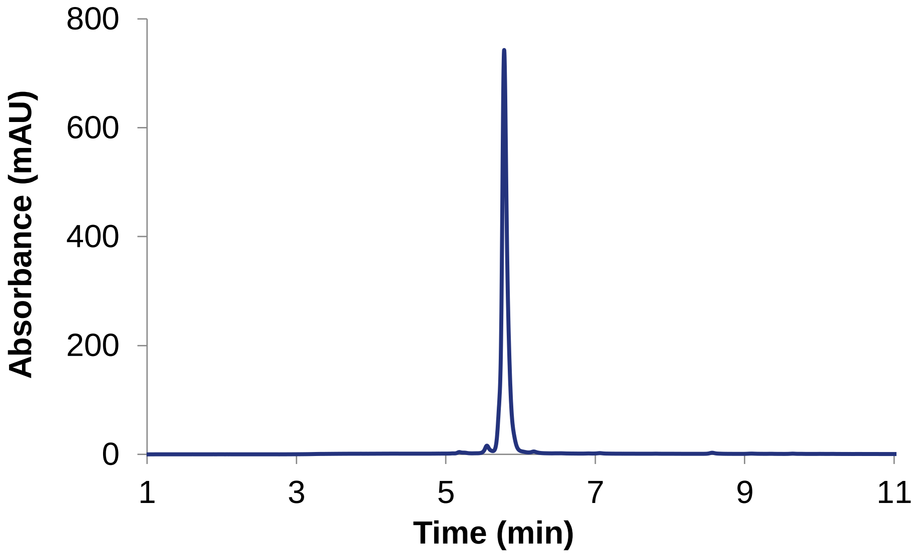

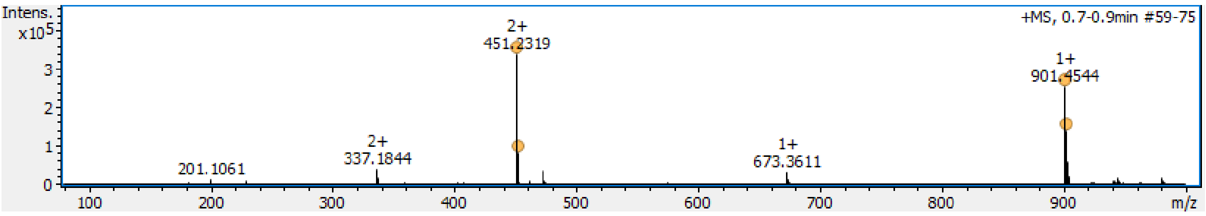

Figure S25. Analytical HPLC trace and ESI MS for product **35**; analytical gradient 10-80% B over 10 min, 210 nm. Calculated mass $[M+H]^{+}$: 901.45, $[M+2H]^{2+}$: 451.23; observed mass $[M+H]^{+}$: 901.45, $[M+2H]^{2+}$: 451.23.

H-(alG)EDLFYQC-NH_2 (**36**)

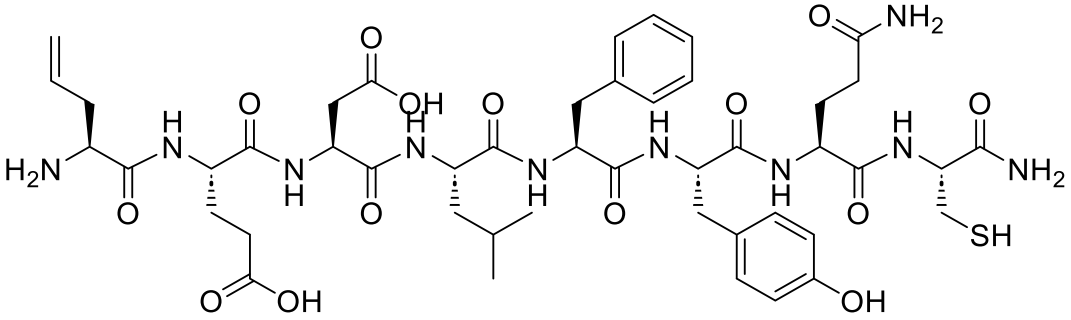

Model peptide **36** was synthesised using the general automated synthesiser procedure with microwave assistance on Rink Amide resin (0.05 mmol). The crude peptide was purified by preparative RP-HPLC (10-70% B over 30 minutes) and lyophilized to produce the desired peptide (19 mg, 18.8 µmol, 38% yield).

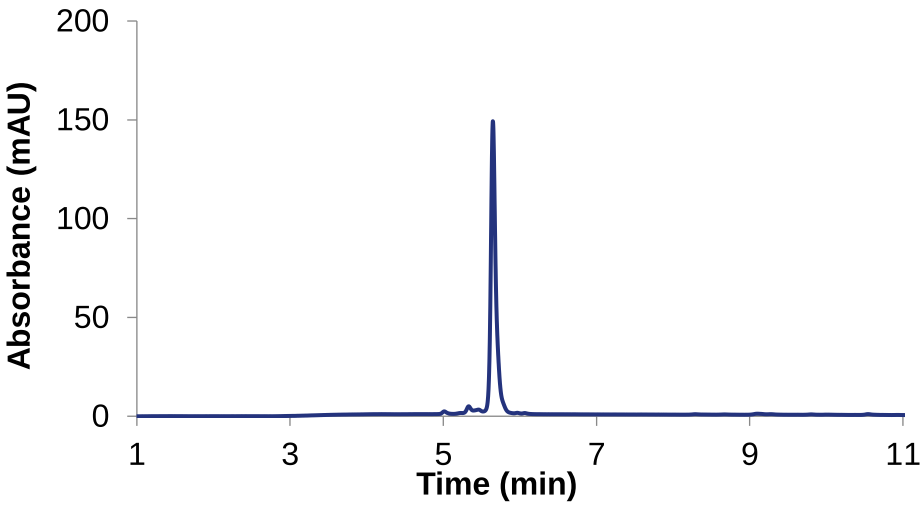

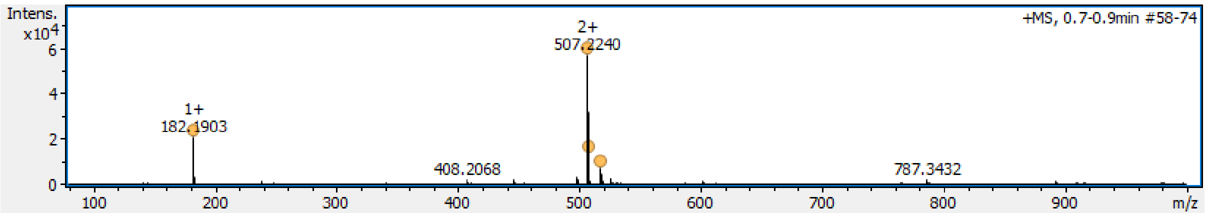

Figure S26. Analytical HPLC trace and ESI MS for product **36**; analytical gradient 10-80% B over 10 min, 210 nm. Calculated mass $[M+2H]^{2+}$: 507.24; observed mass $[M+2H]^{2+}$: 507.24.

H-(alG)DKFNHEC-NH2 (**37**)

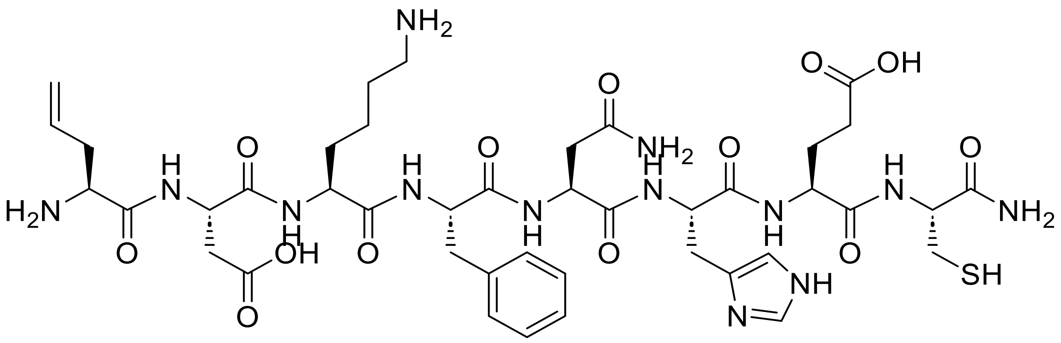

Model peptide **37** was synthesised using the general automated synthesiser procedure with microwave assistance on Rink Amide resin (0.05 mmol). The crude peptide was purified by preparative RP-HPLC (10-70% B over 30 minutes) and lyophilized to produce the desired peptide (14 mg, 14.2 μmol, 28% yield).

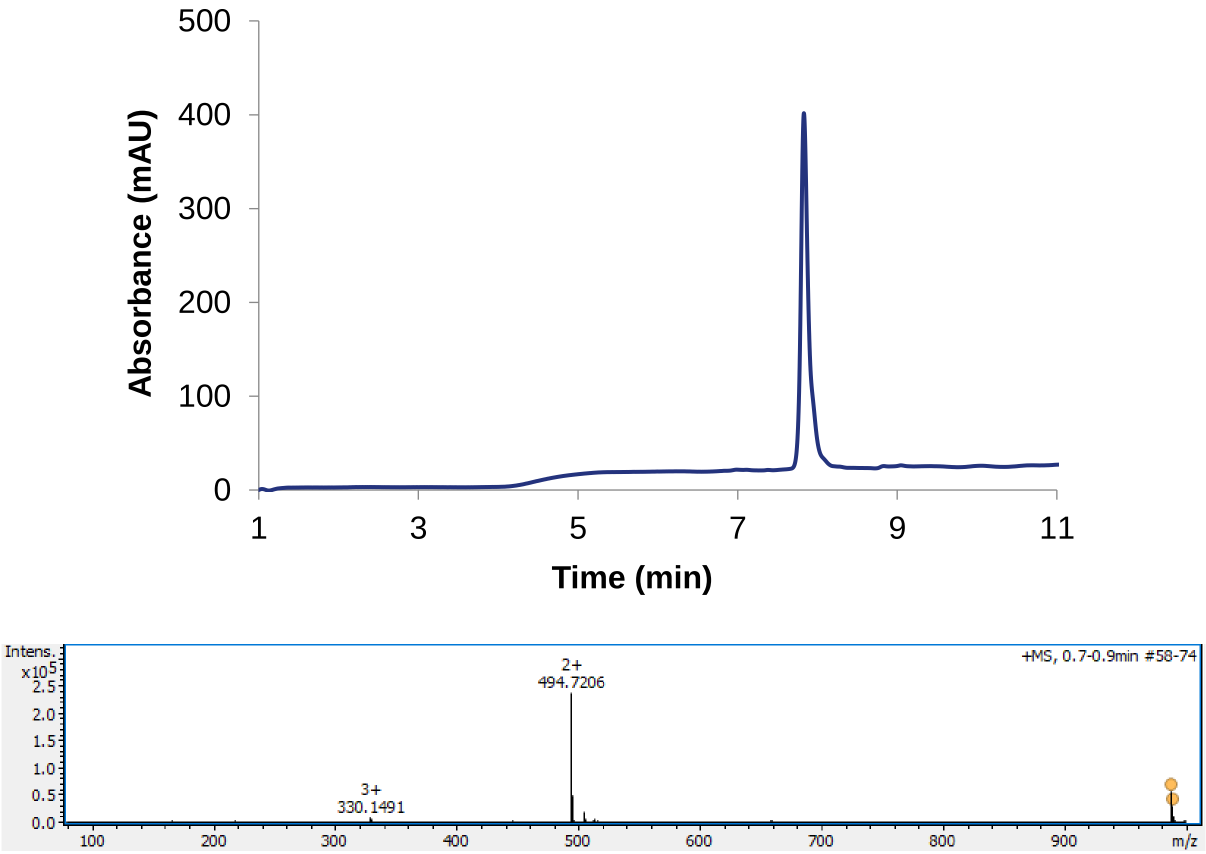

Figure S27. Analytical HPLC trace and ESI MS for product **37**; analytical gradient 2-25% B over 10 min, 210 nm. Calculated mass $[M+2H]^{2+}$: 494.72; observed mass $[M+2H]^{2+}$: 494.72.

Ac-(alG)QVGDSNLC-NH2 (**38**)

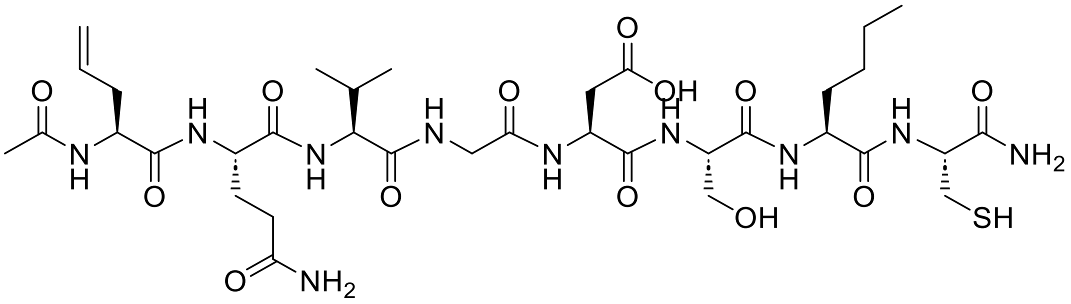

Model peptide **38** was synthesised using the general automated synthesiser procedure with microwave assistance on Rink Amide resin (0.1 mmol). The crude peptide was purified by preparative RP-HPLC (2-95% B over 30 minutes) and lyophilized to produce the desired peptide (yield not calculated).

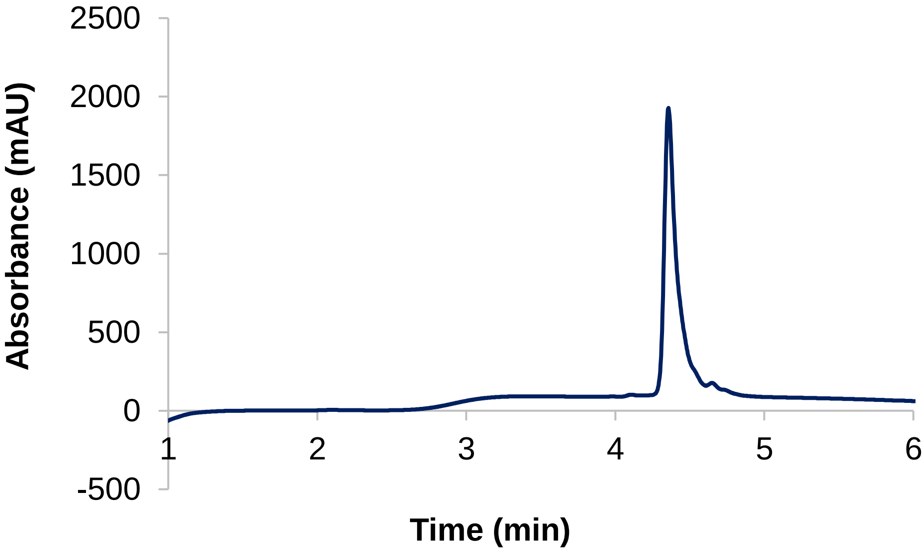

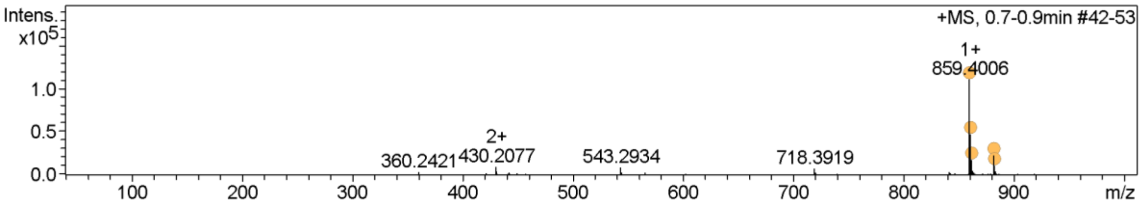

Figure S28. Analytical HPLC trace and ESI MS for product **38**; analytical gradient 2-95% B over 5 min, 210 nm. Calculated mass $[M+H]^+$: 859.97; observed mass $[M+H]^+$: 859.40.

H-(alG)YEPLAWHISKEYC-NH2 (**49**)

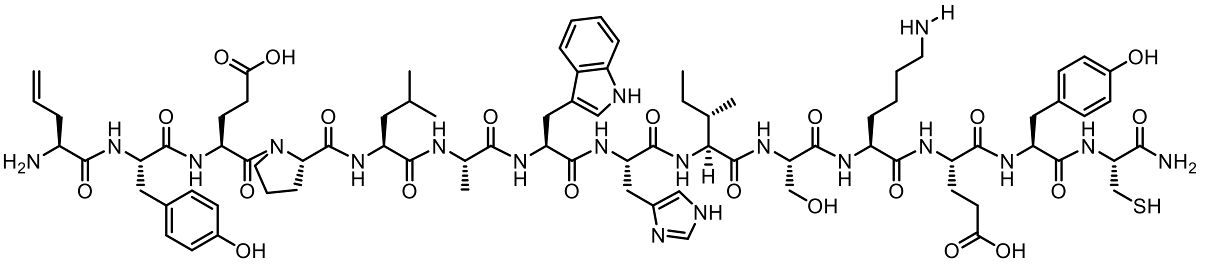

Model peptide **49** was synthesised using the general automated synthesiser procedure with microwave assistance on Rink Amide resin (0.05 mmol). The crude peptide was purified by preparative RP-HPLC (5-60% B over 30 minutes) and lyophilized to produce the desired peptide (20 mg, 11.5 µmol, 23% yield).

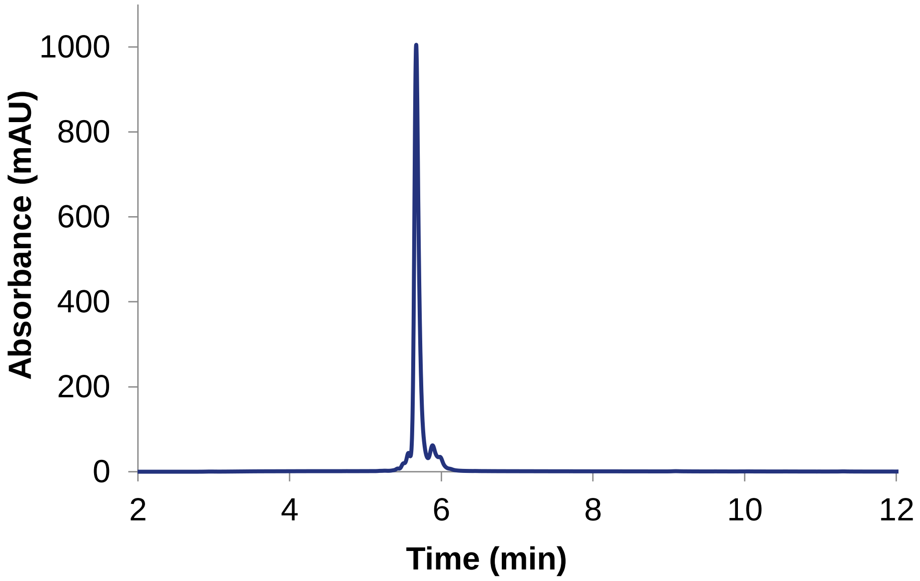

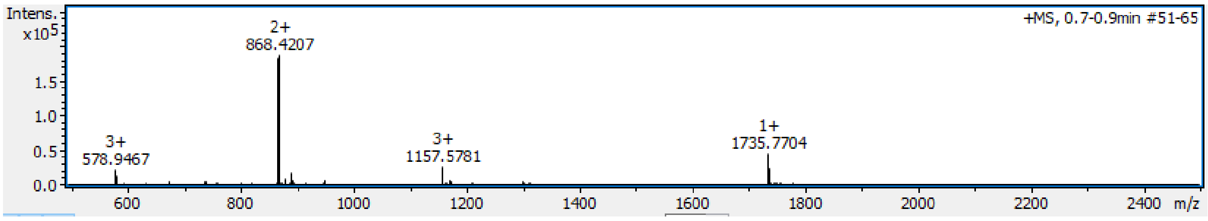

Figure S29. Analytical HPLC trace and ESI MS for product **49**; analytical gradient 10-80% B over 10 min, 210 nm. Calculated Mass $[M+H]^+$: 1735.8392, $[M+2H]^{2+}$: 868.4235. Observed Mass $[M+H]^+$: 1735.7704, $[M+2H]^{2+}$: 868.4207.

H-(alG)YIQNCPLG-NH2 (**51**) (linear Oxytocin)

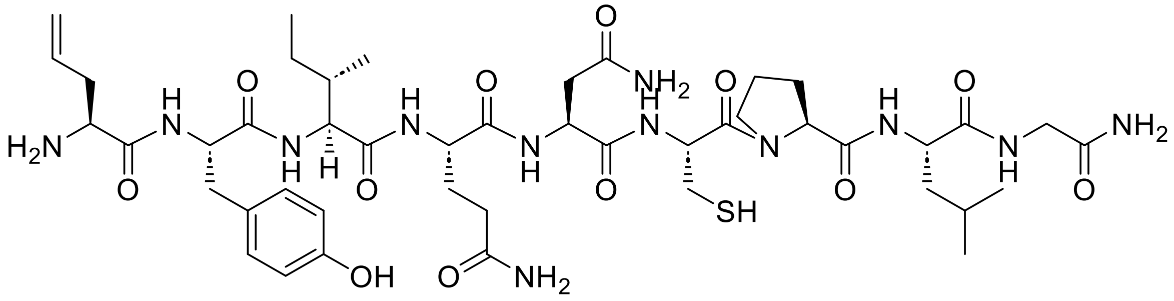

Model peptide **51** was synthesised using the general automated synthesiser procedure with microwave assistance on Rink Amide resin (0.05 mmol). The crude peptide was purified by preparative RP-HPLC (10-70% B over 30 minutes) and lyophilized to produce the desired peptide (18 mg, 18.0 µmol, 36% yield).

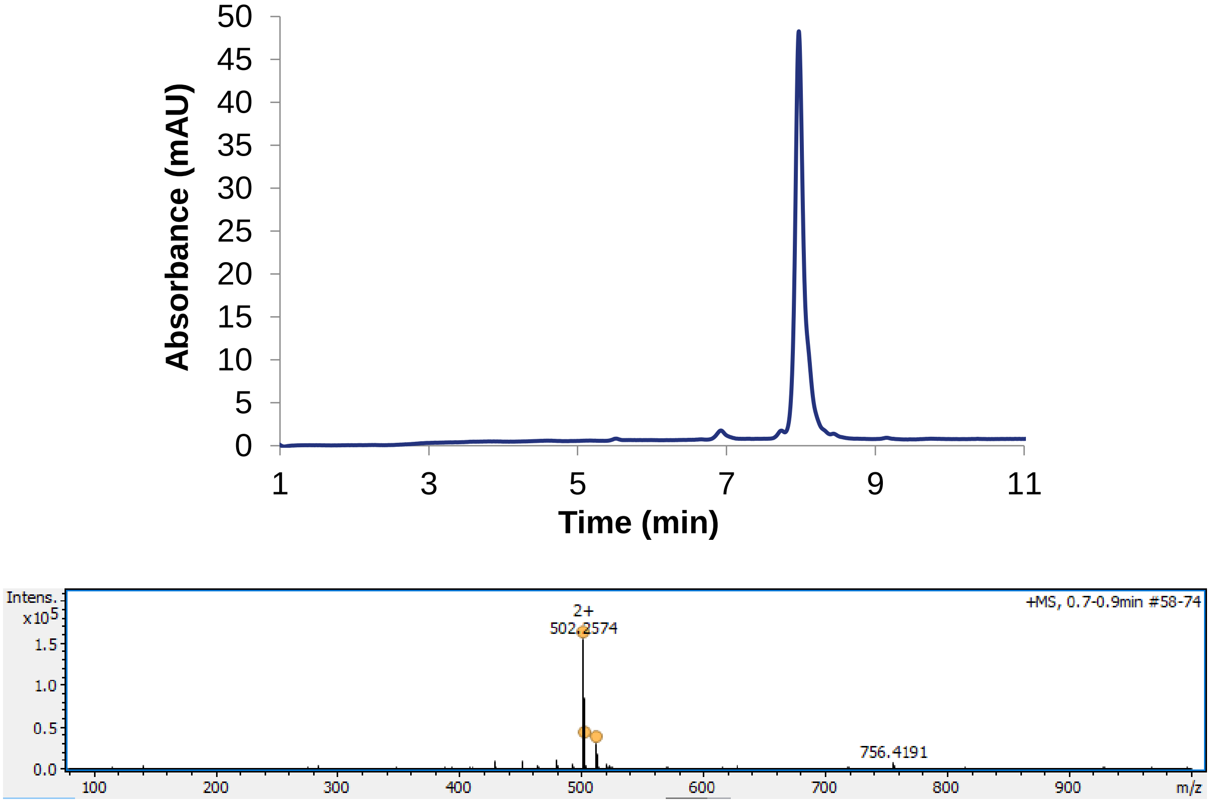

Figure S30. Analytical HPLC trace and ESI MS for product **51**; analytical gradient 10-30% B over 10 min, 210 nm. Calculated mass $[M+2H]^{2+}$: 502.26; observed mass $[M+2H]^{2+}$: 502.26.

H-CYIQN(alG)PLG-NH2 (**52**) (linear Oxytocin)

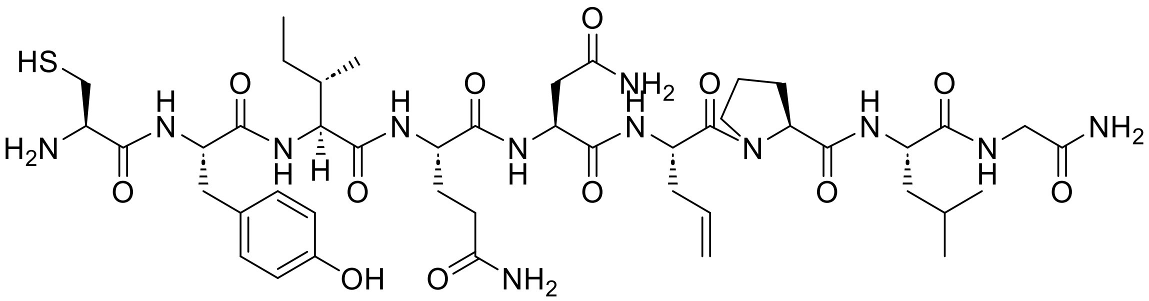

Model peptide **52** was synthesised using the general automated synthesiser procedure with microwave assistance on Rink Amide resin (0.05 mmol). The crude peptide was purified by preparative RP-HPLC (10-70% B over 30 minutes) and lyophilized to produce the desired peptide (20 mg, 20.0 µmol, 40% yield).

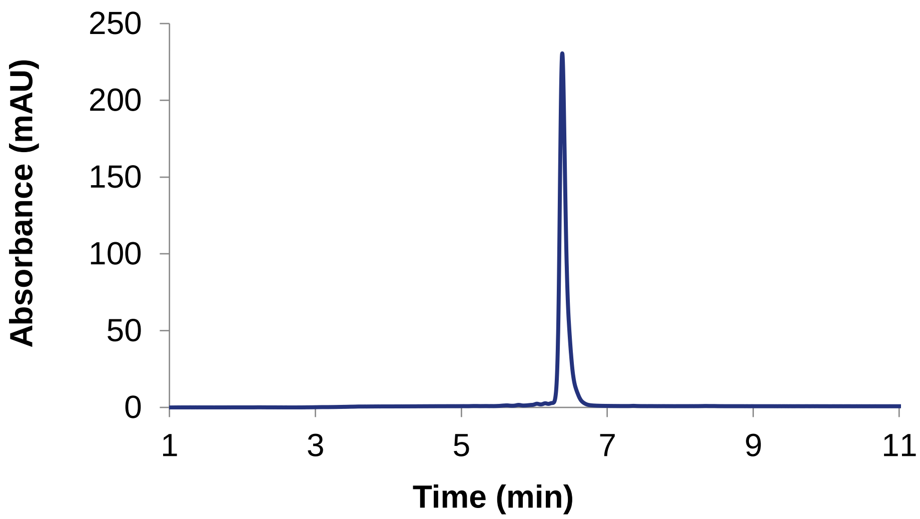

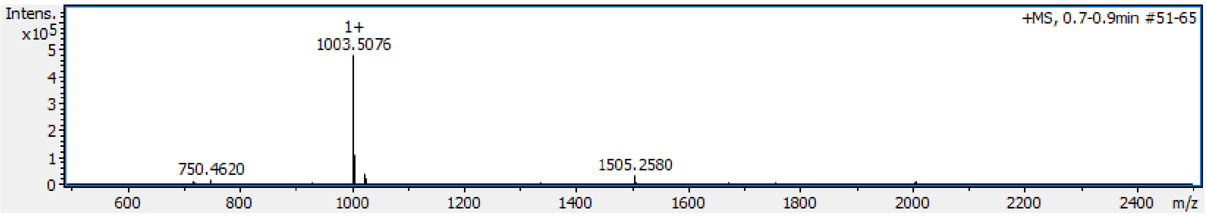

Figure S31. Analytical HPLC trace and ESI MS for product **52**; analytical gradient 10-30% B over 10 min, 210 nm. Calculated mass $[M+2H]^{2+}$: 1003.50; observed mass $[M+2H]^{2+}$: 1003.51.

Ac-HLA(alG)VGDCN_LDR-NH_2 (**54**)

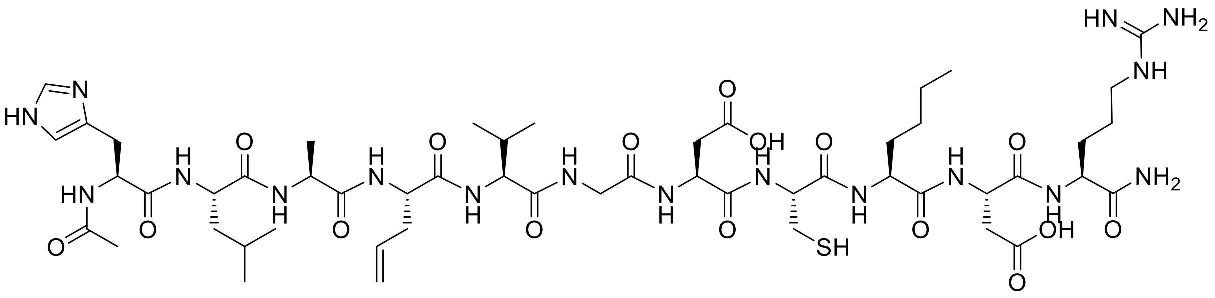

Model peptide **54** was synthesised using the general automated synthesiser procedure with microwave assistance on Rink Amide resin (0.1 mmol). The crude peptide was purified by preparative RP-HPLC (2-95% B over 30 minutes) and lyophilized to produce the desired peptide (yield not calculated).

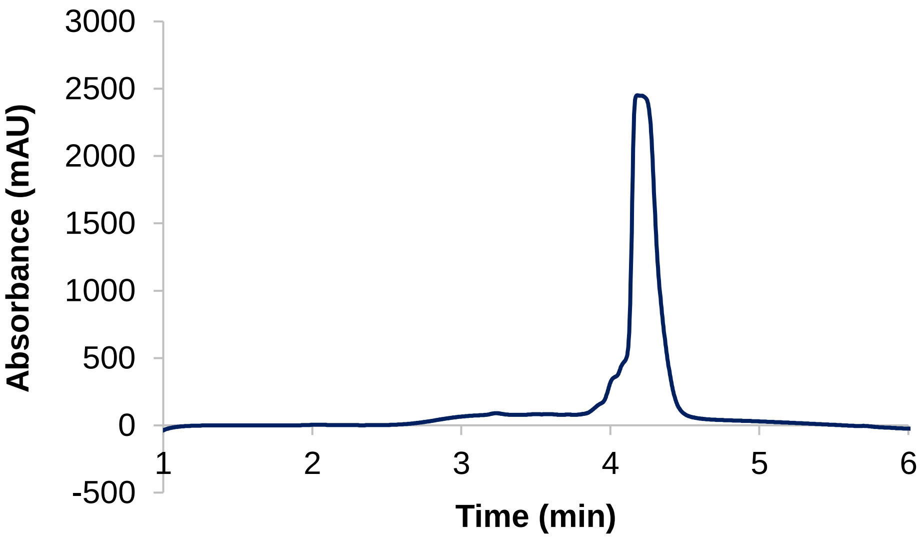

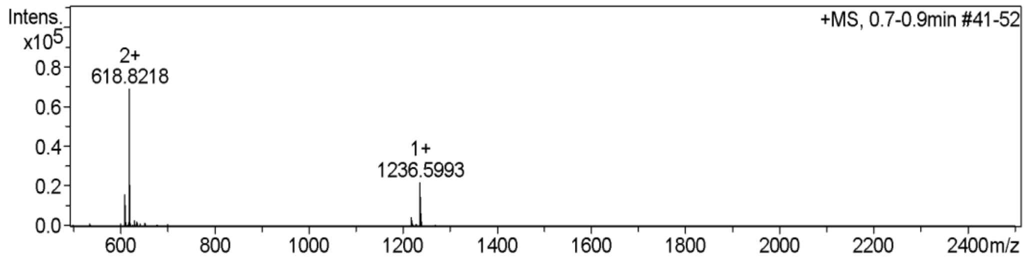

Figure S32. Analytical HPLC trace and ESI MS for product **54**; analytical gradient 2-95% B over 5 min, 210 nm. Calculated mass $[M+H]^+$: 1236.61; observed mass $[M+H]^+$: 1236.60.

Ac-RHL(alG)QVGDSN$_L$CRSI-NH$_2$ (**55**)

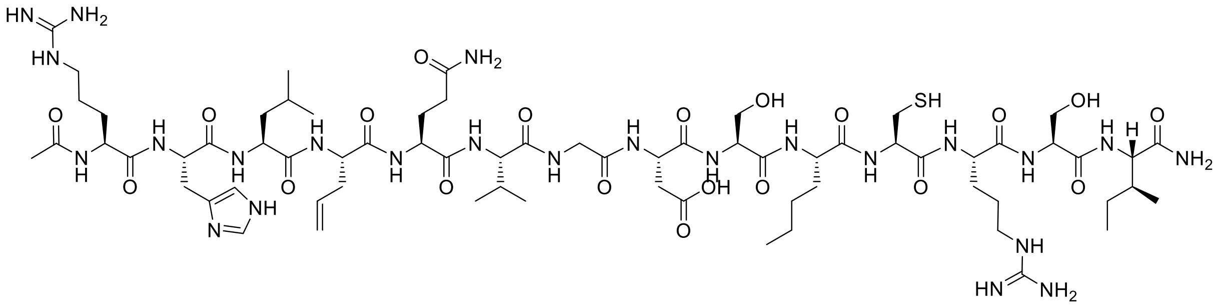

Model peptide **55** was synthesised using the general automated synthesiser procedure with microwave assistance on Rink Amide resin (0.1 mmol). The crude peptide was purified by preparative RP-HPLC (10-70% B over 30 minutes) and lyophilized to produce the desired peptide (yield not calculated).

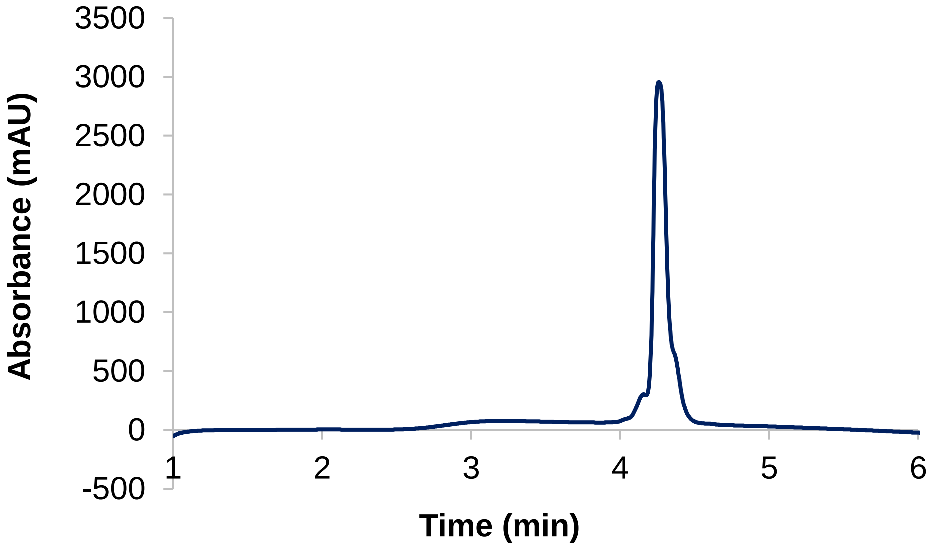

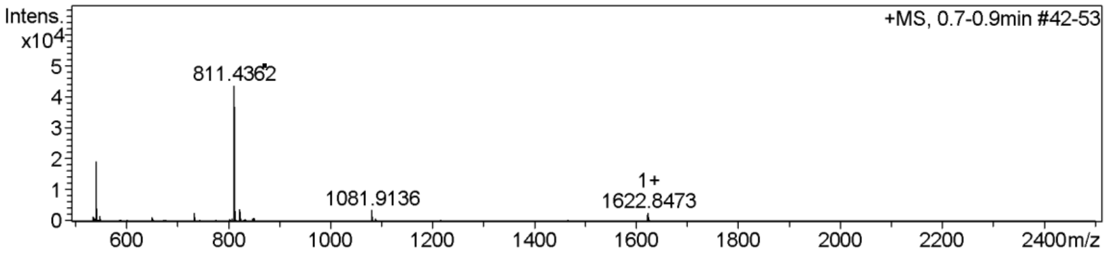

Figure S33. Analytical HPLC trace and ESI MS for product **55**; analytical gradient 2-95% B over 5 min, 210 nm. Calculated mass [M+H]$^+$: 1622.88; observed mass [M+H]$^+$: 1622.85.

Ac-EDIIRNIARHLA(alG)VGDCN$_L$DRSIW-NH$_2$ (**56**)

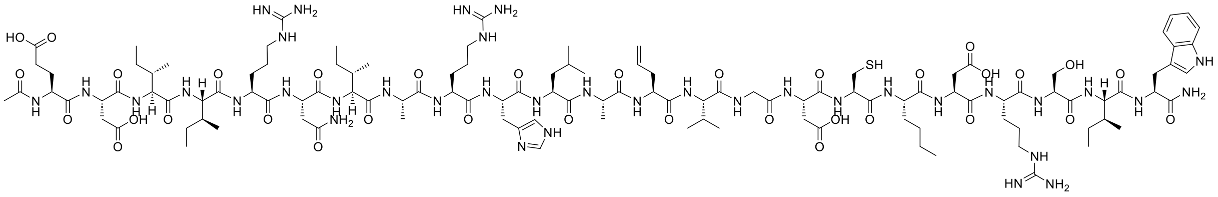

Model peptide **56** was synthesised using the general automated synthesiser procedure with microwave assistance on Rink Amide resin (0.10 mmol). The crude peptide was purified by preparative RP-HPLC (30-100% B over 30 minutes) and lyophilized to produce the desired peptide (11 mg, 4.06 µmol, 4% yield).

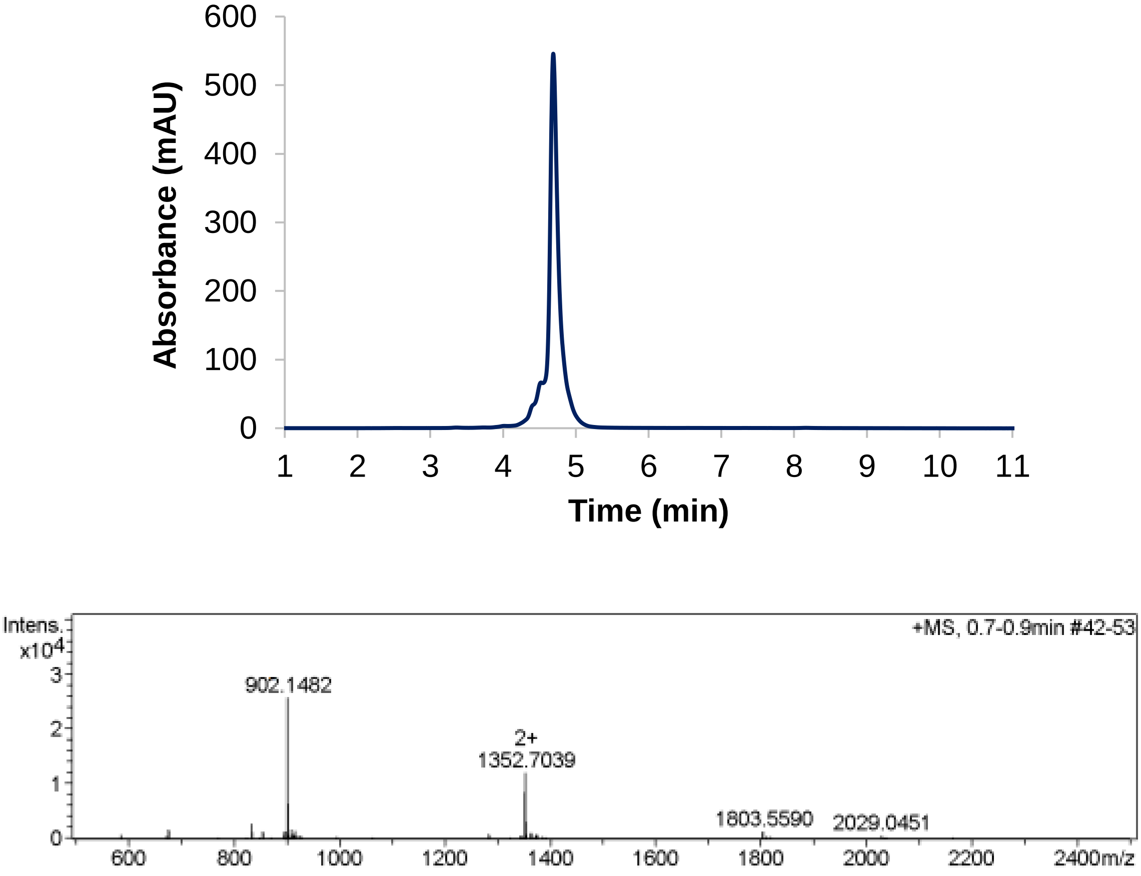

Figure S34. Analytical HPLC trace and ESI MS for product **56**; analytical gradient 30-100% B over 10 min, 280 nm. Calculated mass $[M+2H]^{2+}$: 1352.70; observed mass $[M+2H]^{2+}$: 1352.70.

Peptide Macrocyclisation

Cyclisation of H-S(2-methylallyl)AFAC-NH_2 (**5a/5b**)

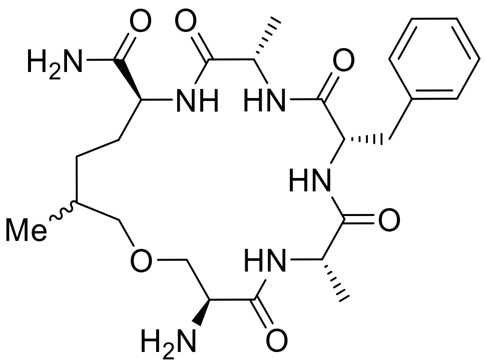

Products **5a** and **5b** was synthesised following the optimised cyclisation protocol using H-S(2-methylallyl)AFAC-NH_2 (**3**, 4 mg, 7.26 µmol). After analysis the remaining solution (7.21 µmol) was purified using semi-preparative HPLC (5-60% B over 30 minutes) and fractions containing the desired product lyophilised to yield the title compound as a fluffy white solid as a mixture of diastereomers - (2.0 mg, 3.88 µmol, 53% yield and 0.9 mg, 1.74 µmol, 24% yield).

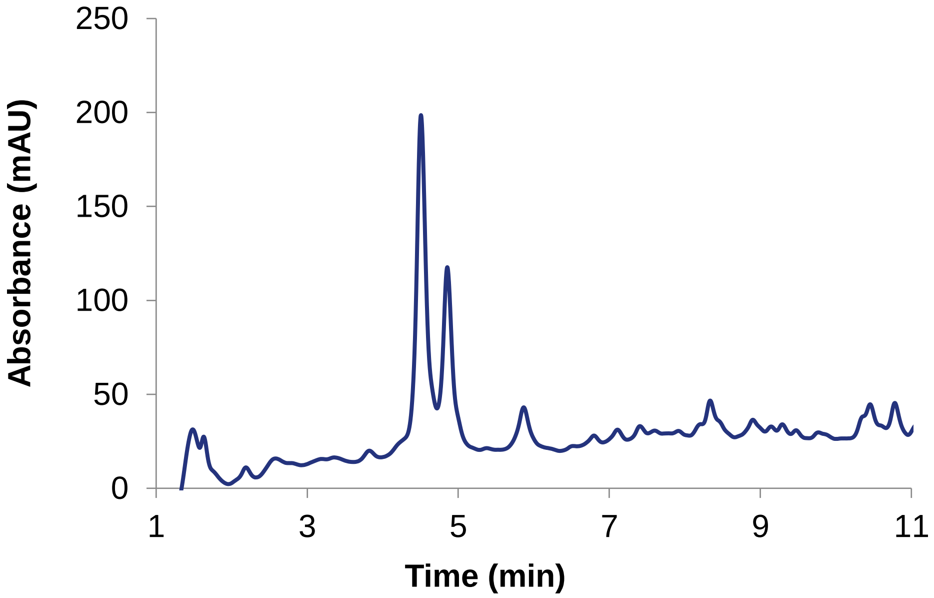

Figure S35. Crude analytical HPLC trace of cyclised H-S(2-methylallyl)AFAC-NH_2 (**5a/5b**); analytical gradient 10-30% B over 10 minutes, 210 nm.

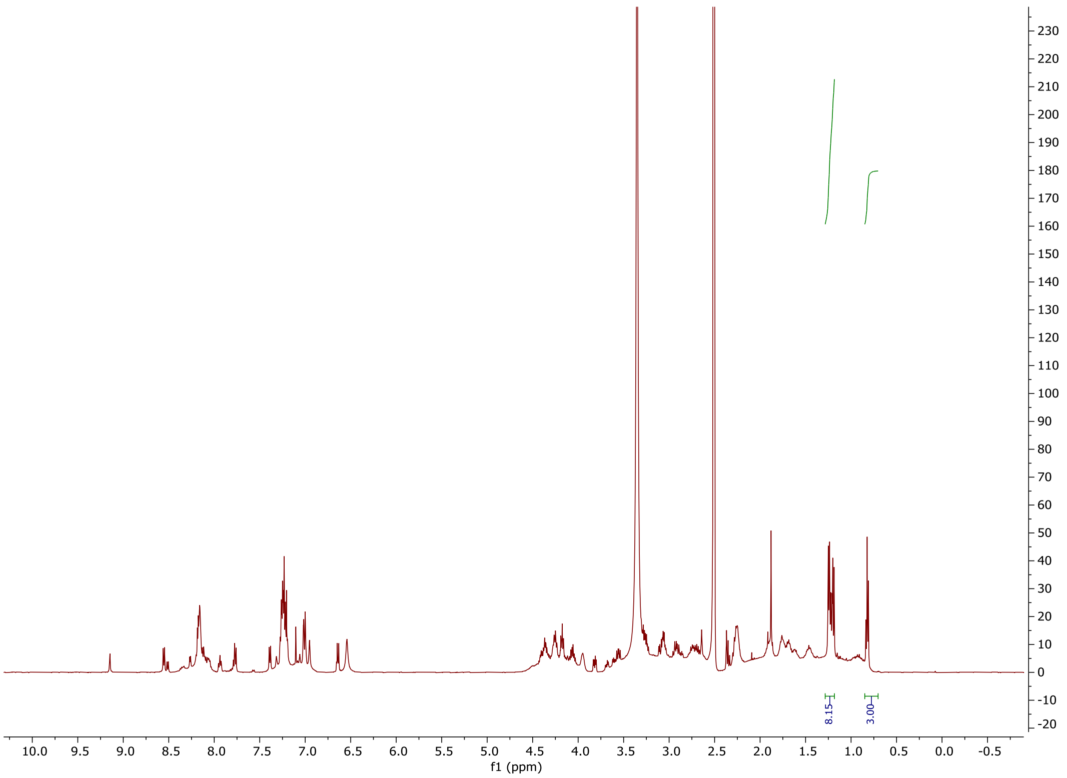

Figure S36. ^{1}H NMR analysis of purified mix of diastereomers **5a/5b**.

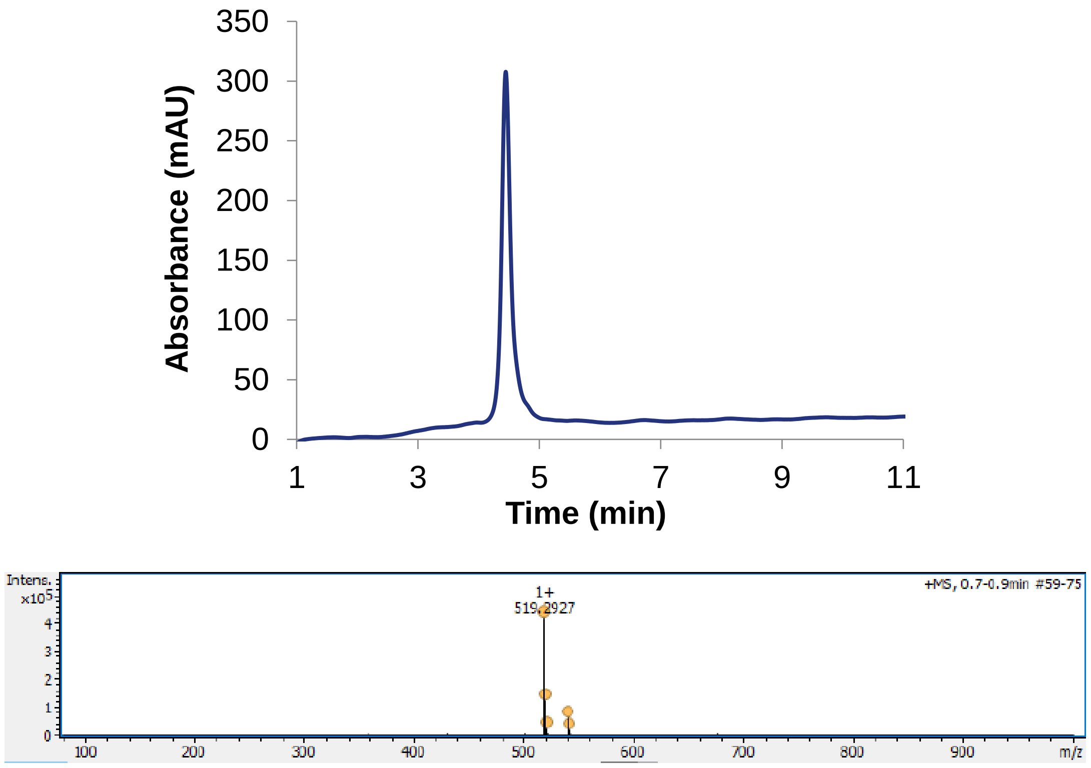

Figure S37. Analytical HPLC trace and ESI MS of purified diastereomer A from the cyclisation of H-S(OAllyl)AFAC-NH_2; analytical gradient 10-30% B over 10 minutes, 210 nm. Calculated mass $[M+H]^+$: 519.29; observed mass $[M+H]^+$: 519.29.

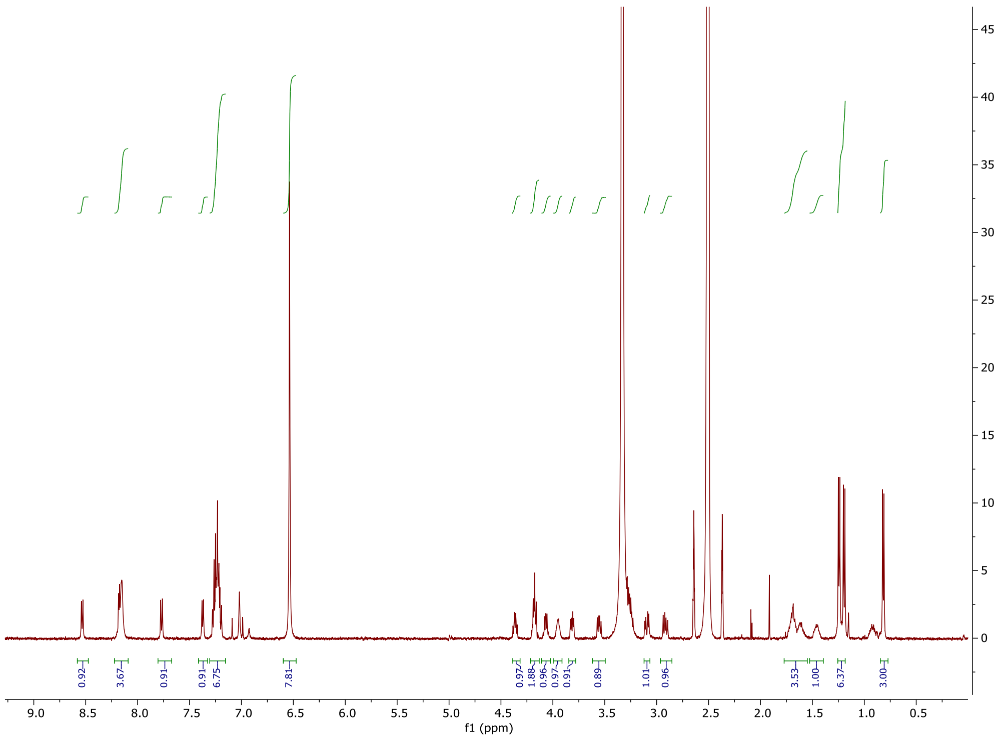

Figure S38. ^{1}H NMR of purified diastereoisomer B.

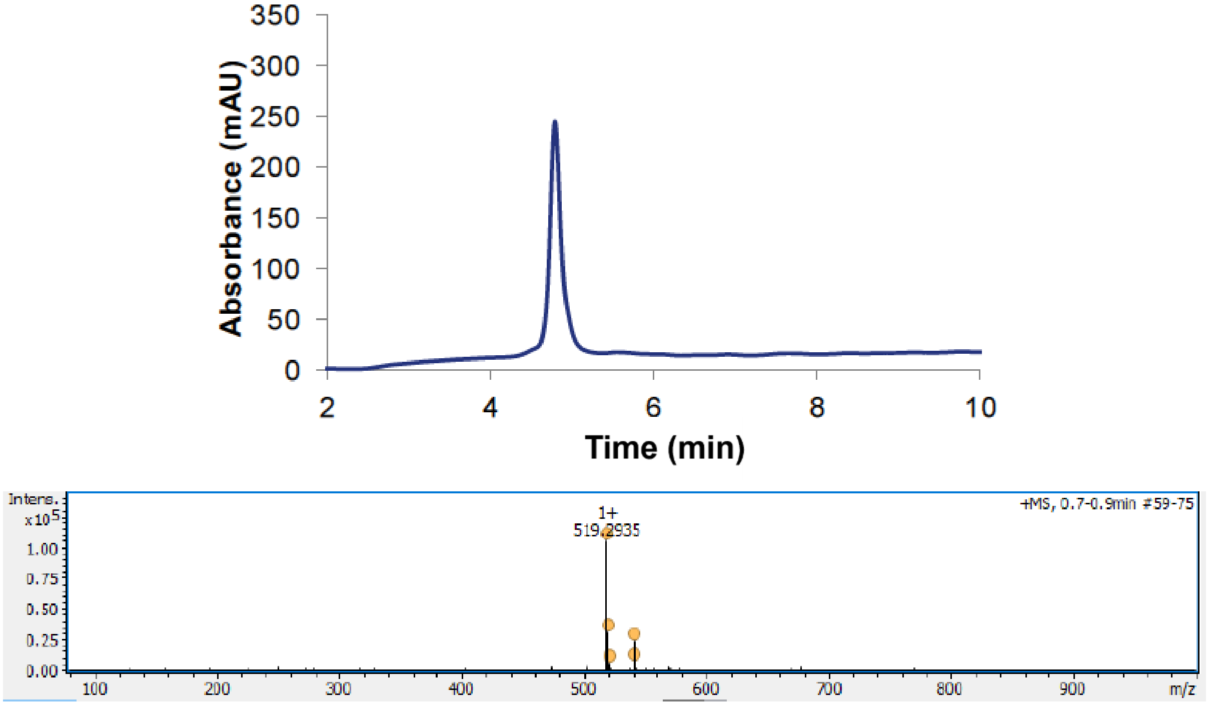

Figure S39. Analytical HPLC trace and ESI MS of purified diastereomer B from the cyclisation of H-S(OAllyl)AFAC-NH_2; analytical gradient 10-30% B over 10 minutes, 210 nm. Calculated mass $[M+H]^+$: 519.29; observed mass $[M+H]^+$: 519.29.

Cyclisation of H-S(OAllyl)AFAC-NH_2 (**6**)

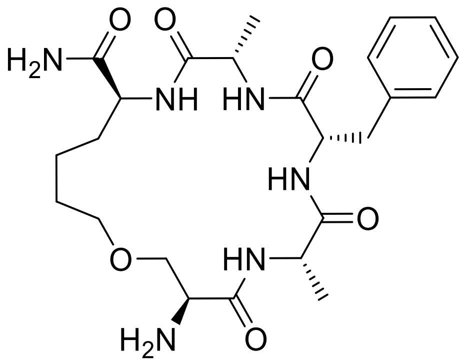

Product **6** was synthesised following the optimised cyclisation protocol using H-S(OAllyl)AFAC-NH_2 (**4**, 4 mg, 7.43 µmol) with 5 mol% Ir catalyst. After analysis the remaining solution (7.35 µmol) was purified using semi-preparative HPLC (5-60% B over 30 minutes) and fractions containing the desired product lyophilised to yield the title compound as a fluffy white solid (2.9 mg, 5.75 µmol, 78% yield).

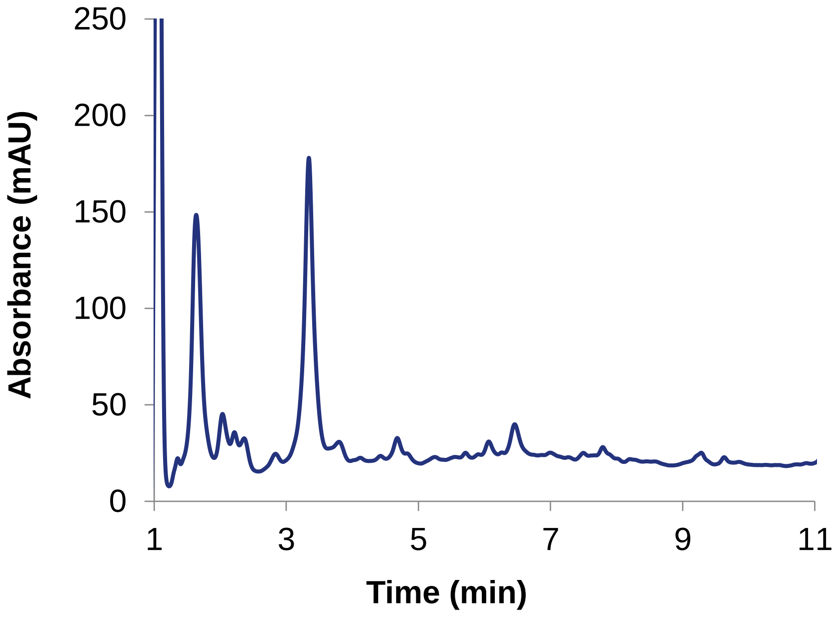

Figure S40. Crude analytical HPLC trace of cyclised H-S(OAllyl)AFAC-NH_2 (**6**); analytical gradient 10-30% B over 10 minutes, 210 nm.

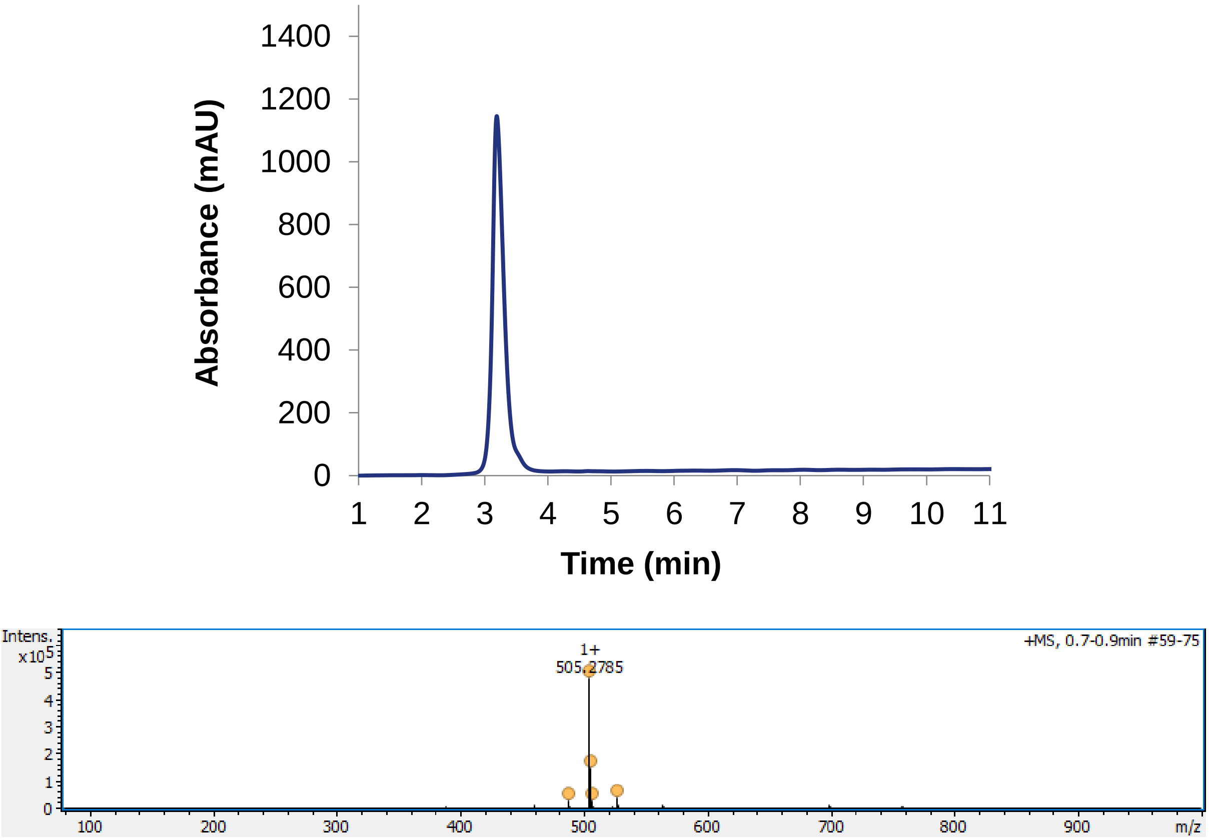

Figure S41. Analytical HPLC trace and ESI MS of cyclised H-S(OAllyl)AFAC-NH_2 (**6**); analytical gradient 2-25% B over 10 minutes, 210 nm. Calculated mass $[M+H]^+$: 505.28; observed mass $[M+H]^+$: 505.28.

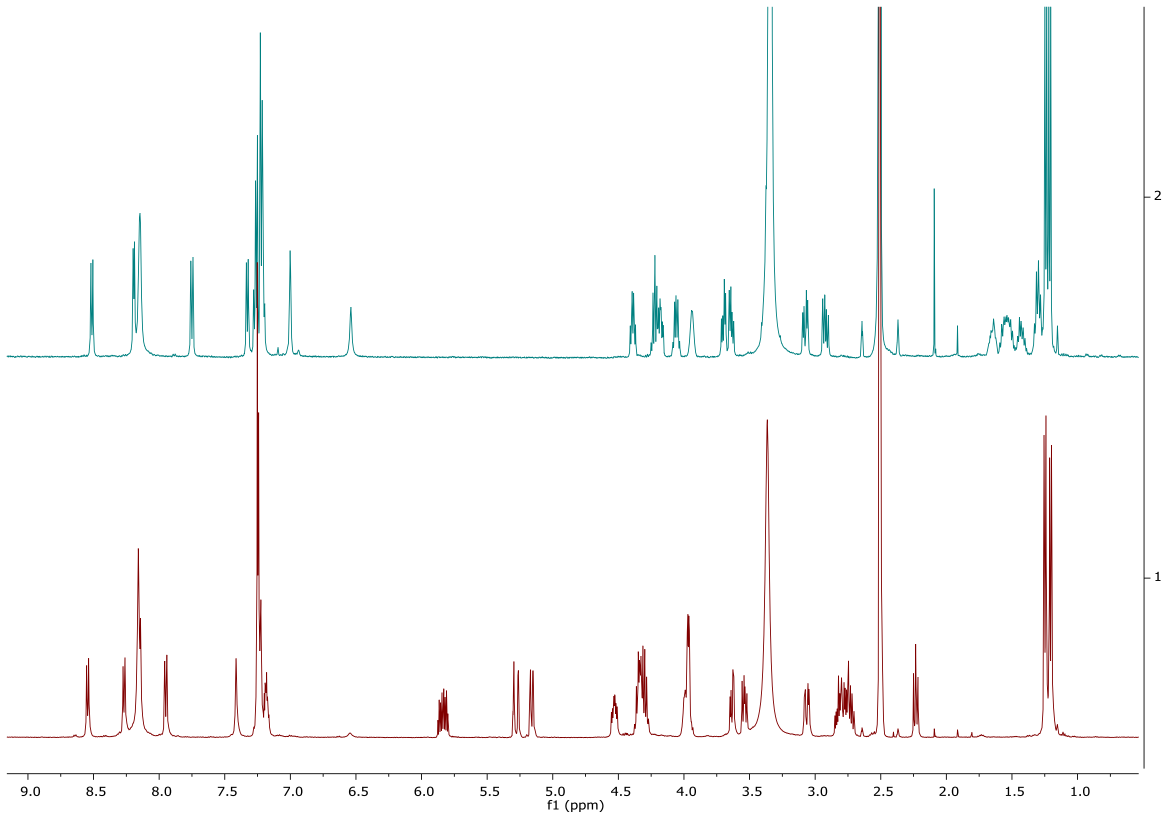

Figure S42. NMR comparison of cyclised H-S(OAllyl)AFAC-NH2 (**6**, top) and the starting peptide **4** (bottom) showing disappearance of allylic protons at 5.71 and 5.15 ppm.

Cyclisation of H-(alG)AFAC-NH2 (**8a**)

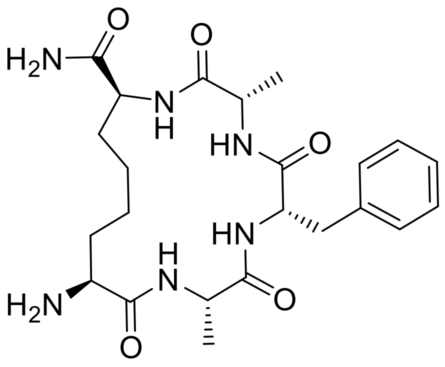

Product **8a** was synthesised following the optimised cyclisation protocol using H-alGAFAC-NH2 (**7a**, 3 mg, 5.92 µmol). After analysis the remaining solution (5.85 µmol) was purified using semi-preparative HPLC (5-60% B over 30 minutes) and fractions containing the desired product lyophilised to yield the title compound as a fluffy white solid (2.2 mg, 4.64 µmol, 79% yield).

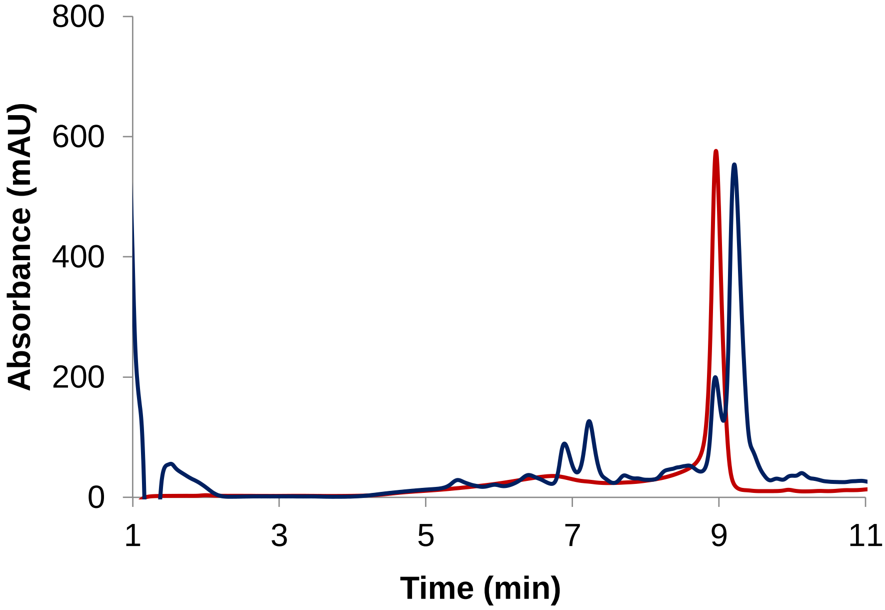

Figure S43. Crude analytical HPLC (blue) trace of cyclised H-(alG)AFAC-NH2 (**8a**); analytical gradient 2-25% B over 10 minutes, 210 nm. Red trace – H-(alG)AFAA-NH2 (**9**).

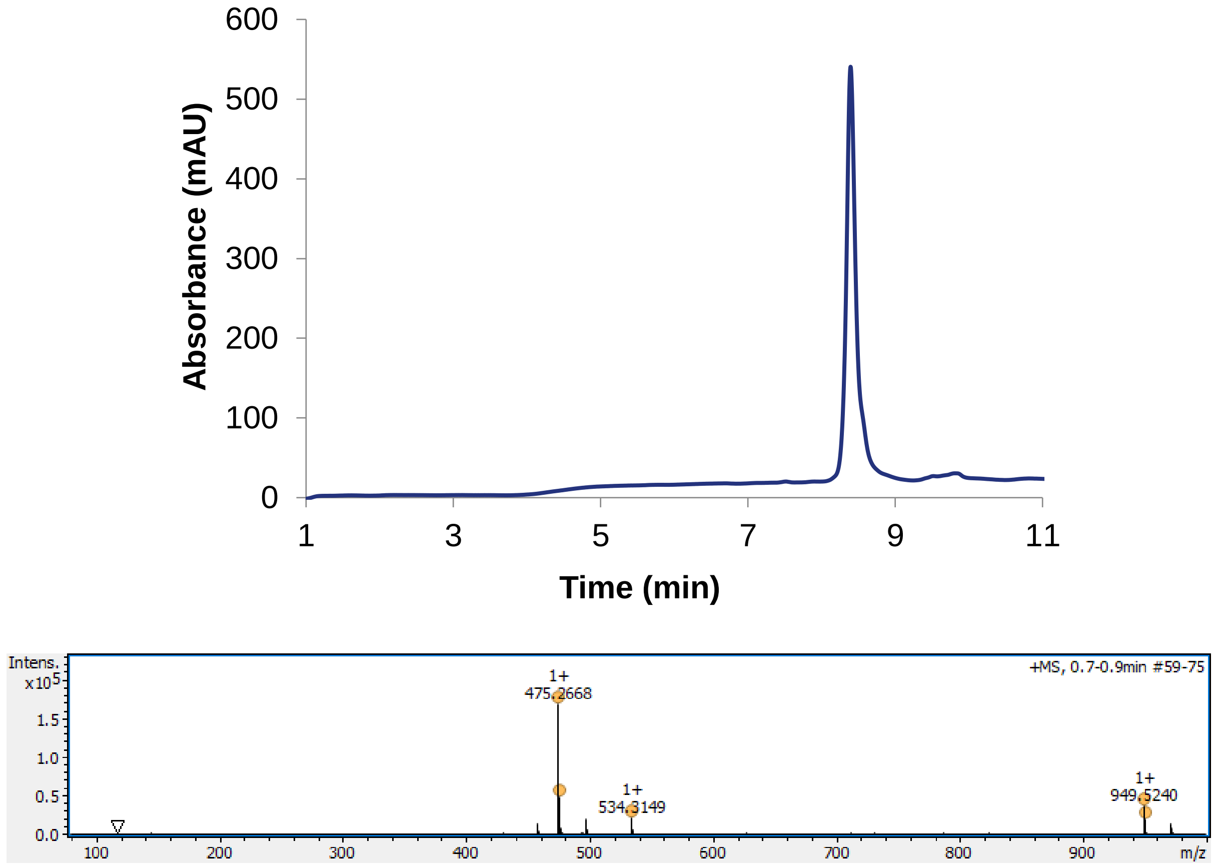

Figure S44. Analytical HPLC trace and ESI MS of cyclised H-(alG)AFAC-NH_2 (**8a**); analytical gradient 2-25% B over 10 minutes, 210 nm. Calculated mass $[M+H]^+$: 475.27; observed mass $[M+H]^+$: 465.27.

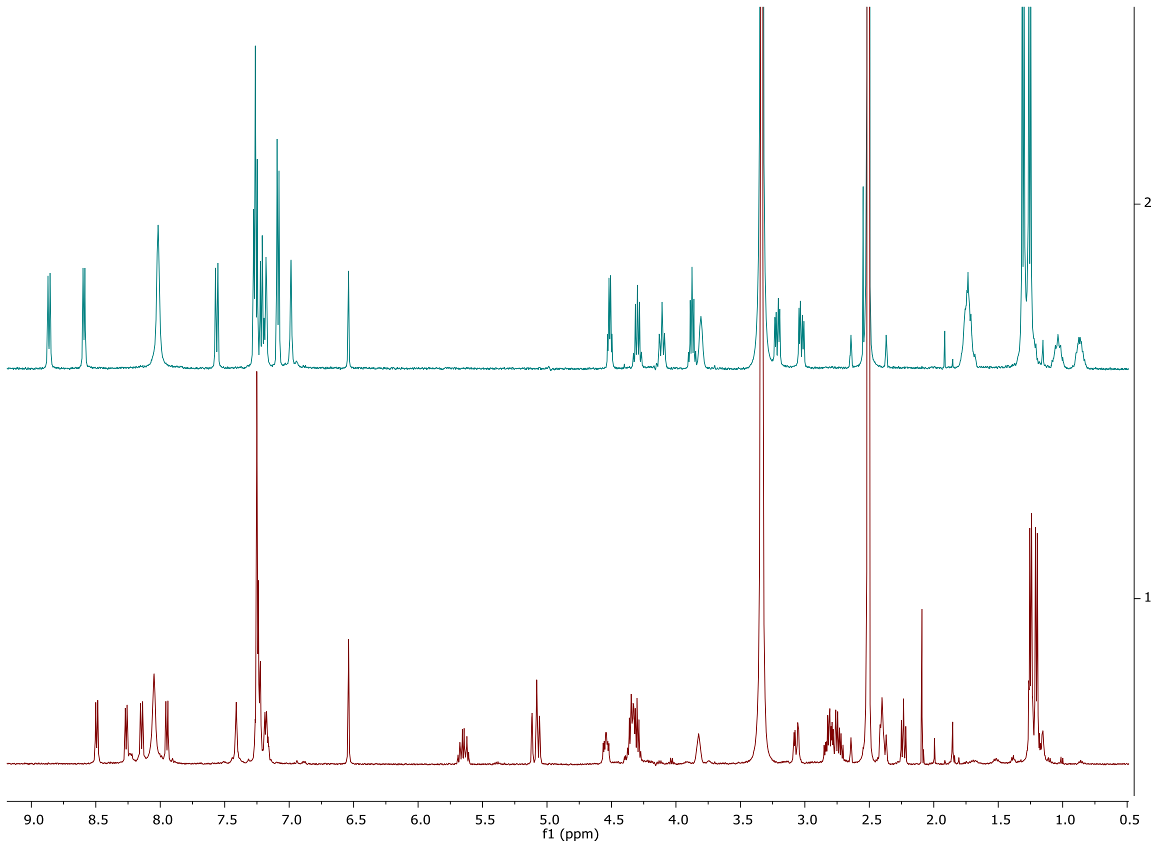

Figure S45. NMR comparison of cyclised H-(alG)AFAC-NH_2 (**8a**, top) and the starting peptide **7a** (bottom), showing disappearance of allylic protons at 5.71 and 5.15 ppm.

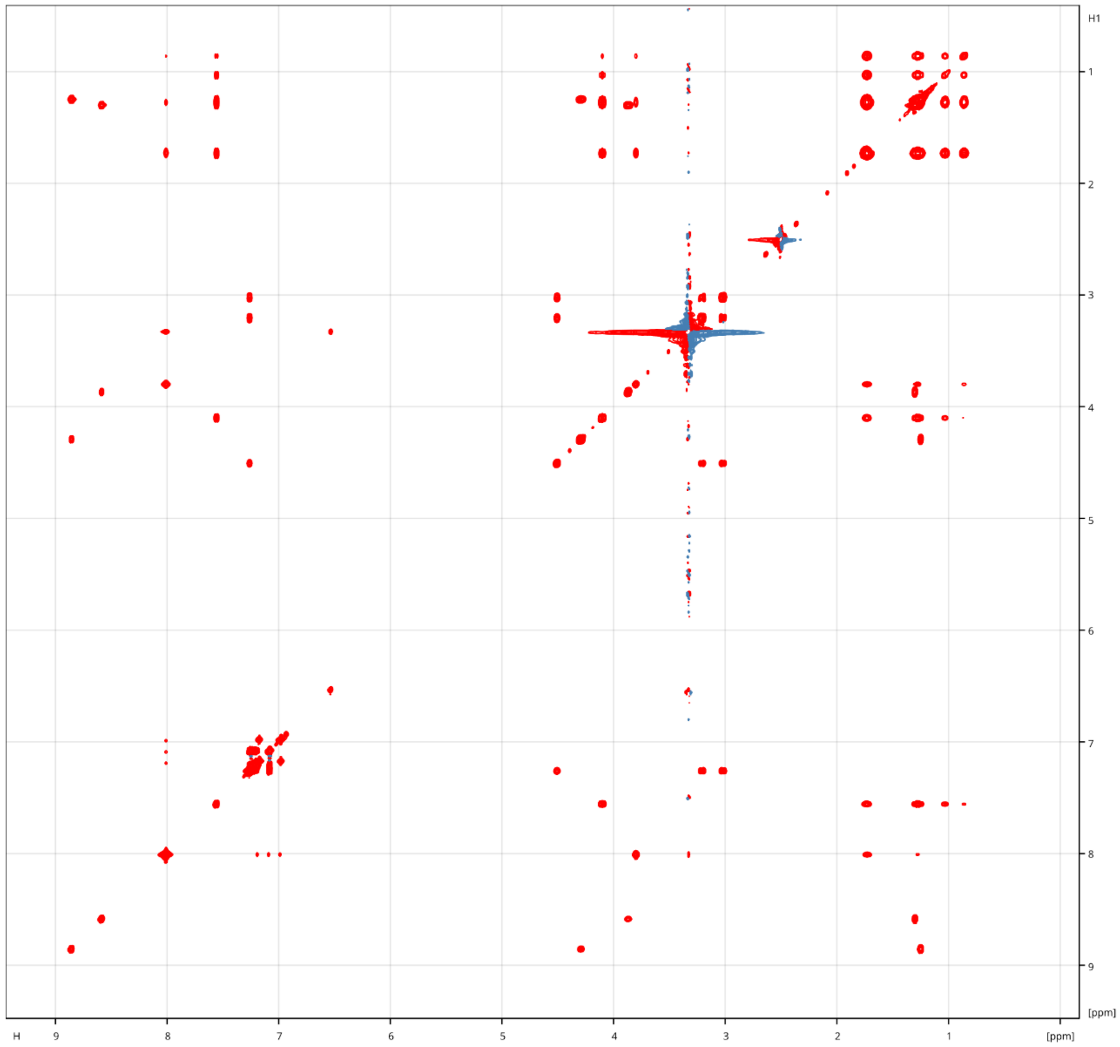

Figure S46. TOCSY NMR spectra of cyclised H-(alG)AFAC-NH_2 (**8a**).

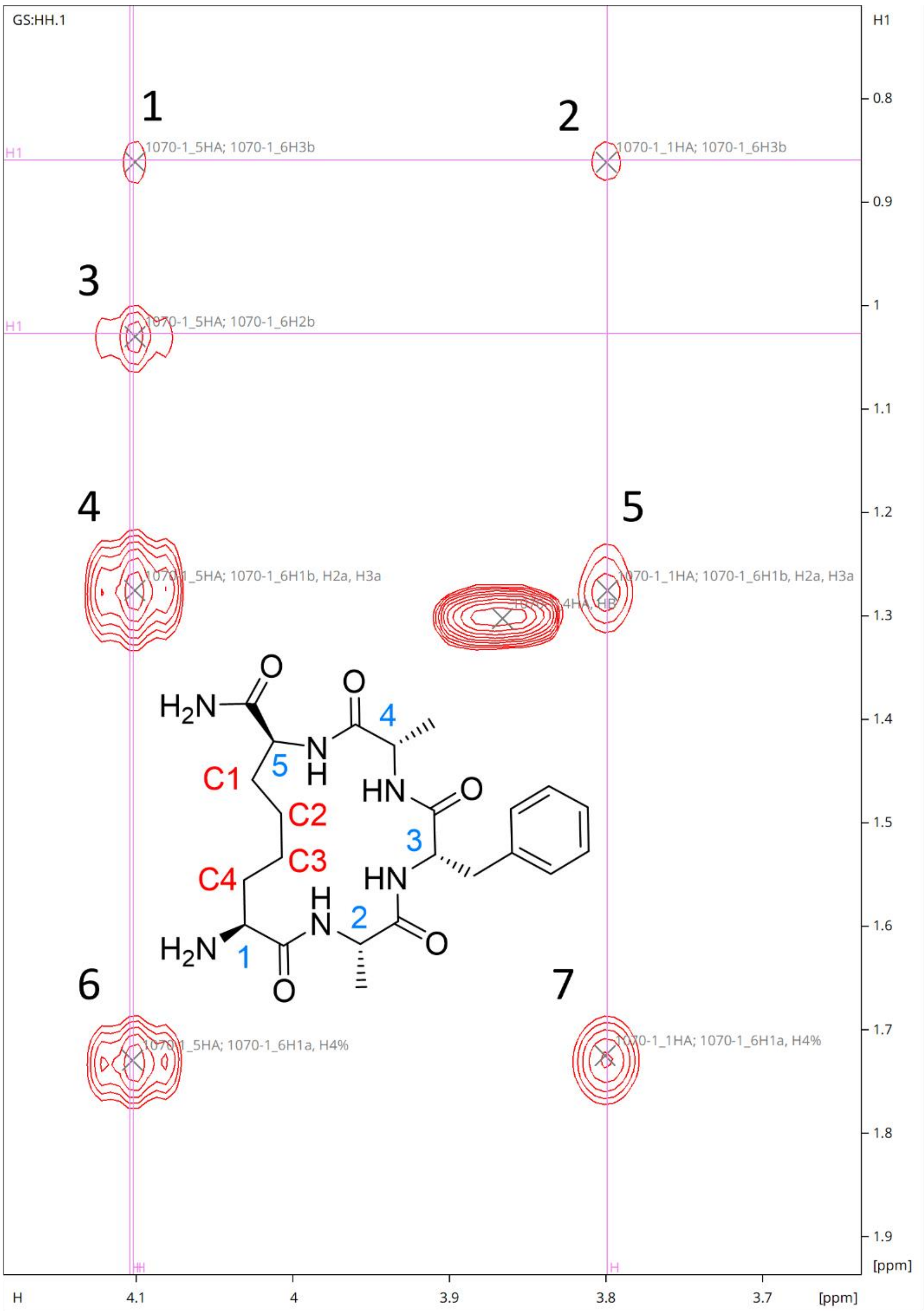

Figure S47. Insert of TOCSY spectra of peptide **8a:** 1. Hα on residue 5 couples to Hβ on linker C3; 2. Hα on residue 1 couples to Hβ on linker C3; 3. Hα on residue 5 couples to Hβ on linker C2; 4. Hα on residue 5 couples to Hβ on linker C1 and Hα on C2 and C3; 5. Hα on residue 1 couples to Hβ on linker C1 and Hα on C2 and C3; 6. Hα on residue 5 couples to Hα on linker C1 and Hα/Hβ C4; 7. Hα on residue 1 couples to Hα on linker C1 and Hα/Hβ C4.

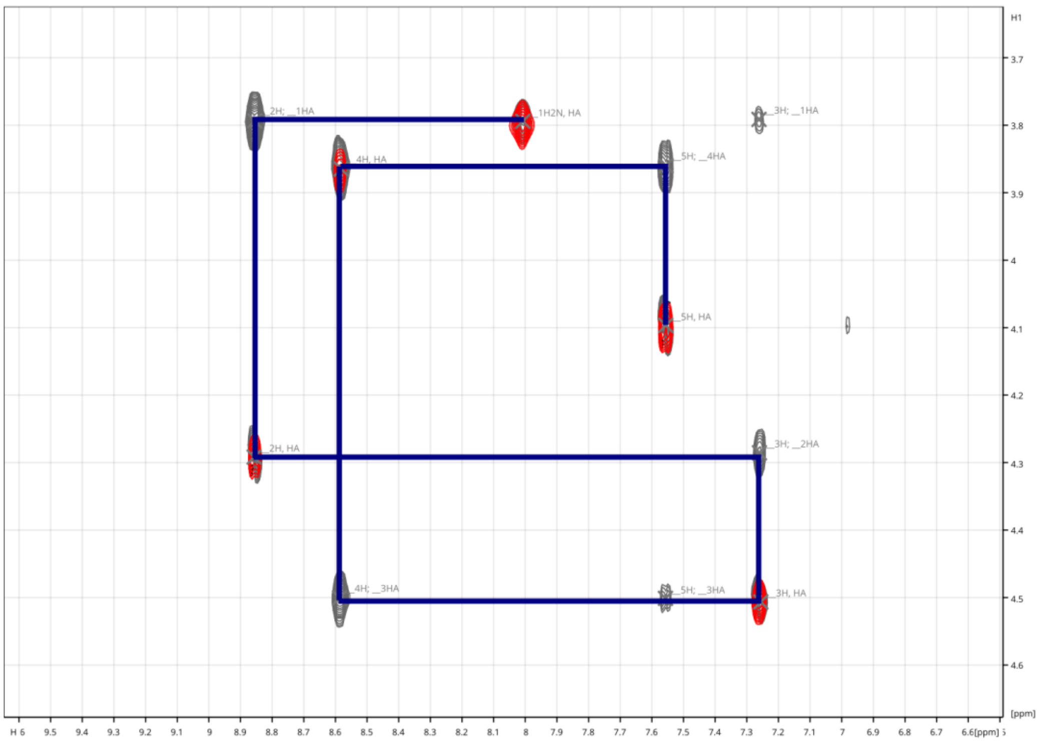

Figure S48. Overlay of NOESY (Grey) and TOCSY (Red) spectra of **8a**. Sequential assignment of the amide backbone is possible from the intra-residue HN-H-alpha (TOCSY and NOESY peak) to the sequential inter-residue H-alpha(i) to HN(i+1) (NOESY only).

Comparing LED light sources

Peptide **7a** was cyclised under the standard conditions using both the PhotoRedOx box and LED light strips for reaction rate comparison. The reaction was shown to reach completion within 45 min using the light strips and 15 min using the PhotoRedOx Box.

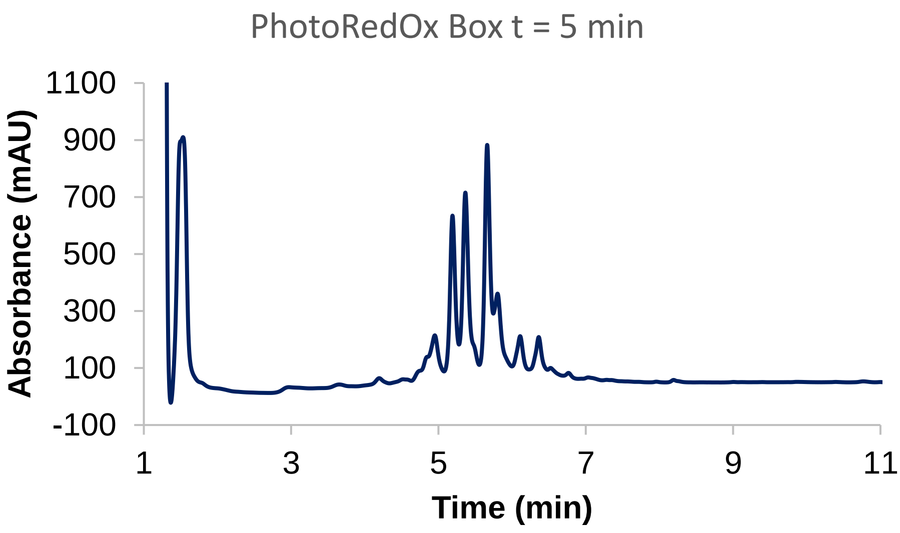

Figure S49. Crude analytical HPLC trace of cyclised H-(alG)AFAC-NH_2 (**8a**) using the PhotoRedOx Box after 5 min; analytical gradient 5-60% B over 10 minutes, 210 nm.

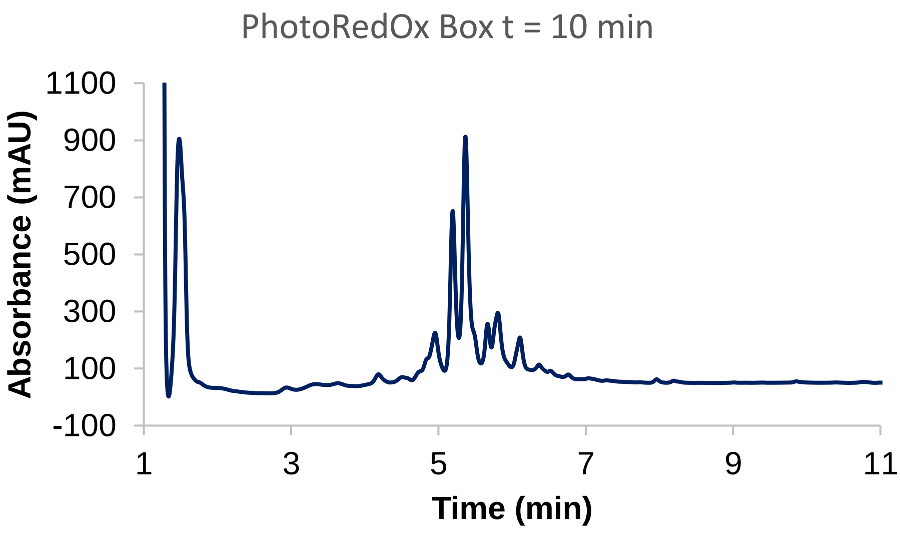

Figure S50. Crude analytical HPLC trace of cyclised H-(alG)AFAC-NH_2 (**8a**) using the PhotoRedOx Box after 10 min; analytical gradient 5-60% B over 10 minutes, 210 nm.

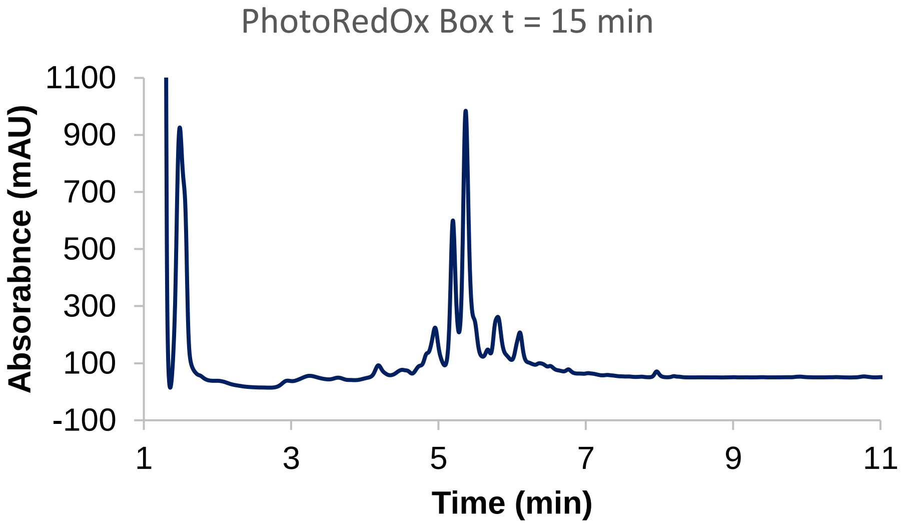

Figure S51. Crude analytical HPLC trace of cyclised H-(alG)AFAC-NH_2 (**8a**) using the PhotoRedOx Box after 15 min; analytical gradient 5-60% B over 10 minutes, 210 nm.

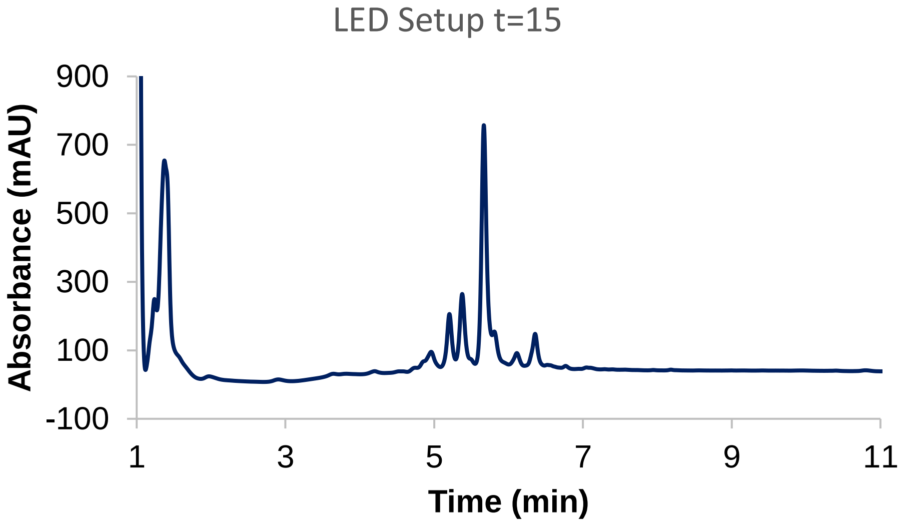

Figure S52. Crude analytical HPLC trace of cyclised H-(alG)AFAC-NH_2 (**8a**) using LED strips after 15 min; analytical gradient 5-60% B over 10 minutes, 210 nm.

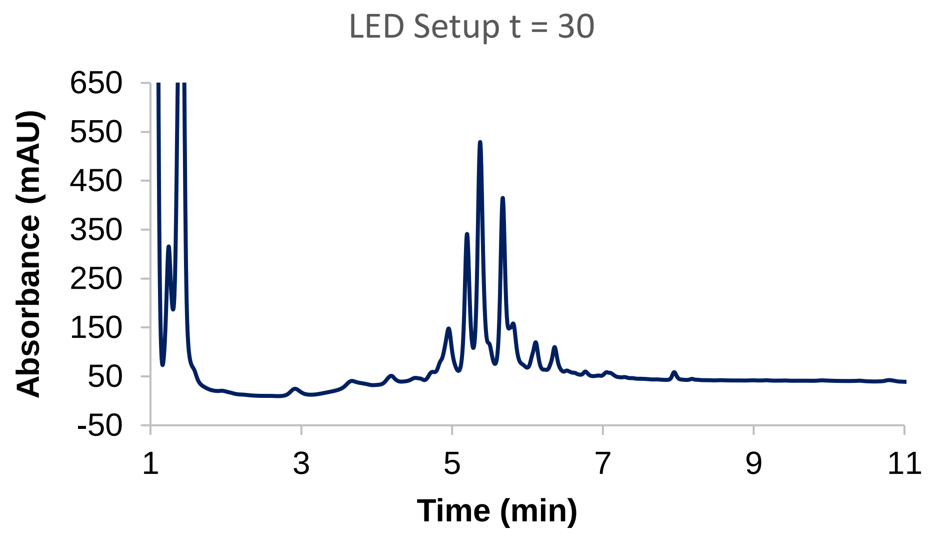

Figure S53. Crude analytical HPLC trace of cyclised H-(alG)AFAC-NH_2 (**8a**) using LED strips after 30 min; analytical gradient 5-60% B over 10 minutes, 210 nm.

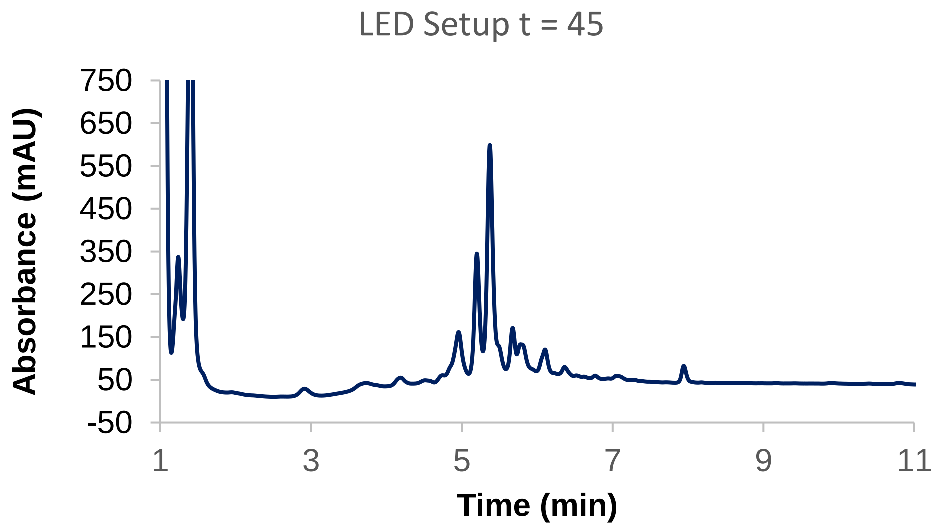

Figure S54. Crude analytical HPLC trace of cyclised H-(alG)AFAC-NH_2 (**8a**) using LED strips after 45 min; analytical gradient 5-60% B over 10 minutes, 210 nm.

Peptide cyclisation in alternative buffers

Product **8a** was synthesised following the optimised cyclisation protocol with variations in buffer for 1 hr using H-(alG)AFAC-NH_2 (**7a**, 1 mg, 1.97 µmol). The reaction was monitored *via* analytical HPLC (2-25% B over 10 min, 210 nm).

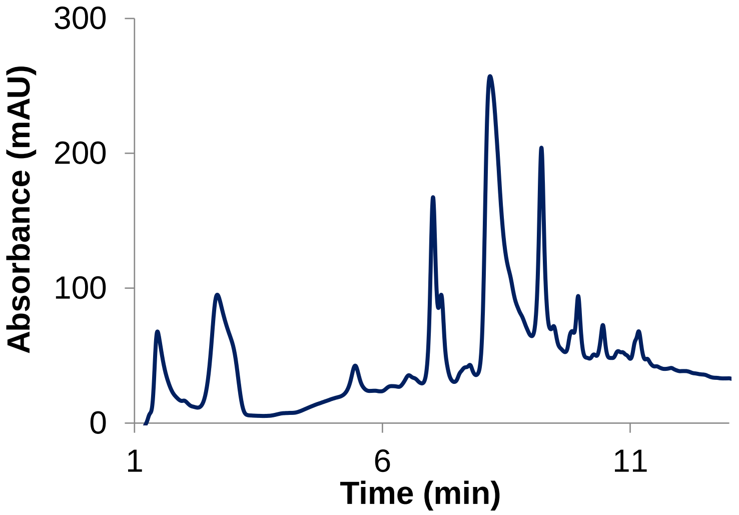

Figure S55. Crude analytical HPLC trace of Ac-(alG)AFAC-NH_2 (**7a**) cyclisation in PBS using the PhotoRedOx Box after 60 min; analytical gradient 2-25% B over 10 minutes, 210 nm.

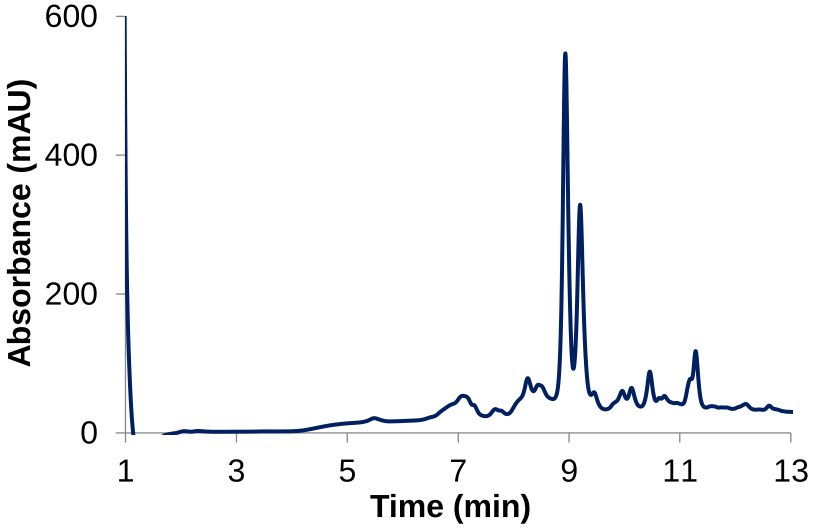

Figure S56. Crude analytical HPLC trace of Ac-(alG)AFAC-NH_2 (**7a**) cyclisation in HEPES using the PhotoRedOx Box after 60 min; analytical gradient 2-25% B over 10 minutes, 210 nm.

Phosphine additive study

3,3′,3″-Phosphanetriyltris(benzenesulfonic acid) trisodium salt (TPPTS)

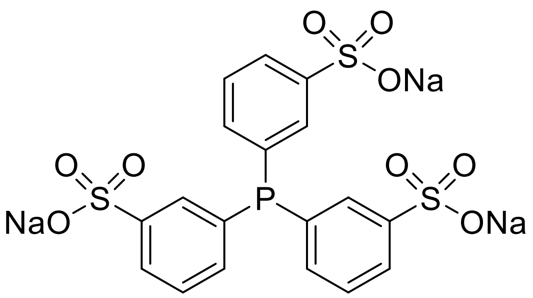

Product **8a** was synthesised following the optimised cyclisation protocol using TPPTS for 1 hr using H-(alG)AFAC-NH_2 (**7a**, 1 mg, 1.97 µmol). The reaction was monitored *via* analytical HPLC (2-25% B over 10 min, 210 nm). Crude not purified.

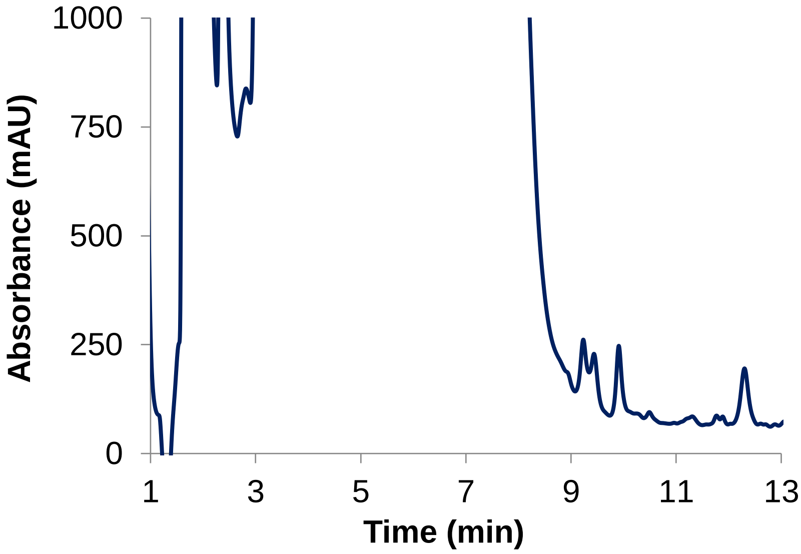

Figure S57. Crude analytical HPLC trace of Ac-(alG)AFAC-NH_2 (**7a**) cyclisation using TPPTS as the phosphine in the PhotoRedOx Box after 60 min; analytical gradient 2-25% B over 10 minutes, 210 nm.

1,3,5-Triaza-7-phosphaadamantane (PTA)

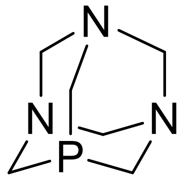

Product **8a** was synthesised following the optimised cyclisation protocol using PTA for 1 hr using H-(alG)AFAC-NH_2 (**7a**, 1 mg, 1.97 µmol). The reaction was monitored *via* analytical HPLC (2-25% B over 10 min, 210 nm). After analysis the remaining solution was purified using semi-preparative HPLC (2-25% B over 30 minutes) and fractions containing the desired product lyophilised to yield the title compound as a fluffy white solid (1.0 mg, 2.11 µmol, 56% yield).

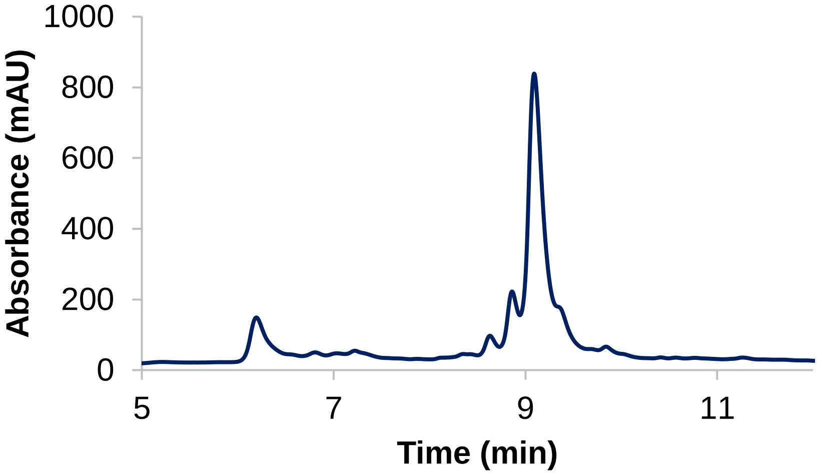

Figure S58. Crude analytical HPLC trace of Ac-(alG)AFAC-NH_2 (**7a**) cyclisation using PTA as the phosphine in the PhotoRedOx Box after 60 min; analytical gradient 2-25% B over 10 minutes, 210 nm.

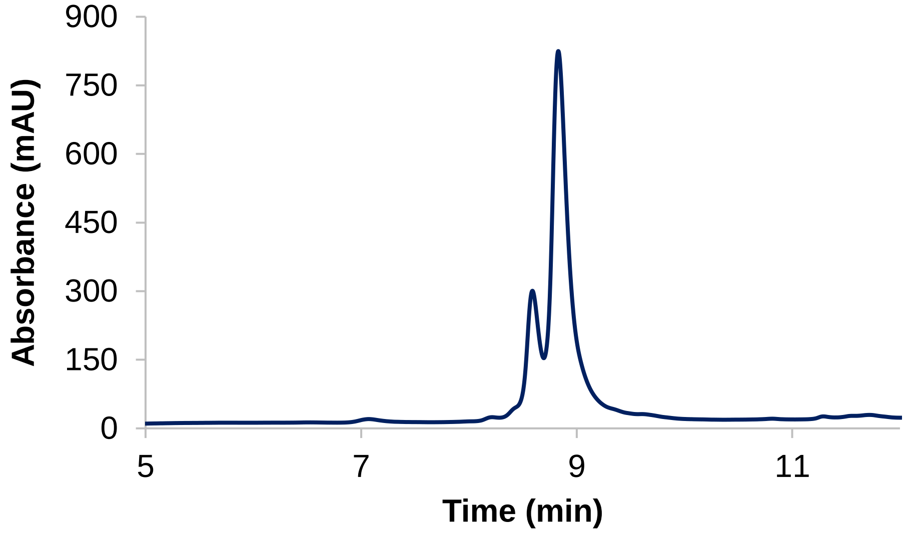

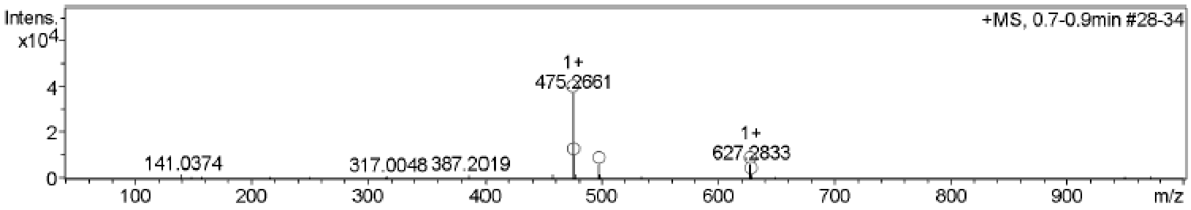

Figure S59. Analytical HPLC trace and ESI MS of linear desulfurised by-product (along with unidentified by-product) isolated at 20.8 min; analytical gradient 2-25% B over 10 minutes, 210 nm.

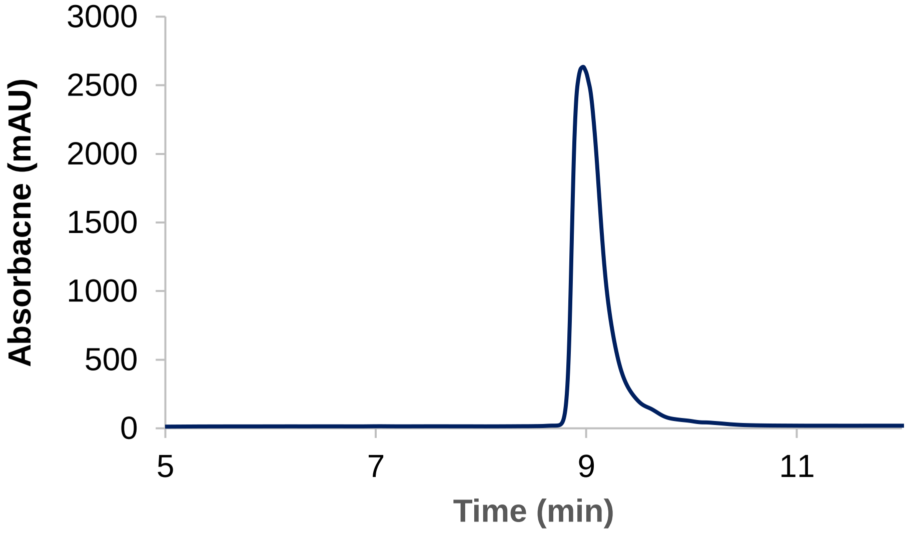

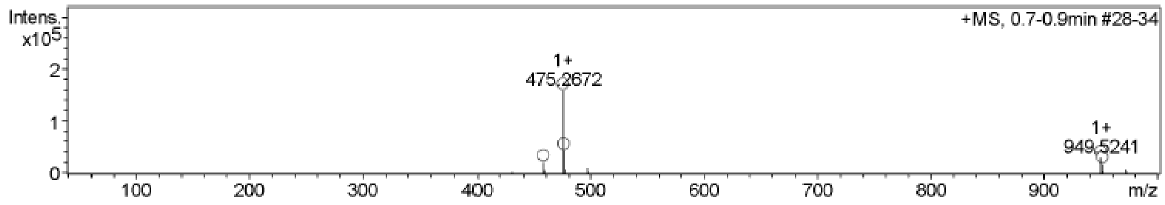

Figure S60. Analytical HPLC trace and ESI MS of desired product (**8a**) isolated at 21.5 min; analytical gradient 2-25% B over 10 minutes, 210 nm.

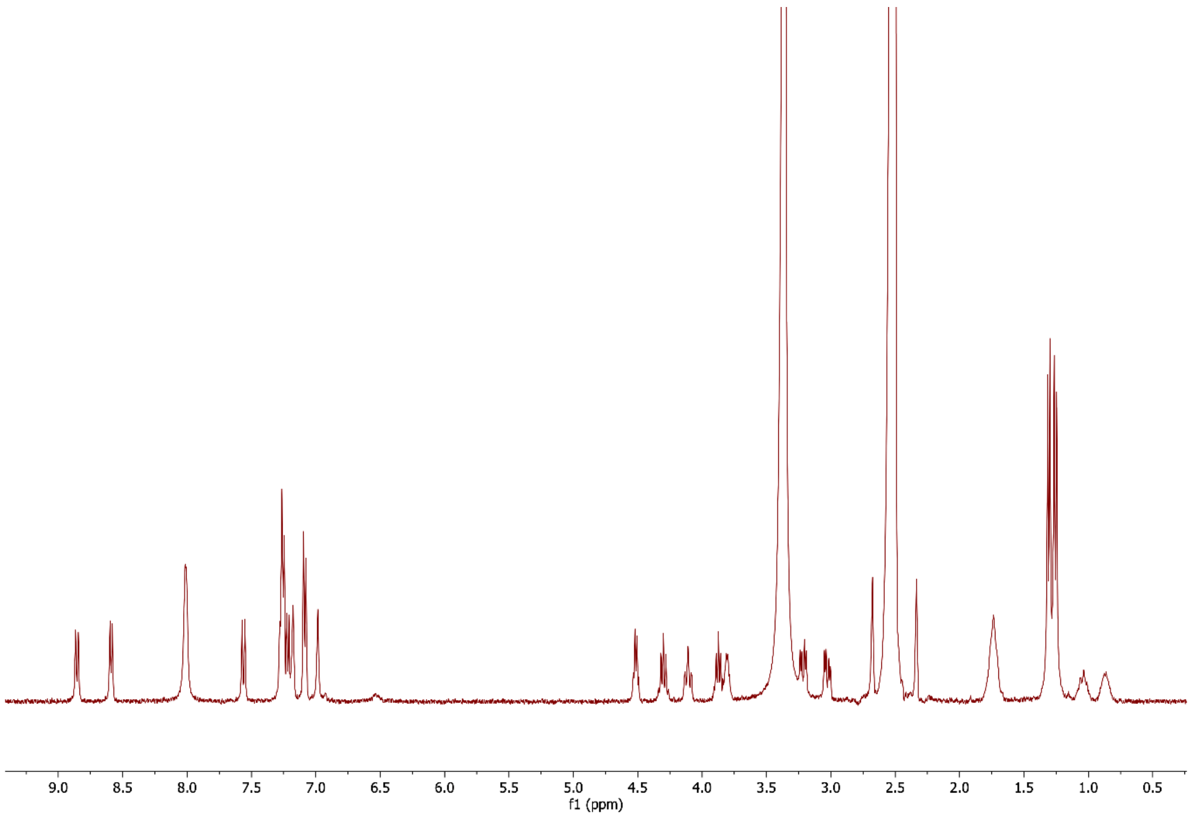

Figure S61. ^{1}H NMR of product **8a** isolated from the PTA reaction showing disappearance of allylic protons at 5.71 and 5.15 ppm.

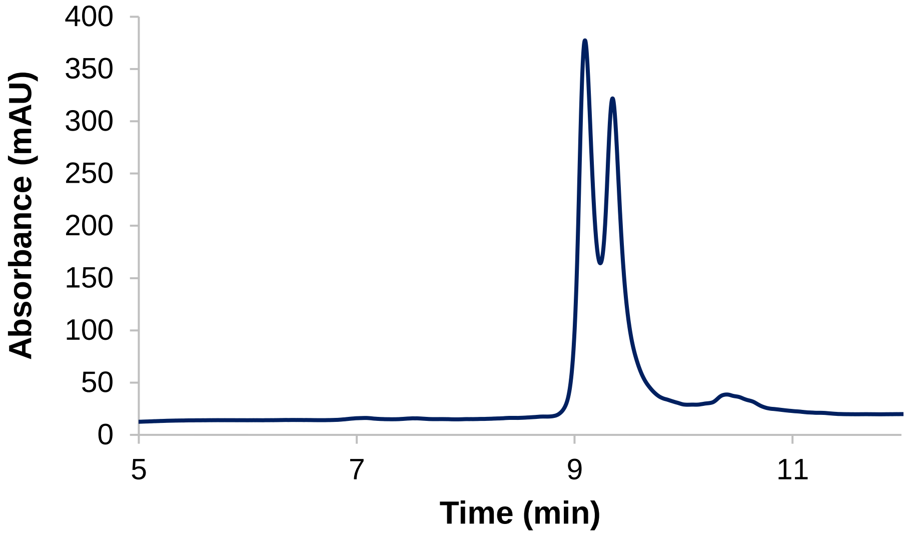

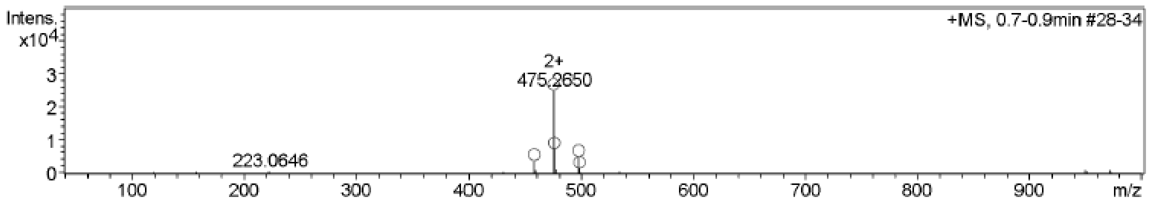

Figure S62. Analytical HPLC trace and ESI MS of potential dimer by-product 21.8 min; analytical gradient 2-25% B over 10 minutes, 210 nm.

Alternative desulfurisation conditions

Product **8a** was synthesised following the conditions from Li *et. al*, *Chem*, **2022**, *8*, 2542–2557[5] using H-(alG)AFAC-NH2 (**7a**). Peptide **7a** (2 mg, 3.94 µmol, 1.0 eq.) was dissolved to a concentration of 1 mM in buffer (6 M Gnd.HCl, 0.2 M sodium acetate, pH = 5). TCEP (789 μL, 0.5 M, 100 eq.) was added and the solution vortexed. $NaBEt_4$ (789 μL, 0.5 M, 100 eq.) was added and the solution was stirred gently for 1 min. The reaction was monitored *via* crude analytical HPLC (2-25% B over 10 min, 210).

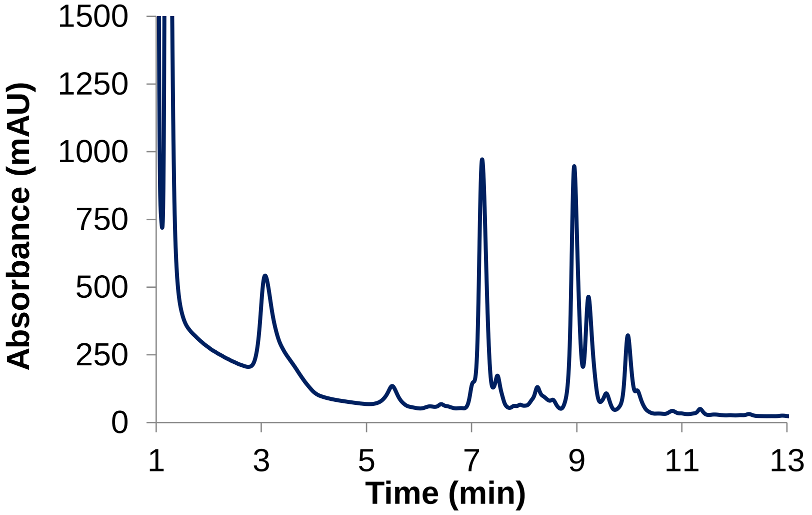

Figure S63. Crude analytical HPLC trace of Ac-(alG)AFAC-NH_2 (**7a**) cyclisation using $NaBEt_4$ after 10 min; analytical gradient 2-25% B over 10 minutes, 210 nm.

Product **8a** was synthesised following the conditions from Guo *et al.* (*Org. Lett.*, **2016**, *18*, 1166–1169)[6] using H-(alG)AFAC-NH2 (**7a**, 2 mg, 3.94 µmol). Peptide **7a** (2 mg, 3.94 µmol, 1.0 eq.) was dissolved to a concentration of 0.5 mM in degassed buffer:MeCN solution (6 M Gnd.HCl, 0.1 M Na_2HPO_4, 9:1). TPPTS or TCEP (395 μL, 0.5 M, 50 eq.) and $Ru(bpy)_3Cl_2$ (197.4 μL, 1 mM, 0.05 eq.) were added and the pH was adjusted to 7.4 with 2 M NaOH (4 μL). The remaining volume of degassed buffer:MeCN solution (1378 μL, 9:1) was added and the solution was further degassed with stirring under a stream of N_2 for 15 min. The solution was irradiated using photochemistry apparatus 2 with either blue or white light. The reaction was monitored *via* crude analytical HPLC (2-25% B over 10 min, 210 nm).

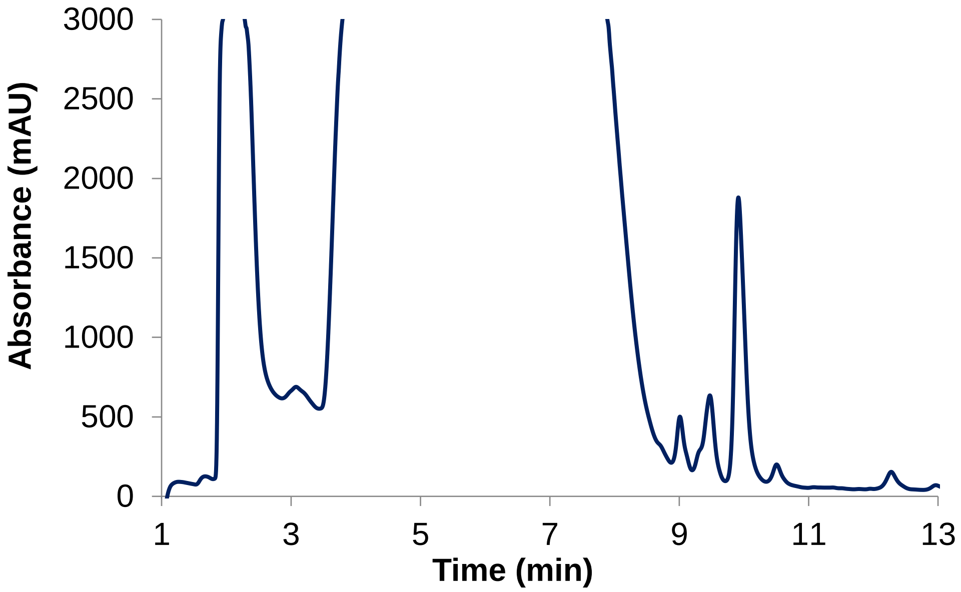

Figure S64. Crude analytical HPLC trace of Ac-(alG)AFAC-NH_2 (**7a**) cyclisation using $Ru(bpy)_3Cl_2$ as a photocatalyst and TPPTS in the PhotoRedOx Box under blue light irradiation no change after 300 min; analytical gradient 2-25% B over 10 minutes, 210 nm. No change after 300 min.

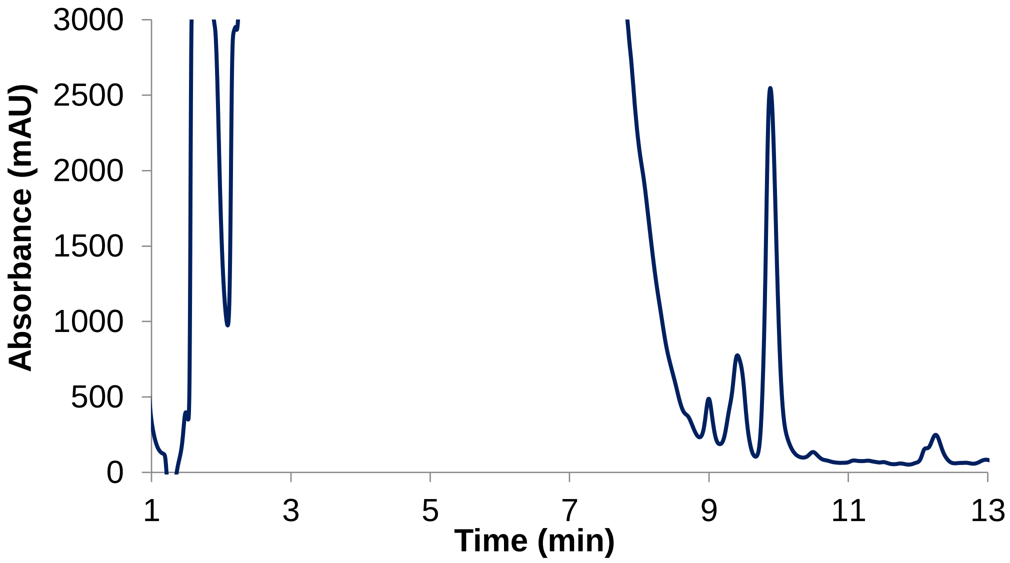

Figure S65. Crude analytical HPLC trace of Ac-(alG)AFAC-NH_2 (**7a**) cyclisation using $Ru(bpy)_3Cl_2$ as a photocatalyst and TPPTS in the PhotoRedOx Box under white light irradiation no change after 300 min; analytical gradient 2-25% B over 10 minutes, 210 nm. No change after 300 min.

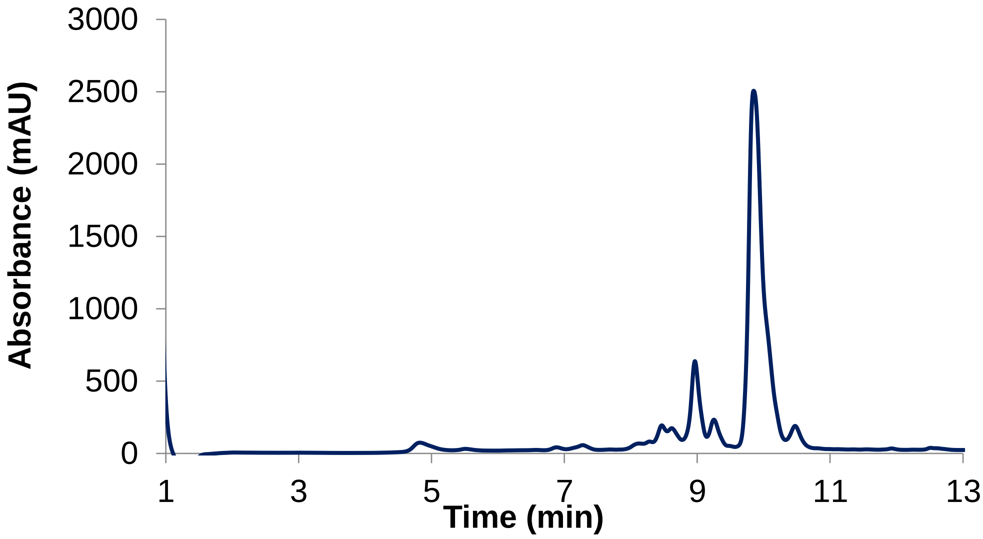

Figure S66. Crude analytical HPLC trace of Ac-(alG)AFAC-NH_2 (**7a**) cyclisation using $Ru(bpy)_3Cl_2$ as a photocatalyst and TCEP in the PhotoRedOx Box under blue light irradiation no change after 300 min; analytical gradient 2-25% B over 10 minutes, 210 nm. No change after 300 min.

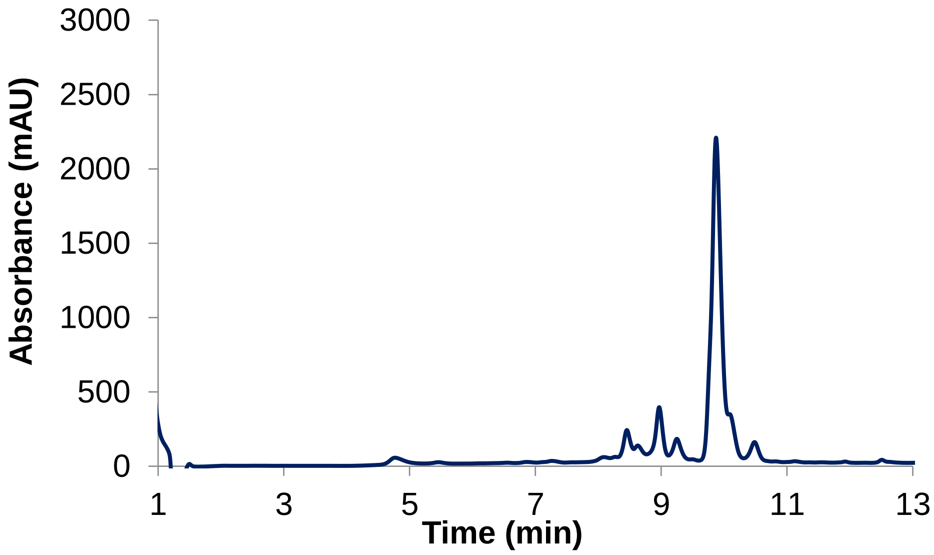

Figure S67. Crude analytical HPLC trace of Ac-(alG)AFAC-NH_2 (**7a**) cyclisation using $Ru(bpy)_3Cl_2$ as a photocatalyst and TCEP in the PhotoRedOx Box under white light irradiation no change after 300 min; analytical gradient 2-25% B over 10 minutes, 210 nm. No change after 300 min.

Cyclisation of H-(alG)AFAC-OH (**8b**)

Product (**8b**) was synthesised following the optimised cyclisation protocol using H-(alG)AFAC-OH (**7b**, 2.0 mg, 3.94 µmol). The solution was purified using semi-preparative HPLC (5-60% B over 30 minutes) however, due to the similar retention times of the linear desulfurised by-product and the cyclised product, no pure material could be isolated. The linear desulfurised by-product:cyclised product ratio appears to be close to 1:1.

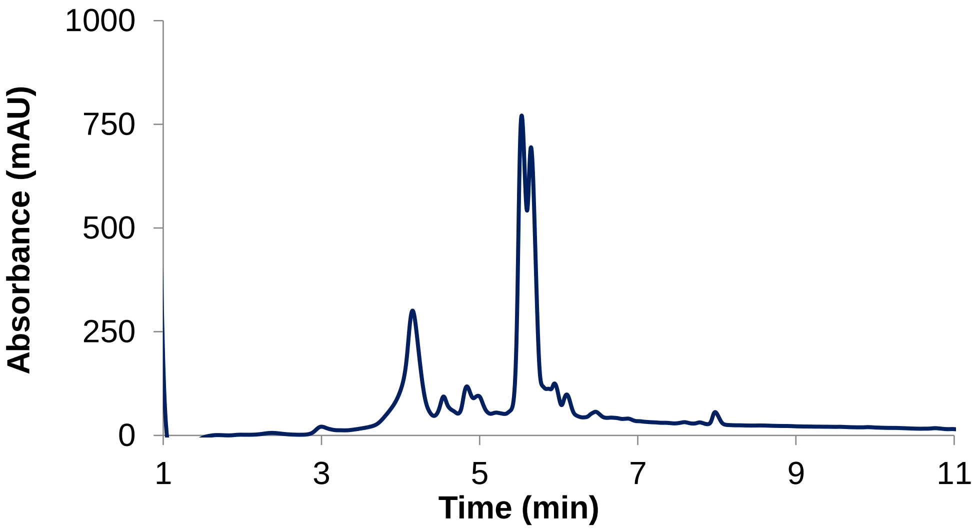

Figure S68. Crude analytical HPLC trace of H-(alG)AFAC-OH cyclisation (**8b**); analytical gradient 5-60% B over 10 minutes, 210 nm.

Cyclisation of H-(pG)AFAC-NH_2 (**11**)

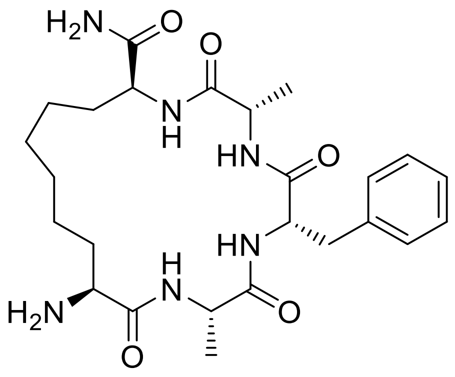

Product **11** was synthesised following the optimised cyclisation protocol using H-(pG)AFAC-NH2 (**10**, 2 mg, 3.47 µmol). The solution was purified using semi-preparative HPLC (5-60% B over 30 minutes) and fractions containing the desired product lyophilised to yield the title compound as a fluffy white solid (0.6 mg, 1.19 µmol, 32% yield).

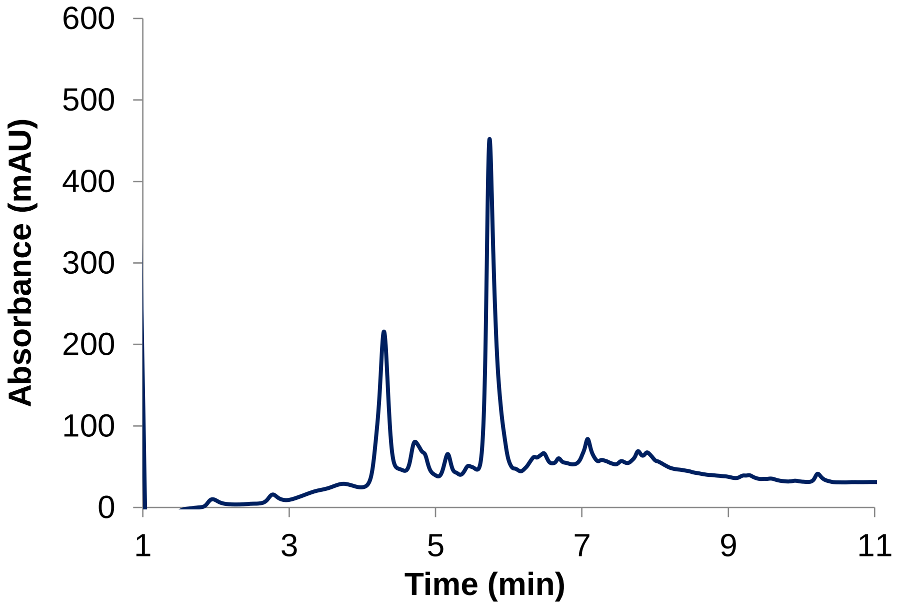

Figure S69. Crude analytical HPLC trace of H-(pG)AFAC-NH_2 cyclisation (**11**); analytical gradient 5-40% B over 10 minutes, 210 nm.

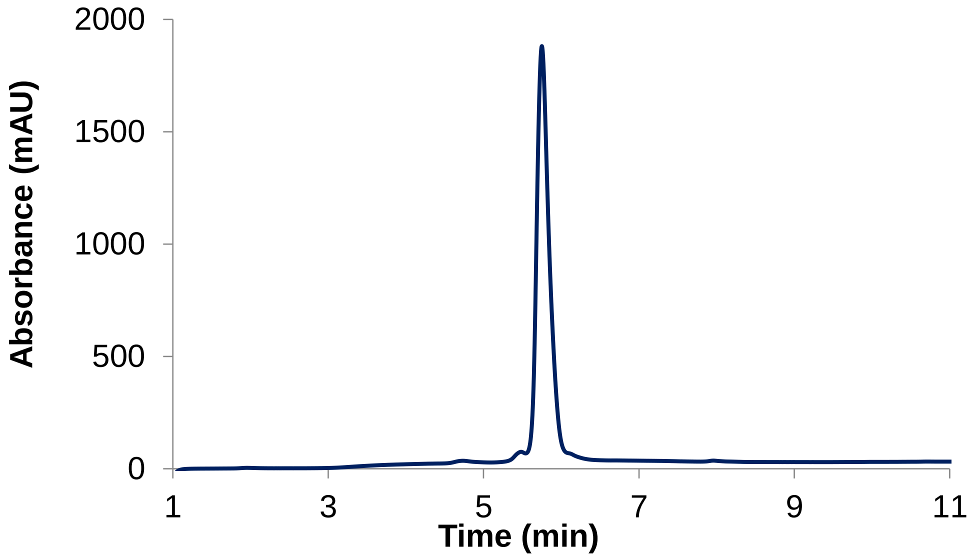

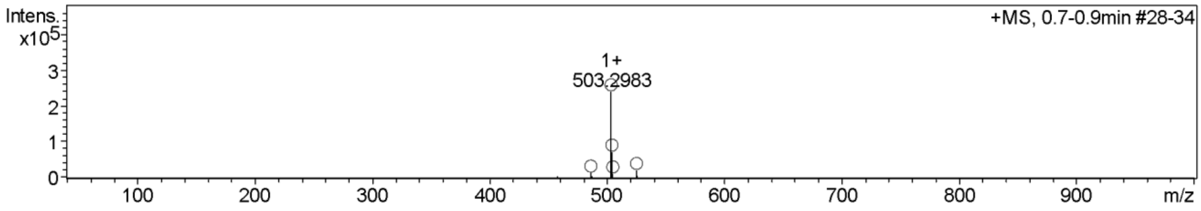

Figure S70. Analytical HPLC trace and ESI MS of purified cyclisation of H-(pG)AFAC-NH_2 (**11**); analytical gradient 5-40% B over 10 minutes, 280 nm. Calculated mass $[M+H]^+$: 502.29; observed mass $[M+H]^+$: 502.30.

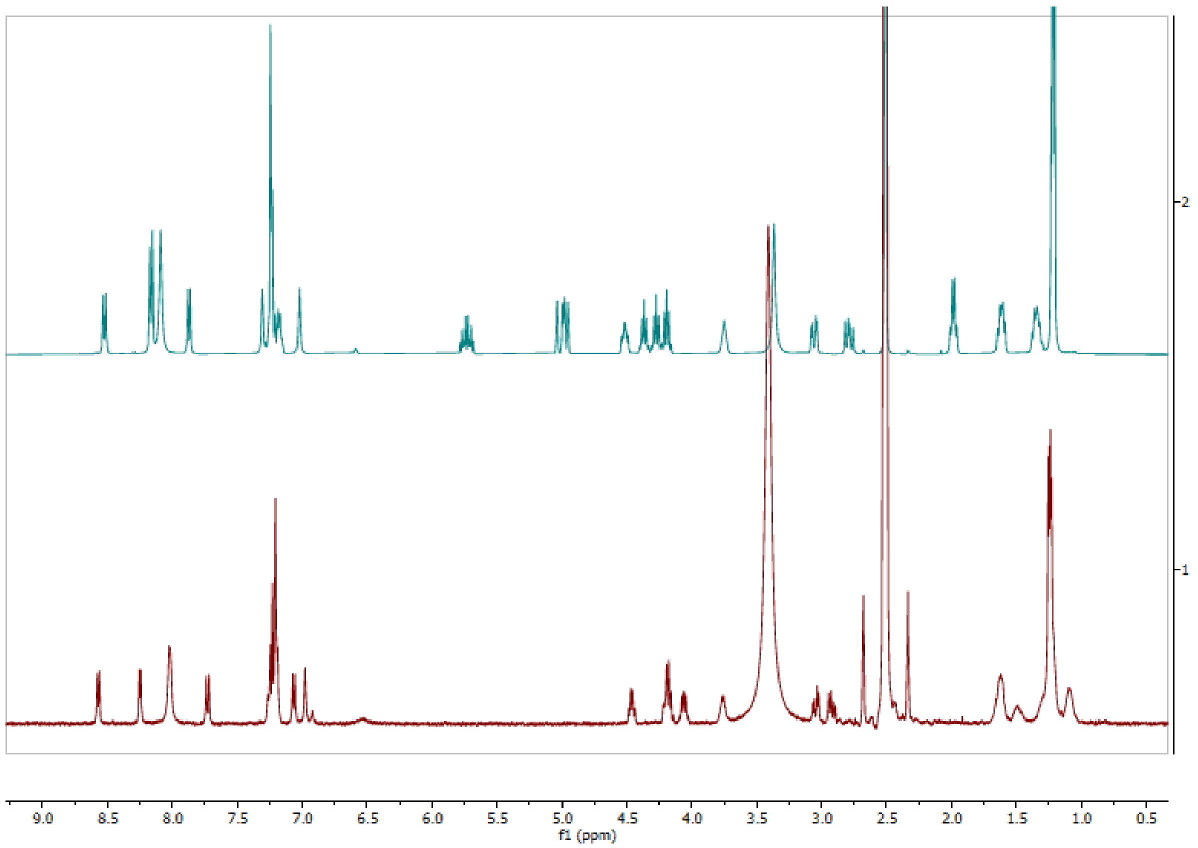

Figure S71. NMR comparison of cyclised of H-(pG)AFAC-NH_2 (**11**; bottom) and the starting peptide **10** (top) showing disappearance of allylic protons at 5.68 and 5.00 ppm.

Cyclisation of Acr-AFAC-NH_2

Peptide **13** (Acr-AFAC-NH_2; 1 mg, 2.16 µmol) was subjected to the optimised cyclisation protocol. The crude reaction mixture was analysed by HPLC and LC-MS.

Crude reaction mixture t = 0 min (prior to irradiation)

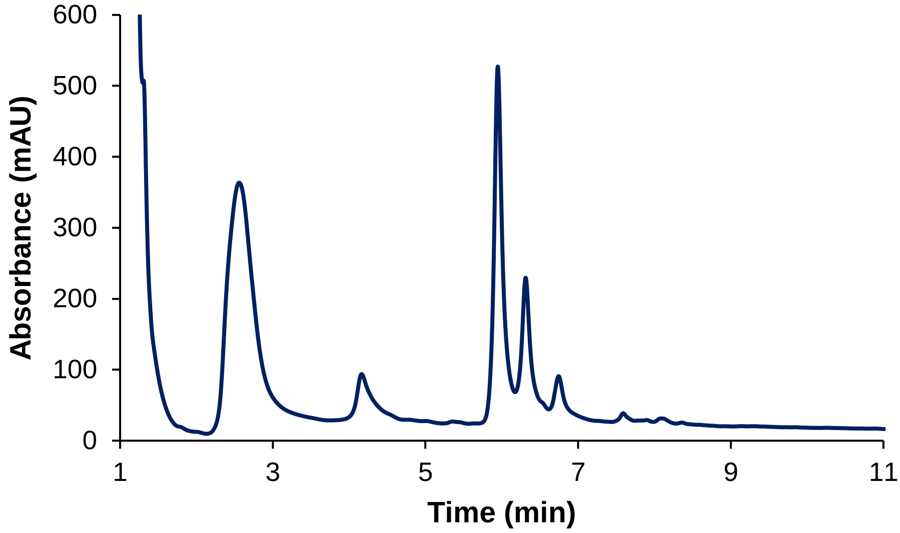

Figure S72. Crude analytical HPLC trace of Acr-AFAC-NH_2 (**13**) cyclisation at t=0 prior to irradiation; analytical gradient 10-50% B over 10 minutes, 210 nm.

Crude reaction mixture t = 60 min

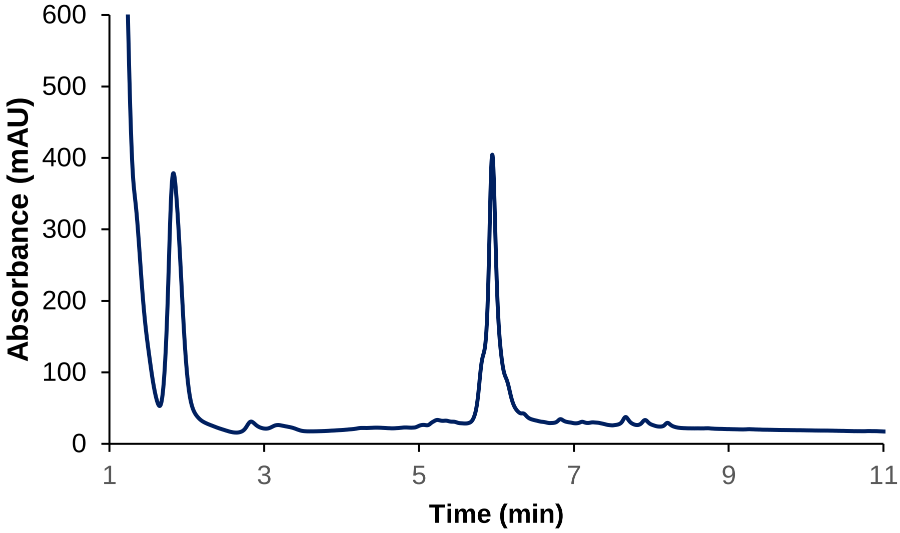

Figure S73. Crude analytical HPLC trace of Acr-AFAC-NH_2 (**13**) cyclisation at 60 mins; analytical gradient 10-50% B over 10 minutes, 210 nm.

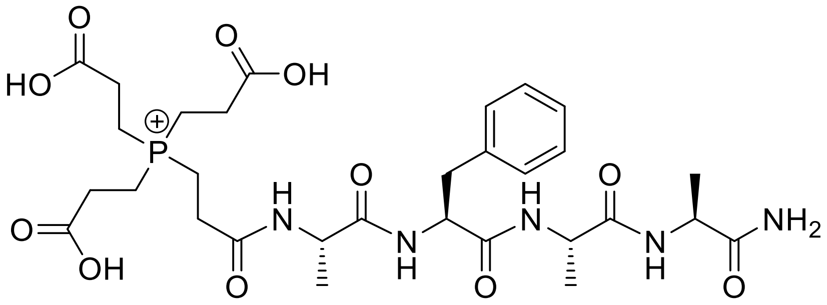

Exact Mass: 682.2848

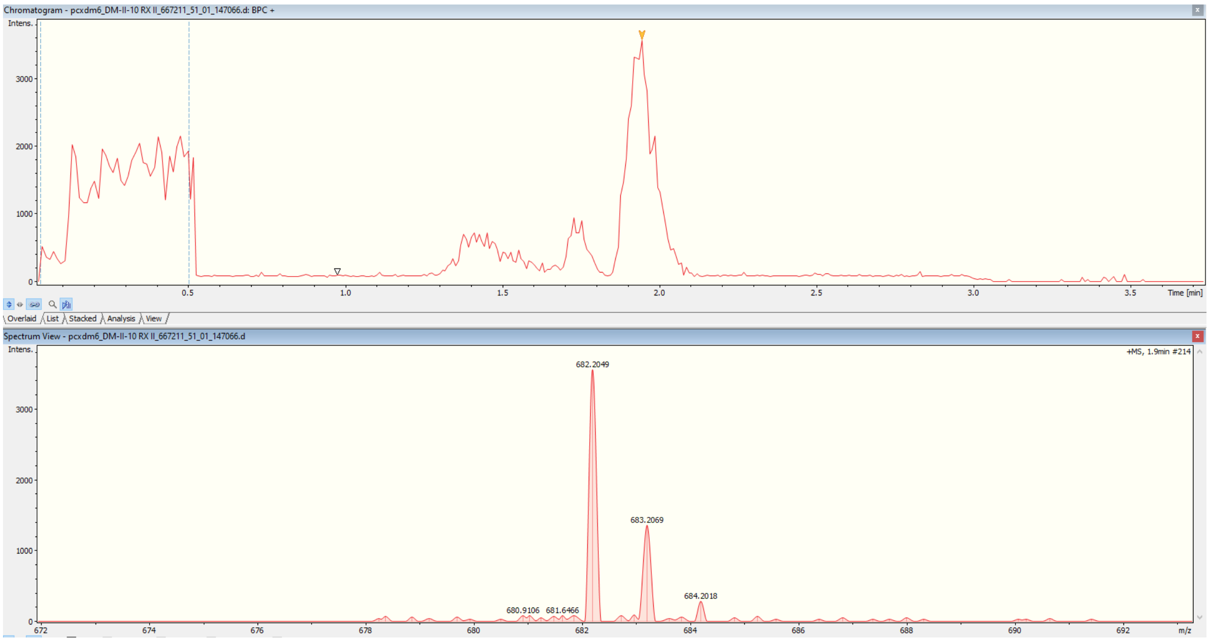

Figure S74. Crude analytical LC-MS TOF trace and MS ESI of crude cyclisation reaction mixture of Acr-AFAC-NH_2 (**13**) at t = 60 minutes; analytical gradient 10-50% B over 10 minutes, 210 nm. Proposed structure of the detected side product formed. Calculated mass $[M+H]^+$: 682.28; observed $[M+H]^+$: 682.20.

Cyclisation of Ac-(alG)AFEC-NH_2 (**21**)

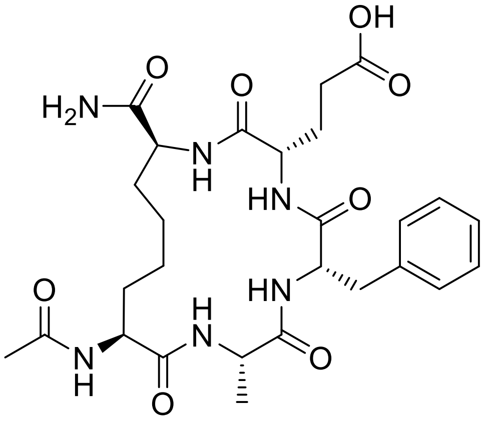

Product **21** was synthesised following the optimised cyclisation protocol using Ac-(alG)AFEC-NH_2 (**14**, 2 mg, 3.30 µmol). After analysis the remaining solution (3.25 µmol) was purified using semi-preparative HPLC (15-80% B over 30 minutes) and fractions containing the desired product lyophilised to yield the title compound as a fluffy white solid (1.4 mg, 2.44 µmol, 74% yield).

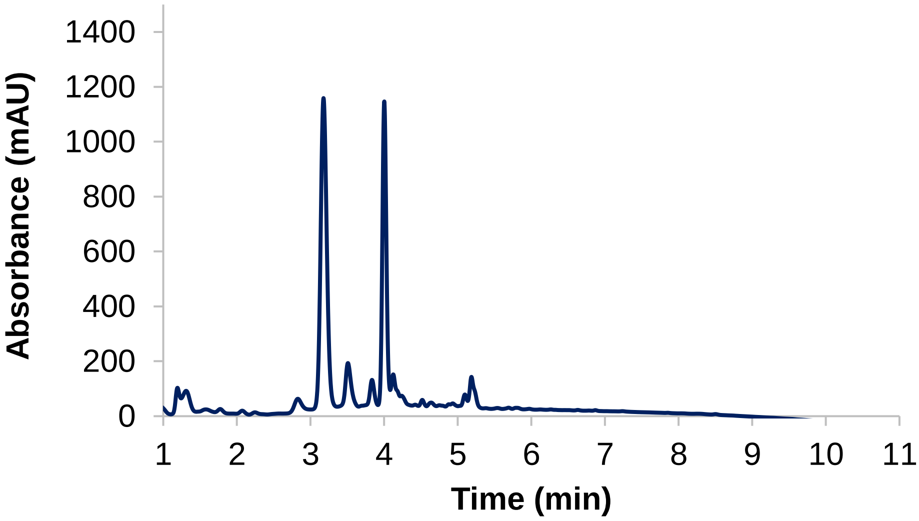

Figure S75. Crude analytical HPLC trace of Ac-(alG)AFEC-NH_2 (**21**); analytical gradient 15-80% B over 10 minutes, 210 nm.

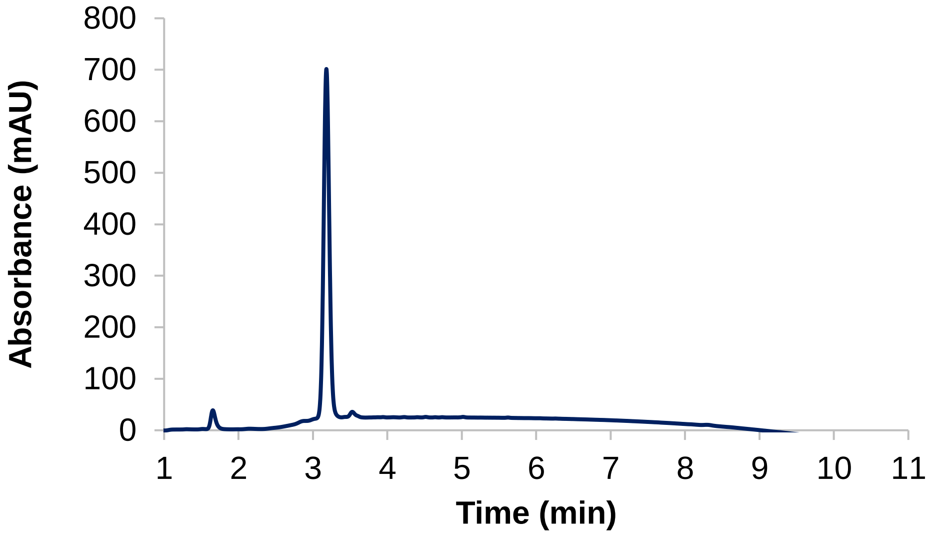

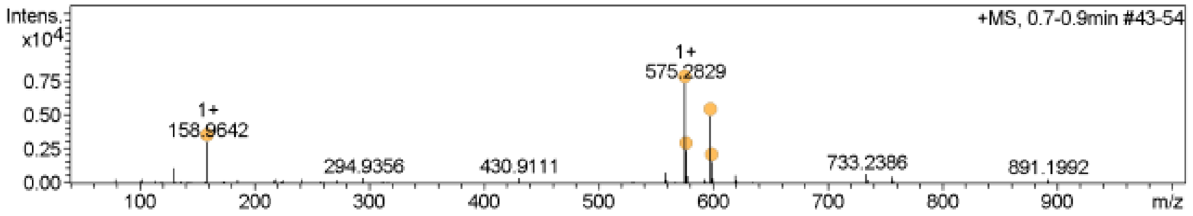

Figure S76. Analytical HPLC trace and ESI MS of purified cyclisation of Ac-(alG)AFEC-NH_2 (**21**); analytical gradient 15-80% B over 10 minutes, 280 nm. Calculated mass $[M+H]^+$: 575.28; observed mass $[M+H]^+$: 575.28.

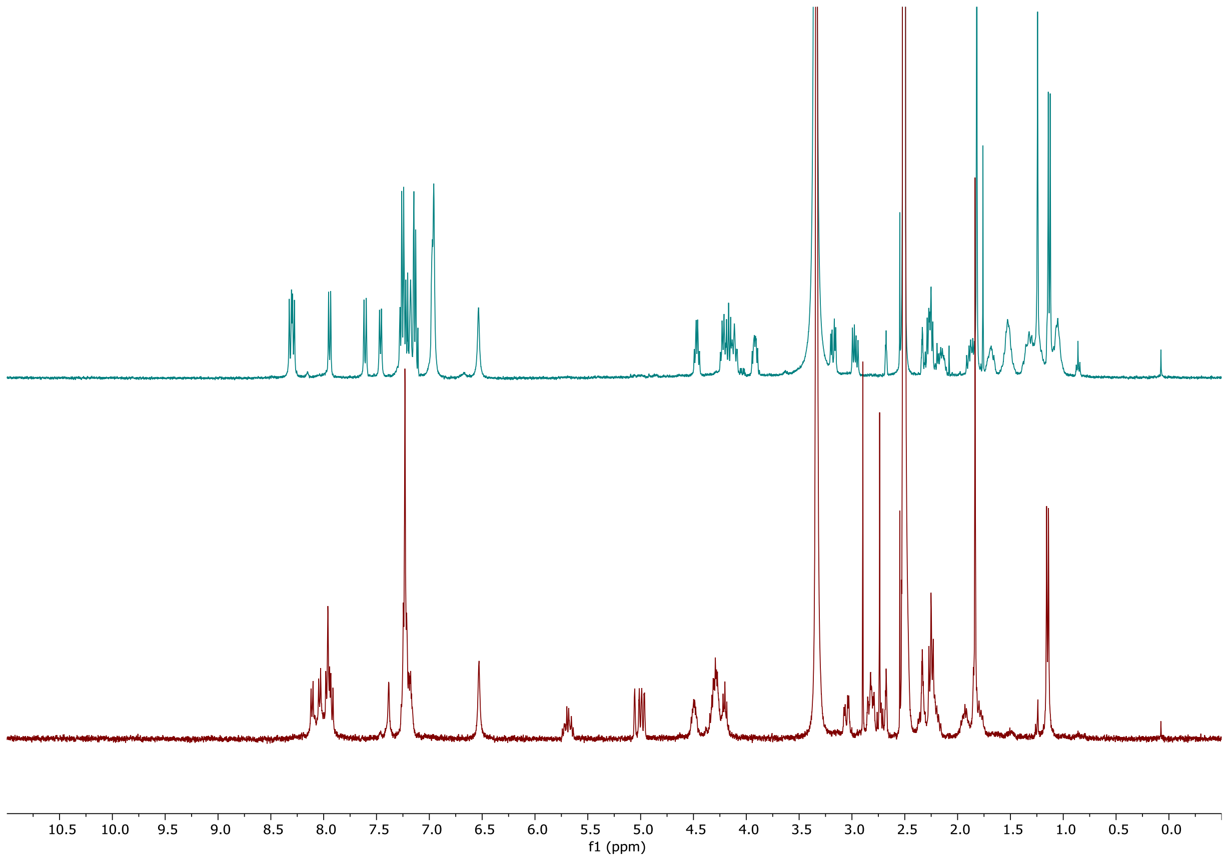

Figure S77. NMR comparison of cyclised Ac-(alG)AFEC-NH_2 (**21**, top) and the starting peptide **14** (bottom) showing disappearance of allylic protons at 5.68 and 5.00 ppm.

Peptide	Acetylated	Free *N*-terminus
H-CAFE(alG)-NH2	0%	25%
H-CIWR(alG)-NH2	7%	0%
H-CLYM(alG)-NH2	21%	0%
H-CVHQ(alG)-NH2	28%	0%
H-CTPK(alG)-NH2	0%	0%

Table S1. Problematic cyclisations for peptides carrying an *N*-terminal Cys residue and a *C*-terminal allyl glycine.

Cyclisation of Ac-(alG)IWRC-NH_2 (**22**)

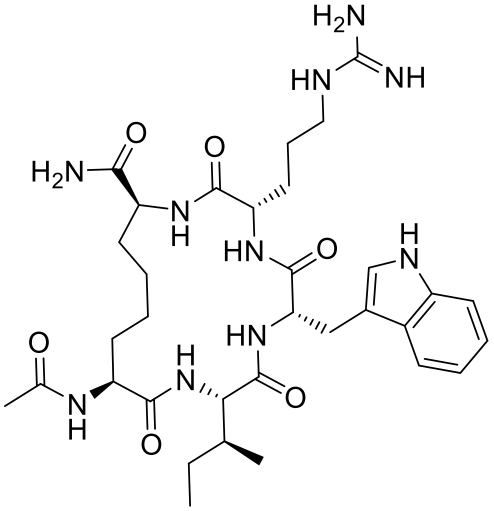

Product **22** was synthesised following the optimised cyclisation protocol using Ac-(alG)IWRC-NH_2 (**15**, 2 mg, 2.80 µmol). After analysis the remaining solution (2.75 µmol) was purified using semi-preparative HPLC (15-80% B over 30 minutes) and fractions containing the desired product lyophilised to yield the title compound as a fluffy white solid (1.1 mg, 1.61 µmol, 59% yield).

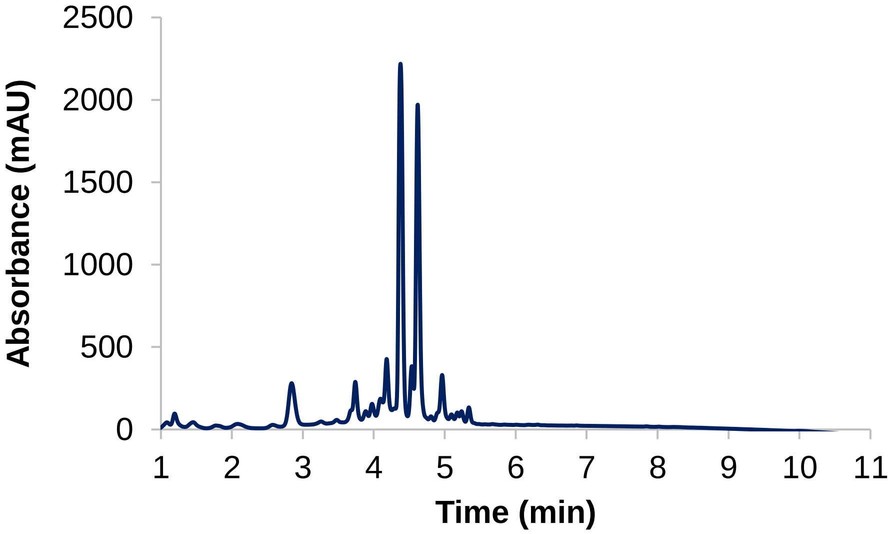

Figure S78. Crude analytical HPLC trace of Ac-(alG)IWRC-NH_2 (**22**); analytical gradient 15-80% B over 10 minutes, 210 nm.

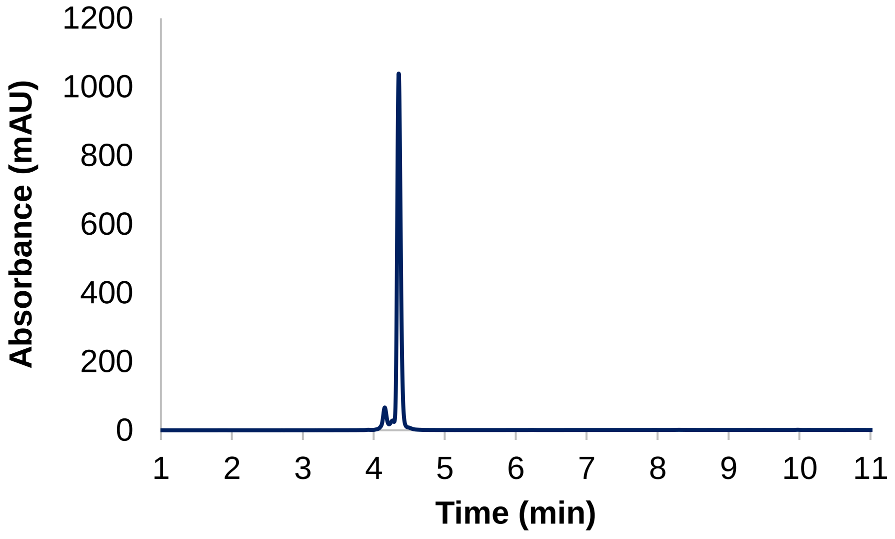

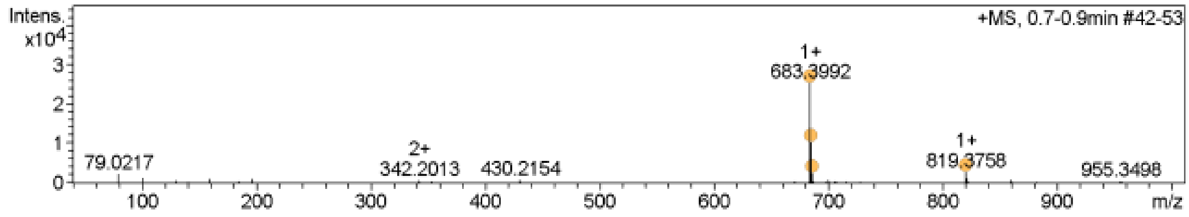

Figure S79. Analytical HPLC trace and ESI MS of purified cyclisation of Ac-(alG)IWRC-NH_2 (**22**); analytical gradient 15-80% B over 10 minutes, 280 nm. Calculated mass $[M+H]^+$: 683.39; observed mass $[M+H]^+$: 683.39.

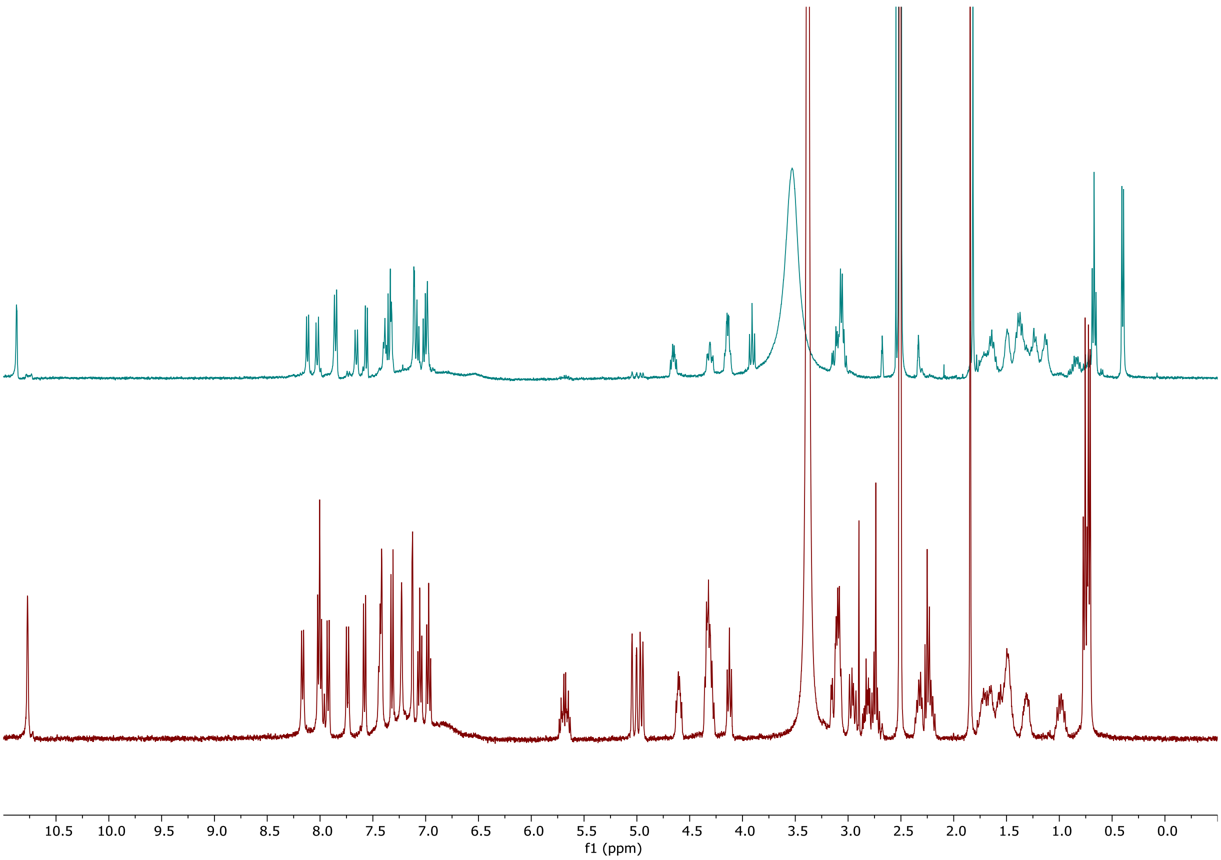

Figure S80. NMR comparison of cyclised Ac-(alG)IWRC-NH_2 (**22**, top) and the starting peptide **15** (bottom) showing disappearance of allylic protons at 5.67 and 4.97 ppm.

Cyclisation of Ac-(alG)LYMC-NH2 (**23a**)

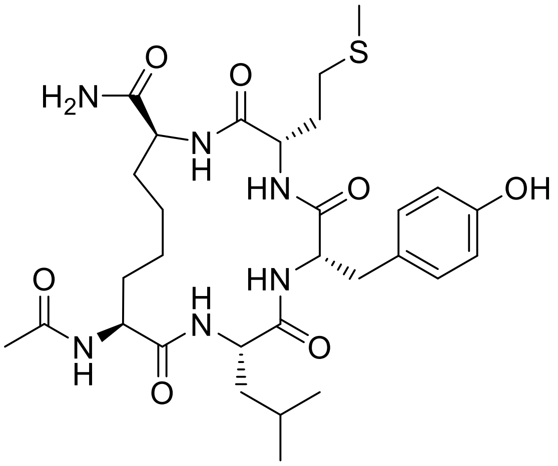

Product **23a** was synthesised following the optimised cyclisation protocol using Ac-(alG)LYMC-NH2 (**16a**, 2 mg, 3.00 µmol). After analysis the remaining solution (2.95 µmol) was purified using semi-preparative HPLC (15-80% B over 30 minutes) and fractions containing the desired product lyophilised to yield the title compound as a fluffy white solid (1.3 mg, 2.05 µmol, 69% yield).

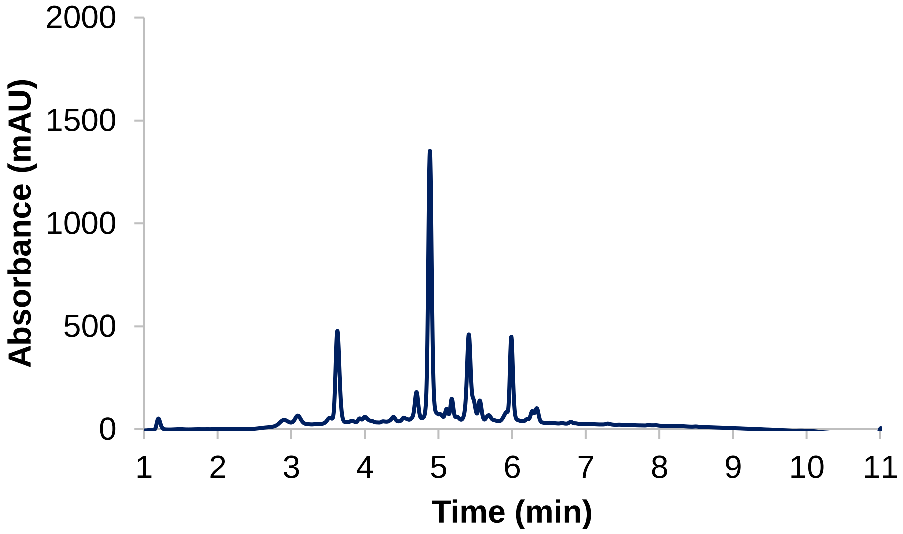

Figure S81. Crude analytical HPLC trace of Ac-(alG)LYMC-NH2 (**23a**); analytical gradient 15-80% B over 10 minutes, 210 nm.

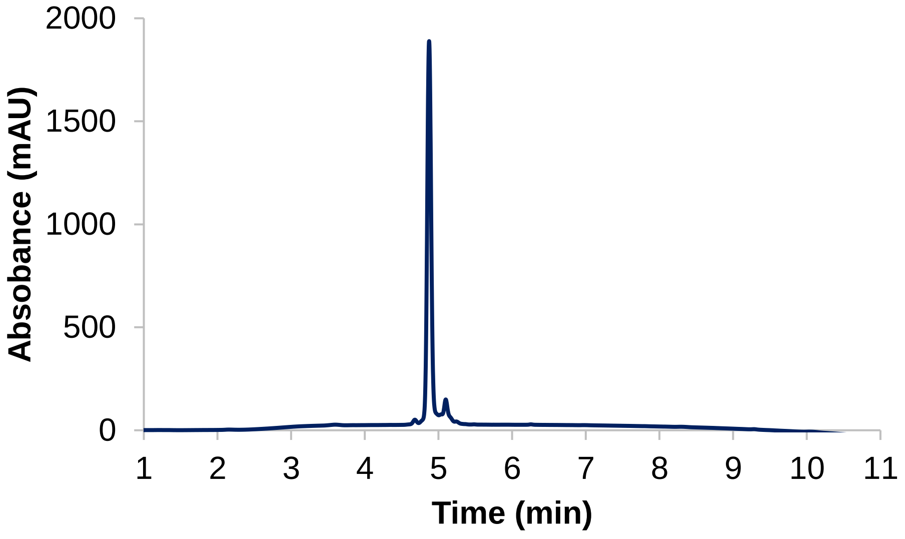

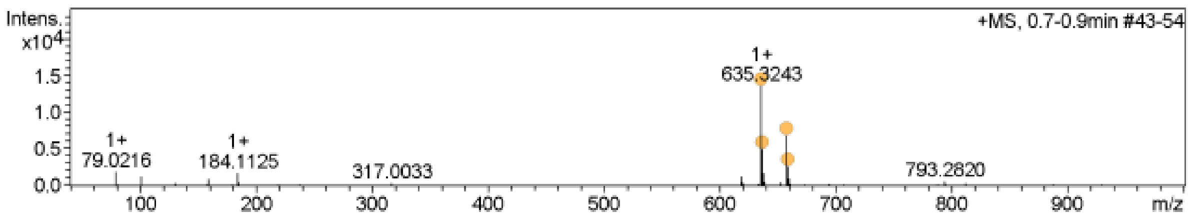

Figure S82. Analytical HPLC trace and ESI MS of purified cyclisation of Ac-(alG)LYMC-NH_2 (**23a**); analytical gradient 15-80% B over 10 minutes, 210 nm. Calculated mass $[M+H]^+$: 635.32; observed mass $[M+H]^+$: 635.32.

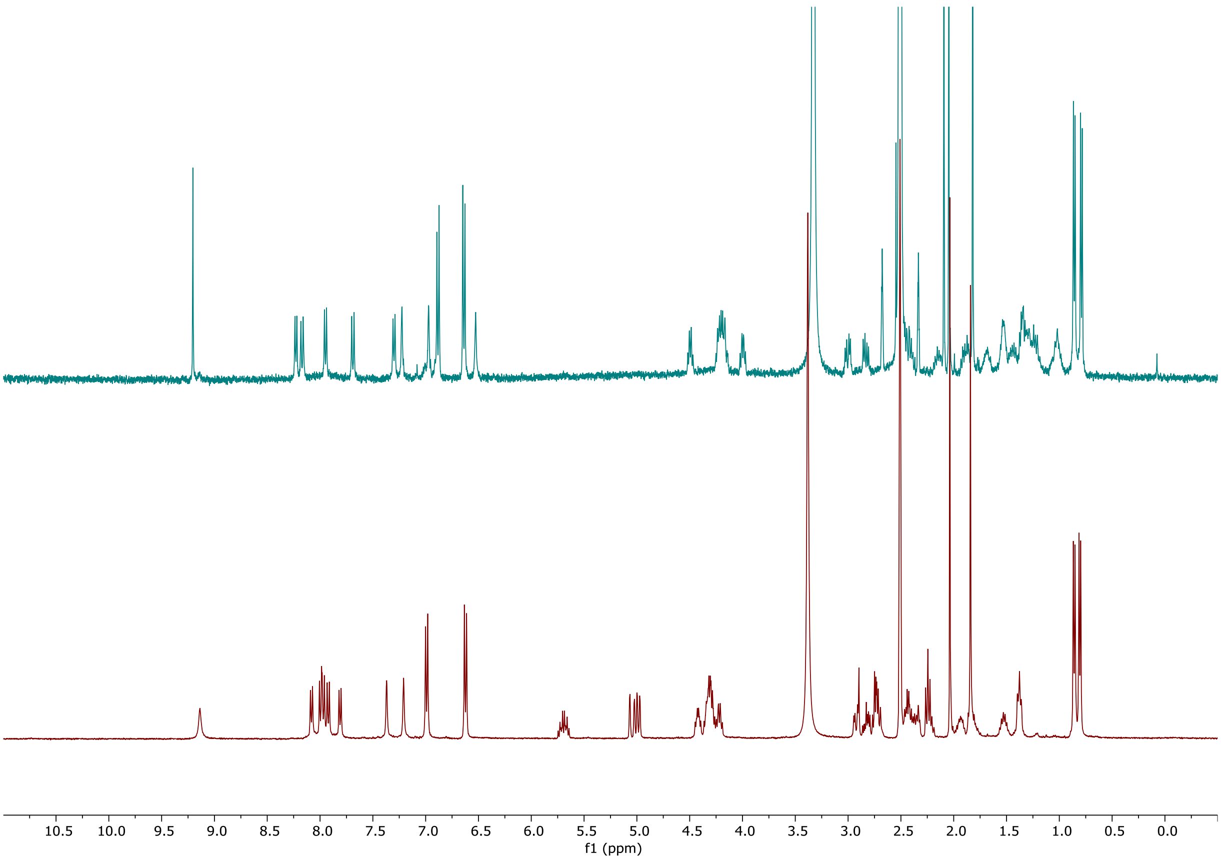

Figure S83. NMR comparison of cyclised Ac-(alG)LYMC-NH2 (**23a**, top) and the starting peptide **16a** (bottom) showing disappearance of allylic protons at 5.69 and 5.02 ppm.

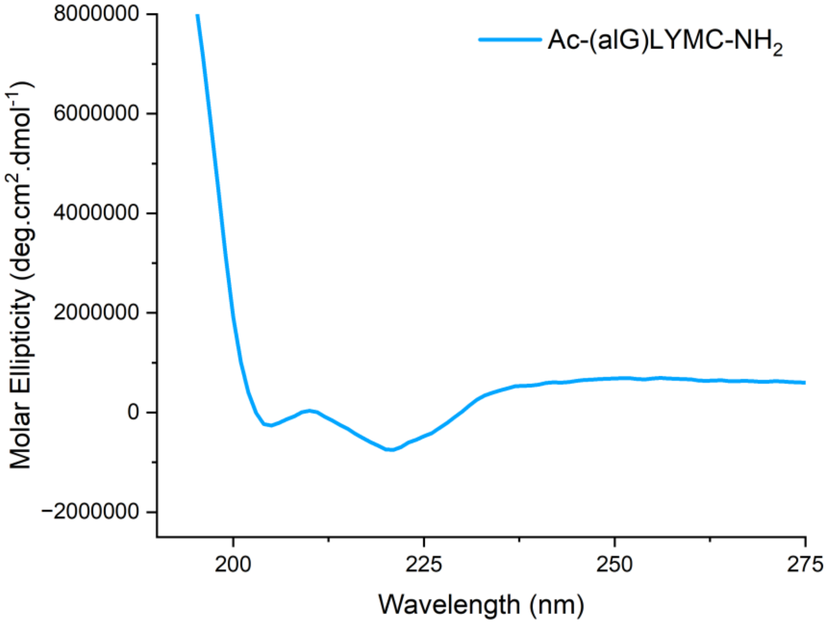

Figure S84. Circular dichroism of cyclised product **23a.**

Cyclisation of H-(alG)LYMC-NH_2 (**23b**)

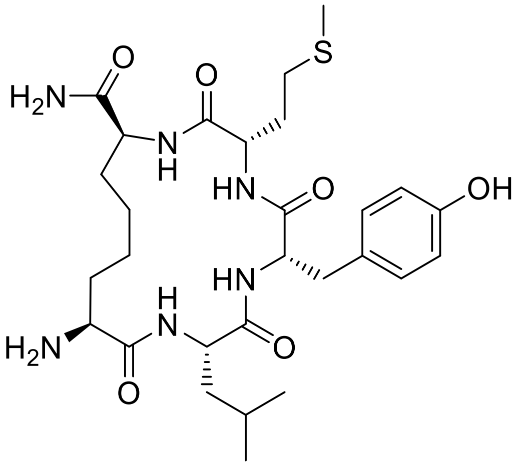

Product **23b** was synthesised following the optimised cyclisation protocol using H-(alG)LYMC-NH_2 (**16b**, 2 mg, 3.21 µmol). After analysis the remaining solution (3.16 µmol) was purified using semi-preparative HPLC (15-80% B over 30 minutes) and fractions containing the desired product lyophilised to yield the title compound as a fluffy white solid (1.0 mg, 1.69 µmol, 54% yield).

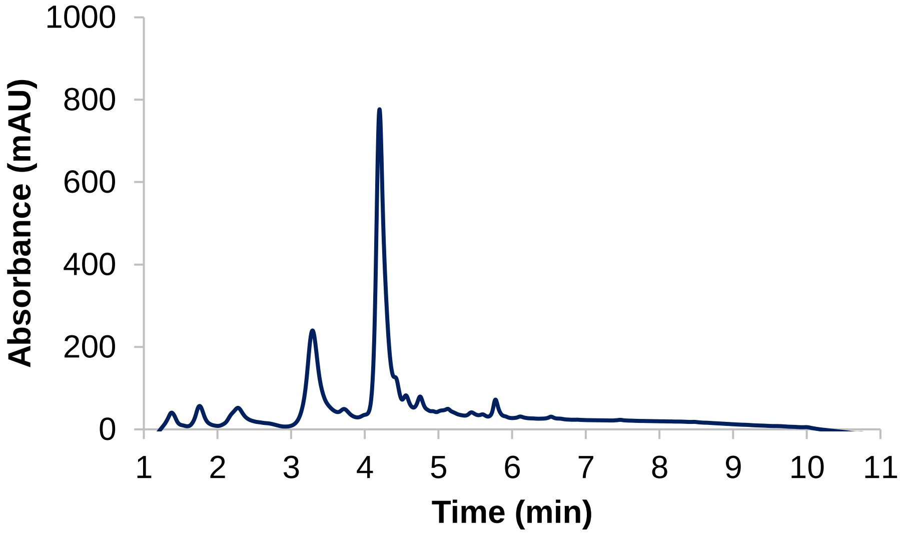

Figure S85. Crude analytical HPLC trace of H-(alG)LYMC-NH_2 (**23b**); analytical gradient 15-80% B over 10 minutes, 210 nm.

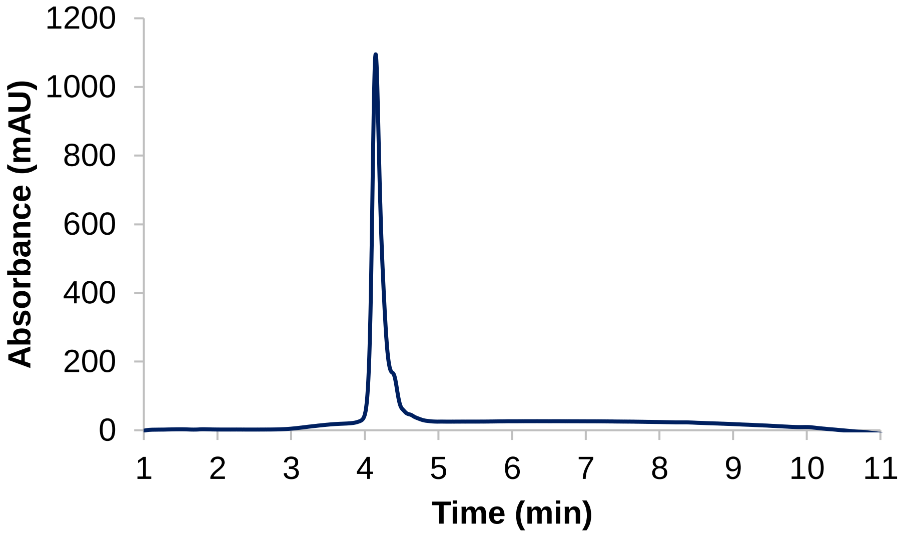

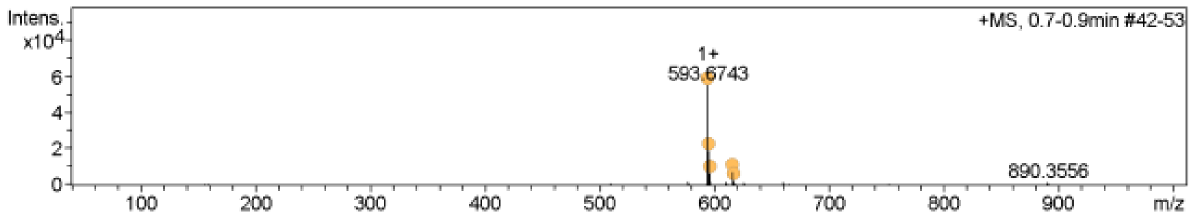

Figure S86. Analytical HPLC trace and ESI MS of purified cyclisation of H-(alG)LYMC-NH_2 (**23b**); analytical gradient 15-80% B over 10 minutes, 210 nm. Calculated mass $[M+H]^+$: 593.31; observed mass $[M+H]^+$: 593.67.

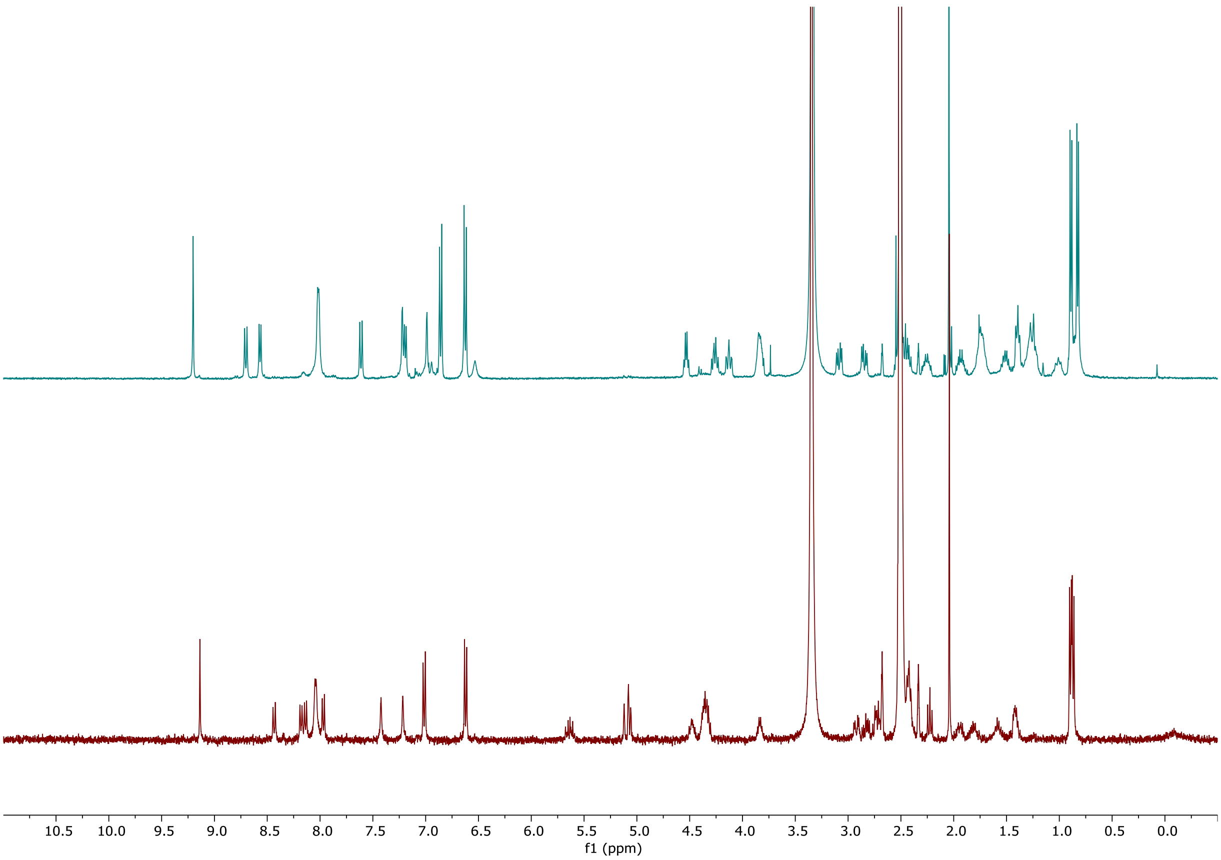

Figure S87. NMR comparison of cyclised H-(alG)LYMC-NH_2 (**23b**, top) and the starting peptide **16b** (bottom) showing disappearance of allylic protons at 5.63 and 5.08 ppm.

Cyclisation of Ac-(alG)VHQC-NH_2 (**24**)

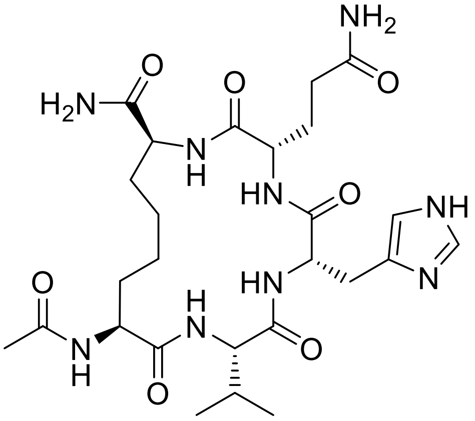

Product **24** was synthesised following the optimised cyclisation protocol using Ac-(alG)VHQC-NH_2 (**17**, 2 mg, 3.21 µmol). After analysis the remaining solution (3.16 µmol) was purified using semi-preparative HPLC (2-95% B over 30 minutes) and fractions containing the desired product lyophilised to yield the title compound as a fluffy white solid (1.1 mg, 1.86 µmol, 59% yield).

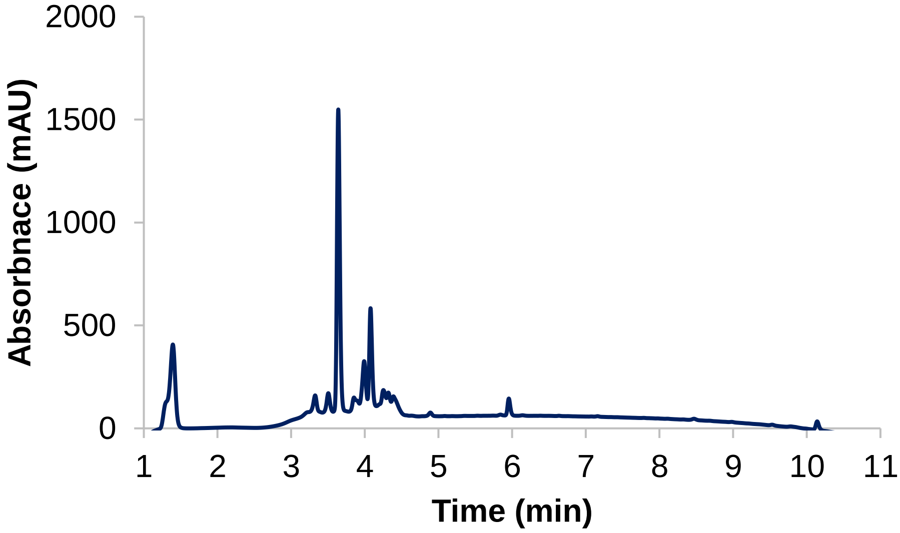

Figure S88. Crude analytical HPLC trace of Ac-(alG)VHQC-NH_2 (**24**); analytical gradient 2-95% B over 10 minutes, 210 nm.

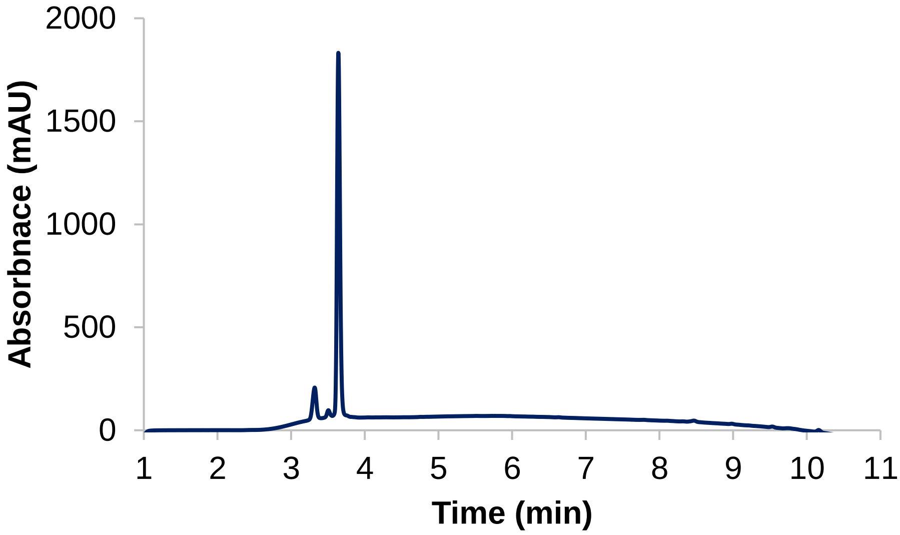

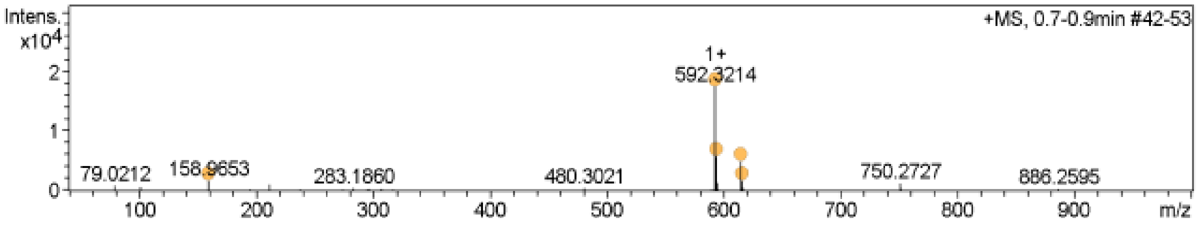

Figure S89. Analytical HPLC trace and ESI MS of purified cyclisation of Ac-(alG)VHQC- NH_2 (**24**); analytical gradient 2-95% B over 10 minutes, 210 nm. Calculated mass $[M+H]^+$: 592.32; observed mass $[M+H]^+$: 592.32.

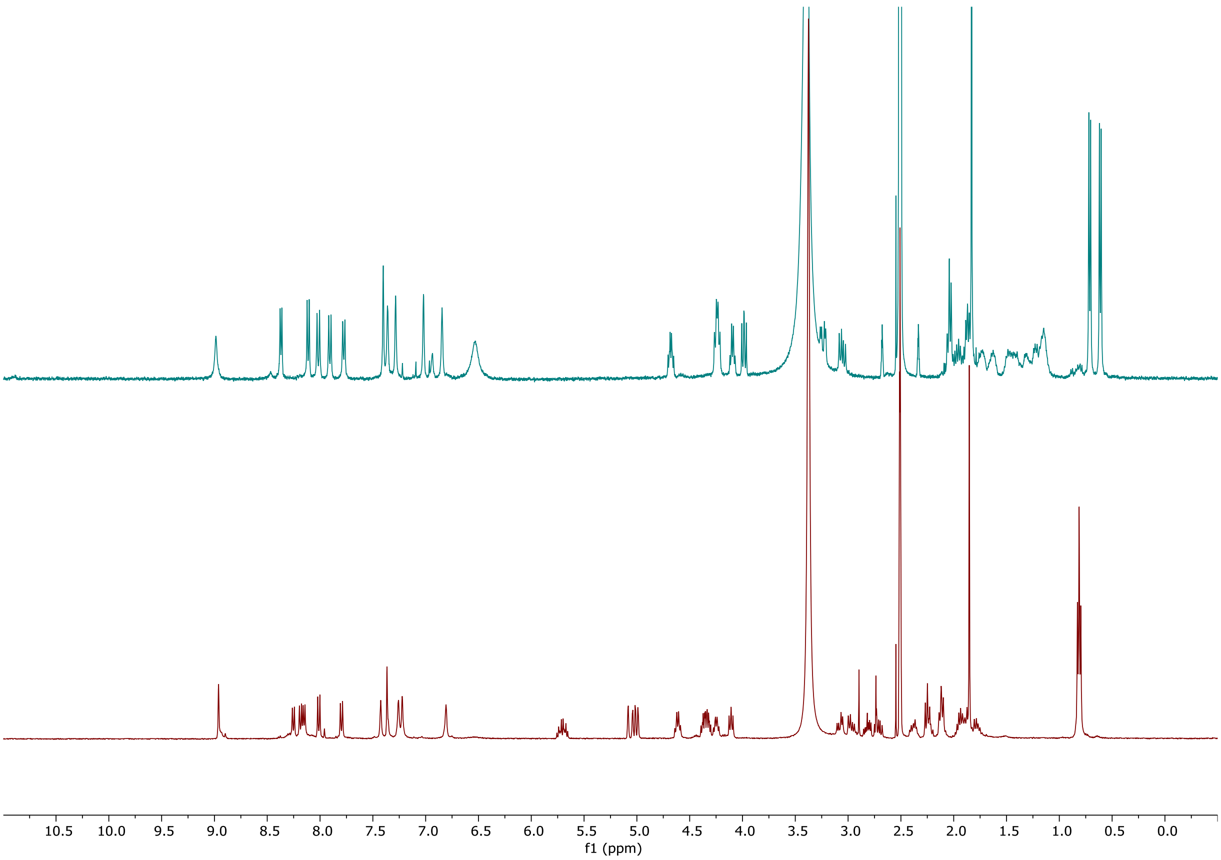

Figure S90. NMR comparison of cyclised Ac-(alG)VHQC- NH_2 (**24**, top) and the starting peptide **17** (bottom) showing disappearance of allylic protons at 5.69 and 5.02 ppm.

Cyclisation of Ac-(alG)TPKC-NH_2 (**25**)

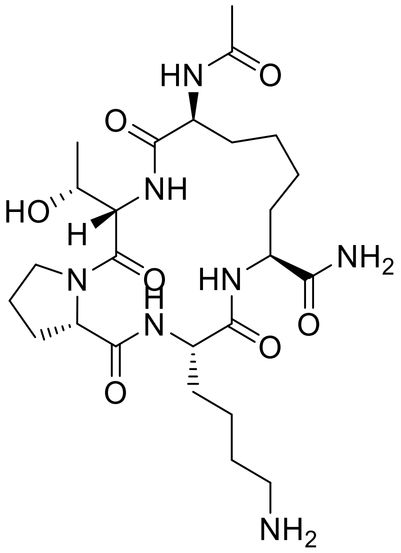

Product **25** was synthesised following the optimised cyclisation protocol using Ac-(alG)TPKC-NH_2 (**18**, 2 mg, 3.42 µmol). After analysis the remaining solution (3.37 µmol) was purified using semi-preparative HPLC (2-95% B over 30 minutes) and fractions containing the desired product lyophilised to yield the title compound as a fluffy white solid (0.9 mg, 1.63 µmol, 48% yield).

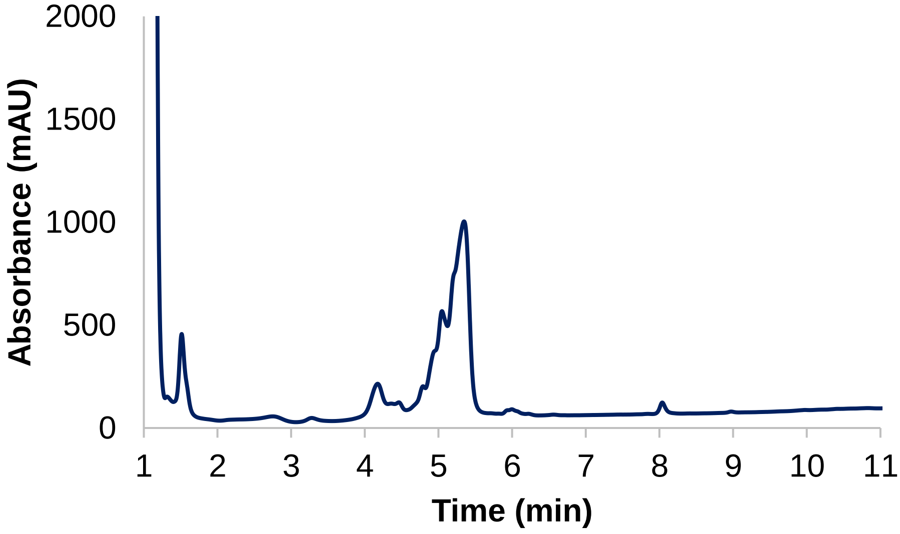

Figure S91. Crude analytical HPLC trace of Ac-(alG)TPKC-NH_2 (**25**); analytical gradient 5-60% B over 10 minutes, 210 nm.

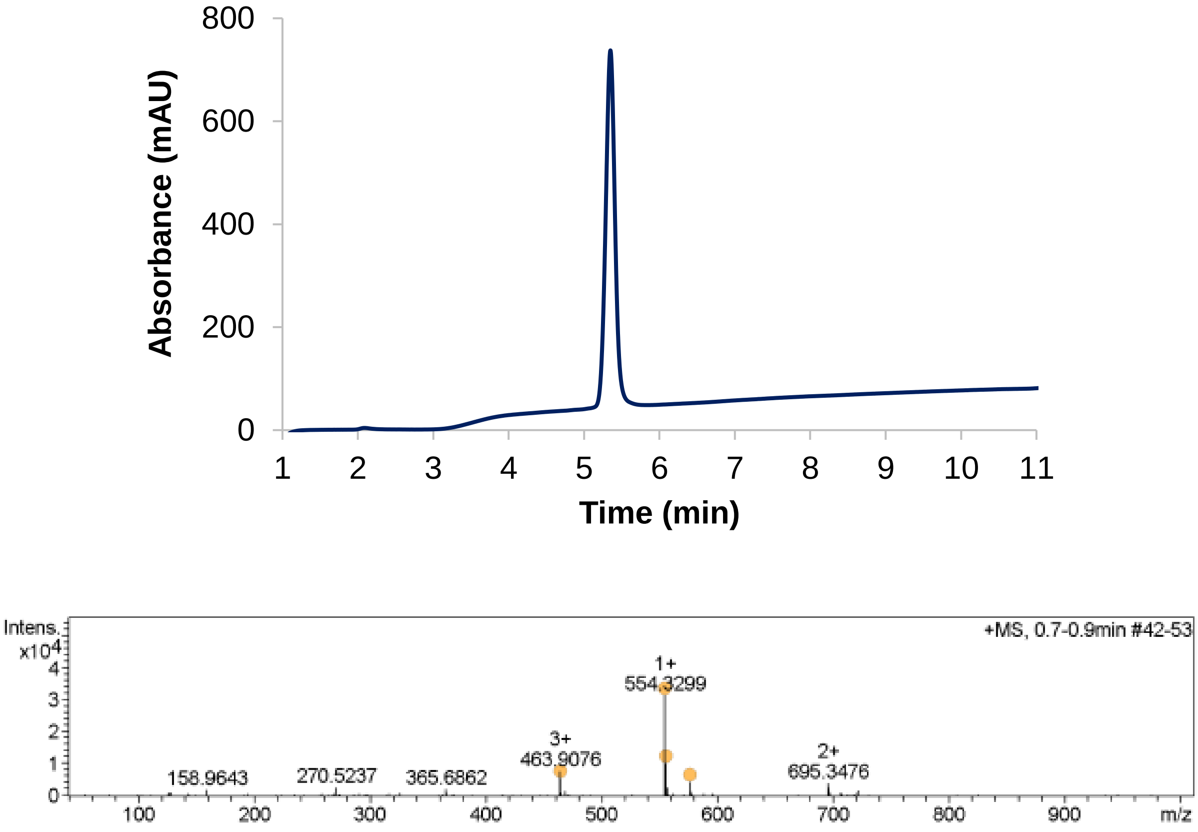

Figure S92. Analytical HPLC trace and ESI MS of purified cyclisation of Ac-(alG)TPKC-NH_2 (**25**); analytical gradient 5-60% B over 10 minutes, 210 nm. Calculated mass $[M+H]^+$: 554.33; observed mass $[M+H]^+$: 554.33.

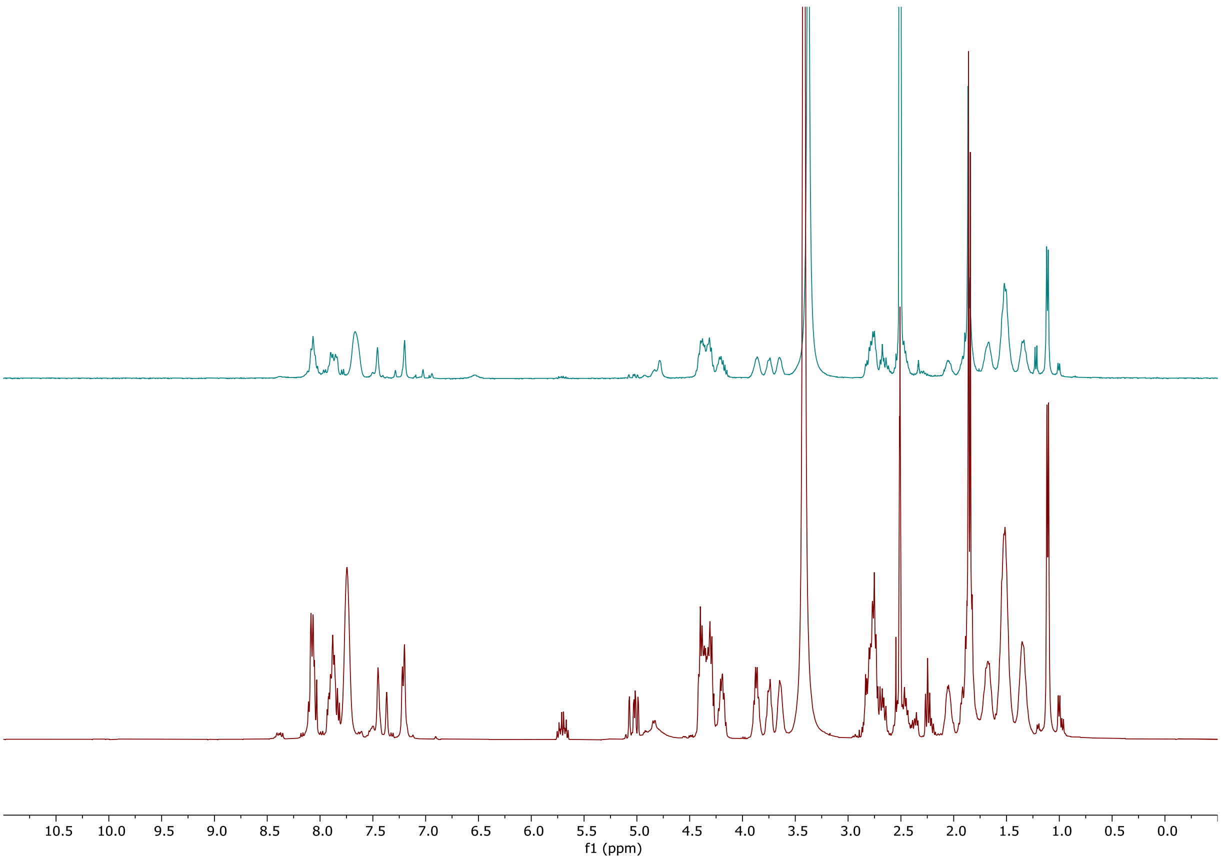

Figure S93. NMR comparison of cyclised Ac-(alG)TPKC-NH_2 (**25**, top) and the starting peptide **18** (bottom) showing disappearance of allylic protons at 5.69 and 5.02 ppm.

Cyclisation of Ac-(alG)VGDC-NH_2 (**26**)

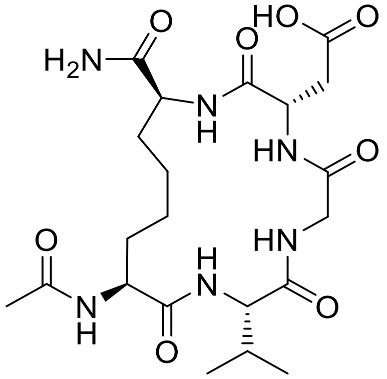

Product **26** was synthesised following the optimised cyclisation protocol using Ac-(alG)VGDC-NH_2 (**19**, 2 mg, 3.77 µmol). After analysis the remaining solution (3.72 µmol) was purified using semi-preparative HPLC (5-60% B over 30 minutes) and fractions containing the desired product lyophilised to yield the title compound as a fluffy white solid (1.0 mg, 2.00 µmol, 54% yield).

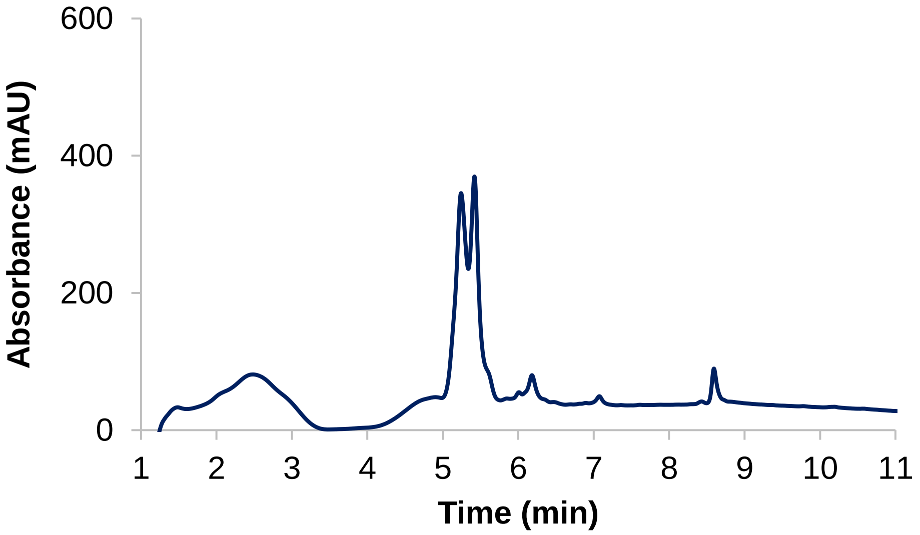

Figure S94. Crude analytical HPLC trace of Ac-(alG)VGDC-NH_2 (**26**); analytical gradient 2-70% B over 10 minutes, 210 nm.

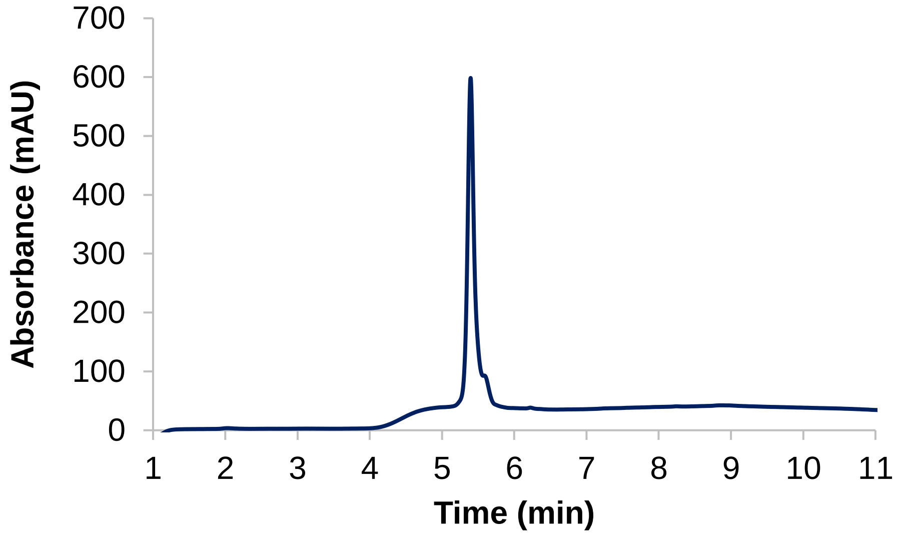

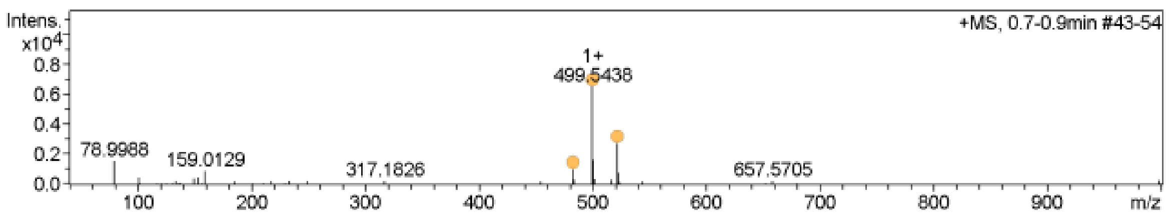

Figure S95. Analytical HPLC trace and ESI MS of purified cyclisation of Ac-(alG)VGDC-NH_2 (**26**); analytical gradient 2-70% B over 10 minutes, 210 nm. Calculated mass $[M+H]^+$: 499.54; observed mass $[M+H]^+$ 499.54.

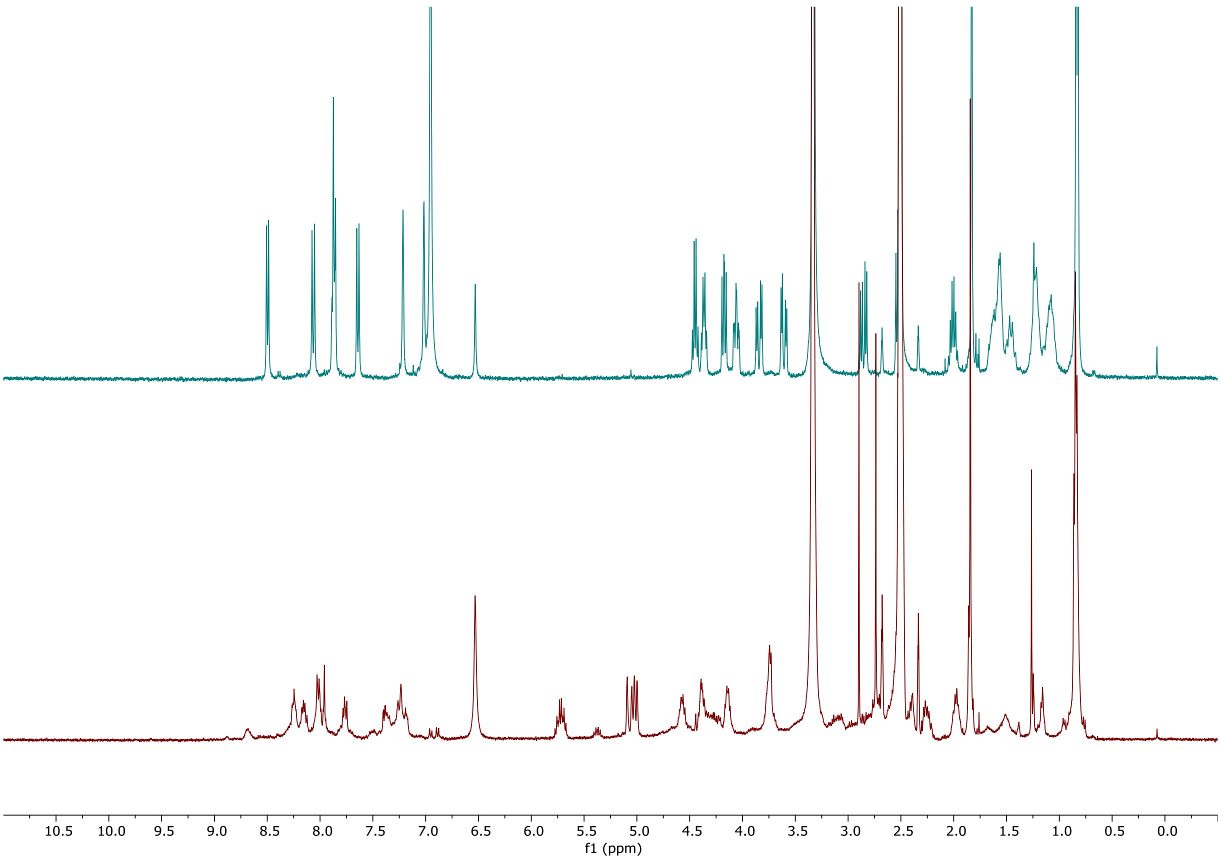

Figure S96. NMR comparison of cyclised Ac-(alG)VGDC-NH_2 (**26**, top) and the starting peptide (**19**, bottom) showing disappearance of allylic protons at 5.72 and 5.04 ppm.

Cyclised H-D(alG)FYQC-NH_2 (**27**)

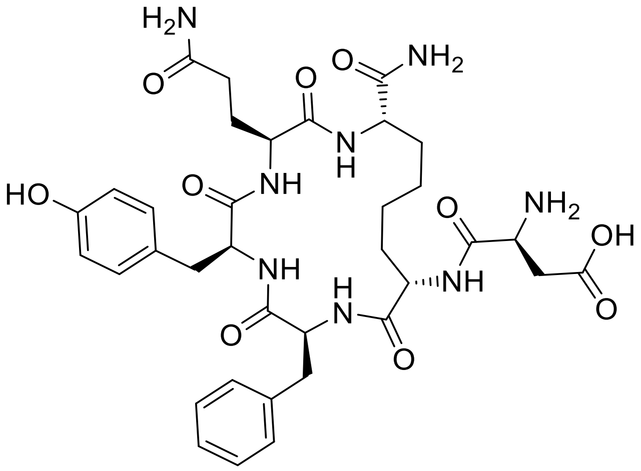

Product **27** was synthesised following the optimised cyclisation protocol using H-DalGFYQCC-NH_2 (**20**, 4 mg, 5.20 µmol). After analysis the remaining solution (5.10 µmol) was purified using semi-preparative HPLC (10-50% B over 30 minutes) and fractions containing the desired product lyophilised to yield the title compound as a fluffy white solid (3.1 mg, 4.20 µmol, 82% yield).

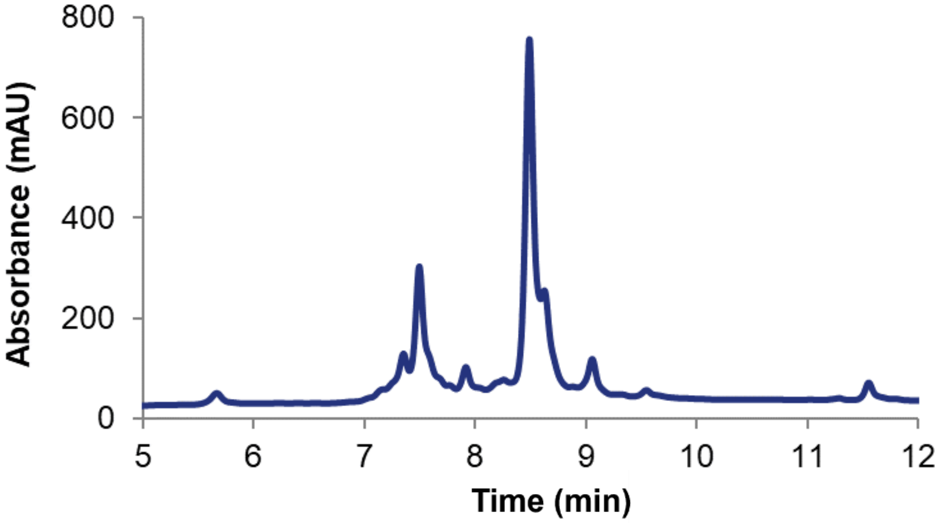

Figure S97. Crude analytical HPLC trace of H-DalGFYQC-NH_2 (**27**) macrocyclisation; analytical gradient 2-40% B over 10 minutes, 210 nm.

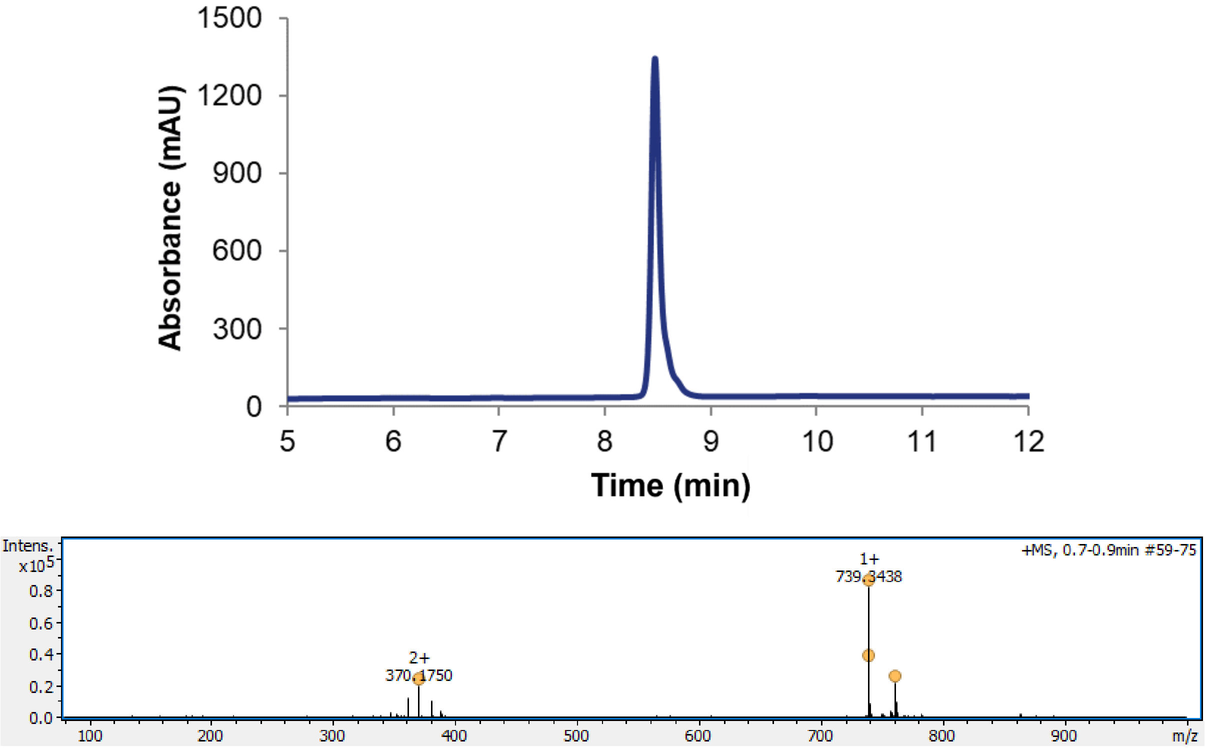

Figure S98. Analytical HPLC trace and ESI MS of cyclised H-DaIGFYQC-NH_2 (**27**); analytical gradient 2-40% B over 10 minutes, 210 nm. Calculated mass $[M+H]^+$: 739.34, $[M+2H]^{2+}$: 370.17; observed mass $[M+H]^+$: 739.34, $[M+2H]^{2+}$: 370.18.

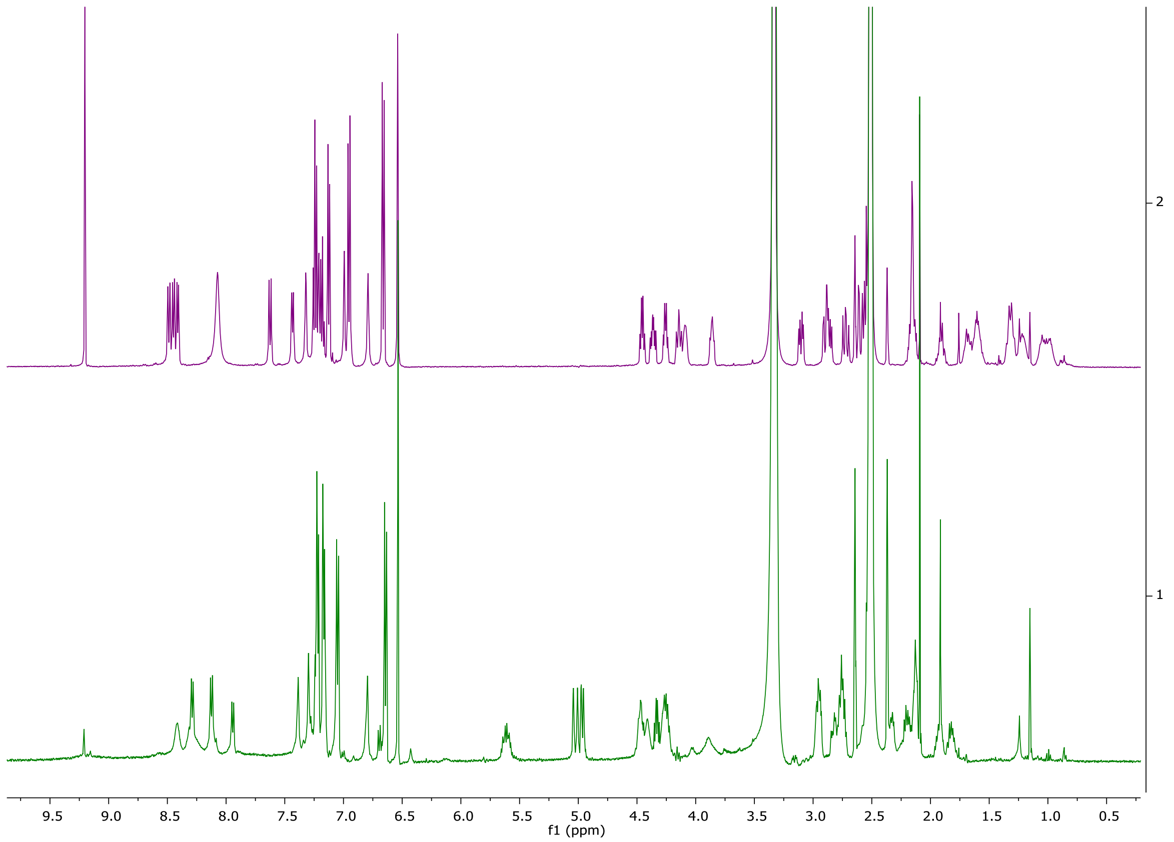

Figure S99. NMR overlay of starting peptide H-D(alG)FYQC-NH_2 (**20**, bottom) and the cyclised product (**27**, top), showing disappearance of allylic protons at 5.7 and 5.0 ppm.

Cyclisation of Ac-(alG)KISYC-NH_2 (**39**)

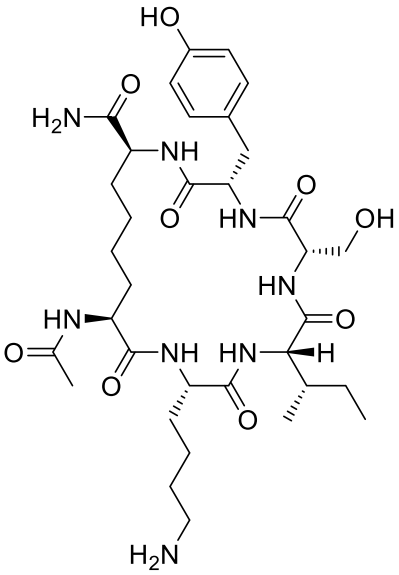

Product **39** was synthesised following the optimised cyclisation protocol using Ac-(alG)KISYC-NH_2 (**28**, 2 mg, 2.67 µmol). After analysis the remaining solution (2.61 µmol) was purified using semi-preparative HPLC (2-95% B over 30 minutes) and fractions containing the desired product lyophilised to yield the title compound as a fluffy white solid (1.2 mg, 1.67 µmol, 64% yield).

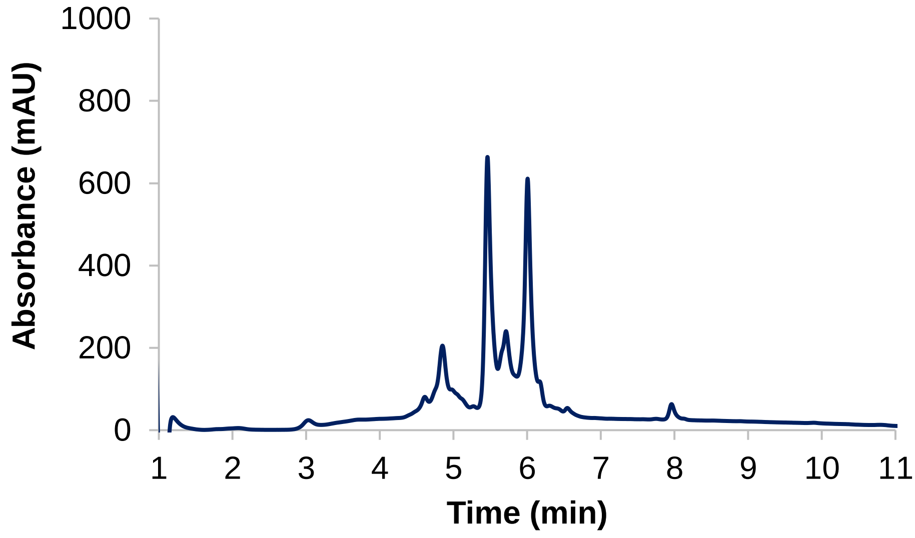

Figure S100. Crude analytical HPLC trace of Ac-(alG)KISYC-NH_2 (**39**); analytical gradient 5-60% B over 10 minutes, 210 nm.

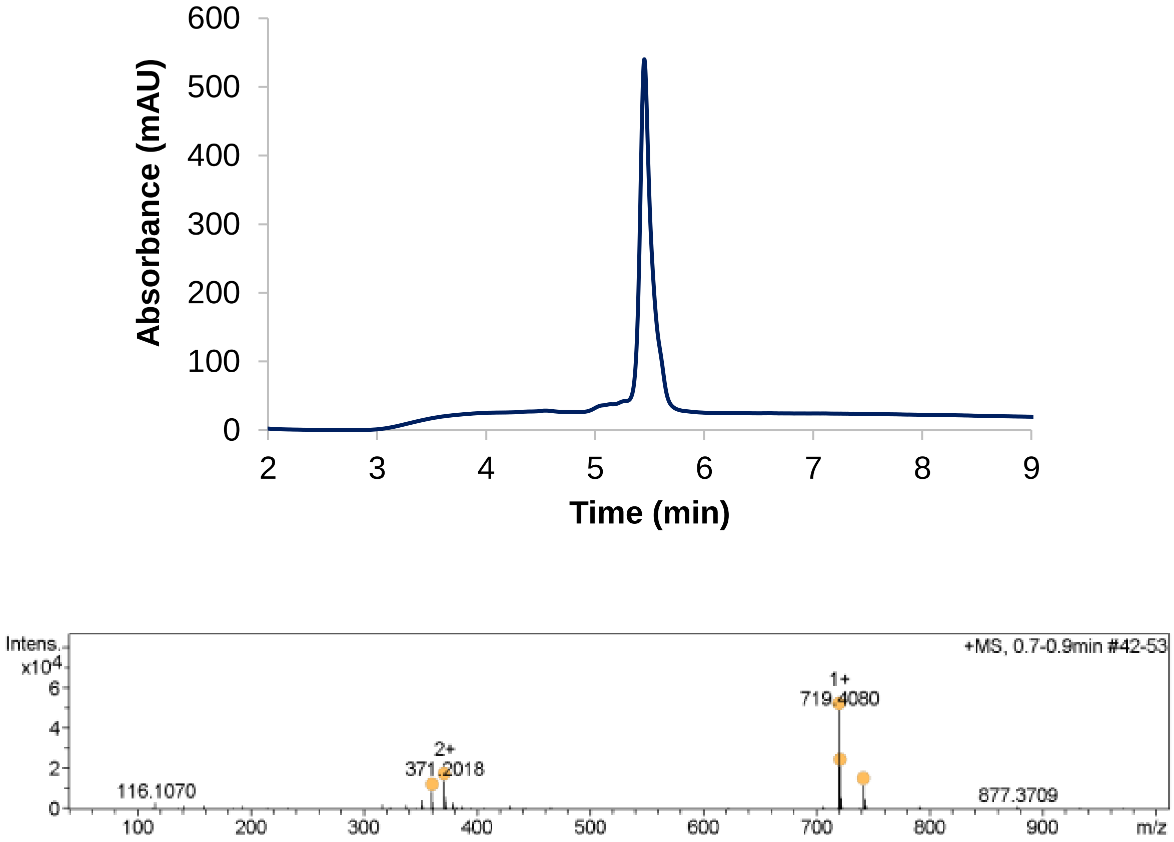

Figure S101. Analytical HPLC trace and ESI MS of purified cyclisation of Ac-(alG)KISYC-NH_2 (**39**); analytical gradient 5-60% B over 10 minutes, 210 nm. Calculated mass $[M+H]^+$: 719.41; observed mass $[M+H]^+$ 719.41.

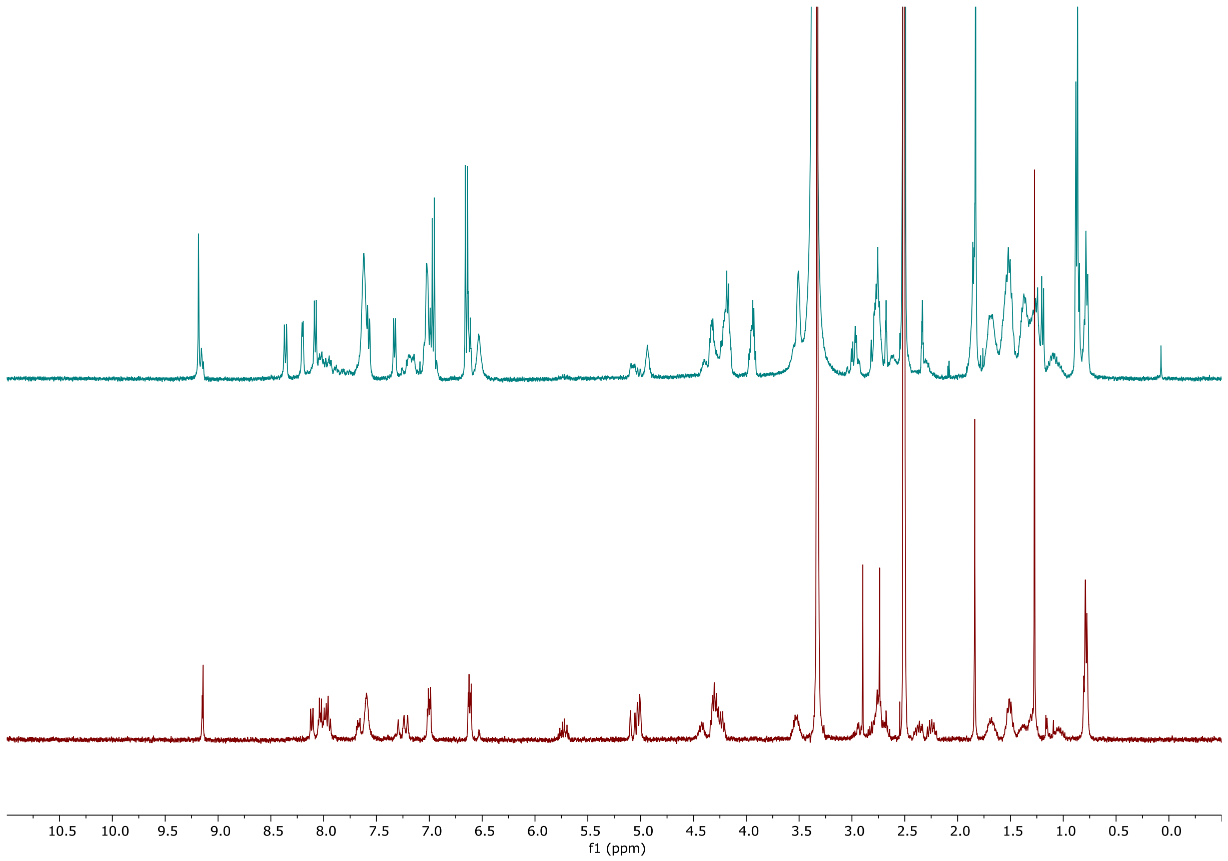

Figure S102. NMR comparison of cyclised Ac-(alG)KISYC-NH_2 (**39**, top) and the starting peptide (**28**, bottom) showing disappearance of allylic protons at 5.72 and 5.05 ppm.

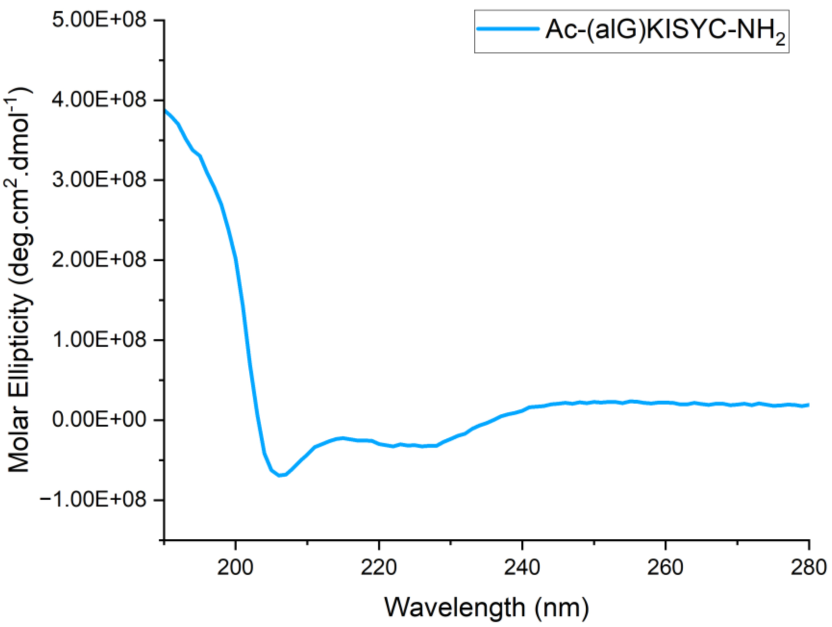

Figure S103. Circular dichroism of cyclised product **39**.

Cyclisation of Ac-(alG)KISYC-NH_2 (**39**) in PBS buffer (10% MeCN)

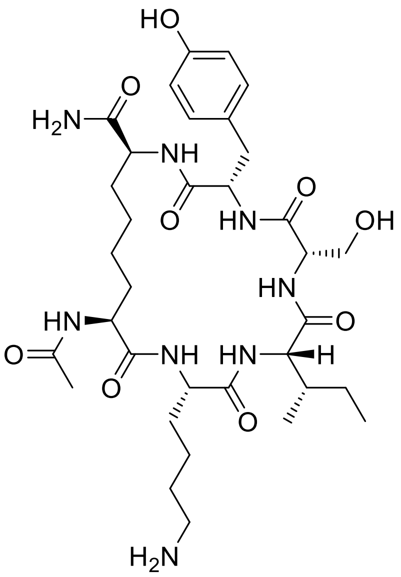

Cyclisation of peptide **28** (2 mg, 2.67 µmol) repeated using standard conditions in 10% MeCN PBS. After analysis the remaining solution (2.61 µmol) was purified using semi-preparative HPLC (2-95% B over 30 minutes) and fractions containing the desired product lyophilised to yield the title compound as a fluffy white solid (0.56 mg, 0.77 µmol, 29% yield).

Note: compared to Figure S100 (i.e., the reaction run in conjugation buffer) the ratio of product to desulfurised-linear by-product is shifted in slight favour to the by-product when running the reaction in PBS.

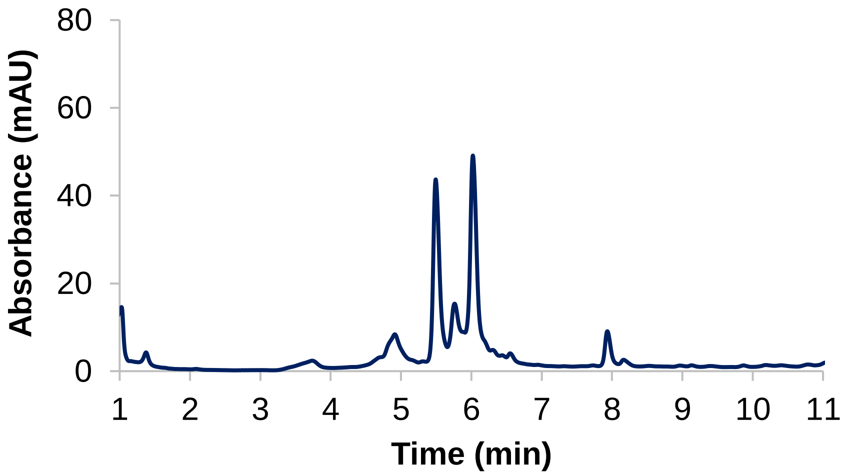

Figure S104. Crude analytical HPLC trace of cyclisation of Ac-(alG)KISYC-NH_2 (**39**) in PBS; analytical gradient 5-60% B over 10 minutes, 280 nm.

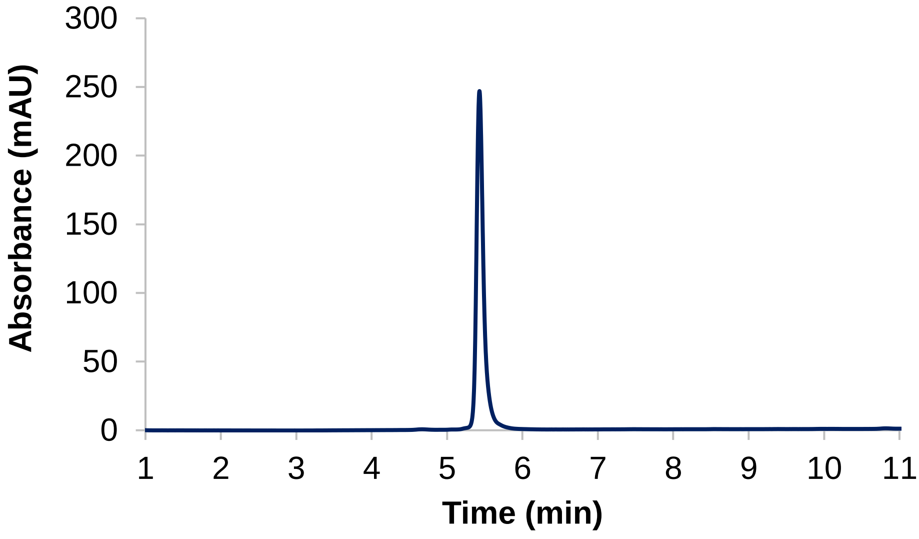

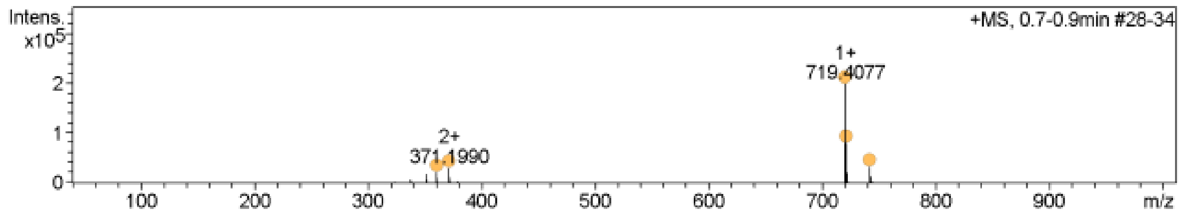

Figure S105. Analytical HPLC trace and ESI MS of purified cyclised product **39**; analytical gradient 5-60% B over 10 minutes, 280 nm. Calculated mass $[M+H]^+$: 719.41; observed mass $[M+H]^+$ 719.41.

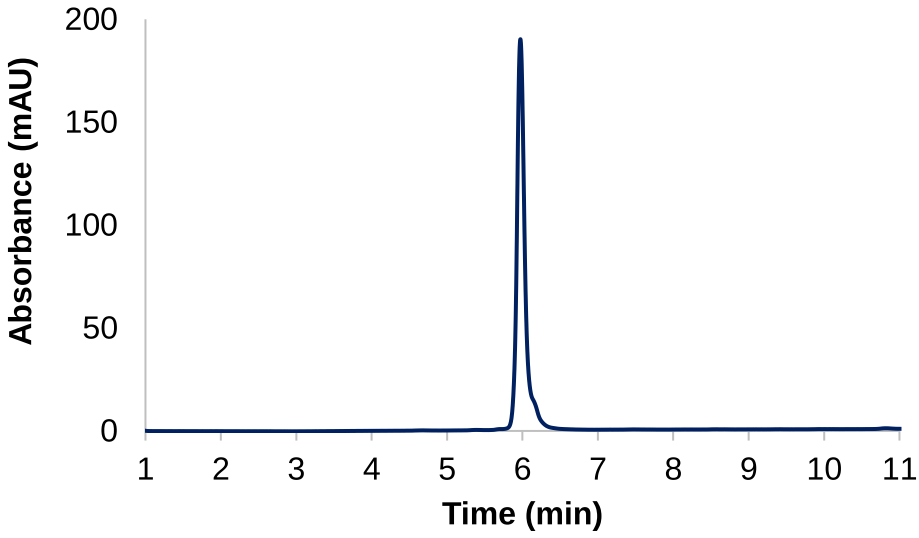

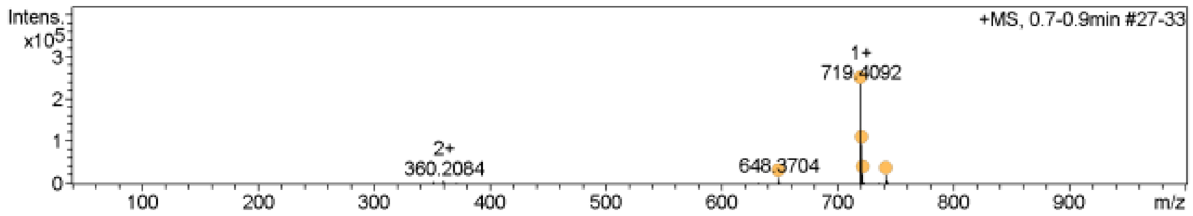

Figure S106. Analytical HPLC trace and ESI MS of purified linear desulfurised by-product from the cyclic product **39**; analytical gradient 5-60% B over 10 minutes, 280 nm. Calculated mass $[M+H]^+$: 719.41; observed mass $[M+H]^+$ 719.41.

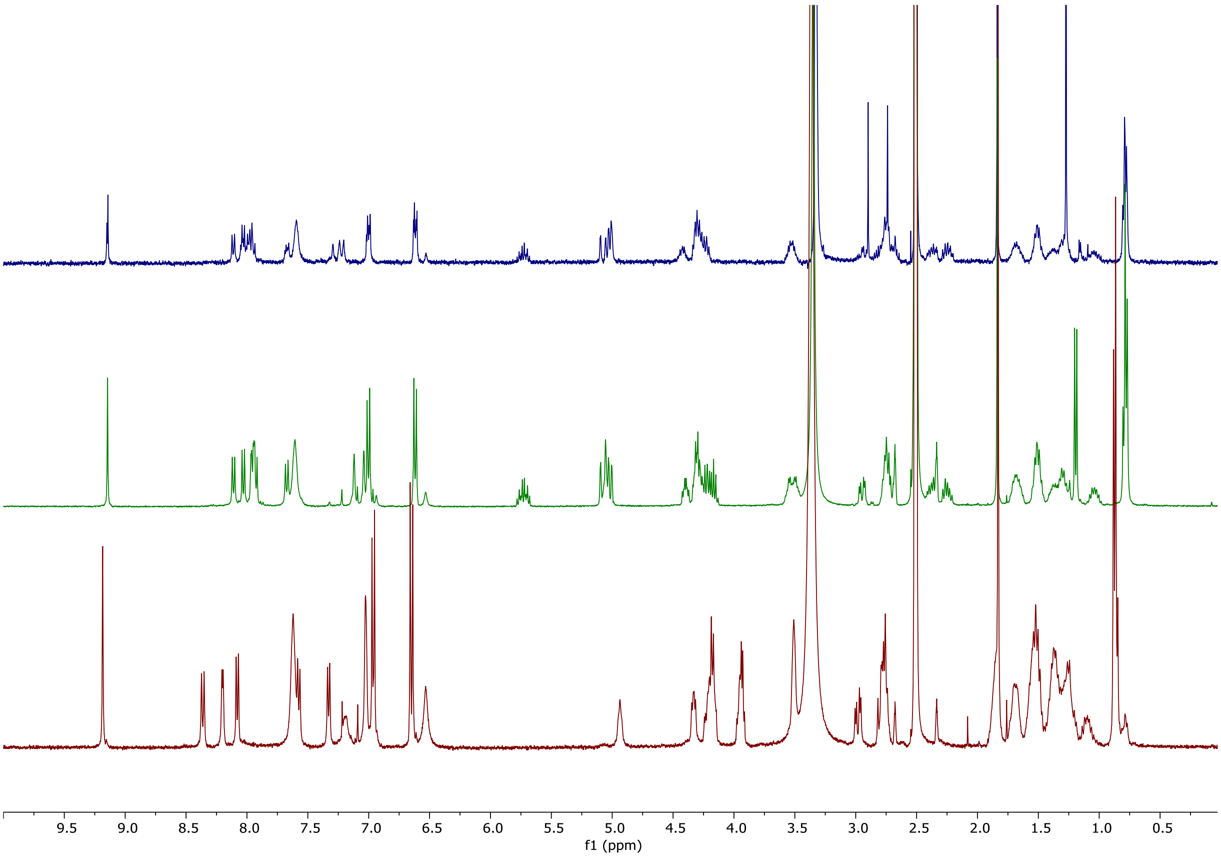

Figure S107. Top spectra – starting peptide **28**; middle spectra – desulfurised linear peptide; bottom spectra – cyclised product **39**.

Cyclisation of Ac-CKISY(alG)-NH_2 (**39**)

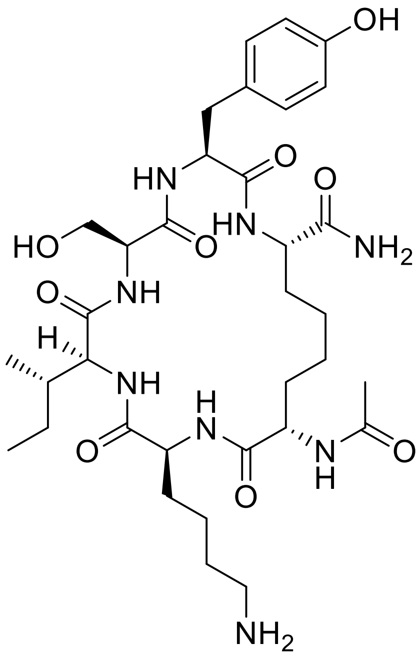

Product **39** was synthesised following the optimised cyclisation protocol using Ac-CKISY(alG)-NH_2 (**29**, 2 mg, 2.67 µmol). After analysis the remaining solution (2.61 µmol) was purified using semi-preparative HPLC (2-95% B over 30 minutes) and fractions containing the desired product lyophilised to yield the title compound as a fluffy white solid (1.2 mg, 1.67 µmol, 64% yield).

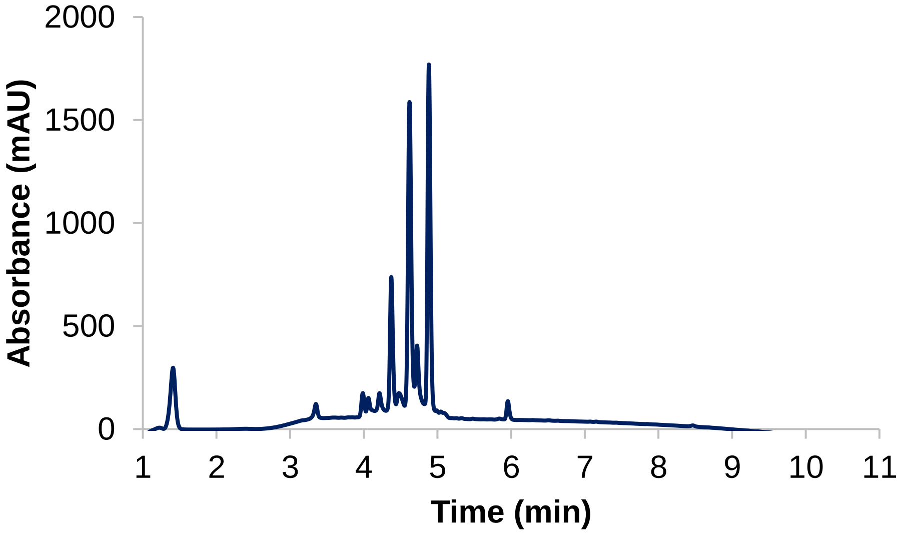

Figure S108. Crude analytical HPLC trace of Ac-CKISY(alG)-NH_2 (**39**); analytical gradient 2-95% B over 10 minutes, 210 nm.

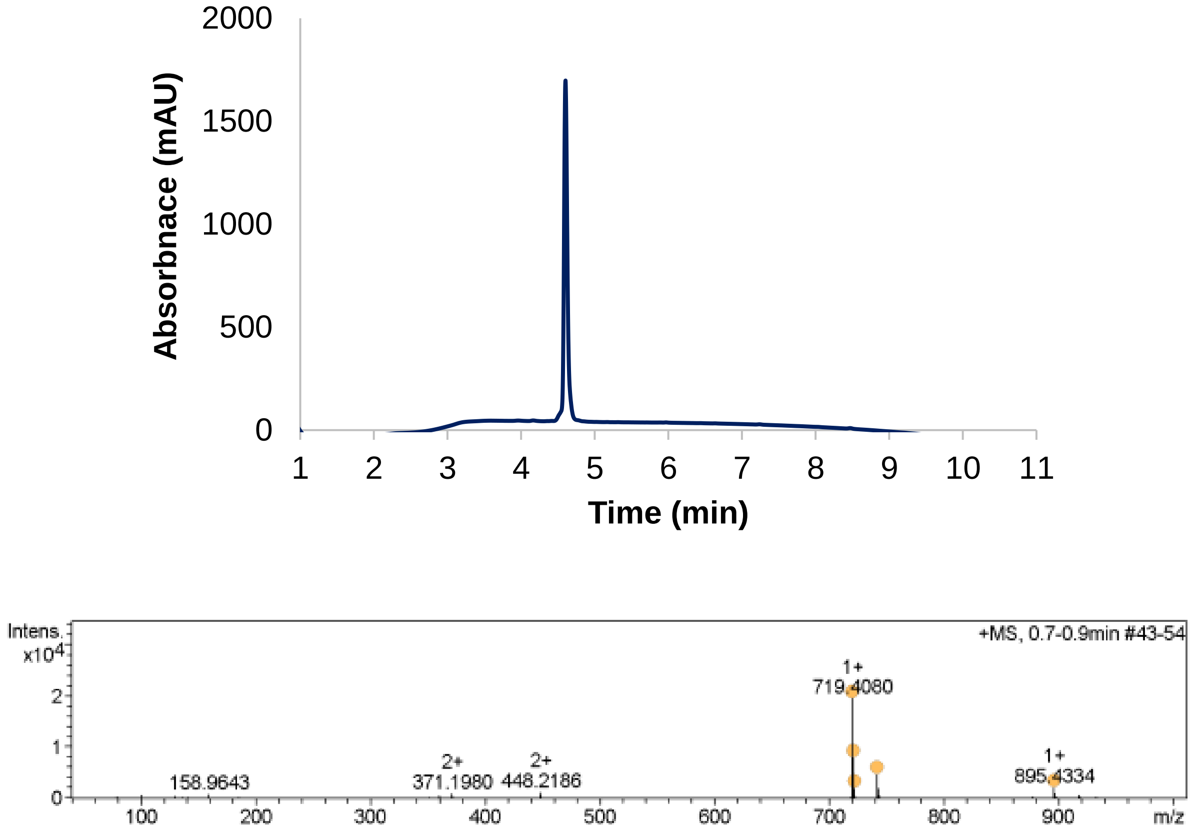

Figure S109. Analytical HPLC trace and ESI MS of purified cyclisation of Ac-CKISY(alG)-NH_2 (**39**); analytical gradient 2-95% B over 10 minutes, 210 nm. Calculated mass $[M+H]^+$: 719.40; observed mass $[M+H]^+$ 719.40.

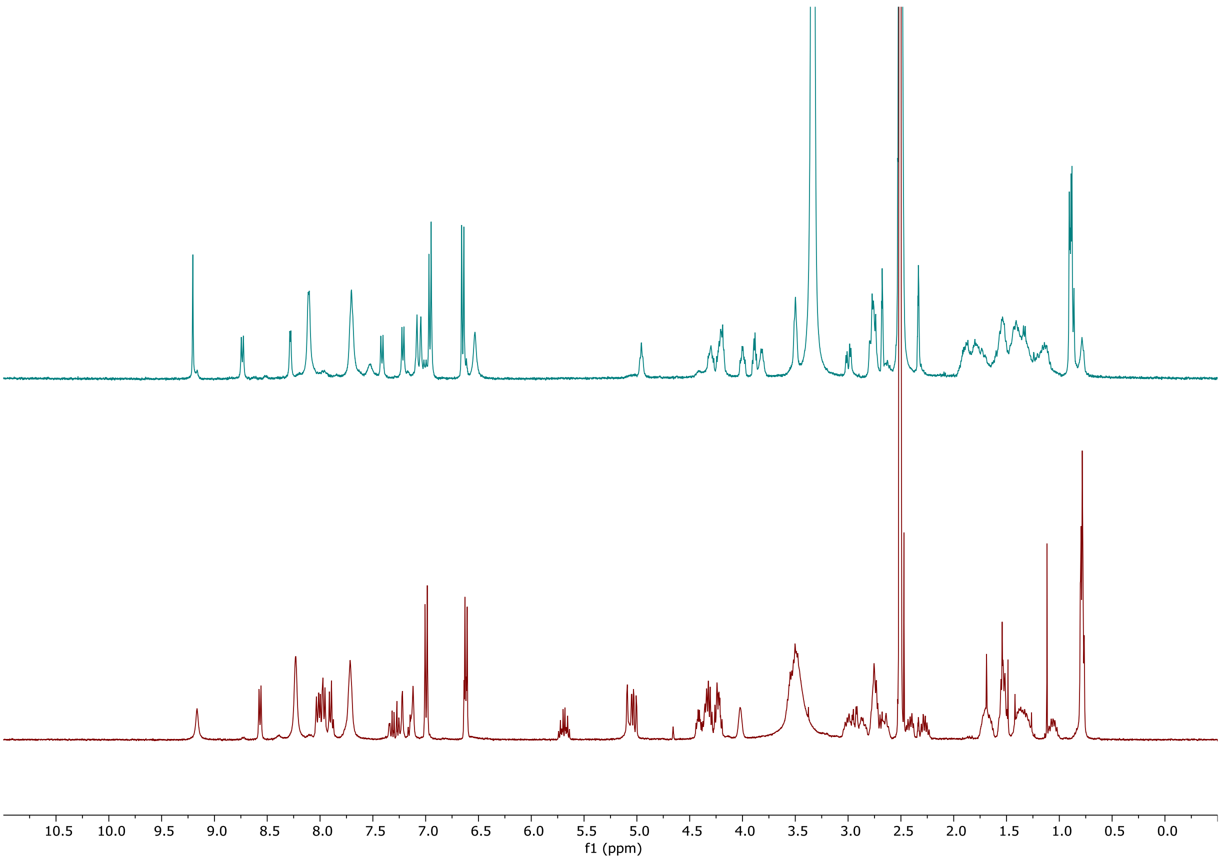

Figure S110. NMR comparison of cyclised Ac-CKISY(alG)-NH_2 (**39**, top) and the starting peptide (**29**, bottom) showing disappearance of allylic protons at 5.68 and 5.05 ppm.

Cyclisation of Ac-(alG)RPMHC-NH_2 (**40b**)

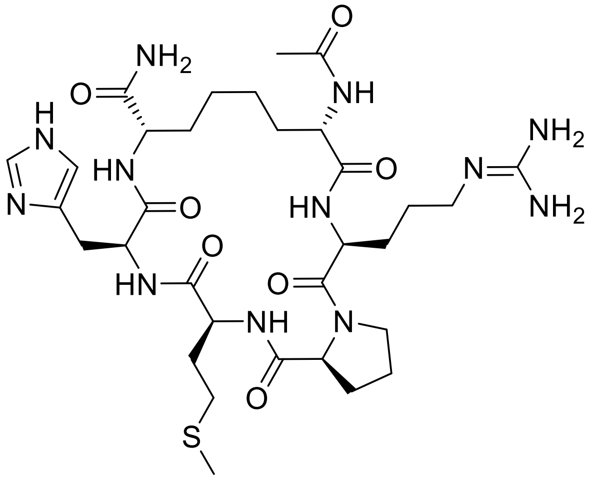

Product **40b** was synthesised following the optimised cyclisation protocol using Ac-(alG)RPMHC-NH_2 (**30b**, 2 mg, 2.56 µmol). After analysis the remaining solution (2.51 µmol) was purified using semi-preparative HPLC (2-95% B over 30 minutes) and fractions containing the desired product lyophilised to yield the title compound as a fluffy white solid (0.9 mg, 1.20 µmol, 48% yield).

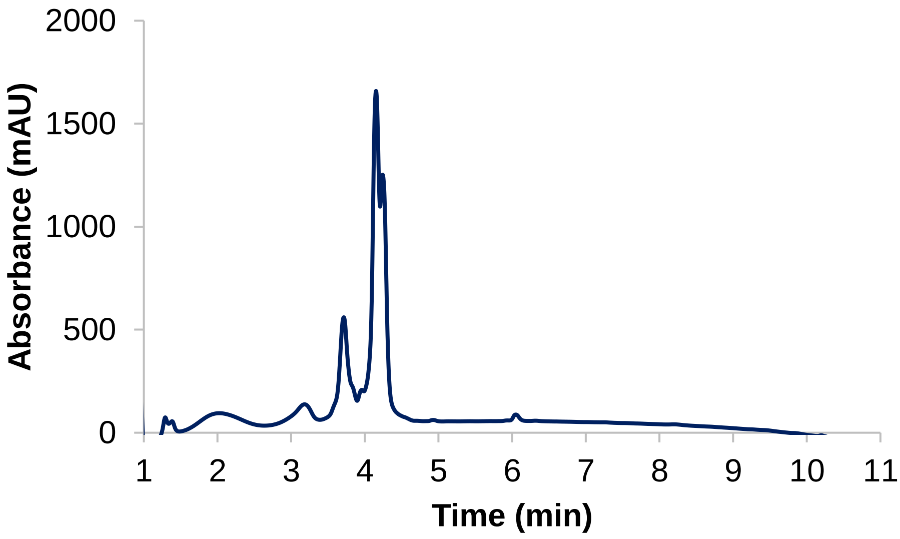

Figure S111. Crude analytical HPLC trace of Ac-(alG)RPMHC-NH_2 (**40b**); analytical gradient 2-95% B over 10 minutes, 210 nm.

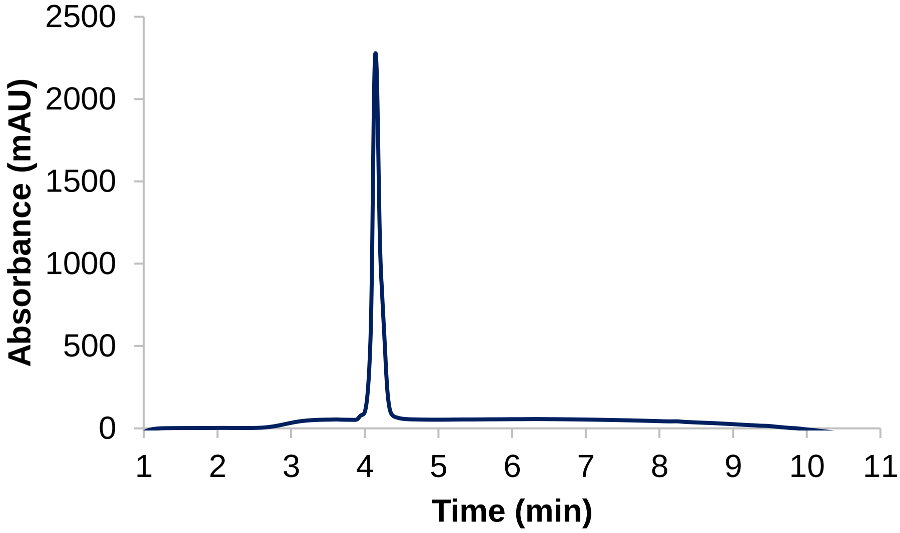

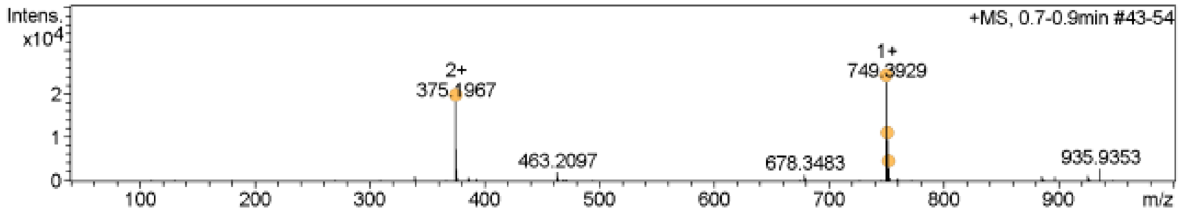

Figure S112. Analytical HPLC trace and ESI MS of purified cyclisation of Ac-(alG)RPMHC-NH_2 (**40b**); analytical gradient 2-95% B over 10 minutes, 210 nm. Calculated mass $[M+H]^+$: 749.39; observed mass $[M+H]^+$ 749.39.

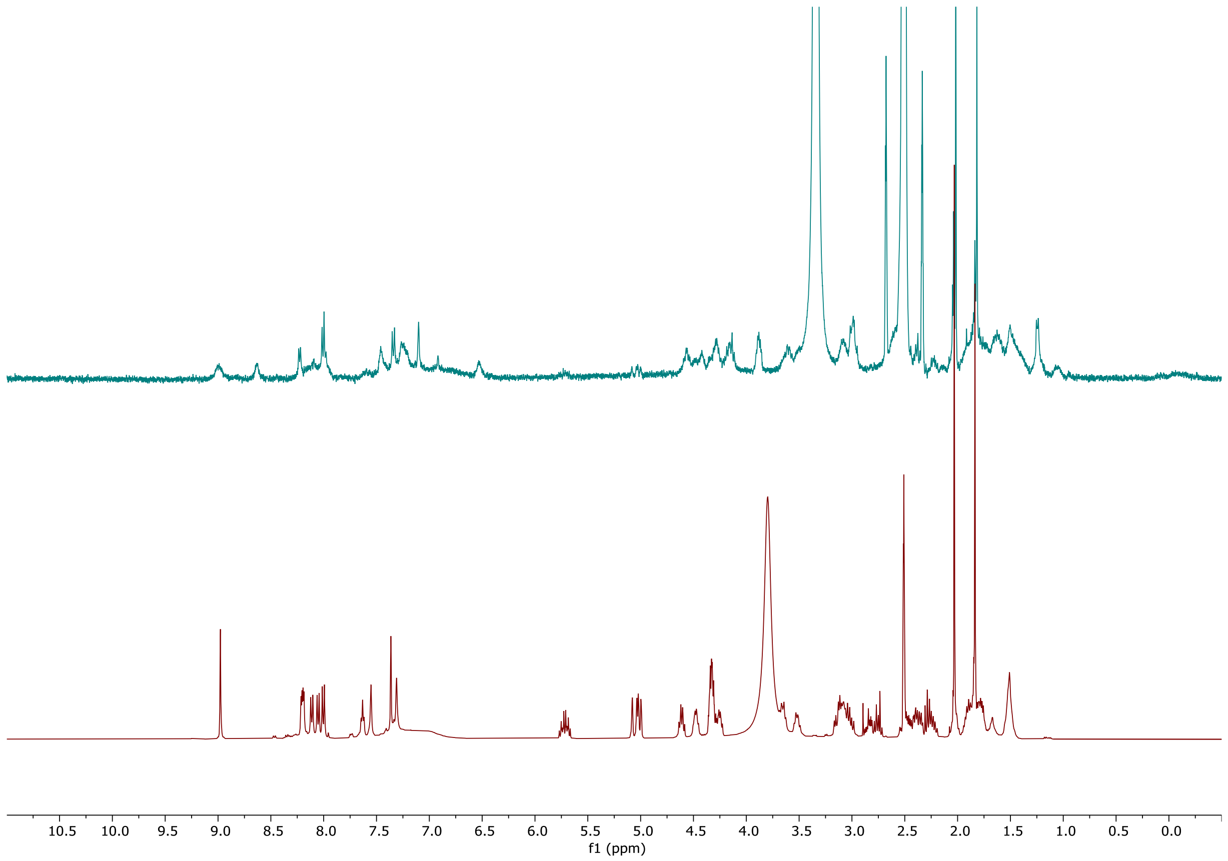

Figure S113. NMR comparison of cyclised Ac-(alG)RPMHC-NH_2 (**40b**, top) and the starting peptide (**30b**, bottom) showing disappearance of allylic protons at 5.71 and 5.04 ppm.

Cyclisation of Ac-(alG)ELQWC-NH2 (**41**)

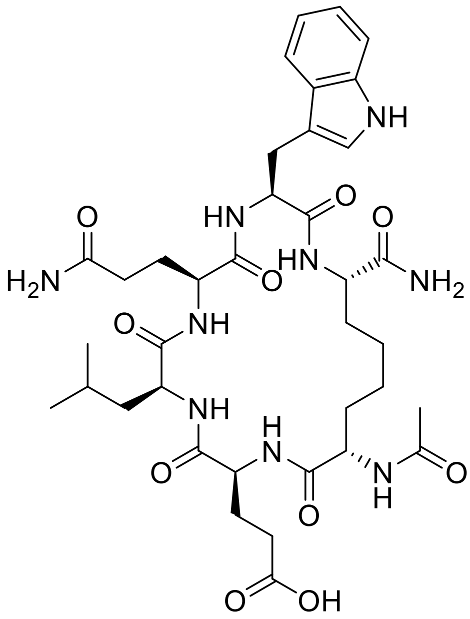

Product **41** was synthesised following the optimised cyclisation protocol using Ac-(alG)ELQWC-NH2 (**31**, 2 mg, 2.45 µmol). After analysis the remaining solution (2.40 µmol) was purified using semi-preparative HPLC (2-95% B over 30 minutes) and fractions containing the desired product lyophilised to yield the title compound as a fluffy white solid (0.7 mg, 0.89 µmol, 37% yield).

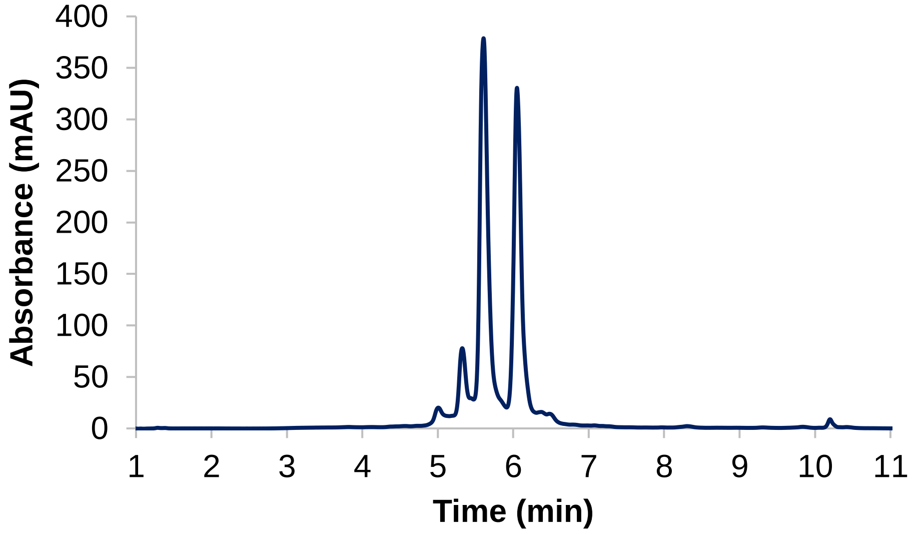

Figure S114. Crude analytical HPLC trace of Ac-(alG)ELQWC-NH2 (**41**); analytical gradient 2-95% B over 10 minutes, 280 nm.

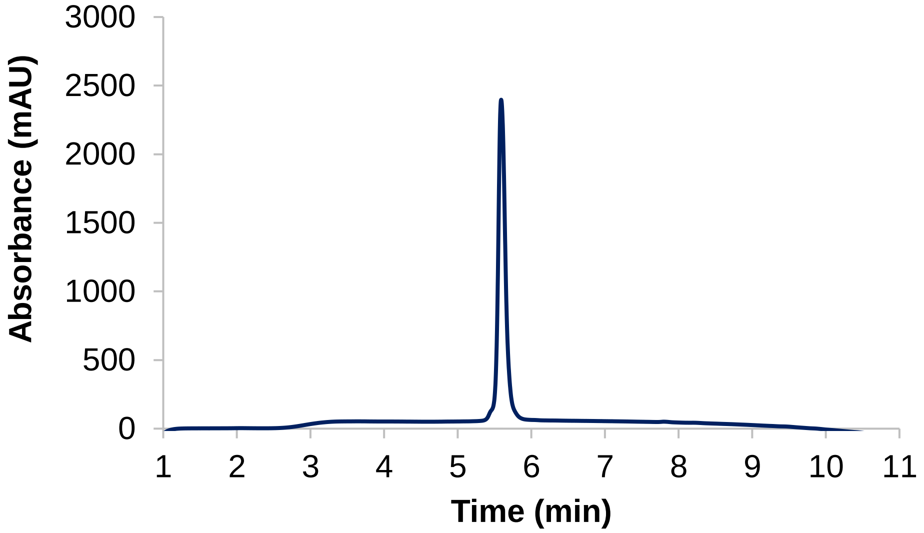

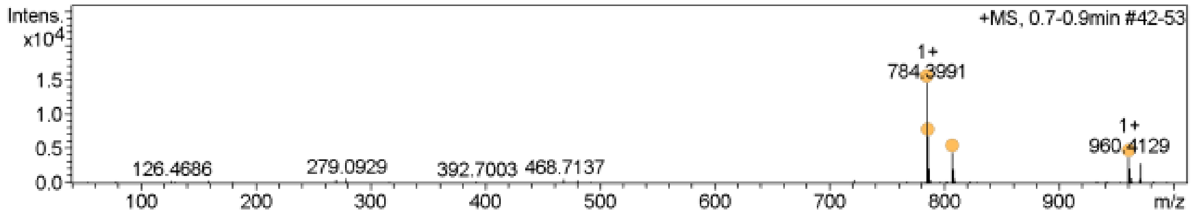

Figure S115. Analytical HPLC trace and ESI MS of purified cyclisation of Ac-(alG)ELQWC-NH_2 (**41**); analytical gradient 2-95% B over 10 minutes, 210 nm. Calculated mass $[M+H]^+$: 784.39; observed mass $[M+H]^+$ 784.39.

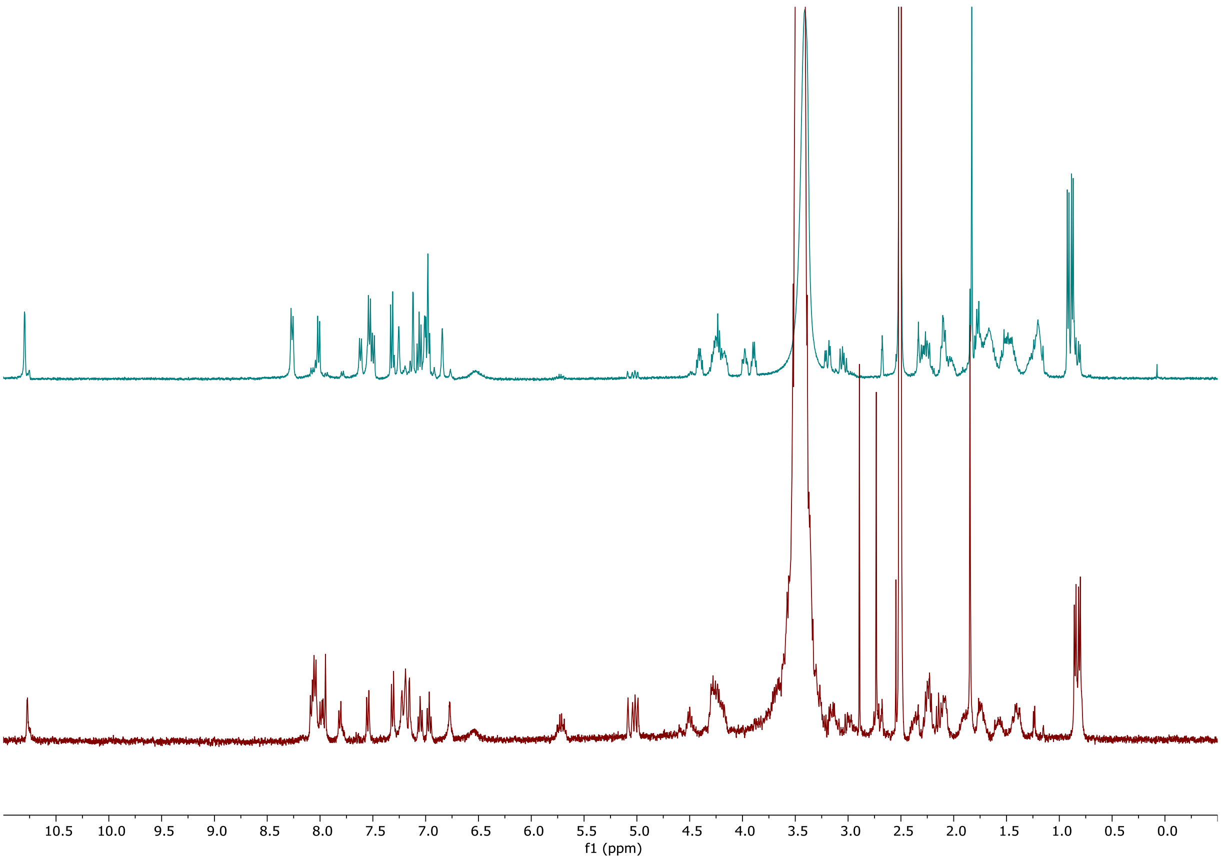

Figure S116. NMR comparison of cyclised Ac-(alG)ELQWC-NH_2 (**41**, top) and the starting peptide (**31**, bottom) showing disappearance of allylic protons at 5.71 and 5.04 ppm.

Cyclisation of Ac-(alG)ELQWC-NH2 (**41**) using PTA in place of TCEP

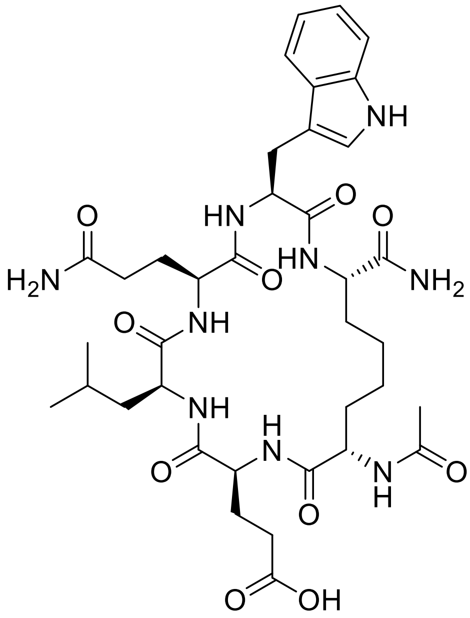

Product **41** was synthesised following the optimised cyclisation protocol using Ac-(alG)ELQWC-NH2 (**31**, 2.5 mg, 3.06 µmol) and PTA as the phosphine additive. After analysis the remaining solution was purified using semi-preparative HPLC (15-80% B over 30 minutes) and fractions containing the desired product lyophilised to yield the title compound as a fluffy white solid (1.2 mg, 1.55 µmol, 52% yield).

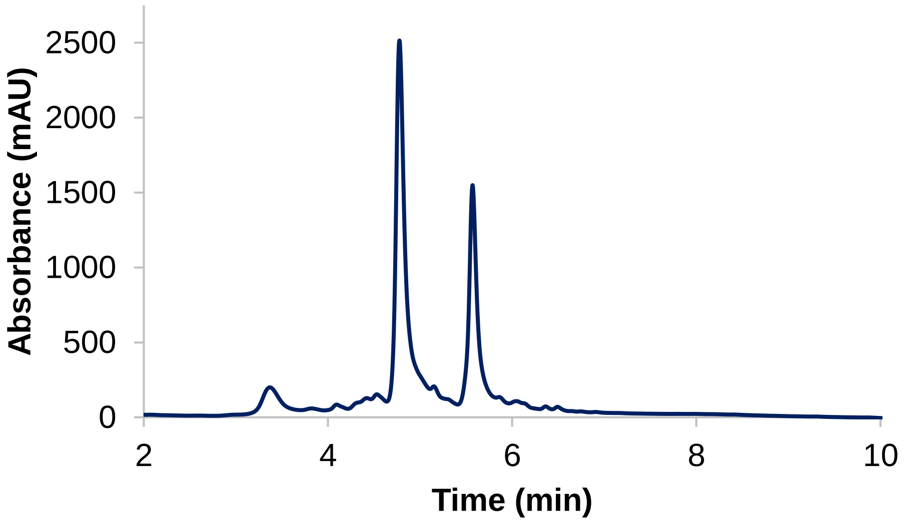

Figure S117. Crude analytical HPLC trace of Ac-(alG)ELQWC-NH2 (**41**) using PTA as the phosphine additive; analytical gradient 15-80% B over 10 minutes, 280 nm.

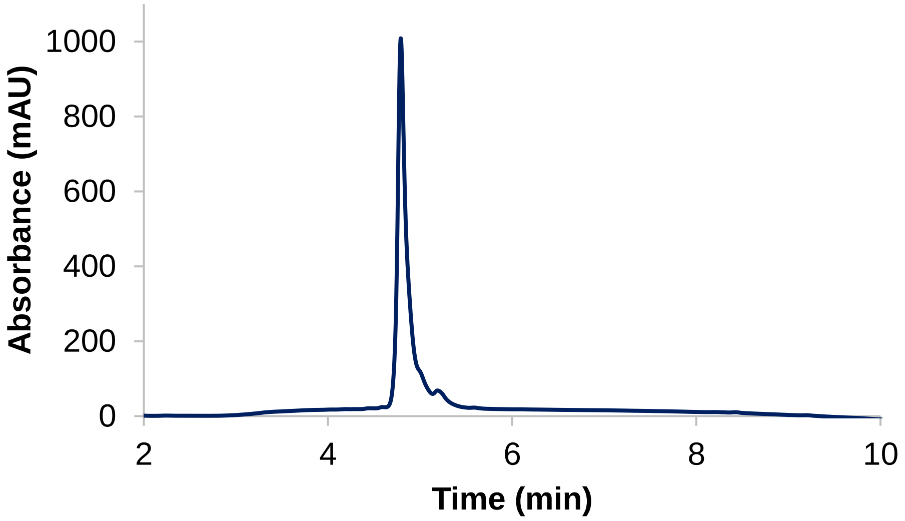

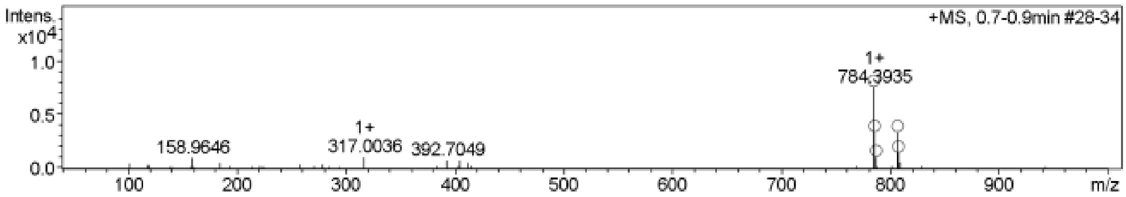

Figure S118. Analytical HPLC trace and ESI MS of purified cyclisation of Ac-(alG)ELQWC-NH_2 (**41**); analytical gradient 15-80% B over 10 minutes, 210 nm. Calculated mass $[M+H]^+$: 784.39; observed mass $[M+H]^+$ 784.39.

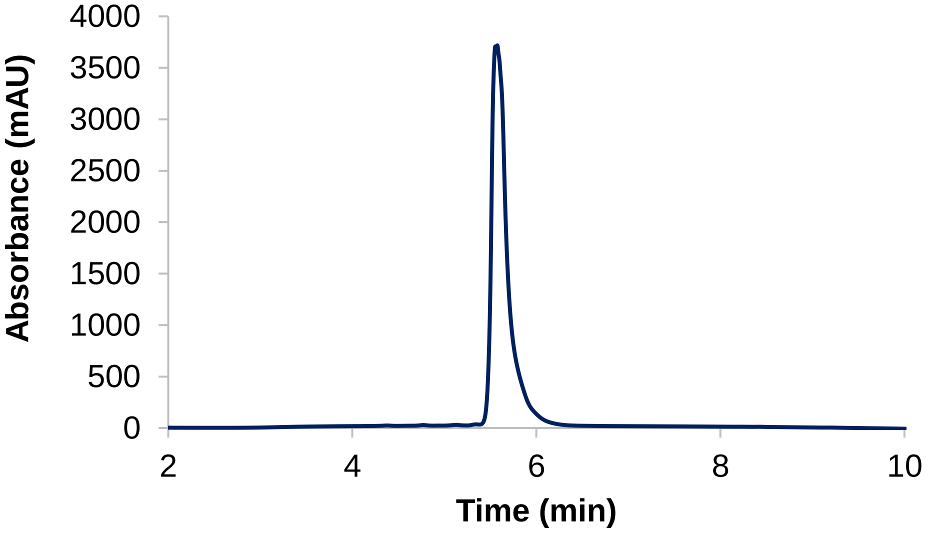

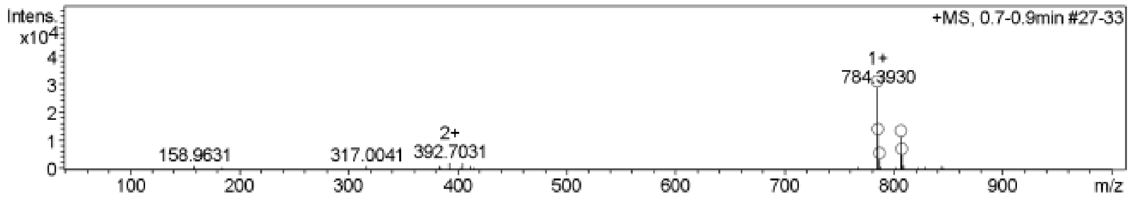

Figure S119. Analytical HPLC trace and ESI MS of purified linear desulfurised by-product Ac-(alG)ELQWA-NH_2; analytical gradient 15-80% B over 10 minutes, 210 nm. Calculated mass $[M+H]^+$: 784.39; observed mass $[M+H]^+$ 784.39.

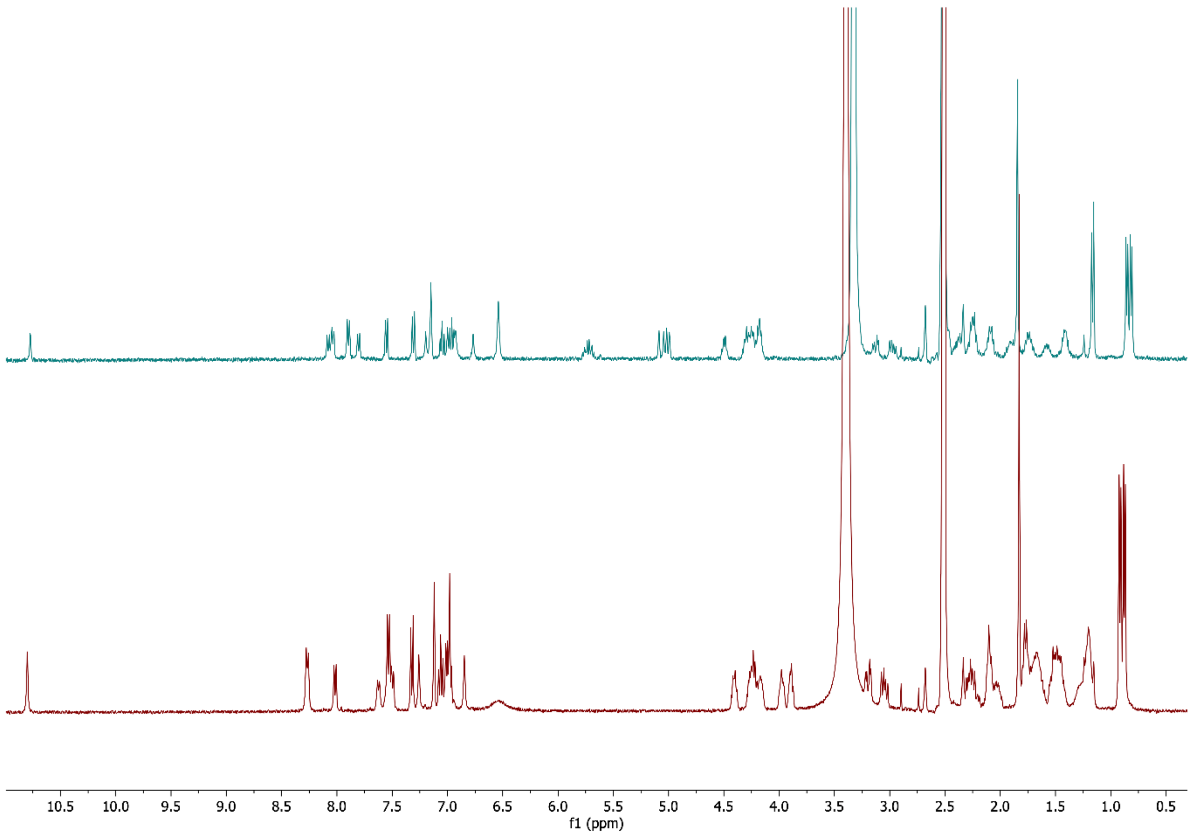

Figure S120. ^{1}H NMR comparison of the isolated linear desulfurised by-product (top) and cyclised Ac-(alG)ELQWC-NH_2 (**41**, bottom) showing disappearance of allylic protons at 5.71 and 5.04 ppm.

Cyclisation of Ac-(alG)DSYKIC-NH_2 (**42**)

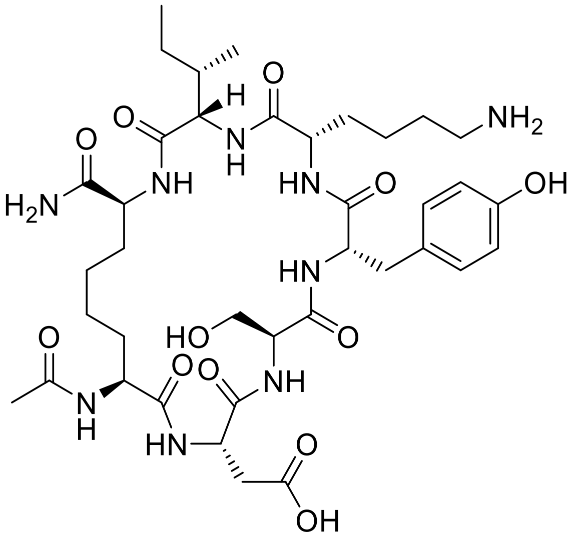

Product **42** was synthesised following the optimised cyclisation protocol using Ac-(alG)DSYKIC-NH_2 (**32**, 2 mg, 2.31 µmol). After analysis the remaining solution (2.26 µmol) was purified using semi-preparative HPLC (5-60% B over 30 minutes) and fractions containing the desired product lyophilised to yield the title compound as a fluffy white solid (1.1 mg, 1.32 µmol, 58% yield).

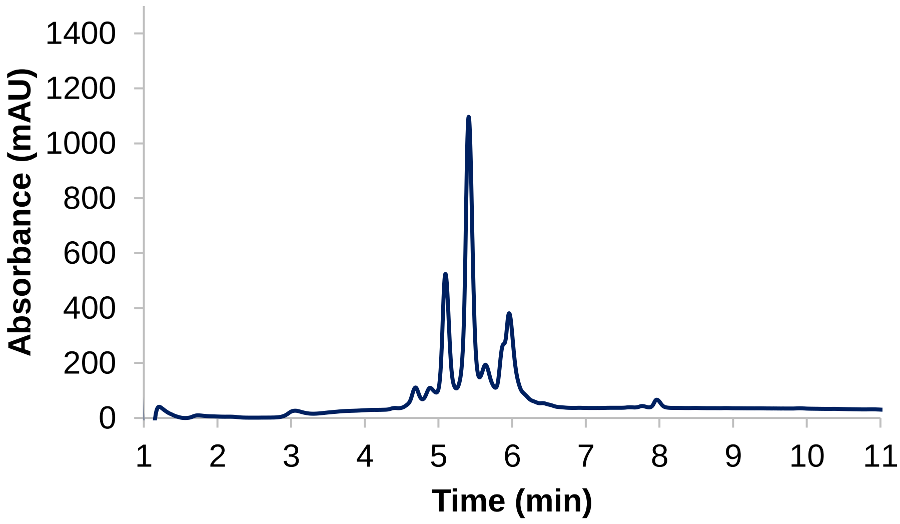

Figure S121. Crude analytical HPLC trace of Ac-(alG)DSYKIC-NH_2 (**42**); analytical gradient 5-60% B over 10 minutes, 210 nm.

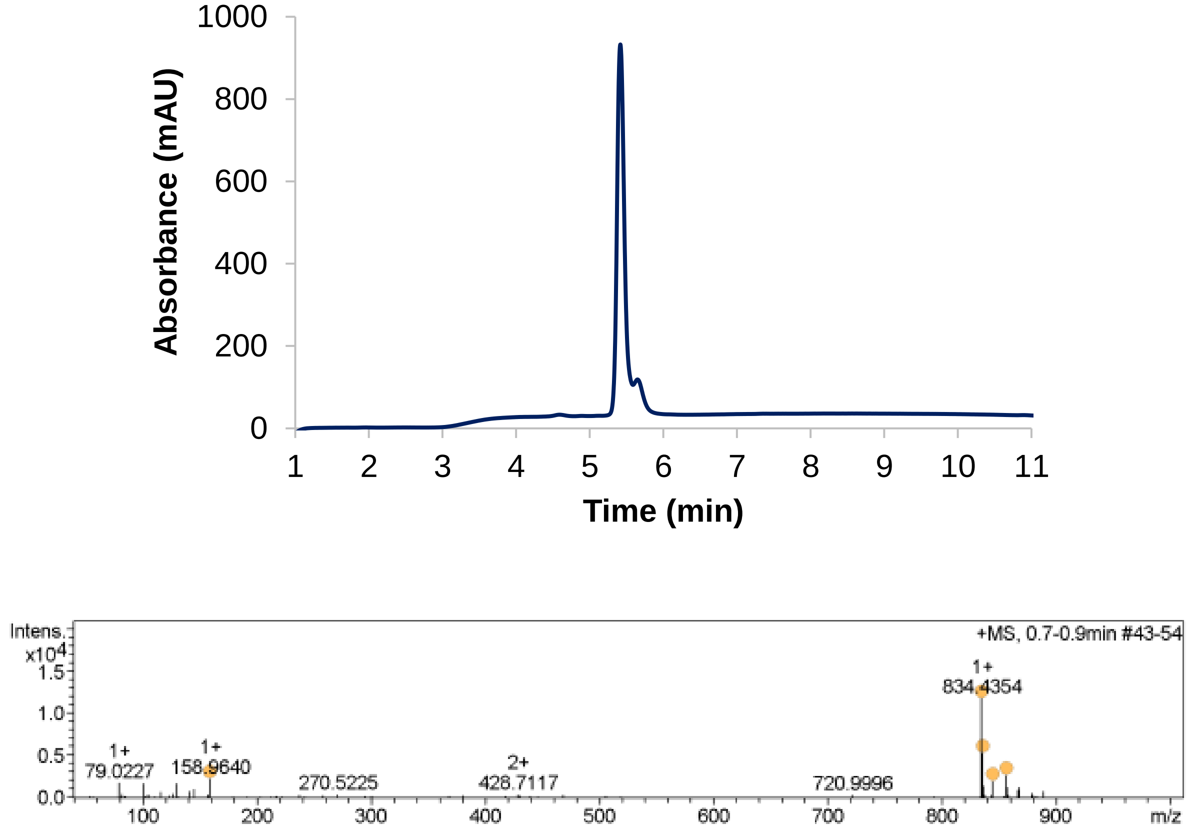

Figure S122. Analytical HPLC trace and ESI MS of purified cyclisation of Ac-(alG)DSYKIC-NH_2 (**42**); analytical gradient 5-60% B over 10 minutes, 210 nm. Calculated mass $[M+H]^+$: 834.43; observed mass $[M+H]^+$ 834.43.

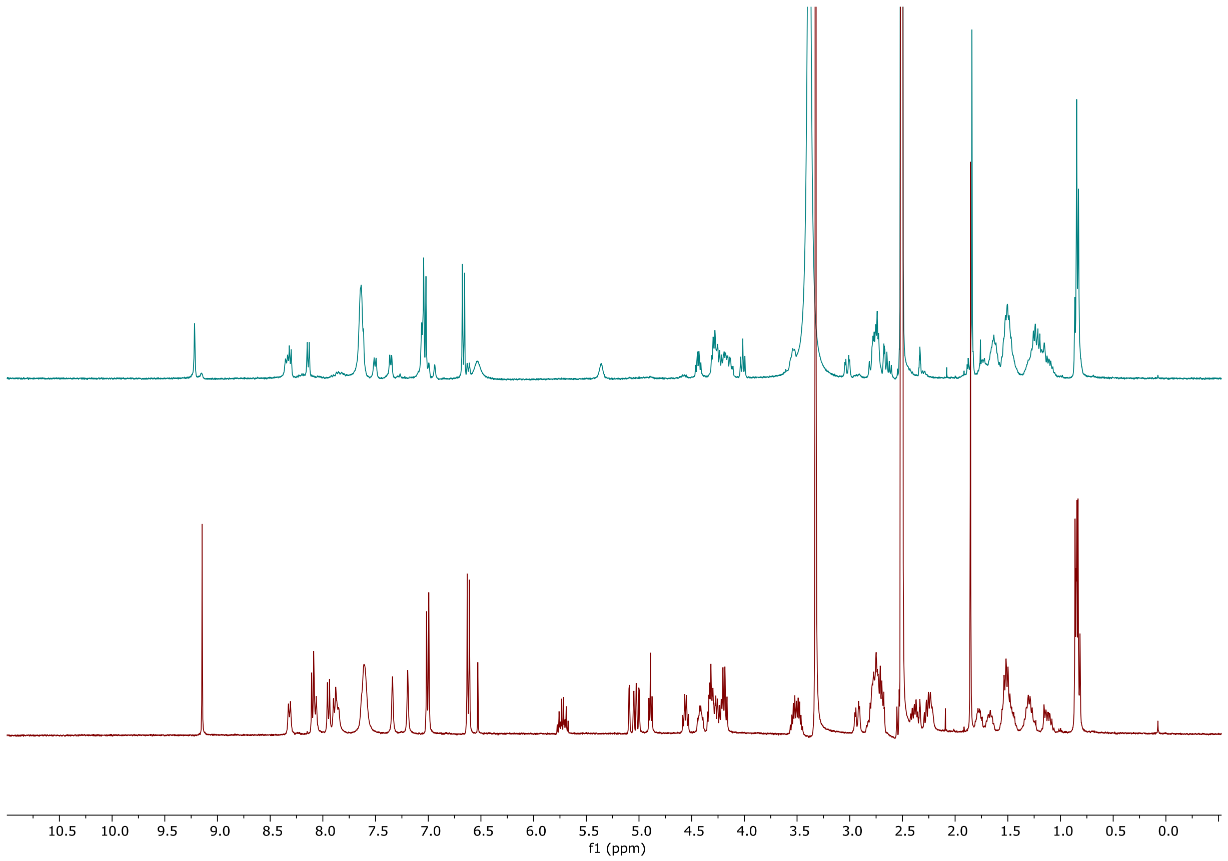

Figure S123. NMR comparison of cyclised Ac-(alG)DSYKIC-NH_2 (**42**, top) and the starting peptide (**32**, bottom) showing disappearance of allylic protons at 5.70 and 5.05 ppm.

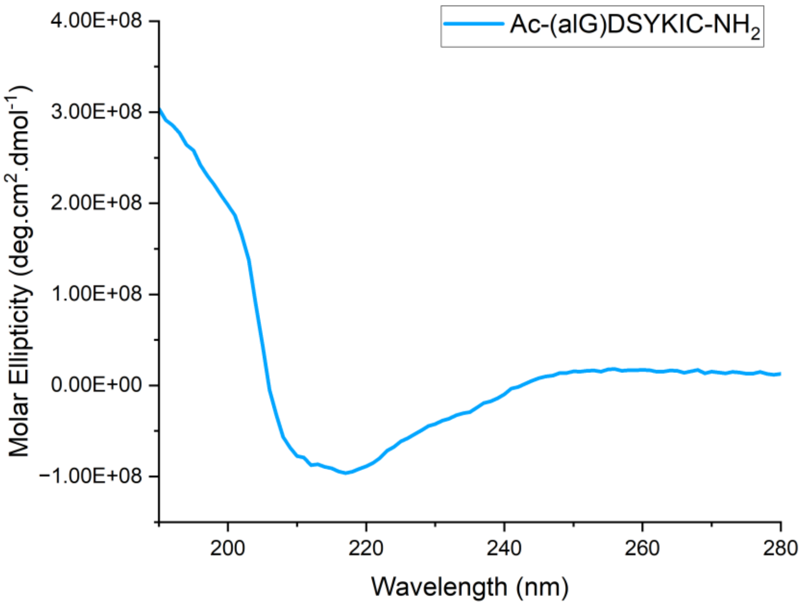

Figure S124. Circular dichroism for cyclised product **42**.

Cyclisation of Ac-(alG)NHPRVC-NH_2 (**43**)

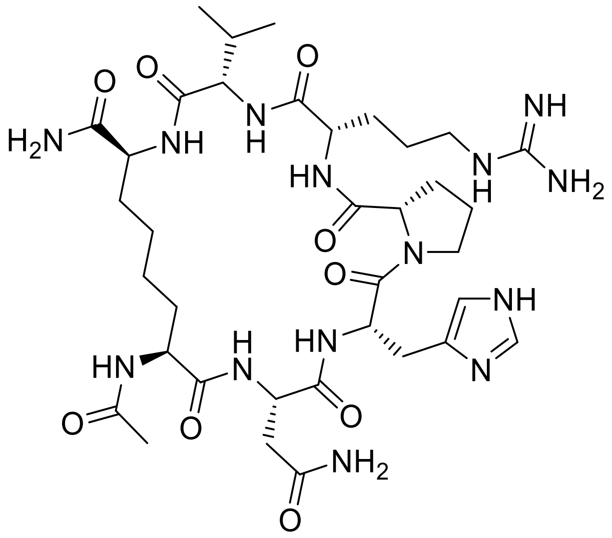

Product **43** was synthesised following the optimised cyclisation protocol using Ac-(alG)NHPRVC-NH_2 (**33**, 2 mg, 2.41 µmol). After analysis the remaining solution (2.27 µmol) was purified using semi-preparative HPLC (5-60% B over 30 minutes) and fractions containing the desired product lyophilised to yield the title compound as a fluffy white solid (0.6 mg, 0.723 µmol, 32% yield).

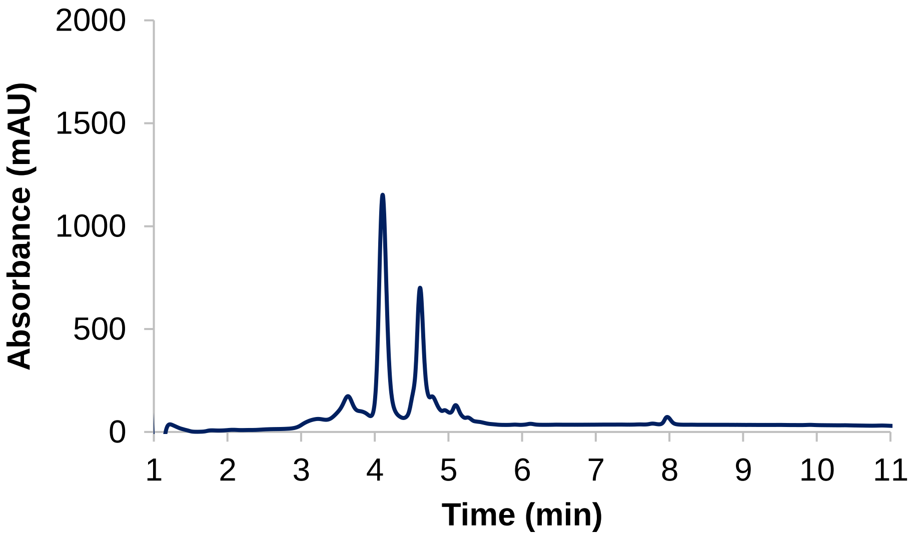

Figure S125. Crude analytical HPLC trace of Ac-(alG)NHPRVC-NH_2 (**43**); analytical gradient 5-60% B over 10 minutes, 210 nm.

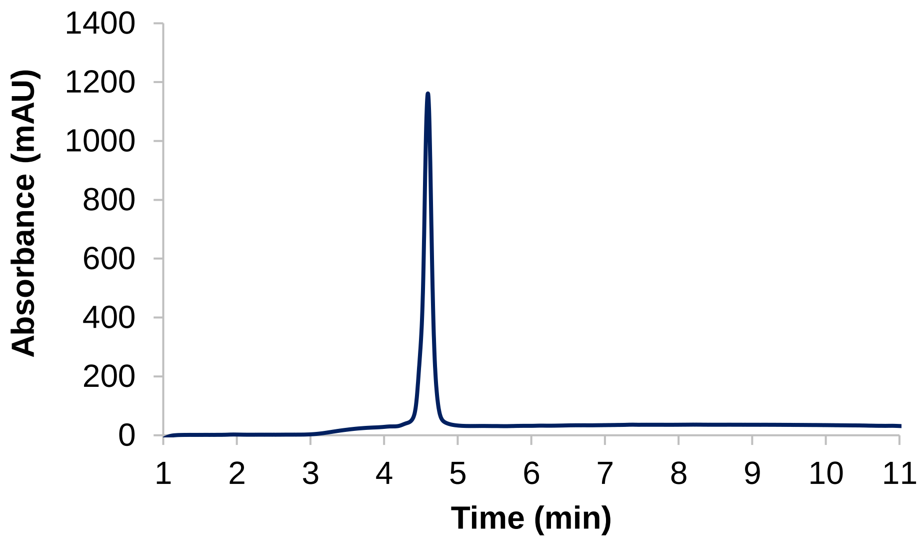

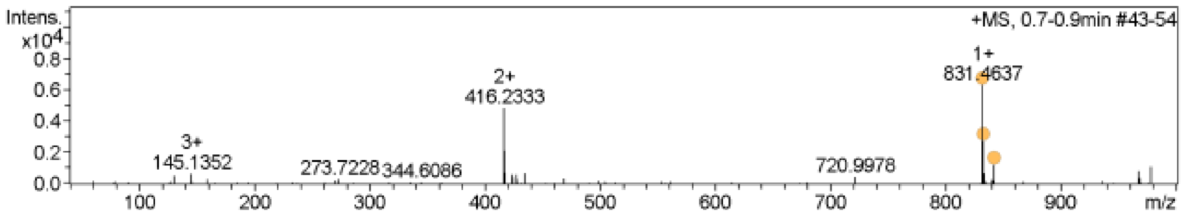

Figure S126. Analytical HPLC trace and ESI MS of purified cyclisation of Ac-(alG)NHPRVC-NH_2 (**43**); analytical gradient 5-60% B over 10 minutes, 210 nm. Calculated mass $[M+H]^+$: 831.46; observed mass $[M+H]^+$ 831.46.

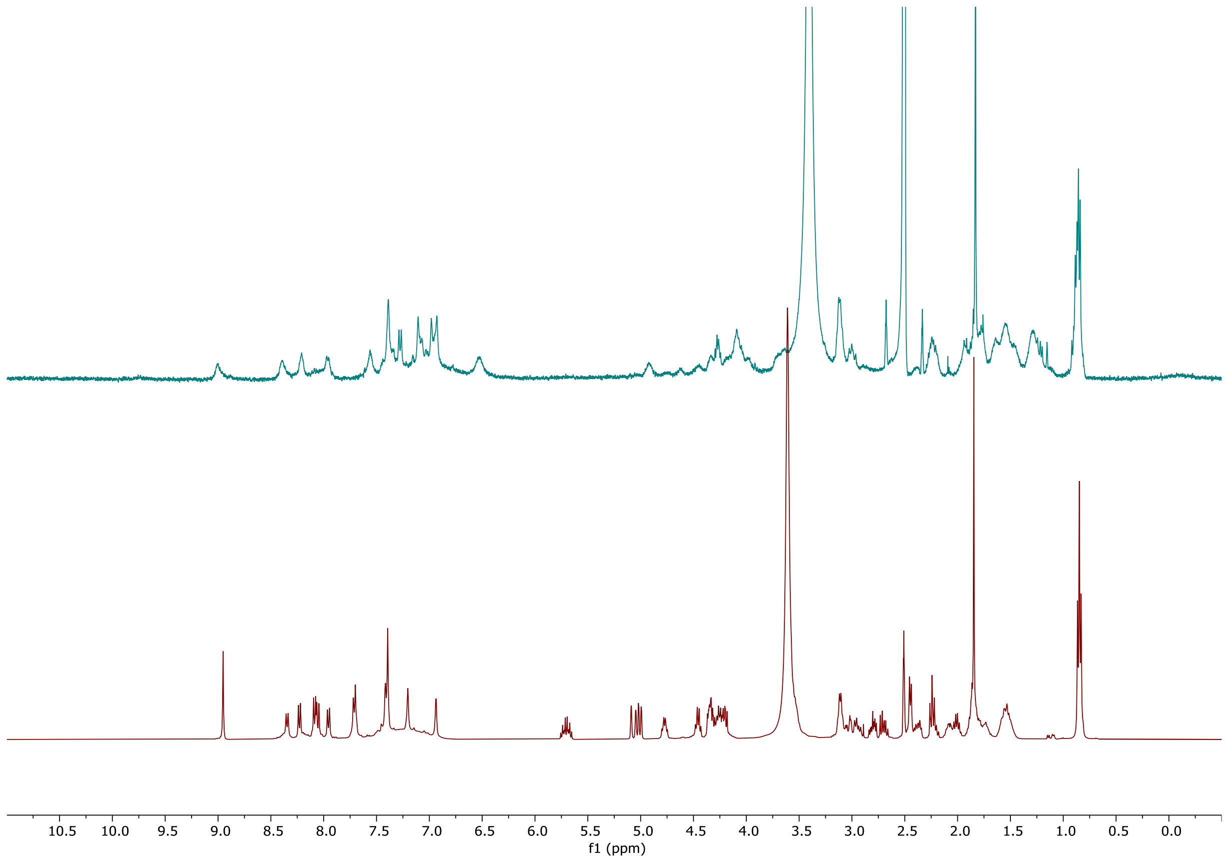

Figure S127. NMR comparison of cyclised Ac-(alG)NHPRVC-NH_2 (**43**, top) and the starting peptide (**33**, bottom) showing disappearance of allylic protons at 5.70 and 5.05 ppm.

Cyclisation of H-(alG)MALEKC-NH2 (**44**)

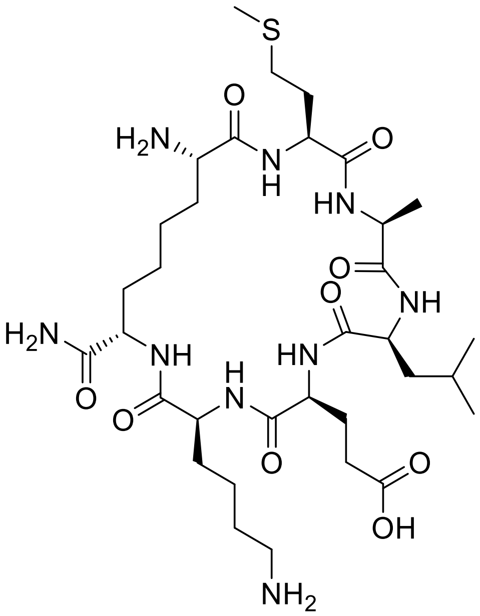

Product **44** was synthesised following the optimised cyclisation protocol using H-(alG)MALEKC-NH2 (**34**, 2 mg, 2.53 µmol). After analysis the remaining solution was purified using semi-preparative HPLC (2-95% B over 30 minutes) and fractions containing the desired product lyophilised to yield the title compound (0.85 mg, 1.12 µmol, 44%).

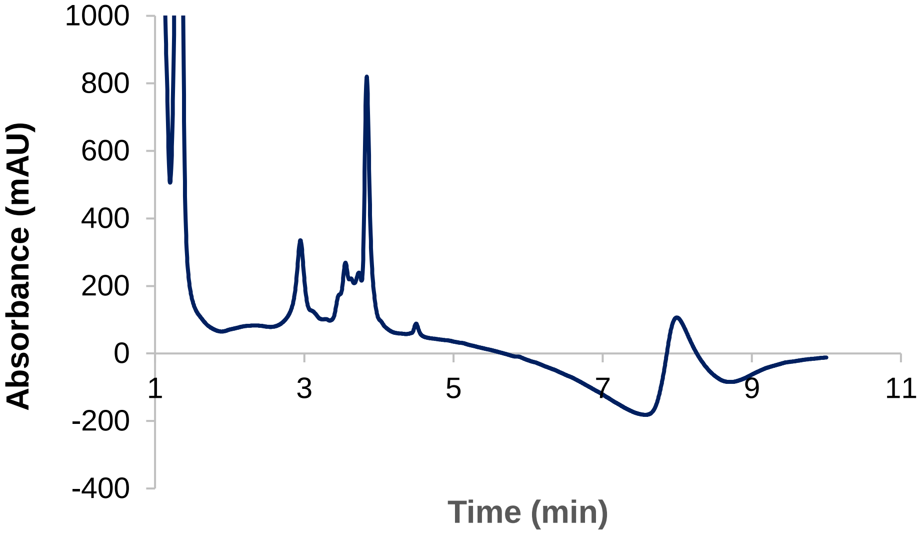

Figure S128. Crude analytical HPLC trace of Ac-(alG)MALEKC-NH2 (**44**); analytical gradient 2-95% B over 5 minutes, 210 nm.

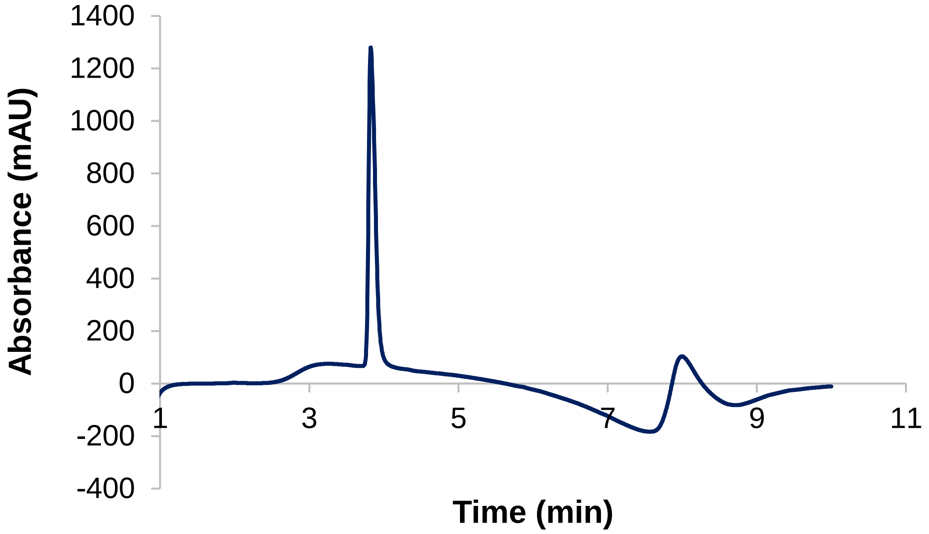

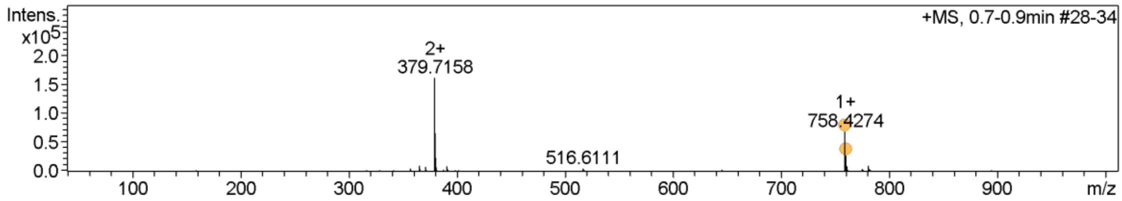

Figure S129. Analytical HPLC trace and ESI MS of purified cyclisation of Ac-(alG)MALEKC-NH_2 (**44**); analytical gradient 2-95% B over 5 minutes, 210 nm. Calculated mass $[M+H]^+$: 758.42; observed mass $[M+H]^+$ 758.43.

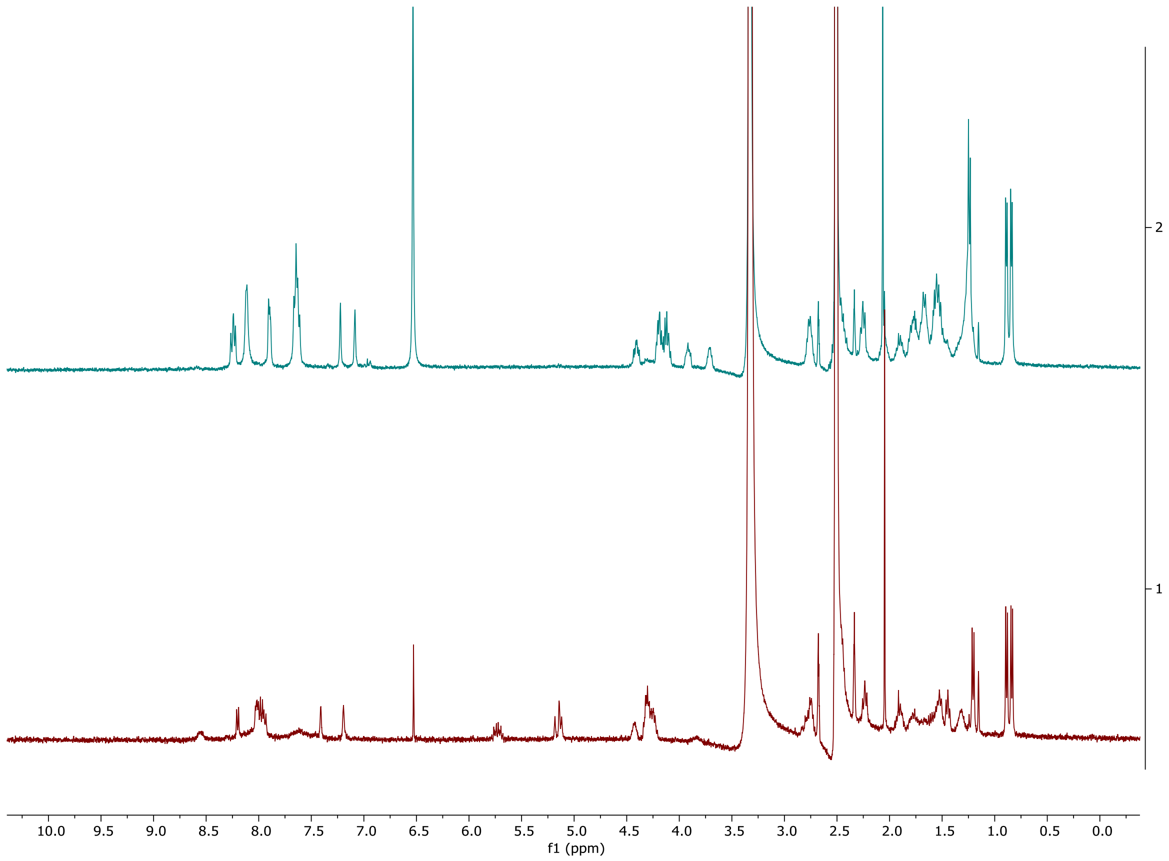

Figure S130. NMR comparison of cyclised Ac-(alG)MALEC-NH_2 (**44**, top) and the starting peptide (**34**, bottom) showing disappearance of allylic protons at 5.70 and 5.05 ppm.

Cyclisation of H-(alG)MPWLRC-NH2 (**45**)

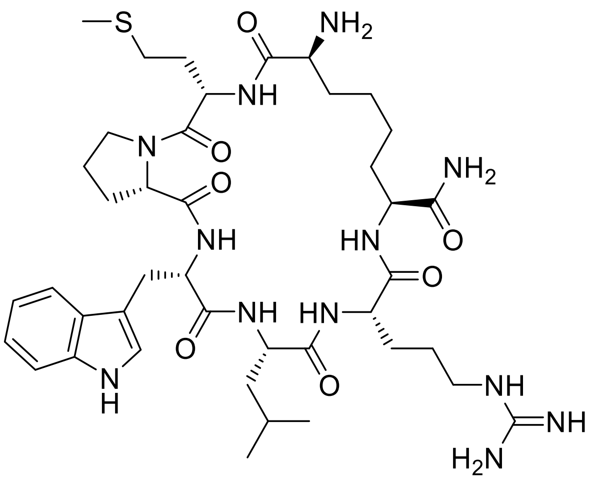

Product **45** was synthesised following the optimised cyclisation protocol using H-(alG)MPWLRC-NH2 (**35**, 2 mg, 2.22 µmol). After analysis the remaining solution (2.12 µmol) was purified using semi-preparative HPLC (10-70% B over 30 minutes) and fractions containing the desired product lyophilised to yield the title compound as a fluffy white solid (1.2 mg, 1.38 µmol, 65% yield).

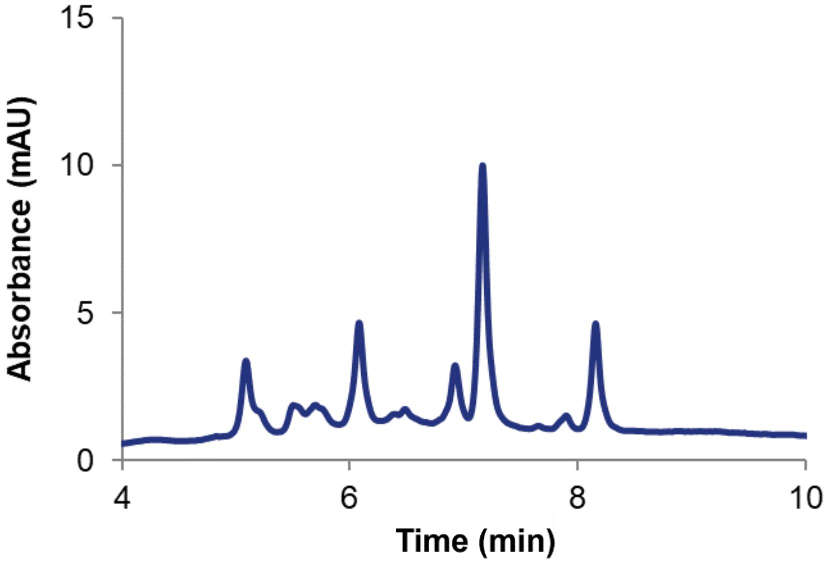

Figure S131. Crude analytical HPLC trace of H-(alG)MPWLRC-NH2 (**45**) cyclisation; analytical gradient 10-50% B over 10 minutes, 210 nm.

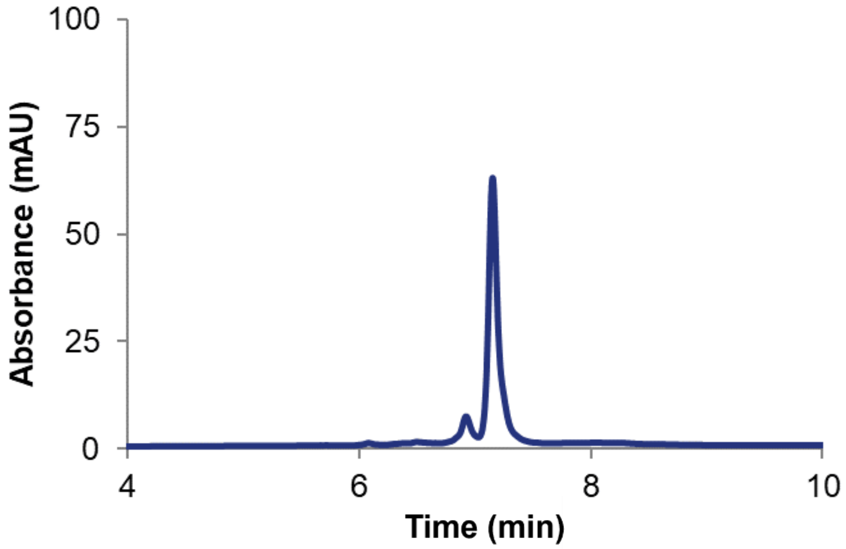

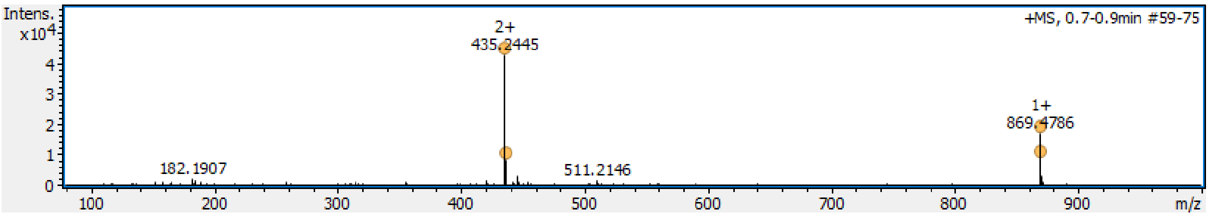

Figure S132. Analytical HPLC trace and ESI MS of cyclised H-(alG)MPWLRC-NH_2 (**45**); analytical gradient 10-50% B over 10 minutes, 210 nm. Calculated mass $[M+H]^+$: 869.48, $[M+2H]^{2+}$: 435.24; observed mass $[M+H]^+$: 869.48, $[M+2H]^{2+}$: 435.24.

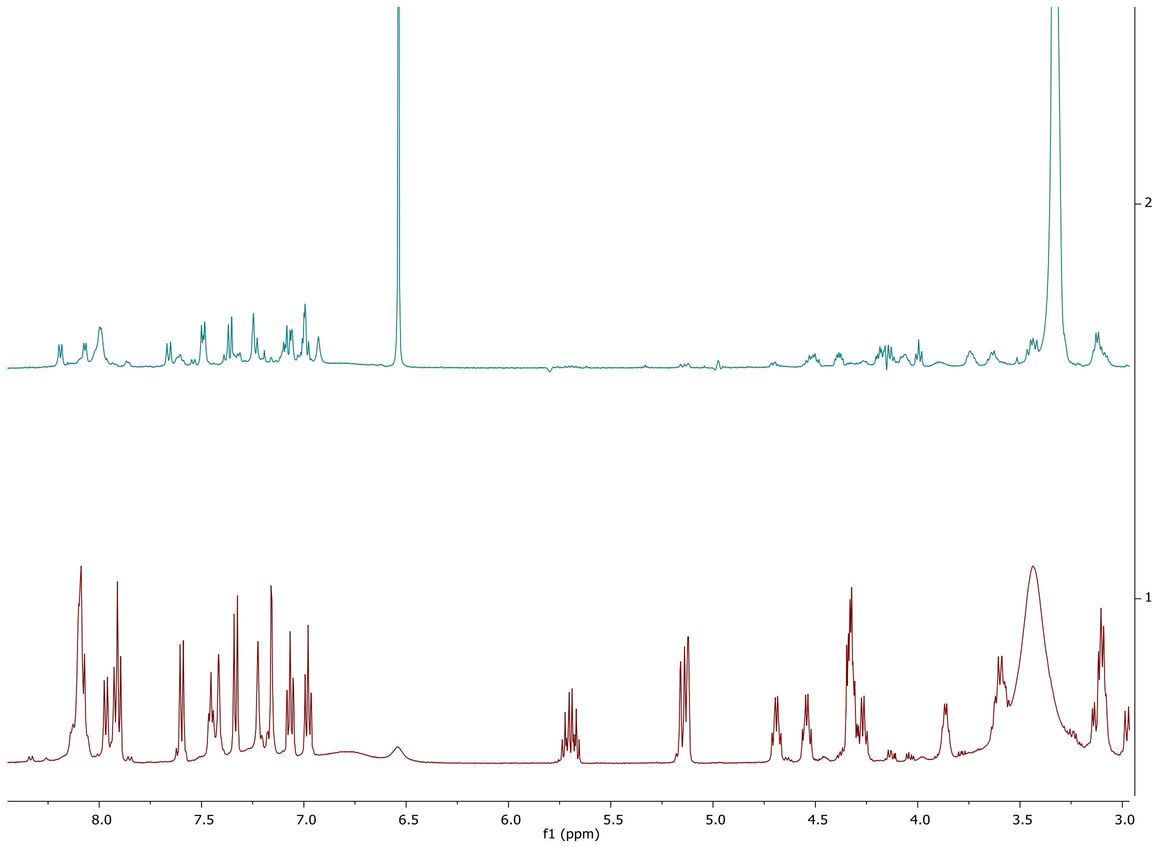

Figure S133. NMR overlay comparing model starting peptide H-(alG)MPWLRC-NH_2 (**35**, bottom) and the cyclised product (**45**, top), showing disappearance of allylic proton peaks at 5.75 and 5.20 ppm.

Cyclisation of H-(alG)EDLFYQC-NH_2 (**46**)

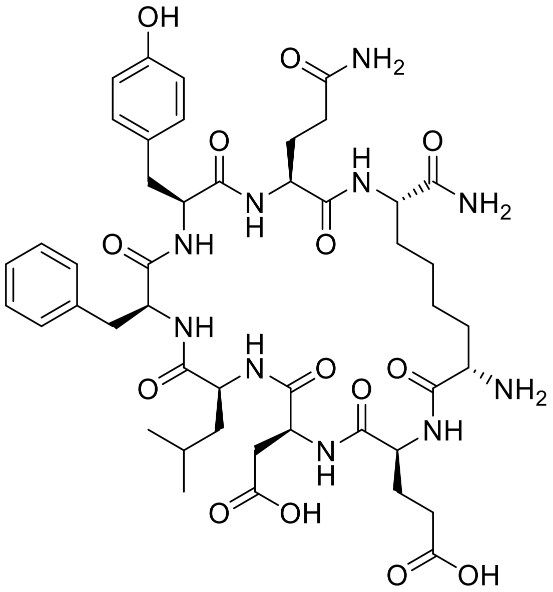

Product **46** was synthesised following the optimised cyclisation protocol using H-(alG)EDLFYQC-NH_2 (**36**, 2 mg, 1.97 µmol). After analysis the remaining solution (1.88 µmol) was purified using semi-preparative HPLC (15-80% B over 30 minutes) and fractions containing the desired product lyophilised to yield the title compound as a fluffy white solid (1.2 mg, 1.22 µmol, 65% yield).

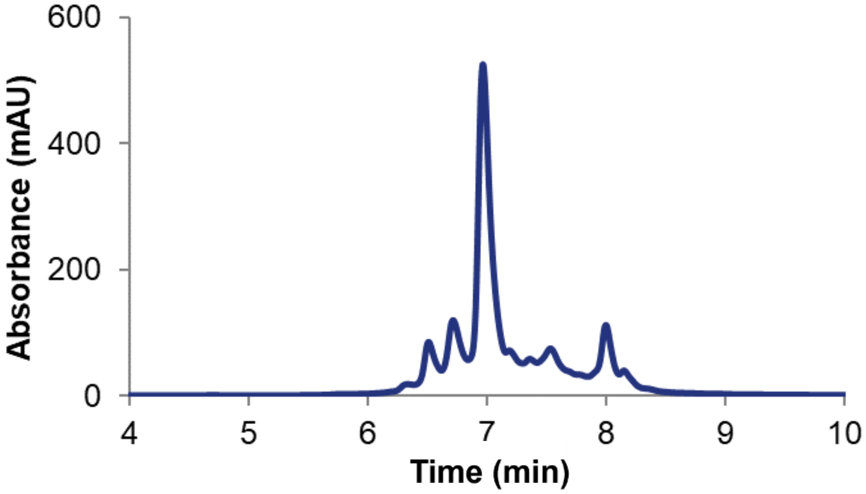

Figure S134. Crude analytical HPLC trace of H-(alG)EDLFYQC-NH_2 (**46**) macrocyclisation; analytical gradient 10-50% B over 10 minutes, 210 nm.

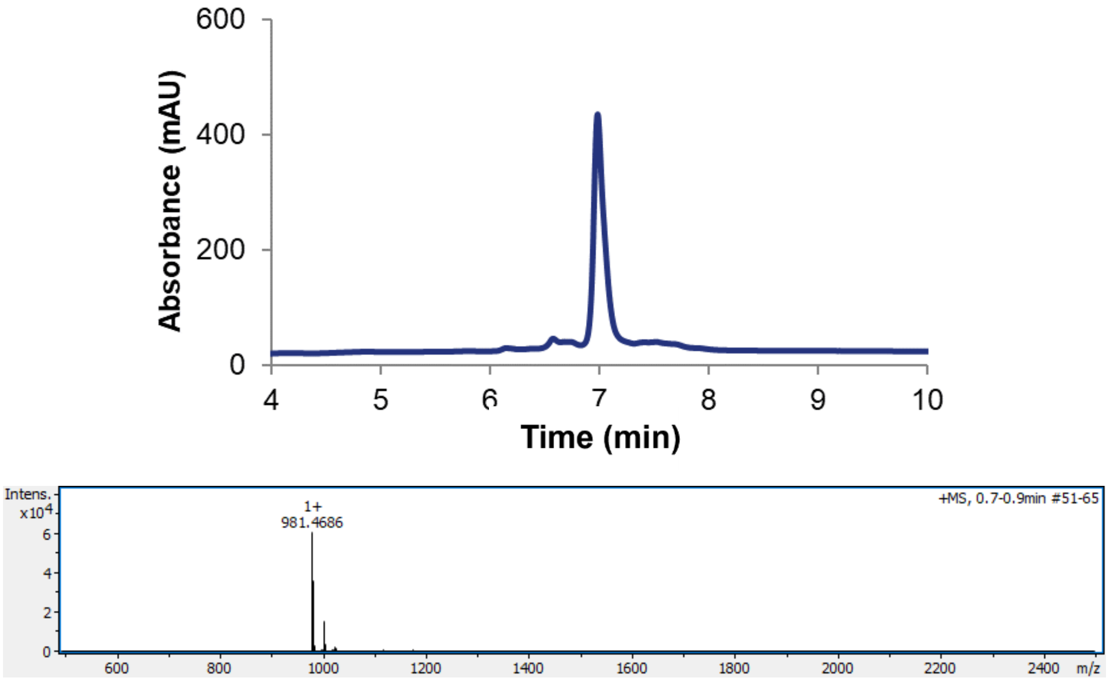

Figure S135. Analytical HPLC trace and ESI MS of cyclised H-(alG)EDLFYQC-NH_2 (**46**); analytical gradient 10-50% B over 10 minutes, 210 nm. Calculated mass $[M+H]^+$: 981.46; observed mass $[M+H]^+$: 981.47.

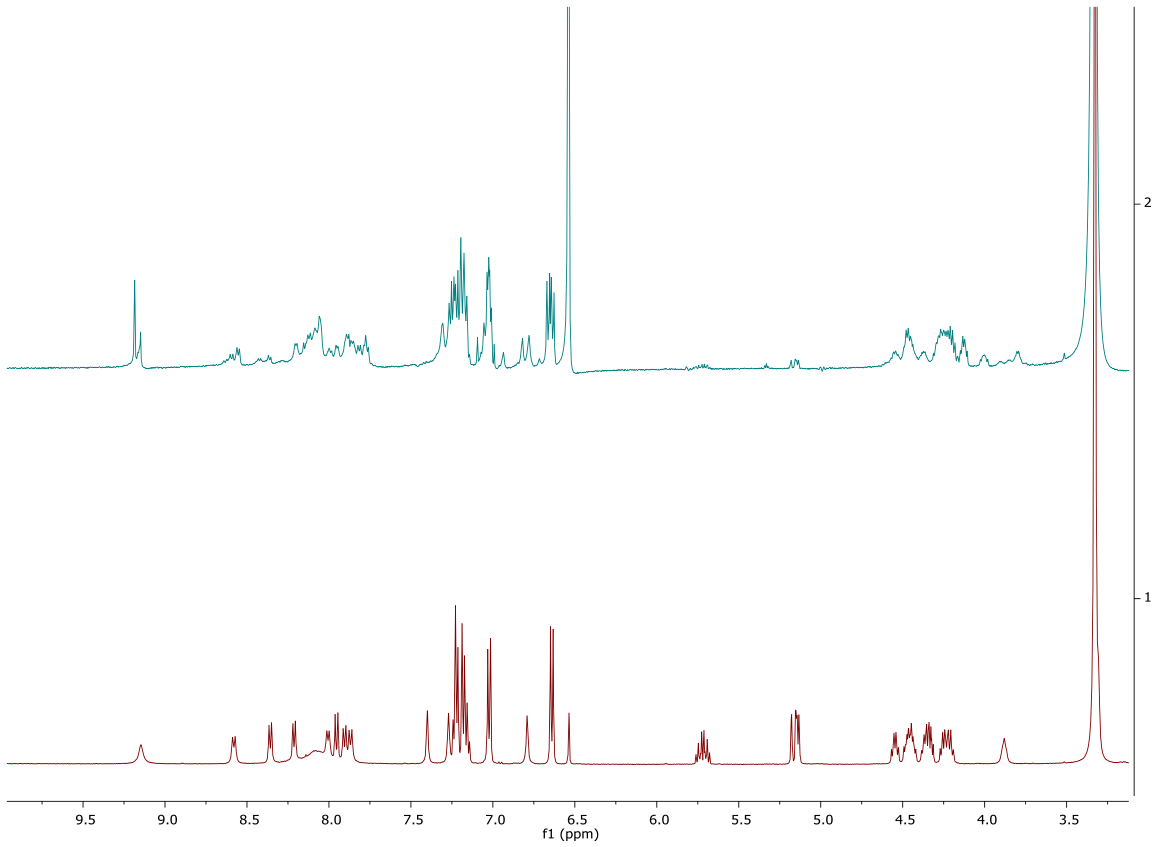

Figure S136. NMR overlay of starting peptide H-(alG)EDLFYQC-NH_2 (**36**, bottom) and desulfurised/cyclised product (**46**, top), showing disappearance of allylic proton peaks at 5.75 and 5.20 ppm.

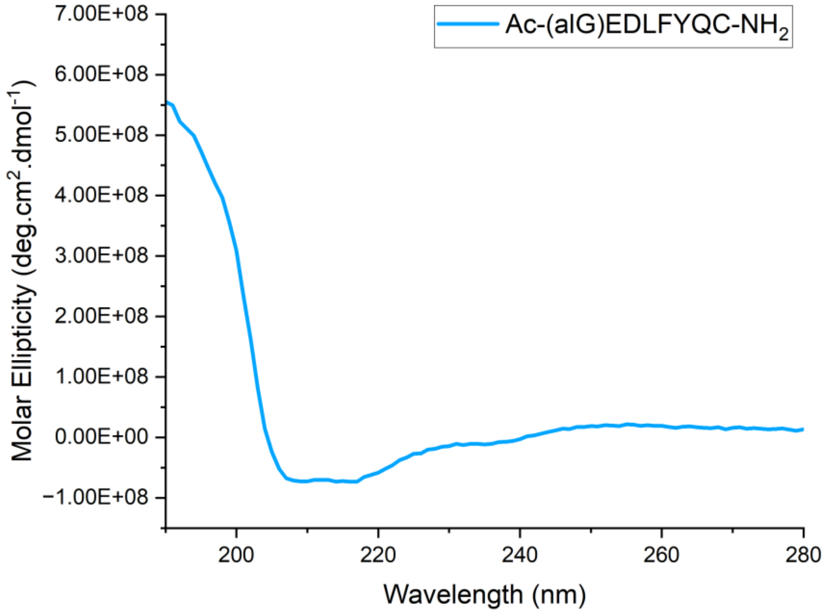

Figure S137. Circular dichroism of cyclised product **46.**

Cyclisation of H-(alG)DKFNHEC-NH_2 (**47**)

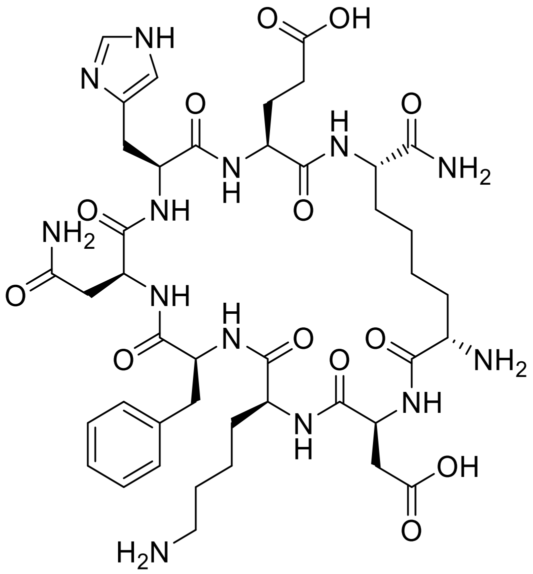

Product **47** was synthesised following the optimised cyclisation protocol using H-(alG)DKFNHEC-NH_2 (**37**, 2 mg, 2.02 µmol). After analysis the remaining solution (1.93 µmol) was purified using semi-preparative HPLC (2-60% B over 30 minutes) and fractions containing the desired product lyophilised to yield the title compound as a fluffy white solid (1.3 mg, 1.36 µmol, 70% yield).

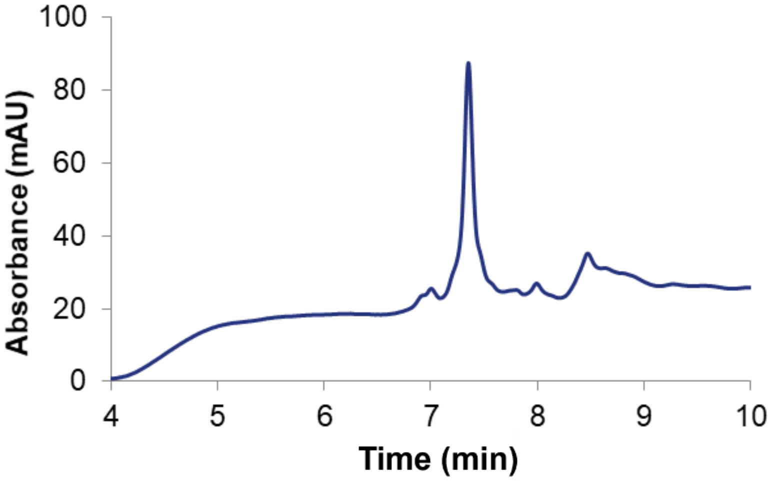

Figure S138. Crude analytical HPLC trace of H-(alG)DKFNHEC-NH_2 (**47**) macrocyclisation; analytical gradient 2-25% B over 10 minutes, 210 nm.

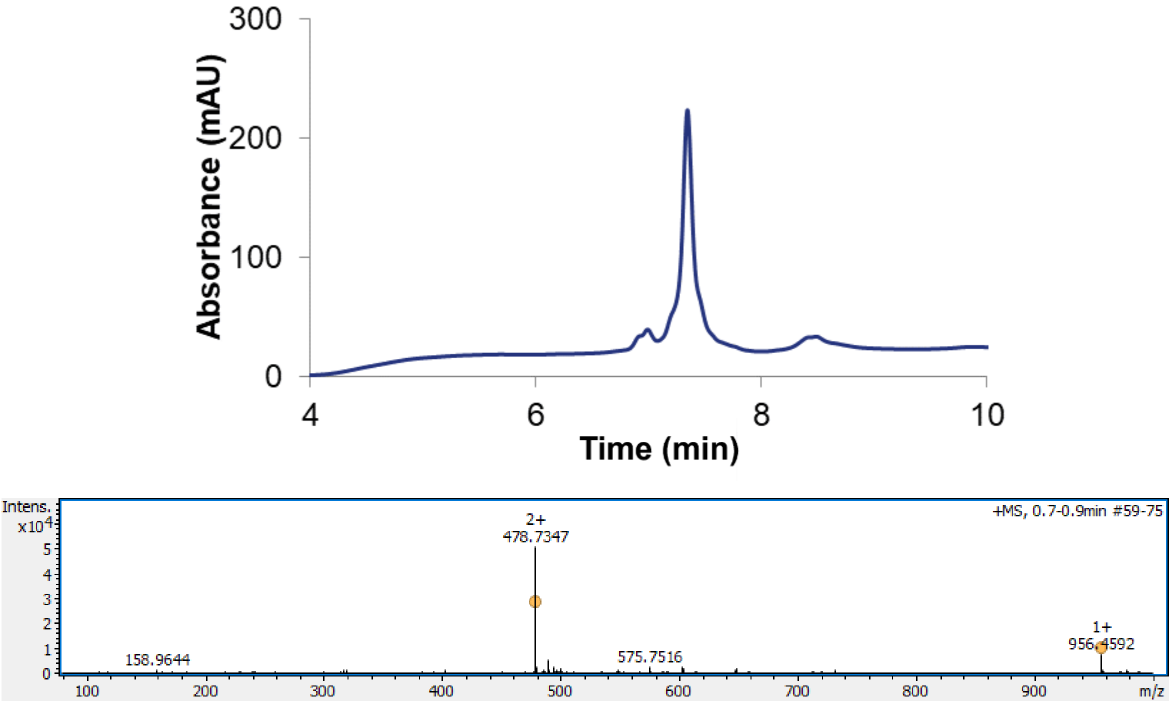

Figure S139. Analytical HPLC trace and ESI MS of cyclised H-(aIG)DKFNHEC-NH_2 (**47**); analytical gradient 2-25% B over 10 minutes, 210 nm. Calculated mass $[M+H]^+$: 956.46, $[M+2H]^{2+}$: 478.73; observed mass $[M+H]^+$: 956.46, $[M+2H]^{2+}$: 478.73.

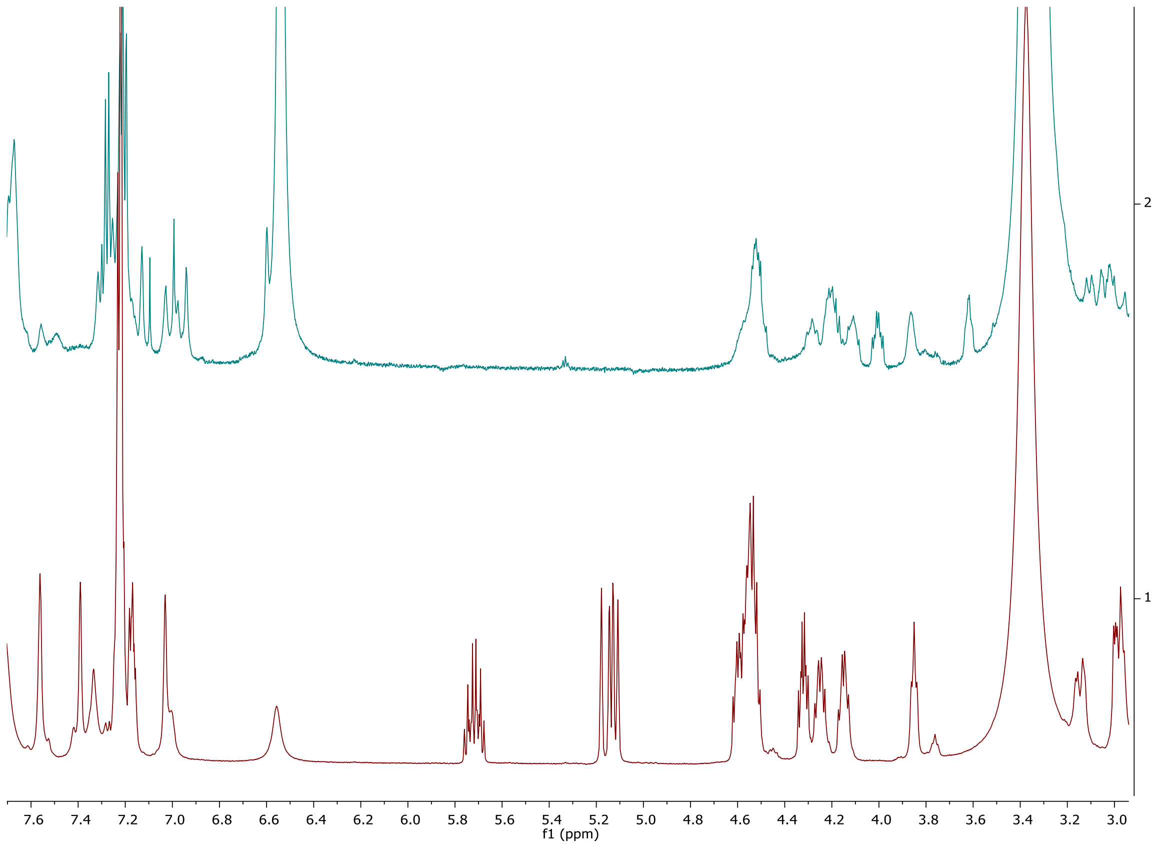

Figure S140. NMR comparison of cyclised H-(alG)DKFNHEC-NH_2 (**47**, top) and the starting peptide (**37**, bottom), showing disappearance of allylic protons at 5.71 and 5.15 ppm.

Cyclization of Ac-(alG)QVGDSN$_L$C-NH_2 (**48**)

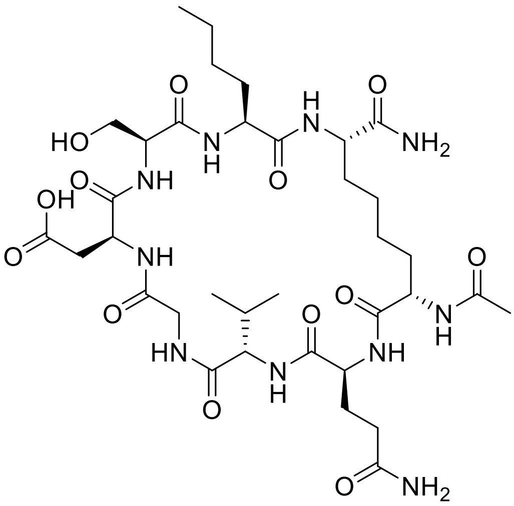

Product **48** was synthesised following the optimised cyclisation protocol using H-(alG)QVGDSN$_L$C-NH_2 (**38**, 1.5 mg, 1.78 µmol). After analysis the remaining solution was purified using semi-preparative HPLC (2-95% B over 30 minutes) and fractions containing the desired product lyophilised to yield the title compound as a fluffy white solid (1.1 mg, 1.33 µmol, 76% yield).

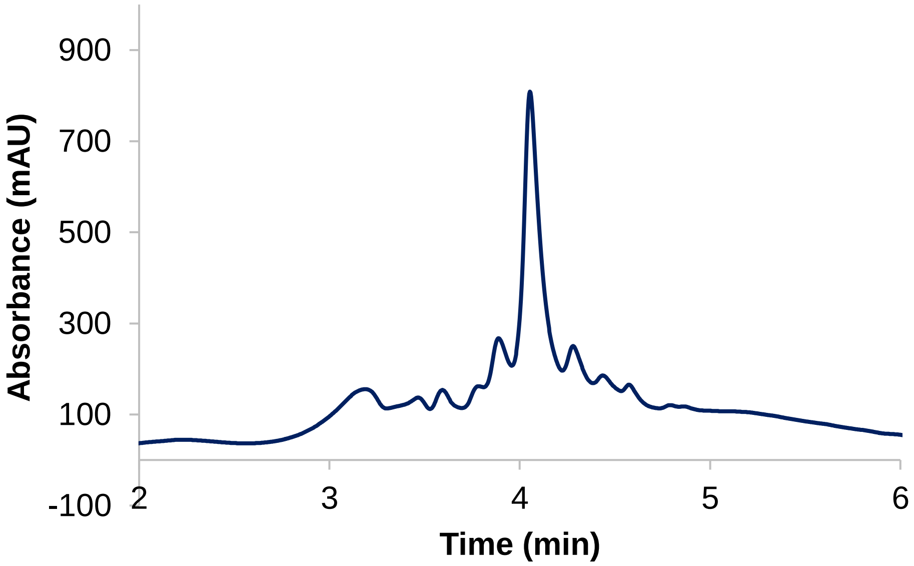

Figure S141. Crude analytical HPLC trace of Ac-(alG)QVGDSN$_L$C-NH_2 (**48**) cyclisation; analytical gradient 2-95% B over 5 minutes, 210 nm.

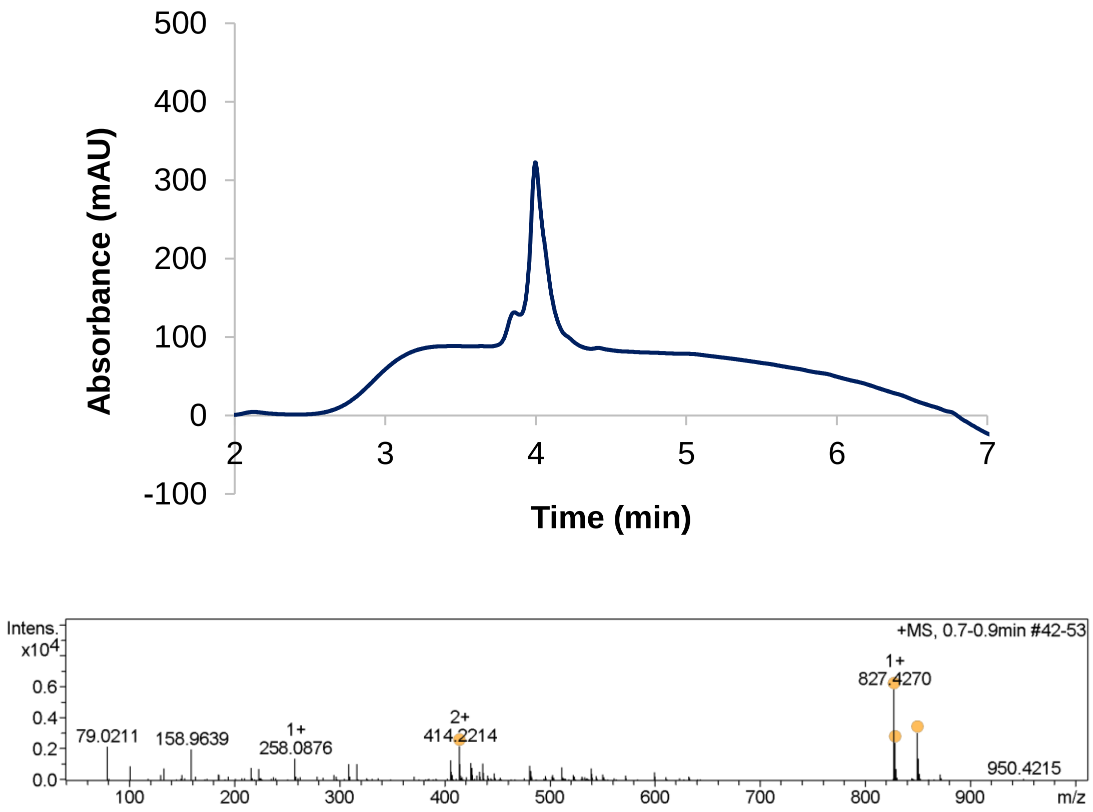

Figure S142. Analytical HPLC trace and ESI MS of cyclised Ac-(alG)QVGDSN$_L$C-NH$_2$ (**48**); analytical gradient 2-95% B over 5 minutes, 210 nm. Calculated mass $[M+H]^+$: 827.41; observed mass $[M+H]^+$: 827.43.

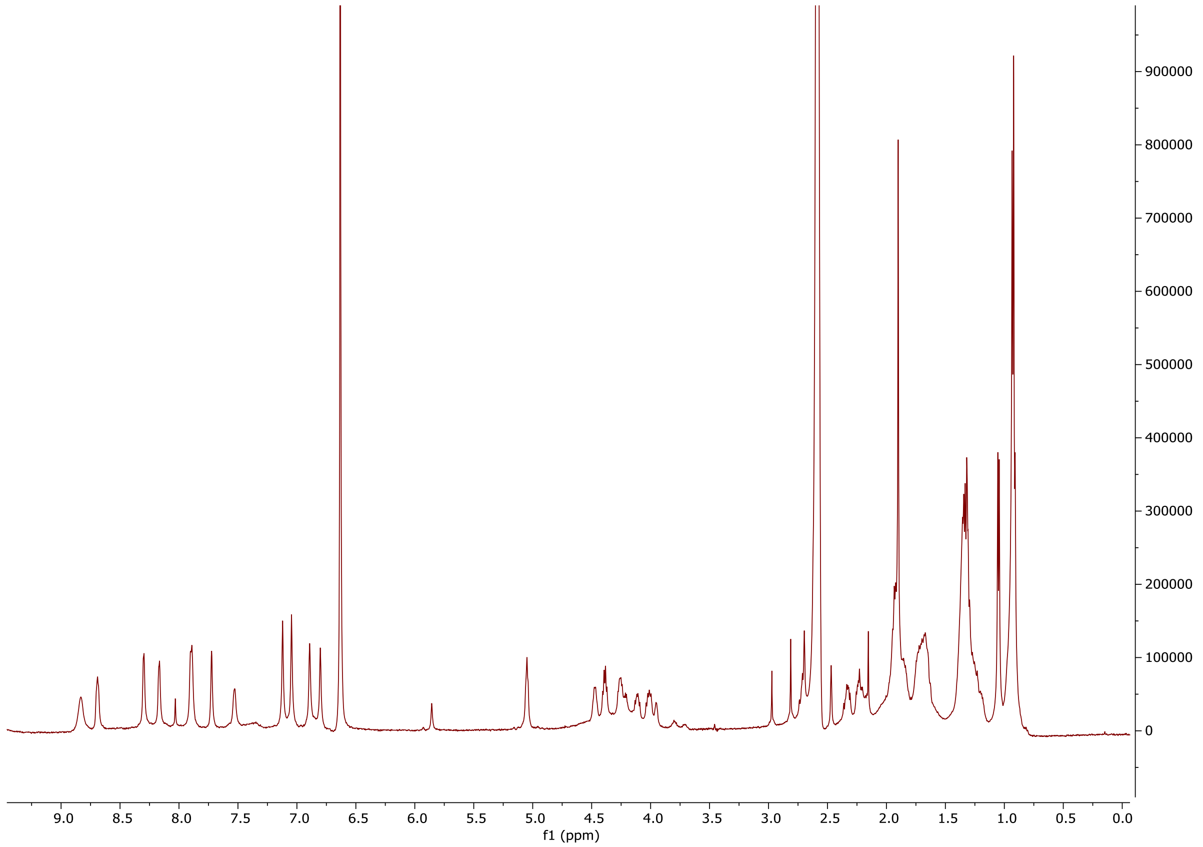

Figure S143. ^{1}H NMR spectra of cyclised Ac-(alG)QVGDSN$_L$C-NH$_2$ (**48**) absence of allylic protons at 5.71 and 5.15 ppm.

Failed cyclisation of H-(alG)YEPLAWHISKEYC-NH2 (**50**)

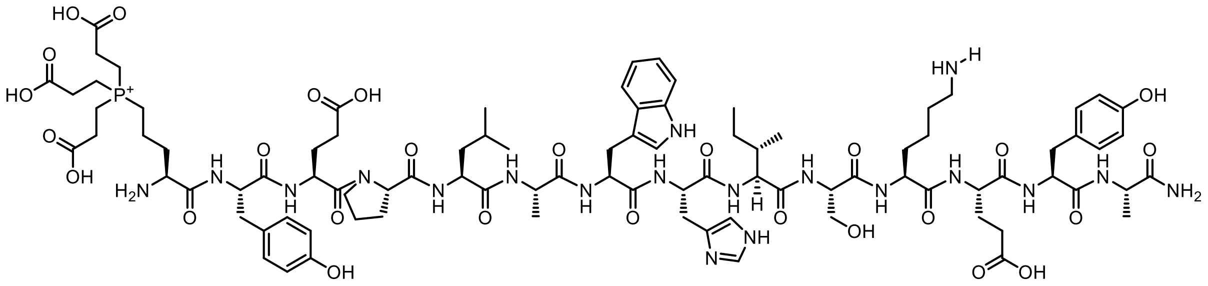

Peptide **49** was subjected to the optimised cyclisation conditions (2 mg, 1.15 µmol); the reaction produced an inseparable mixture of cyclised and desulfurised linear byproduct.

The reaction was repeated using 5 mol% of the Ir PC on 2 mg, 1.15 µmol of peptide **49**. After analysis the remaining solution (1.06 µmol) was purified using semi-preparative HPLC (10-80% B over 30 minutes) and fractions containing the desired product lyophilised to yield the title compound as a fluffy white solid (1.2 mg, 0.614 µmol, 58% yield).

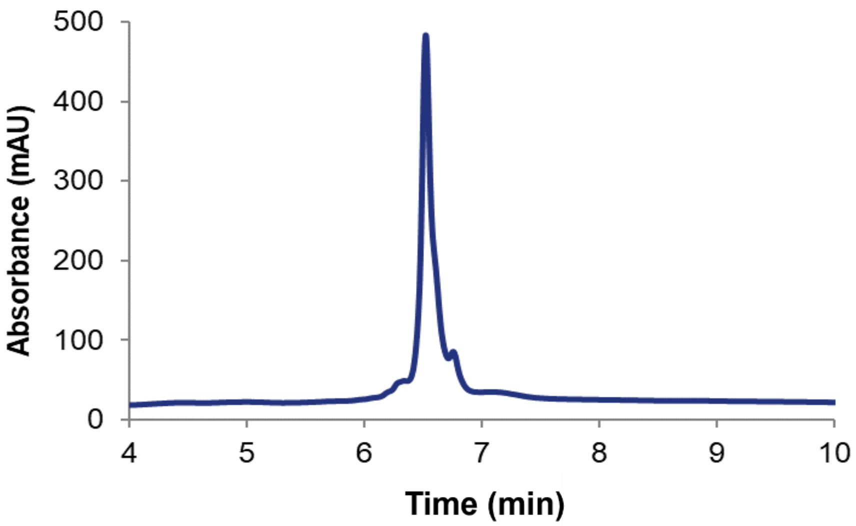

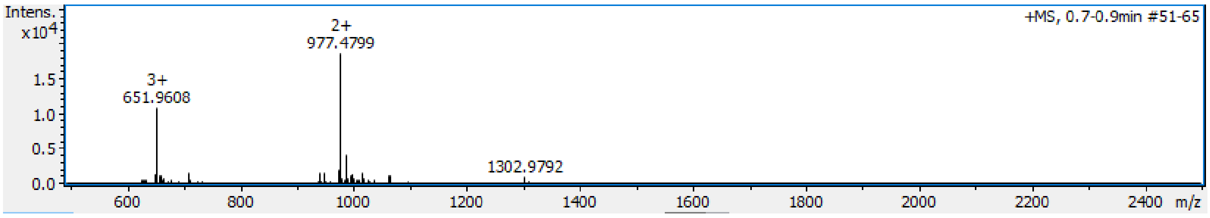

Figure S144. Pure analytical HPLC trace and ESI MS of desulfurised and phospha-Michael addition product **50**. Analytical gradient 10-50% B over 10 minutes, 210 nm. Calculated Mass $[M+2H]^{2+}$: 977.4678, $[M+3H]^{3+}$: 651.9811. Observed Mass $[M+2H]^{2+}$: 977.4799, $[M+3H]^{3+}$: 651.9608.

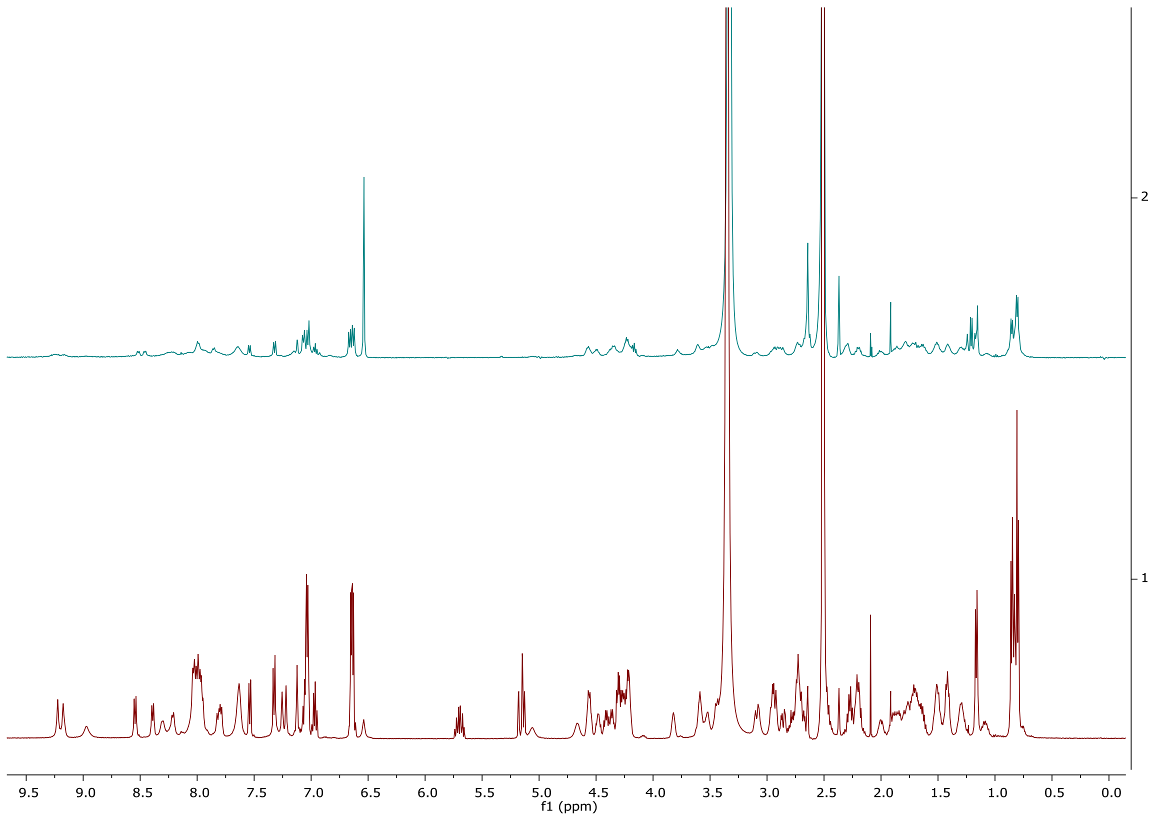

Figure S145. Pure starting material H-(alG)YEPLAWHISKEYC-NH_2 (**49**) (bottom) and purified product **50** (top) NMR comparison, showing disappearance of allylic protons.

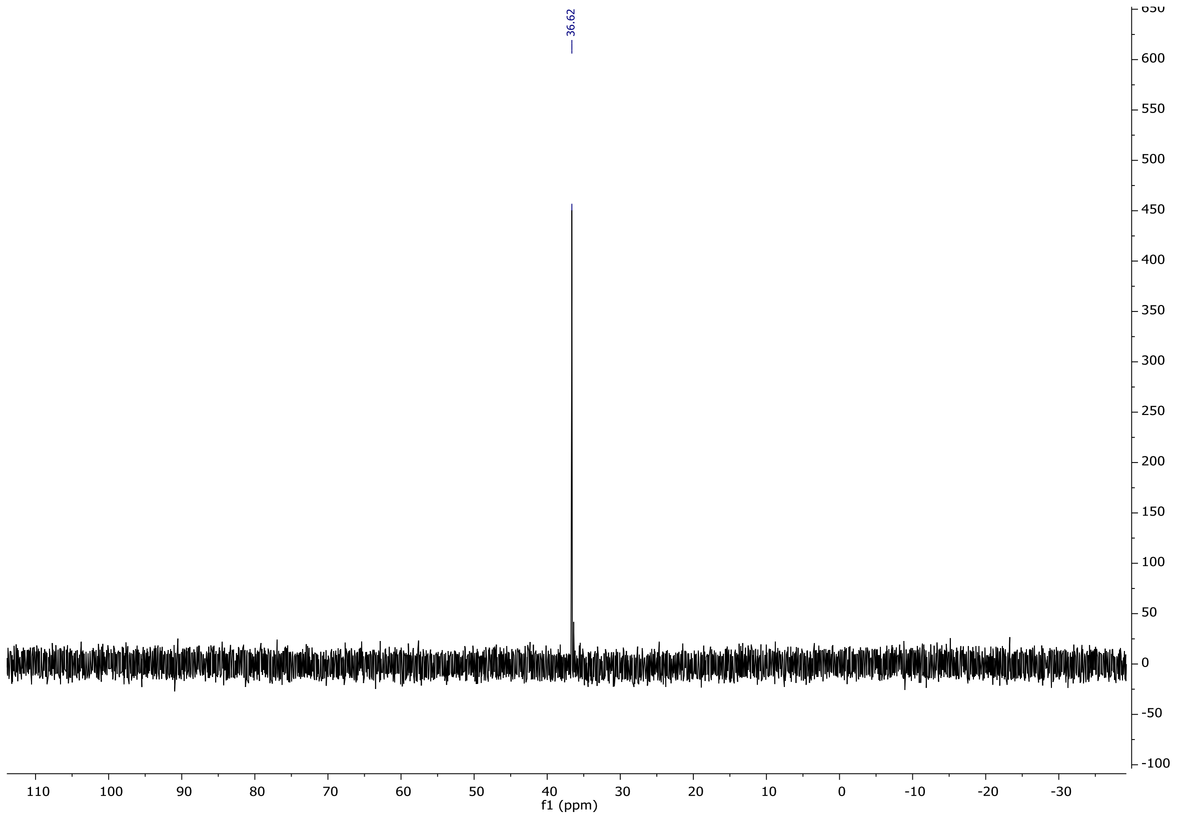

Figure S146. ^{31}P NMR (162 MHz, DMSO) δ 36.62 of the TCEP-Michael addition (desulfurised) product **50**.

Cyclisation of H-CYIQN(alG)PLG-NH_2 (carba-oxytocin) (**53**)

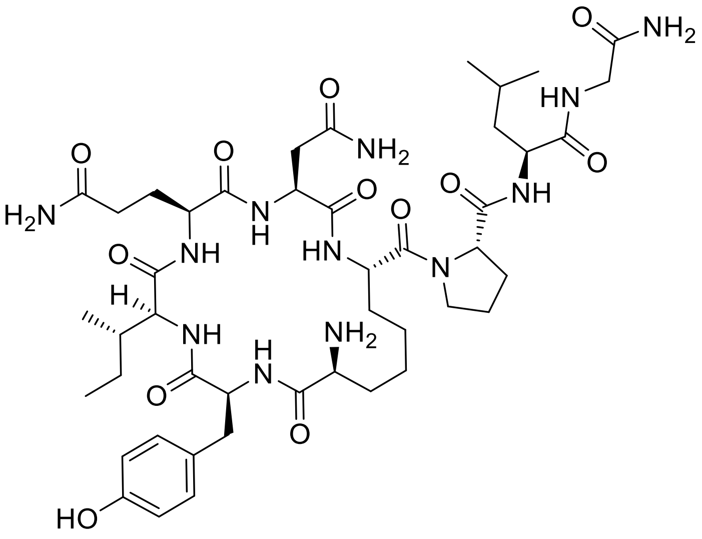

Product **53** was synthesised following the optimised cyclisation protocol using H-CYIQN(alG)PLG-NH_2 (**52**, 5 mg, 4.98 µmol). After analysis the remaining solution (4.89 µmol) was purified using semi-preparative HPLC (10-70% B over 30 minutes) and fractions containing the desired product lyophilised to yield the uncyclised title compound (1.7 mg, 1.73 µmol, 35% yield) and the cyclised title compound (2.5 mg, 2.54 µmol, 52% yield) as a fluffy white solid.

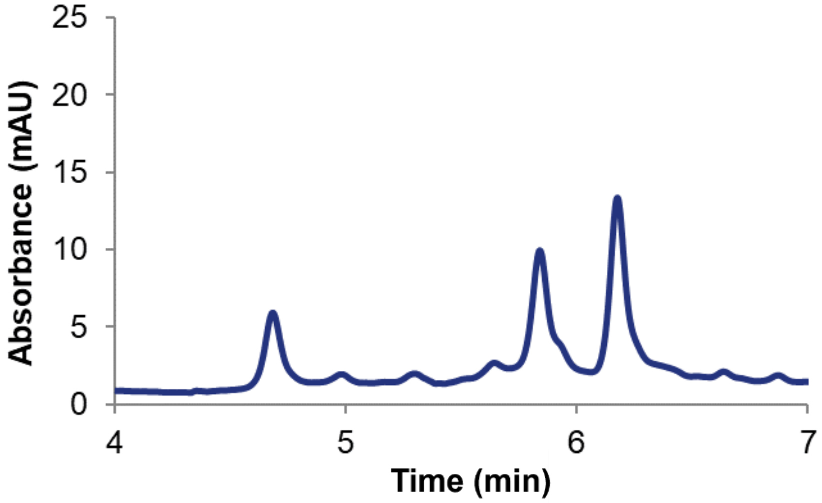

Figure S147. Crude analytical HPLC trace of H-CYIQN(alG)PLG-NH_2 (**53**) cyclisation; analytical gradient 10-50% B over 10 minutes, 210 nm.

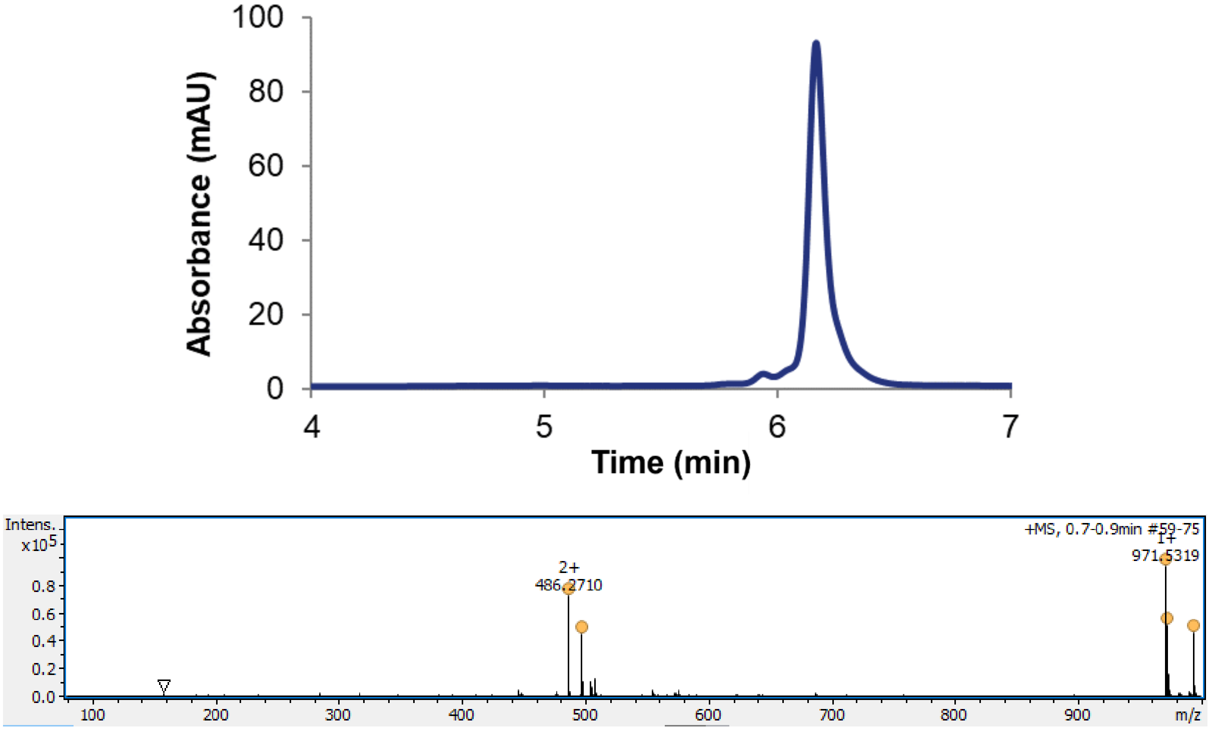

Figure S148. Analytical HPLC trace and ESI MS of cyclised H-CYIQN(alG)PLG-NH_2 (**53**); analytical gradient 10-50% B over 10 minutes, 210 nm. Calculated mass $[M+H]^+$: 971.53, $[M+2H]^{2+}$: 486.27; observed mass $[M+H]^+$: 971.53, $[M+2H]^{2+}$: 486.27.

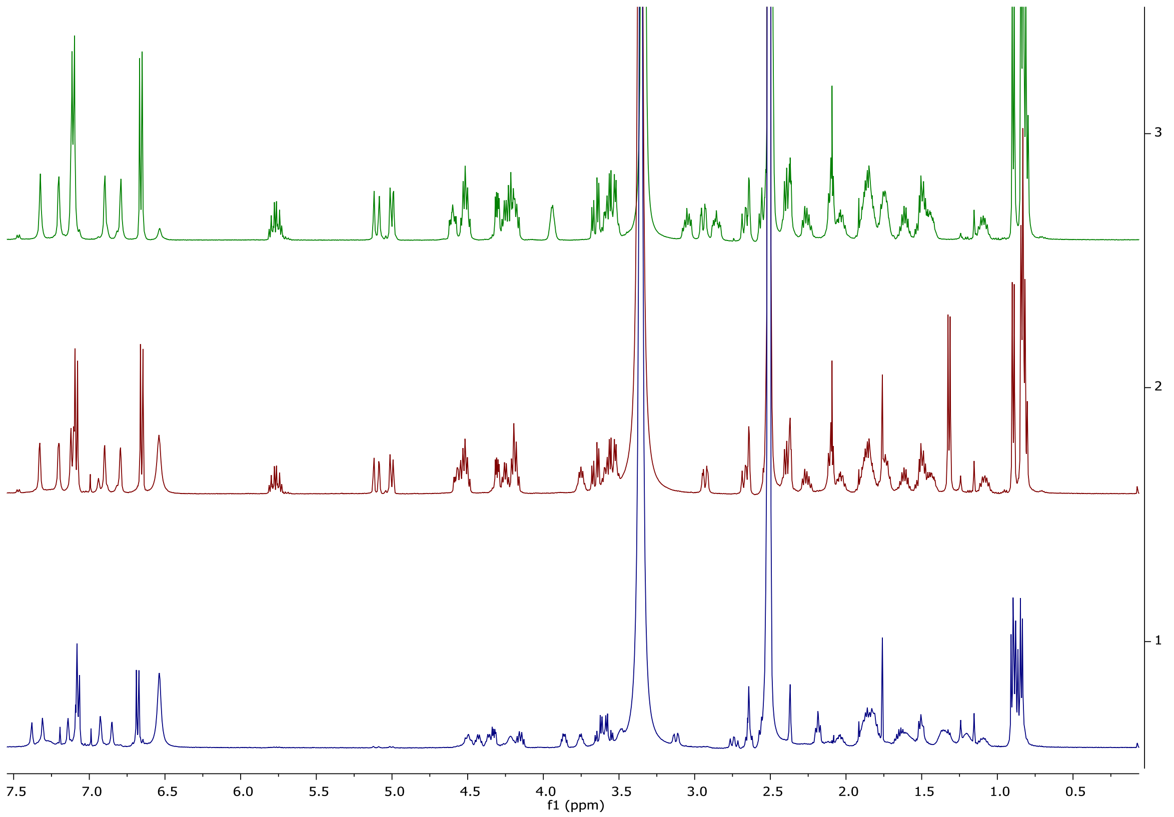

Figure S149. NMR overlay comparing oxytocin model starting peptide H-CYIQN(alG)PLG-NH_2 (**52**, top), desulfurised-uncyclised peptide (middle) and cyclised product (**53**, bottom).

Cyclisation of Ac-HLA(alG)VGDCN$_L$DR-NH$_2$ (**57**)

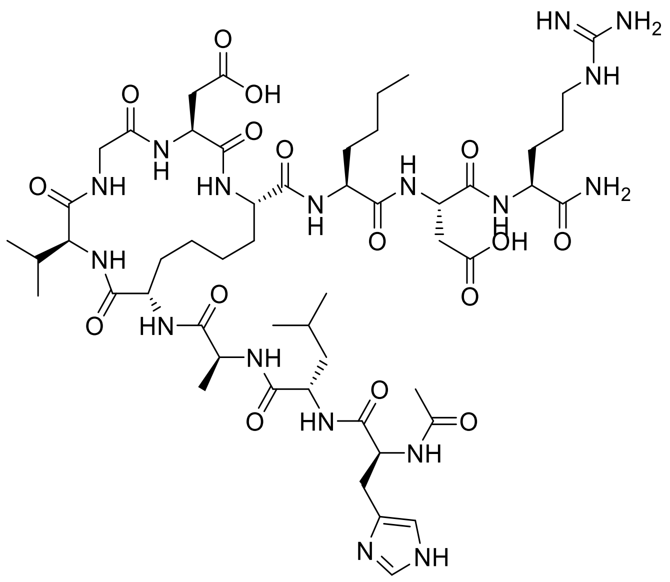

Product **57** was synthesised following the optimised cyclisation protocol using Ac-HLA(alG)VGDCN$_L$DR-NH$_2$ (**54**, 2.0 mg, 1.62 µmol). After analysis the remaining solution was purified using semi-preparative HPLC (2-95% B over 30 minutes) and fractions containing the desired product lyophilised to yield the title compound as a fluffy white solid (0.45 mg, 0.37 µmol, 23% yield).

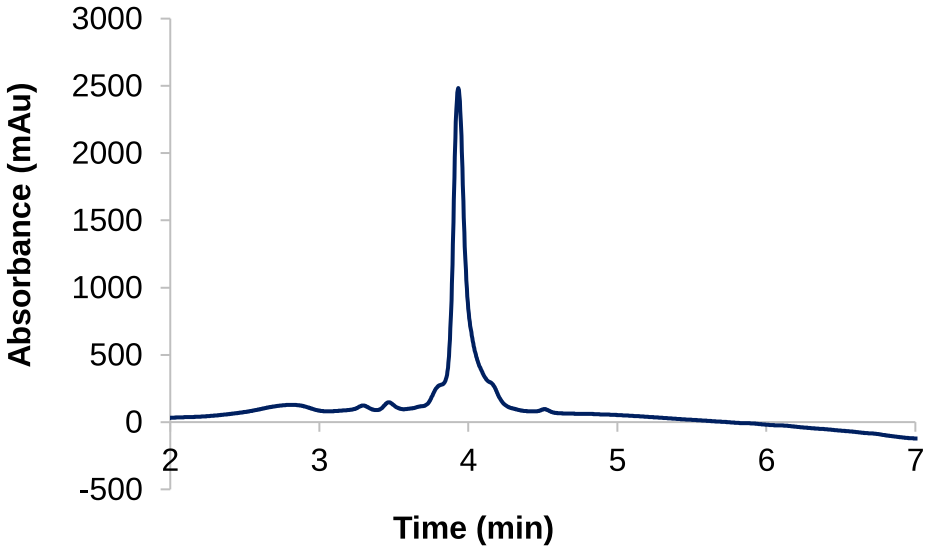

Figure S150. Crude analytical HPLC trace of Ac-HLA(alG)VGDCN$_L$DR-NH$_2$ (**57**) cyclisation; analytical gradient 2-95% B over 5 minutes, 210 nm.

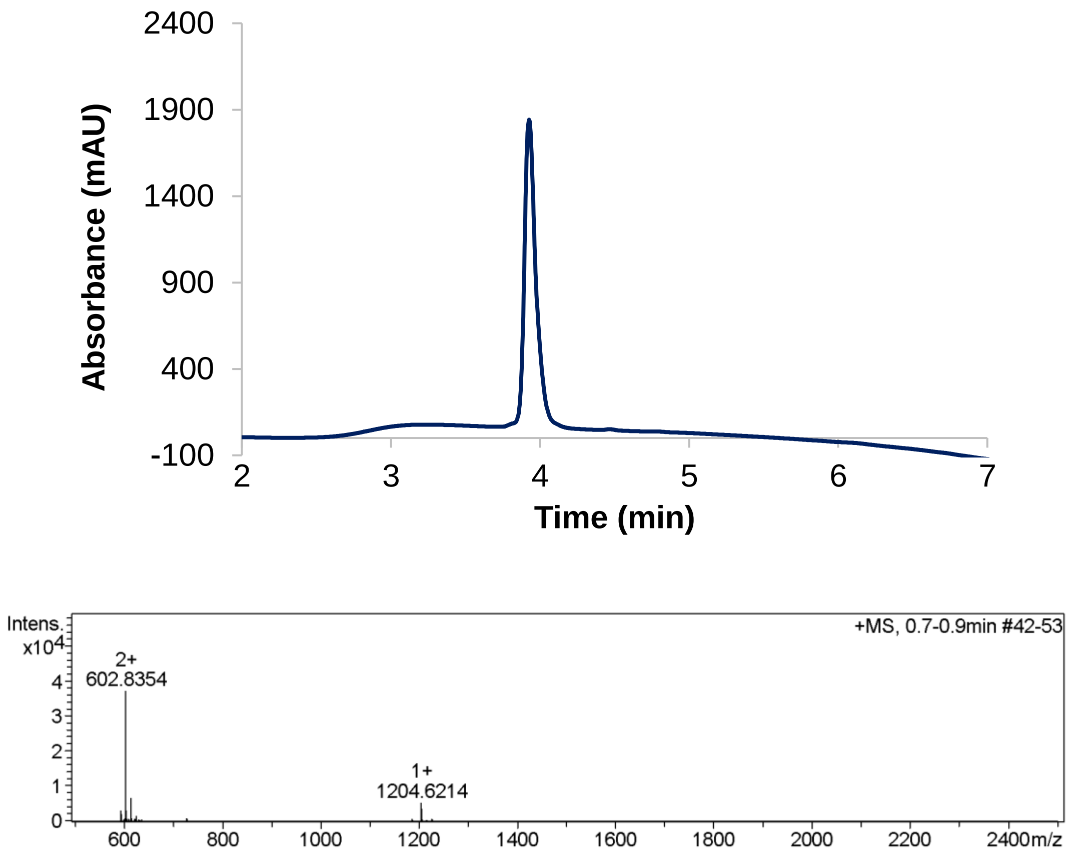

Figure S151. Analytical HPLC trace and ESI MS of cyclised Ac-HLA(alG)VGDCN$_L$DR-NH$_2$ (**57**); analytical gradient 2-95% B over 5 minutes, 210 nm. Calculated mass $[M+H]^+$: 1204.63; observed mass $[M+H]^+$: 1204.62.

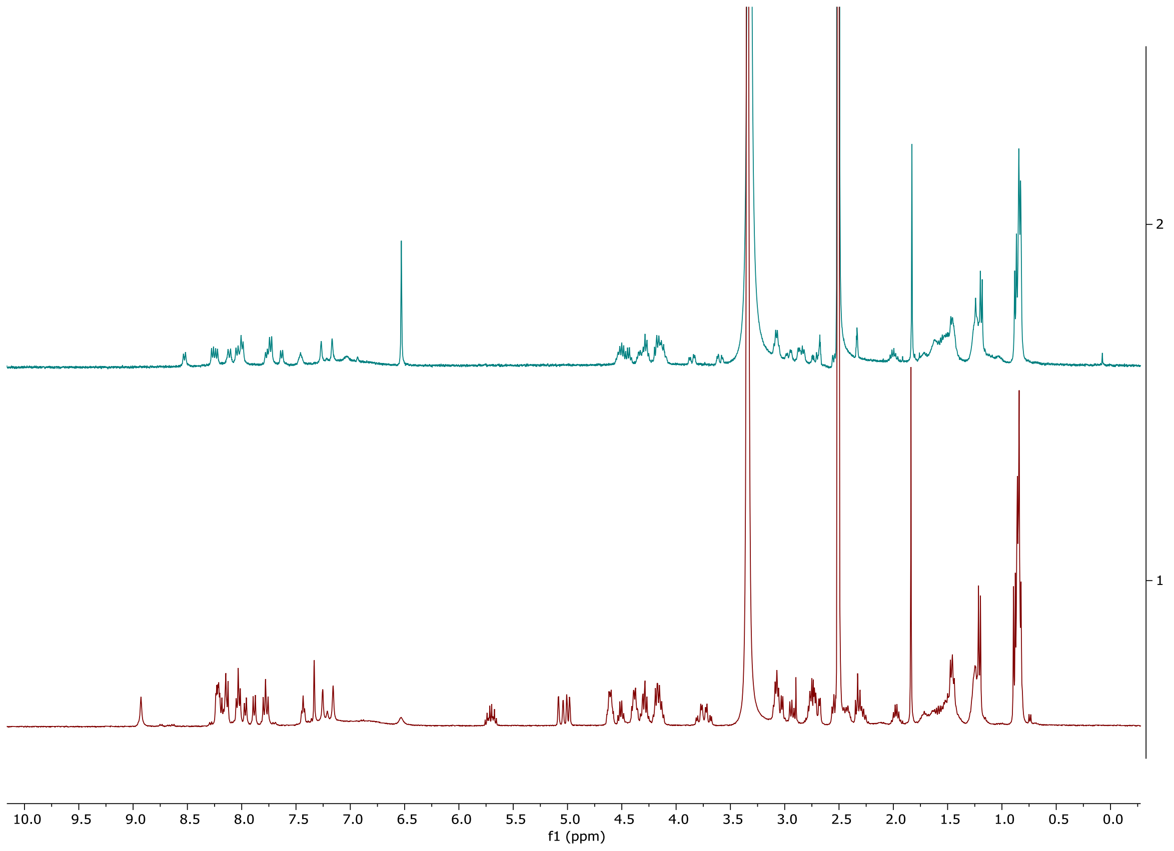

Figure S152. NMR comparison of cyclised Ac-HLA(alG)VGDCN$_L$DR-NH$_2$ (**57**; top) and the starting peptide (**54**, bottom), showing disappearance of allylic protons at 5.71 and 5.05 ppm.

Cyclization of Ac-RHL(alG)QVGDSN$_L$CRSI-NH$_2$ (**58**)

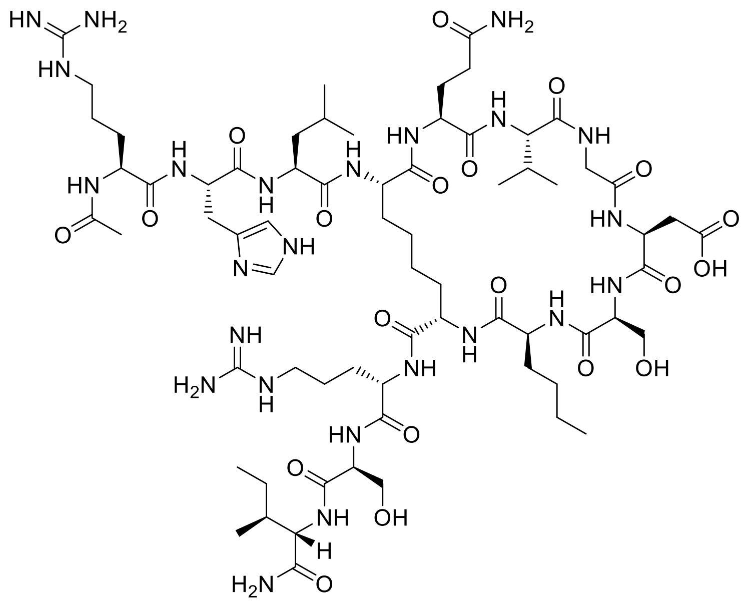

Product **58** was synthesised following the optimised cyclisation protocol using Ac-RHL(alG)QVGDSN$_L$CRSI-NH$_2$ (**55**, 2.0 mg, 1.23 µmol). After analysis the remaining solution was purified using semi-preparative HPLC (2-95% B over 30 minutes) and fractions containing the desired product lyophilised to yield the title compound as a fluffy white solid (0.45 mg, 0.28 µmol, 23% yield).

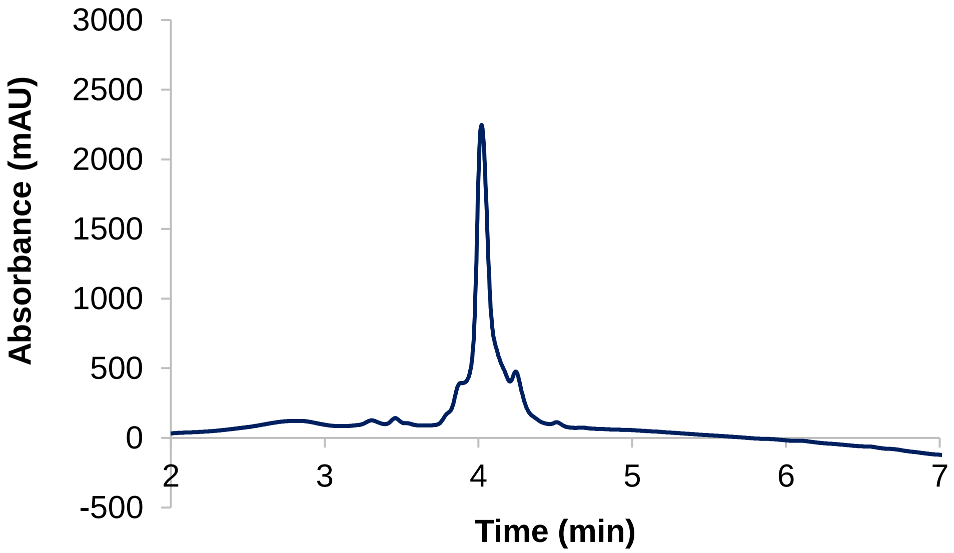

Figure S153. Crude analytical HPLC trace of Ac-RHL(alG)QVGDSN$_L$CRSI-NH$_2$ (**58**) cyclisation; analytical gradient 2-95% B over 5 minutes, 210 nm.

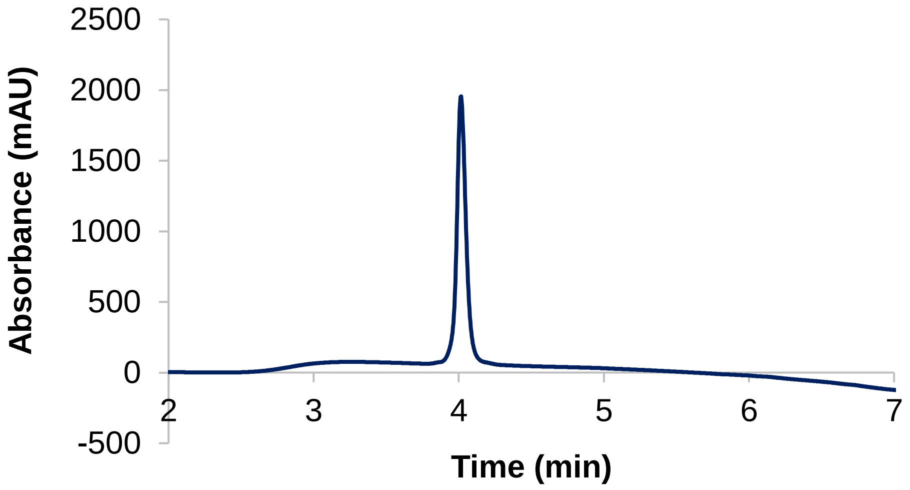

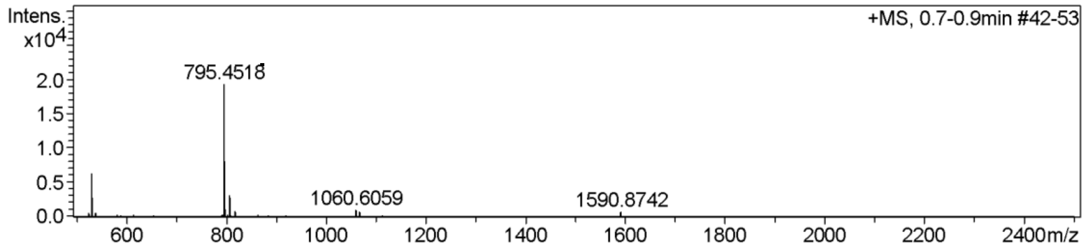

Figure S154. Analytical HPLC and ESI MS of cyclic peptide Ac-RHL(alG)QVGDSN$_L$CRSI-NH$_2$ (**58**) after purification with a gradient of 2-95% B over 5 mins, 210 nm. Calculated mass $[M+H]^{1+}$: 1589.88; observed mass $[M+H]^{1+}$: 1590.87.

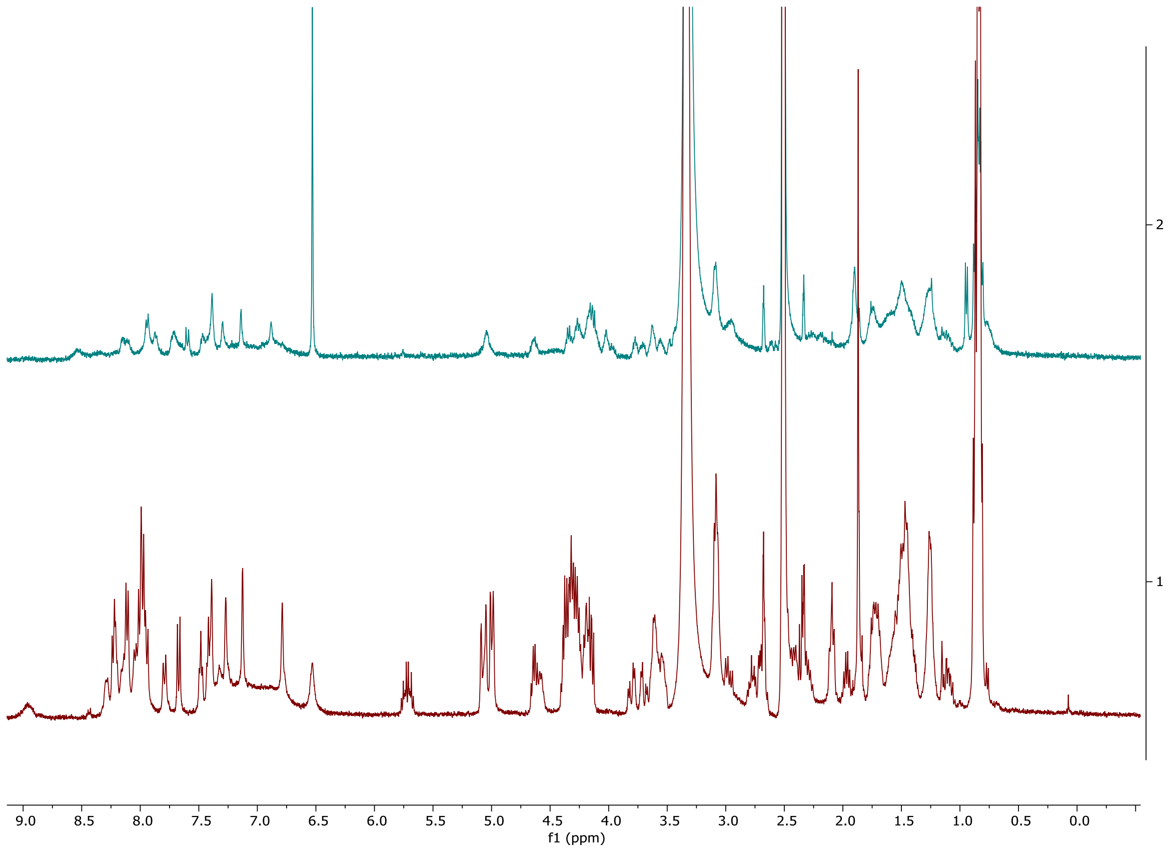

Figure S155. NMR comparison of cyclised Ac-RHL(alG)QVGDSN$_L$CRSI-NH$_2$ (**58**; top) and the starting peptide (**55**, bottom), showing disappearance of allylic protons at 5.71 and 5.05 ppm.

Cyclisation of Ac-EDIIRNIARHLA(alG)VGDCN$_L$DRSIW-NH$_2$ (**59**)

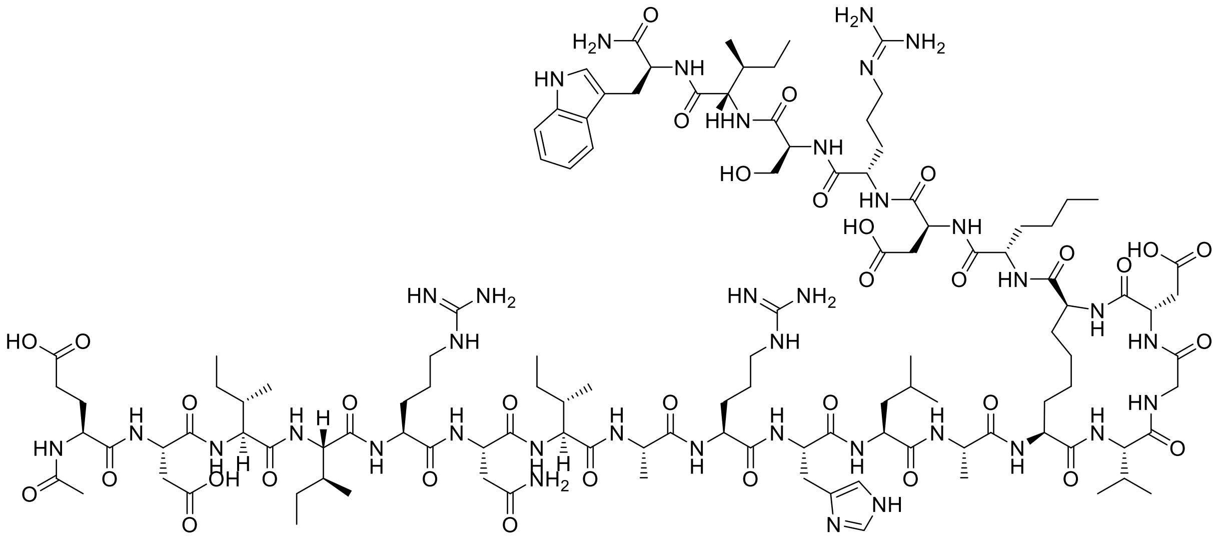

Product **59** was synthesised following the optimised cyclisation protocol using Ac-EDIIRNIARHLA(alG)VGDCN$_L$DRSIW-NH$_2$ (**56**, 4.5 mg, 1.66 µmol). After analysis the remaining solution (1.61 µmol) was purified using semi-preparative HPLC (30-100% B over 30 minutes) and fractions containing the desired product lyophilised to yield the title compound as a fluffy white solid (2.1 mg, 0.786 µmol, 49% yield).

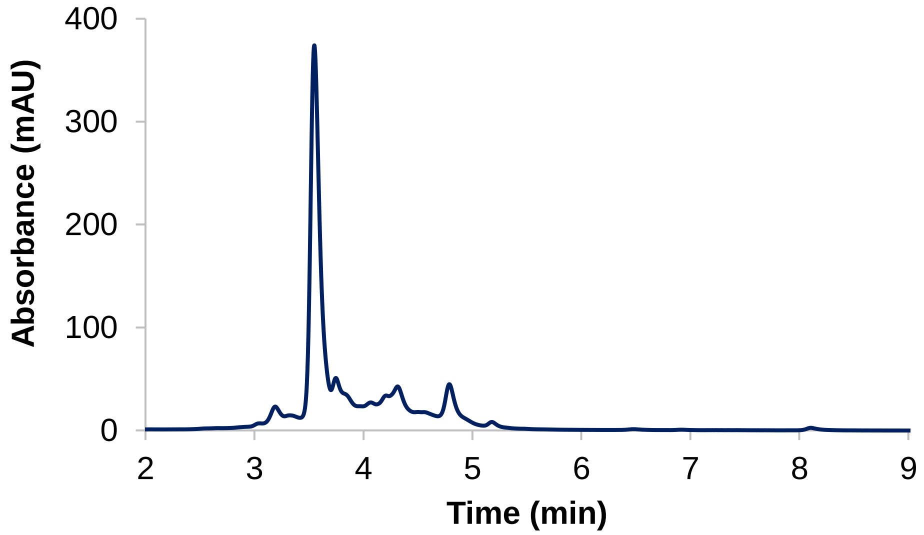

Figure S156. Crude analytical HPLC trace of Ac-EDIIRNIARHLA(alG)VGDCN$_L$DRSIW-NH$_2$ (**59**); analytical gradient 30-100% B over 10 minutes, 280 nm.

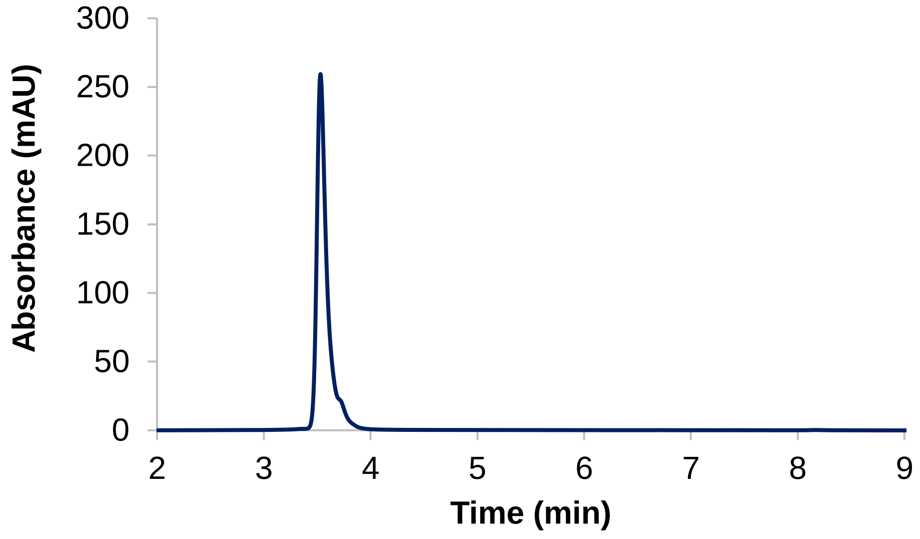

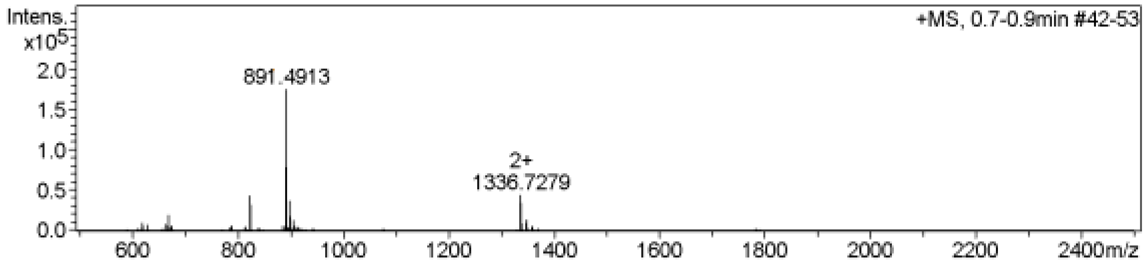

Figure S157. Analytical HPLC trace and ESI MS of purified cyclisation of Ac-EDIIRNIARHLA(alG)VGDCN$_L$DRSIW-NH$_2$ (**59**); analytical gradient 30-100% B over 10 minutes, 280 nm. Calculated mass $[M+2H]^{2+}$: 1336.73; observed mass $[M+2H]^{2+}$ 1336.73.

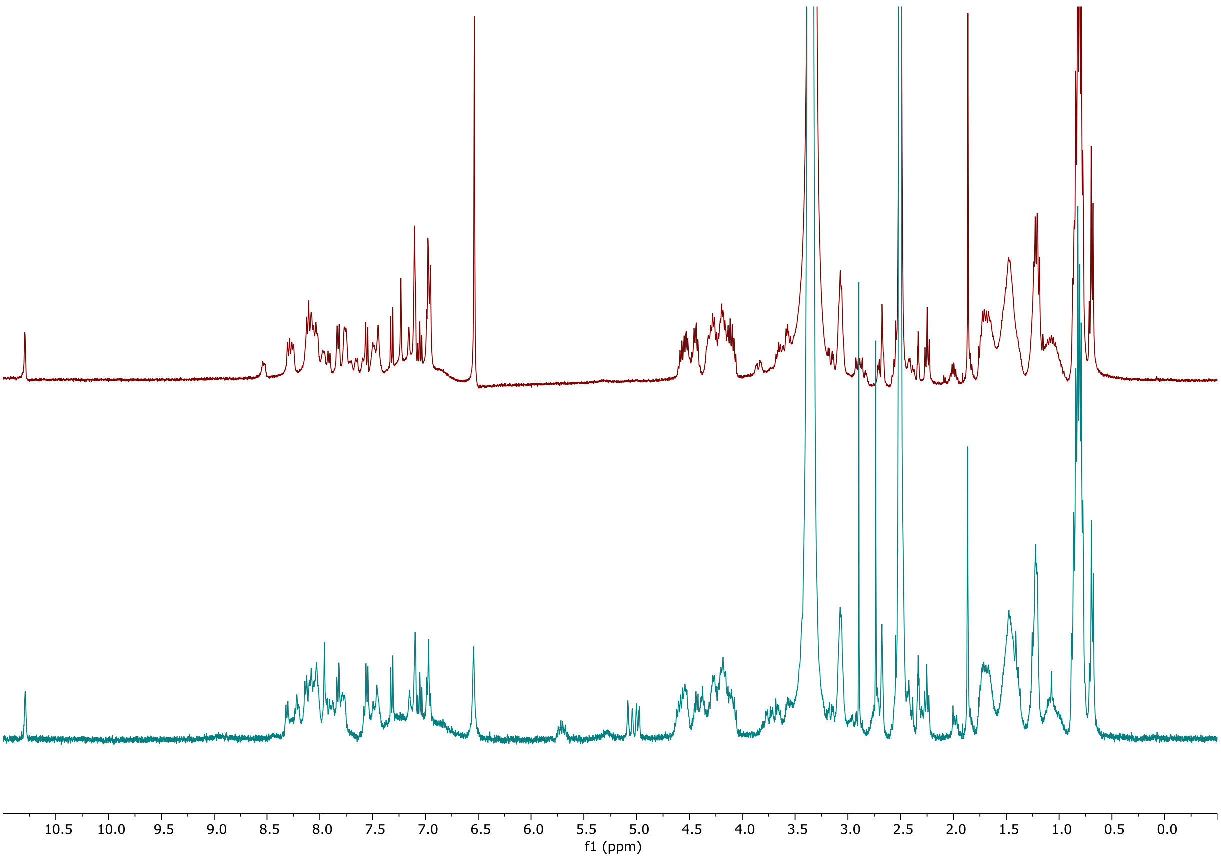

Figure S158. NMR comparison of cyclised Ac-EDIIRNIARHLA(alG)VGDCN$_L$DRSIW-NH$_2$ (**59**, top) and the starting peptide (**56**, bottom) showing disappearance of allylic protons at 5.70 and 5.02 ppm.

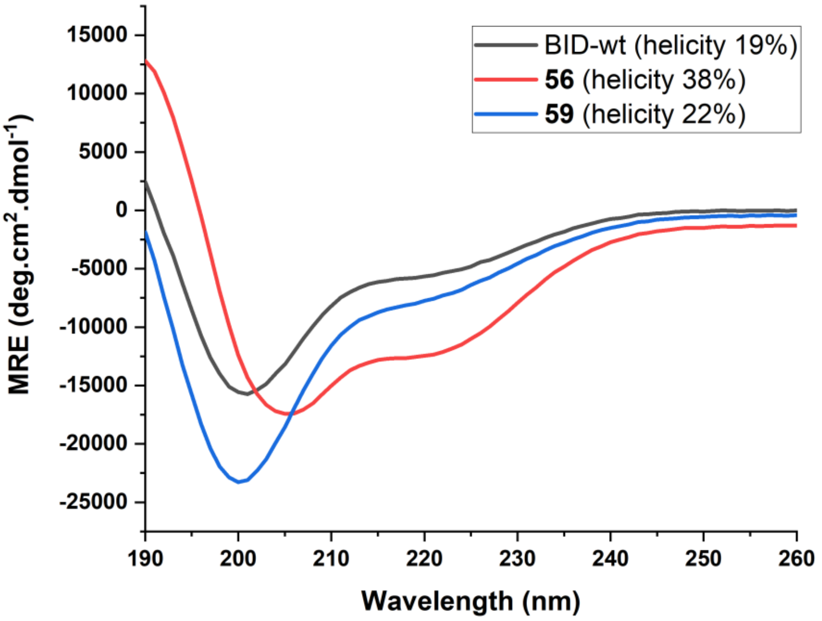

Figure S159. Circular dichroism of linear peptide **56**, cyclic peptide **59** and the native BID sequence (EDIIRNIARHLAQVGDSN$_L$DRSIW).

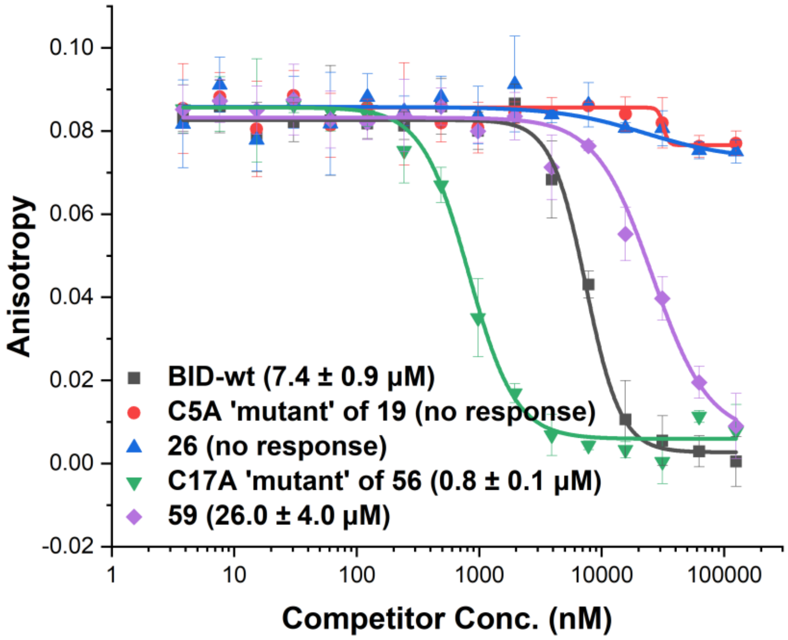

Figure S160. Fluorescence anisotropy measurements for binding of BID-wt (EDIIRNIARHLAQVGDSN$_L$DRSIW), C5A 'mutant' of **19**, cyclic **26**, C17A 'mutant' of **56** and cyclic **59** to MCL-1.

References

[1] P. Zhang, M. Walko, A. J. Wilson, *Chem. Commun.* **2023**, *59*, 1697-1700.

[2] J. A. Miles, D. J. Yeo, P. Rowell, S. Rodriguez-Marin, C. M. Pask, S. L. Warriner, T. A. Edwards, A. J. Wilson, *Chem. Sci.* **2016**, *7*, 3694-3702.

[3] J. M. Fletcher, K. A. Horner, G. J. Bartlett, G. G. Rhys, A. J. Wilson, D. N. Woolfson, *Chem. Sci.* **2018**, *9*, 7656-7665.

[4] M. P. Glenn, L. K. Pattenden, R. C. Reid, D. P. Tyssen, J. D. Tyndall, C. J. Birch, D. P. Fairlie, *J. Med. Chem.* **2002**, *45*, 371-381.

[5] Z. Sun, W. Ma, Y. Cao, T. Wei, X. Mo, H. Y. Chow, Y. Tan, C. H. Cheung, J. Liu, H. K. Lee, E. C. Tse, H. Liu, X. Li, *Chem* **2022**, *8*, 2542-2557.

[6] X.-F. Gao, J.-J. Du, Z. Liu, J. Guo, *Org. Lett.* **2016**, *18*, 1166-1169.
